# Computational and Memory Efficiency in Heartbeat Rate Detection: A Review of ECG and PPG Techniques

**DOI:** 10.3390/s26082409

**Published:** 2026-04-14

**Authors:** Manuel Merino-Monge, Clara Lebrato-Vázquez, Juan Antonio Castro-García, Gemma Sánchez-Antón, Alberto Jesús Molina-Cantero

**Affiliations:** Departamento de Tecnología Electrónica, Escuela Técnica Superior de Ingeniería Informática, Universidad de Sevilla, 41012 Sevilla, Spain; clebrato@us.es (C.L.-V.); jacastro@us.es (J.A.C.-G.); gemma@us.es (G.S.-A.); almolina@us.es (A.J.M.-C.)

**Keywords:** heart rate, computational efficiency, memory footprint, wearable devices, embedded signal processing, ECG, PPG

## Abstract

(1) Background: Heartbeat detection from electrocardiogram (ECG) and photoplethysmograph (PPG) signals is widely used in wearable devices for health monitoring, fitness tracking, and stress assessment. While numerous methods have been proposed, their practical suitability depends not only on accuracy but also on computational and memory constraints inherent to resource-limited systems. (2) Methods: A scoping review of 52 studies published between 2017 and 2024 was conducted, covering time-domain, frequency-domain, matrix-based, and machine learning approaches. The methods were evaluated according to estimation accuracy, computational complexity, memory footprint, and suitability for on-device implementation. (3) Results: Time-domain peak detection methods consistently provide high accuracy (minimum of 79.25%, maximum of 99.96%, and median ≥99.69%) for ECG and reliable heart rate estimation for PPG with linear computational complexity, low memory requirements and low energy consumption. Frequency-domain approaches are suitable for average heart rate estimation from PPG but do not preserve inter-beat intervals (error range of [1.07, 6.4] beats per minute (BPM)). Matrix-based and machine learning methods often entail higher computational cost without proportional performance gains in wearable contexts (error range of [1.07, 6.4] BPM for PPG signals; accuracy in range of [95.4, 99.96]% for ECG). (4) Conclusions: Lightweight signal-processing techniques offer the most favorable trade-off between accuracy and efficiency for wearable implementations, whereas computationally intensive approaches are better suited for edge- or cloud-based processing.

## 1. Introduction

Cardiac activity analysis is one of the most widely used procedures to assess the condition of a subject [1,2], detect potential health risks [3], monitor sports performance [4], determine the level of stress [5], etc. Electrocardiogram (ECG) and photoplethysmograph (PPG) are two common noninvasive techniques to register cardiac activity. The former is a well-established technique based on measuring the electrical activity of the heart by means of electrodes placed mainly on the torso. In ECG signals, up to six waves can be distinguished, with the QRS complex, which is generated by ventricular contraction, being the most prominent. The second method, PPG, is based on the emission of light of a certain wavelength onto the skin. A receiver detects variations in the light passing through the tissue caused by changes in blood volume due to cardiac activity and its absorption by oxyhemoglobin. The recording of this signal is usually performed on the index finger, the wrist, or the earlobe [6].

The information provided by ECG is superior to the one given by PPG in terms of cardiac disease detection [7]. Nevertheless, heart rate (HR) and its variability can be obtained from both ECG and PPG [8]. HR is the most commonly measure used to analyze a subject’s state during the practice of sports [9], the cognitive load associated with a task [10] or the level of stress [11], and heart rate variability (HRV) has been successfully utilized for assessing the influence of the autonomic nervous system on cardiac activity, unveiling pathologies [12], monitoring the growth of a fetus [13] or detecting diabetes neurophaties [14].

HR can be calculated from the temporal distance between QRS complexes in ECG or as the principal frequency component in PPG signals, while HRV requires analyzing the series with the time intervals between consecutive heartbeats. Several factors must be taken into account when deciding between PPG and ECG: sensitivity to movement [15], the cost of the disposable electrodes, and the comfort of the measurement method. Maybe PPG is more comfortable, because it goes unnoticed in many smartwatch models that people are familiar with [16].

The literature contains a large number of review articles on techniques related to heart rhythm, ECG, and PPG signals. In [17], automated techniques for assisting in the diagnosis of pathologies are analyzed through the detection of the P wave, QRS complex, and T wave, with a focus on ECG-based research. Similarly, Ref. [18] reviews denoising techniques aimed at improving the quality of ECG signals and evaluates algorithms for detecting morphological features such as the QRS complex.

On the other hand, Ref. [19] focuses on the sensitivity of PPG signals to motion artifacts, reviewing various denoising and data reconstruction techniques and assessing their reliability. In [20], the lack of standardization in the acquisition and processing of PPG signals is analyzed, highlighting its negative impact on the generalizability and reproducibility of results. The work also reviews the main parameters influencing PPG signals that must be considered when evaluating sensors and proposes guidelines for future standardization efforts.

However, these review articles lack a critical aspect for practical implementation: an analysis of the computational implications of the proposed techniques.

Unlike existing reviews, this work focuses on analyzing different software algorithms for detecting heartbeat, as well as their computational and memory implications in portable systems with limited resources, and explicitly analyzes their suitability for applications oriented towards HR and HRV. This analysis is not usual in review papers, which is a differentiating element with respect to other similar works, as well as an added value, providing the reader with useful information when establishing a strategy for calculating HR and HRV. Likewise, the development of devices for acquiring ECG and/or PPG data is not the subject of this review, but it can help in the design process when establishing minimum requirements for memory, computing power, and energy consumption.

## 2. Materials and Methods

**Figure 1:**
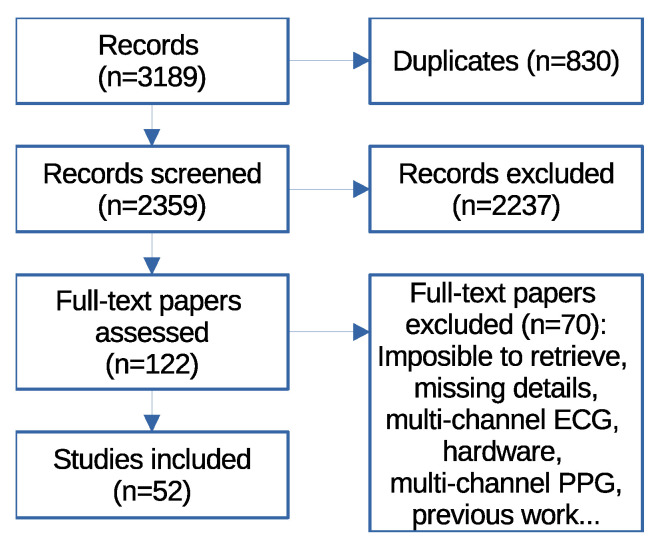
Scoping review. Keywords of first query: “ECG” AND “detect” AND “qrs”. Keywords of second query: “PPG” AND (“hr” OR “heartbeat rate”).

This study focuses on analyzing the computational complexity, memory requirements, energy consumption and accuracy of different algorithms aimed at detecting human heart rate using ECG or PPG signals in wearable devices. This review was conducted as a scoping review following the methodological framework proposed by Arksey and O’Malley [21], further refined by Levac et al. [22], and the Joanna Briggs Institute guidelines [23], according to a five-step process: identification of the research question, identification of relevant studies, study selection, data charting, and the collation, summarization, and reporting of the results. The reporting of this review was conducted in accordance with the PRISMA Extension for Scoping Reviews (PRISMA-ScR) guidelines, and it is available as Supplementary Material of this study. The aim of this study is to answer the primary research question: “How can heartbeats be detected in ECG and PPG signals?”. Additionally, the following secondary questions are addressed: “What computational limitations are involved?” and “What are the associated energy constraints?”. The PRISMA flow diagram is shown in Figure 1.

This study consists of a search process conducted across three major public scientific databases—Scopus (www.scopus.com), PubMed (https://pubmed.ncbi.nlm.nih.gov), and IEEE Xplore (ieeexplore.ieee.org)—to evaluate the techniques used in recent years for heart rate detection. These databases were selected due to their extensive coverage of scientific publications from multiple publishers. Specifically, IEEE Xplore was chosen because it is a leading database in engineering; Scopus was selected because it is a more general and comprehensive database; and PubMed was considered because it is a leading database in health sciences. The search was limited to publications from 2017 to 1 August 2024. The review protocol was registered in the Open Science Framework (https://osf.io/6ex2r, accessed on 13 April 2026), and the Supplementary Material lists all the papers analyzed in this study, along with the reasons for their exclusion.

### 2.1. Eligibility Criteria for the Scoping Review

To clearly define the scope of the review and address the study questions, eligibility criteria were established based on three elements: (1) data must be collected from humans and recorded using ECG and PPG physiological signals, so that they can be used to calculate HR; (2) the studies must describe algorithms that enable heartbeat detection and heart rate estimation; (3) the studies must be potentially applicable to wearable devices and embedded devices with limited resources, where real-time processing and energy efficiency are fundamental requirements. With these elements in mind, this study included only scientific papers focused on HR estimation using single-channel ECG or PPG signals. Multi-channel approaches were excluded to avoid introducing additional computational complexity, memory requirements, and energy consumption. Furthermore, the following exclusion criteria were applied: (1) papers not written in English or Spanish; (2) studies focused on hardware implementations, as this work aims to analyze algorithms rather than specific devices; (3) studies related to cardiac pathologies, animal data, denoising techniques, or fetal ECG, as they fall outside the scope of this work; (4) studies with non-reproducible results due to the use of non-public or unavailable datasets; (5) studies that did not report accuracy metrics; and (6) review articles.

The objective of this work is to identify, categorize, and compare methods for estimating HR, with a particular emphasis on their computational requirements and suitability for implementation in resource-constrained systems. In accordance with the exploratory nature of scoping reviews, this study is designed to provide a comprehensive mapping of the existing literature and to identify research trends, rather than to assess the methodological quality or risk of bias of individual studies.

Consistent with this objective and the adopted scoping review framework, no restrictions were applied based on publication type (journal, conference, or book chapter), in order to capture a broad range of relevant contributions. Likewise, no formal critical appraisal was conducted, as the focus of the review is placed on the characterization and comparative analysis of heartbeat detection methods from a computational and implementation perspective.

### 2.2. Information Sources, Search Terms and Screening for the Scoping Review

The search was conducted using consistent terminology across all databases. The first query employed the terms (“ECG” AND “detect” AND “QRS”), while the second query used (“PPG” AND (“HR” OR “heartbeat rate”)). In Scopus, the search was performed within the fields “Article title, Abstract, Keywords”. In IEEE Xplore, an advanced search was conducted using metadata fields, specifically (“All Metadata”:ecg) AND (“All Metadata”:detect) AND (“All Metadata”:qrs). In PubMed, the advanced search was performed using the query box.

For each database, two searches were conducted, limiting results to the period between 2017 and 1 August 2024. A total of 3189 articles were retrieved: 1155 from Scopus (550 ECG-based and 605 PPG-based), 1024 from IEEE Xplore (614 ECG-based and 410 PPG-based), and 1010 from PubMed (759 ECG-based and 251 PPG-based). After removing 830 duplicate records, a total of 2359 articles remained for screening. No formal quality assessment of the included studies was performed.

### 2.3. Data Extraction (Charting) Process and Items

One reviewer (M.M.M.) extracted descriptive data from the papers and organized them into a spreadsheet for the review process. Subsequently, three reviewers (M.M.M., C.L.V., and J.A.C.G.) screened the articles retrieved from Scopus and IEEE Xplore, while PubMed articles were reviewed by M.M.M., C.L.V., and G.S.A. All reviewers discussed the results and continuously updated the data charting form in an iterative process.

Due to the large number of studies, the screening process was conducted in three rounds. In the first round, filtering was based on titles; in the second round, abstracts were evaluated to exclude studies not aligned with the scope. In the third round, full-text analysis was performed. From the selected papers, accuracy metrics, datasets used, and descriptions of the techniques employed for heart rate detection were extracted. No pilot study was conducted during this process; instead, the data were used directly in our study.

The spreadsheet used in the study for the selection and data extraction process consisted of eight columns. The first five contained the information needed to identify and access the papers: title, authors, publication year, DOI, and abstract. The last three columns were used for the selection process. Each reviewer used a binary system to indicate whether the paper would proceed to the next phase or the reason for exclusion. Likewise, these last columns reflected the relevant information from the papers selected for inclusion in this study.

### 2.4. Synthesis of Results

The synthesis was based on the extraction of information directly from the selected studies and the subsequent analysis of the algorithms to determine their hardware requirements. No attempt was made to reduce the analysis time in order to preserve scientific rigor. The conclusions were derived from data extracted from the articles and organized into summary tables. The final synthesis was conducted by two reviewers (M.M.M. and A.J.M.C.).

## 3. Literature Review

A total of 2359 articles were analyzed. A screening process based on the title and abstract was conducted, after which 122 papers advanced to the full-text review phase. Of these, 52 were finally chosen to form part of this review after eliminating those that were not accessible (17 papers), described other topics (8 papers), did not use available databases (11 papers), lacked details in the explanation of the techniques (11 papers), used more than one channel of ECG or PPG (14 papers), or did not propose a new technique for detecting QRS complexes but rather employed techniques analyzed in other articles identified in this search (9 papers).

### 3.1. Heartbeat Rate Estimation from ECG Signals

Heartbeat estimation from the ECG signals typically involves multiple stages. First, the signal undergoes preprocessing to eliminate artifacts. Then, the QRS complex waves are detected. Finally, the HR is calculated using the formula $60/RR_{QRS}\left(i\right)$, where $RR_{QRS}$ denotes the time interval in seconds between two successive QRS complexes. The techniques discussed in this paper are summarized in Table 1 and Table 2.

#### 3.1.1. ECG Preprocessing

ECG signals often contain artifacts that can hinder accurate analysis. In clinical ECG acquisition, preprocessing typically aims to suppress baseline wander (<0.5 Hz) and power-line interference (50 or 60 Hz), commonly using a high-pass filter around 0.5 Hz and a notch filter near of 50 or 60 Hz [24]. Narrower bandpass filters, such as the 5–20 Hz range, are sometimes employed specifically to enhance QRS complexes for detection purposes [25,26,27,28,29,30,31,32,33,34,35,36,37,38,39,40,41,42]. However, this configuration does not represent standard ECG preprocessing and is primarily optimized for heartbeat detection rather than for full morphological or clinical analysis. Also, comparable results can be achieved using adaptive multiresolution techniques such as wavelet transform (WT), variational mode decomposition (VMD), and empirical mode decomposition (EMD), which decompose the signal into components across different frequency bands. These methods provide both time and frequency information, allowing for the selection of the frequency ranges associated with QRS activity [37,43,44,45,46,47,48,49,50,51].

In addition, several other preprocessing approaches have been explored: normalization with respect to the maximum amplitude [41,50,52], mean subtraction to remove DC offset [50,53,54], adaptive filtering based on the cuckoo search optimization algorithm (CSOA) [55], median filtering for low-frequency noise and power-line interference removal [31,48,56,57,58], moving average filter to ECG data [53,59] or the first derivative [54], and filtering via exponential weight mean–variance (EWMV) to smooth the signal and enforce positivity [58].

**Table 1:** Main features of the selected manuscripts obtained through the scoping review for ECG (part I). By default, the databases are gathered from Physionet (https://physionet.org/). Acronyms: MBA—MIT-BIH arrhythmia; NST—MIT-BIH noise stress test; NSR—MIT-BIH normal sinus rhythm; MSA—MIT-BIH supraventricular arrhythmia; INCART—St Petersburg INCART 12-lead arrhythmia; ESTT—European ST-T; QT—QT databse; FANT—MIT-BIH Fantasy; BPF—bandpass filter; MF—median filter; AT—adaptive threshold; HT—Hilbert transform; WT—wavelet transform; EMD—empirical mode decomposition; SE—Shannon energy; EV—envelope; NN—neural network; FD—first derivative; ^*B*^—the best case; ^*W*^—the worst case.

Ref.	Database	Main Techniques	Accuracy (%)	Comments
[43]	MBA	WT; HT	99.83	
[60]	QT	Entropy; WT	99.85	11 records
[25]	MBA	Pan–Tompkins process but different AT process	96.26	48 records of 10 s; positions of Q and S waves are calculated
[61]	MBA	Non-negative matrix factorization; AT	99.69	First channel of each ECG recording and excluded episodes of ventricular flutter from record 207
[44]	MBA; NSR	WT; AT	99.93 ^*B*^, 98.92 ^*W*^	
[26]	MBA	BPF; SE; digital first-order differentiator; HT	99.85	
[27]	12 databases	BPF; smoothing; EV	99.92	
[62]	MBA	VFCDM	99.89	
[45]	MBA; QT	EMD	99.86	Integrated P-wave detection
[28]	MBA	BPF; HT; AT	99.50	
[63]	MBA; QT; CPSC2019 **	NN	99.96	
[29]	MBA	BPF; variance; AT	99.69	
[30]	MBA	BPF; exponential transform; AT	99.71	
[46]	QT; NST; INCART; CPSC2019 **	WT; NN	99.71	ECG split into 10 s
[64]	MBA	EV; AT	99.64	Integrated detection of P and T waves
[65]	MBA; ESTT; INCART; MSA	NN	98.09	
[31]	MBA	BPF; MF	99.96	Integrated detection of P and T waves
[32]	MBA	BPF; SE; HT	99.69	
[33]	MBA; QT	BPF; normalized cubic power; AT	99.66 ^*W*^, 99.79 ^*B*^	Integrated detection of P and T waves
[66]	QT; Lobachevsky	NN	96.90 ^*B*^, 95.40 ^*W*^	Integrated detection of P and T waves
[55]	MBA	CSOA; AT	99.56	
[56]	Own data; QT; NST	EV; SE	99.92 ^*B*^, 88.25 ^*W*^	
[67]	QT	NN	96.2	Integrated detection of P and T waves
[34]	MBA	BPF; SE; HT	99.86	
[35]	MBA	BPF; S-transform using zero-order prolate spheroidal wave functions	99.92	
[47]	MBA; INCART; Long-Term ST; PTB Diagnostic	FD; WT	99.82	Integrated detection and (on/off-)set estimation of all ECG waves
[36]	MBA; QT	Hierarchical clustering	99.83	Integrated detection and (on/off-)set estimation of all ECG waves
[48]	MBA	WT; MF; AT	99.62	
[57]	MBA; FANT	MF; segmentation; AT; statistical false-peak elimination	99.72	
[37]	QT	EMD; BPF	99.90	
[38]	MBA; NST; ESTT; QT	Nonlinear filter; AT	99.98 ^*B*^, 97.78 ^*W*^	
[49]	MBA; QT	WT-BPF; AT	97.40	Integrated detection of Q and S waves

** Available at https://github.com/yshanyes/CPSC-2019-QRS-Detection-and-HR-Estimation, accessed on 13 April 2026.

**Table 2:** Main features of the selected manuscripts obtained through the scoping review for ECG (part II). By default, the databases are gathered from Physionet (https://physionet.org/). Acronyms: MBA—MIT-BIH arrhythmia; NST—MIT-BIH noise stress test; NSR—MIT-BIH normal sinus rhythm; MSA—MIT-BIH supraventricular arrhythmia; INCART—St Petersburg INCART 12-lead arrhythmia; ESTT—European ST-T; QT—QT database; FANT—MIT-BIH Fantasy; BPF—bandpass filter; MF—median filter; MAF—moving average filter; AT—adaptive threshold; NOR—normalization in [−1, +1]; LMS—least mean square; SG—Savitzky–Golay filter; HT—Hilbert transform; WT—wavelet transform; SE—Shannon energy; EV—envelope; NN—neural network; FD—first derivative.

[50]	MBA	BPF based on VMD; FD; SE; HT	99.84
[39]	MBA; NSR; MSA; ESTT; FANT	WT; EV	99.95
[40]	MBA	BPF; EV; AT	99.56
[58]	MBA; NSR; ST Change; Challenge 2014	MF; exponential weight variance; AT	99.66
[41]	MBA	BPF; Segmentation; fixed thresholds	99.63
[53]	MBA; ESTT; QT; INCART	MAF; Max.–Min. subtraction; AT	99.29
[51]	MBA	FD; MAF; NOR	99.84
[54]	MBA	LMS; SG; AT	99.52
[68]	MBA; INCART	NN	99.68
[42]	MBA; NST	Probabilistic analysis of the maxima/minima of the FD	79.25
[59]	MBA	MAF; peak detection; variance	99.04
[52]	MBA; CPSC2020 ***	NN	98.99

*** Available at https://github.com/DeepPSP/cpsc2020, accessed on 13 April 2026.

Some studies incorporate an additional enhancement step to improve QRS visibility by reducing high-frequency noise that can be mistaken for QRS peaks. Methods used include differentiators [25,26,28,32,34,42,44,51,68], Savitzky–Golay filters [55], envelope filters [56], nonlinear filters using monostable stochastic resonance [38], methods that scale the cubed signal [33] and normalization techniques in the range [−1, 1] [33,51,53].

#### 3.1.2. Heart Rate Estimation from ECG Signal

Once preprocessing is complete, QRS complexes are located—usually via a thresholding mechanism—to compute HR. Some methods include a preliminary candidate selection step to facilitate more precise localization, though other approaches bypass this and locate QRS peaks directly [27,58,62]. Techniques can broadly be categorized into time-domain and time–frequency-domain methods.

##### Time-Domain Enhancement

In time-domain approaches, the goal is usually to create a transformed signal in which each QRS appears as a distinct, bell-shaped pulse roughly matching the width of the actual complex. This is typically done by rectifying the signal, followed by a sliding window integrator [25,26,28,32,34,42,44,48,55,56] or a moving average filter [54]. Some methods include intermediate processing, such as computing Shannon energy [26,32,34,43,50,56], defined as $-(d{\left[n\right]}^{2}\log\left(d{\left[n\right]}^{2}\right))$, where d[n] is the first derivative of the rescaled signal. This step concentrates energy around QRS regions. Rectification may involve absolute values, squaring, or even exponential transforms of the first derivative [30]. The order of rectification and integration can also be reversed without major effect [57]. Alternatives to this process include envelope extraction [38,40,64], median filtering [31], or calculating signal variance [29]. Furthermore, in [54], a stage preceding rectification and integration is established, such that the prediction error of the output from an adaptive filter based on least mean square (LMS), to which a Savitzky–Golay filter is applied for smoothing, is used as data for QRS detection.

A similar result is obtained by applying a Shannon energy moving average filter to the normalized first derivative over the interval [−1, 1] [51], which is subsequently processed using a moving average filter of the square of the normalized first derivative [−1, 1] from the previous step, by calculating the variance of the 60 ms segment around the ECG peaks [59], or by applying a sliding window to calculate the difference between the maximum and minimum values [53]. In addition, in [53], the authors apply two parallel modulations using an ascending and a descending ramp function, both applied to the ECG signal and its derivative, to generate a set of peaks that are candidates for QRS.

A different strategy is used in [41], where to enhance the QRS complexes and better distinguish them from the other components, the output of an FIR filter is normalized to the range [−1, 1]. In this filter, all coefficients are set to −1, except for the central coefficient, which is equal to twice the number of filter coefficients. The goal here is not to create a bell-shaped curve that summarizes the QRS, but rather to preserve the morphology of the QRS complex so that it can be analyzed later to determine the locations of the Q, R, and S waves.

Another approach is taken in [59], where a probabilistic analysis is applied to determine the QRS complexes. In the study, various statistical parameters (peak slopes, peak amplitudes, and Bravais–Pearson correlation) are calculated from the processed data, which are then used to generate a posteriori probabilities using Bayer’s rules and Kullback–Leibler divergence.

##### Time–Frequency Transforms

Time–frequency approaches use transforms that provide simultaneous temporal and spectral information. For example, in [60], the first four levels of a WT decomposition are analyzed post-segmentation, selecting windows with maximum entropy. In [49], levels 1, 2, and 8 of an eight-level WT are discarded due to their frequency content, whereas [39] dynamically selects decomposition levels using $\left\lfloor \log_{2}\left(F_{s}\right)\right\rfloor$, where $F_{s}$ is the sampling rate, keeping only those within the [5, 20] Hz QRS band.

Other methods include using the module of Hilbert transform (HT) to generate envelopes aligned with QRS morphology [28], employing the variable frequency complex decomposition method (VFCDM) to retain levels 2–4 of a 12-level decomposition [62], or applying the S-transform with a prolate spheroidal wave kernel [35]. Also, in [61], non-negative matrix factorization is used on the spectrogram to separate QRS content from other components.

##### Thresholding

Following enhancement, thresholding is applied to detect the precise timing of QRS complexes. A common technique uses HT to identify zero crossings with positive slopes, indicating QRS peaks [26,32,34,43,50]. A similar approach is used in [60], where zero crossings in the first level of the WT are located within entropy-maximizing windows.

Other studies replace these transforms with thresholds against which to compare the data. Simple fixed-threshold methods [31,38,42] are limited in detecting low-amplitude QRS, prompting the use of adaptive thresholding.In [41], three fixed thresholds (0.22, −0.2, and 0.52) are established, one for positive peaks and the other for negative peaks. QRS complex candidates are determined based on the evaluation of four cases at points where the thresholds are exceeded (one peak and one trough; two peaks and one trough between them; a single peak exceeding 0.52; other combinations). Subsequently, the maximum QRS complex point is obtained by shape analysis of the waveforms of the QRS candidates. Other techniques propose to use thresholds that adapt to the amplitude of the data. For example, Ref. [56] uses a 3 Hz low-pass filter followed by peak detection, while others define thresholds as fractions of the max QRS value [25,35,37,64]. Some techniques average upper and lower envelopes [27,39] or calculate the average of the first 70 ms of the squared derivative ordered in descending magnitude [47], where false-positive rejection is achieved by checking derivative zero crossings within a 112 ms window centered around the QRS candidate or the threshold is set as the value of the maximum peak of the derivative modulated by a descending ramp [53].

Adaptive thresholding is often implemented with recursive updates based on past and current QRS amplitudes. Strategies include weighted moving averages [48,49,54,55,59], exponential decay [58], or other custom schemes [30,40]. An additional step is described in [59], where statistical parameters are used to determine the QRS, and in [40], where the authors use QRS template matching for final peak location. In [59], two additional thresholds are calculated based on the amplitude and kurtosis of the candidate waves, such that QRS complexes are identified for those values that exceed both thresholds. In [40], the intervals between heartbeats are estimated by exploiting the repetitive morphological structure of cardiac cycles rather than explicitly detecting peaks. This approach generates a template from incoming signal segments and uses it to locate heartbeats in the input data at points of maximum similarity. In this method, a 120 ms template is generated from the first five QRS detected, and similarity is calculated using cross-correlation between the data segment where a heartbeat has been determined and this template. The R peak of the QRS complex will be located at the point where the maximum correlation occurs.

Several studies propose dual-threshold systems [33,57]. These are useful for detecting both upright and inverted QRS complexes. Thresholds may be tuned iteratively until convergence [28,29,69] or computed automatically from histograms [45,61].

Temporal filtering is also applied to discard spurious detections closer than 250 ms apart [30,36,37,39,45,50,57,62,63]. The time threshold can also be adjustable, as in [51], where two time thresholds are established: one to rule out false positives and another to detect false negatives and trigger a review process based on that threshold.

#### 3.1.3. Machine Learning in ECG

Machine learning approaches have gained traction for QRS detection, with many models trained to predict the likelihood of a QRS within a given segment.

Ref. [67] compares three architectures: a two-layer fully connected network and two convolutional neural networks (CNNs), one of which includes dropout. The best performance is achieved by the dropout-free CNN, featuring two convolution–maxpool layers, followed by vectorization and two dense layers.

Ref. [63] introduces two CNNs differing in the use of stacked long short-term memory (LSTM) networks. The base model includes three parallel blocks, each with three convolution–maxpool subblocks, followed by a channel attention mechanism and a dense output layer with sigmoid activation.

In [46], an 11-branch CNN processes low- and high-frequency components extracted via WT. Octave convolutions [70] are applied at the input and near the output to merge frequency content. The outputs are then aligned and concatenated before feeding into two fully connected layers.

Ref. [68] describes a structure consisting of two parallel CNNs, each with two layers, in which 1D convolutions are applied to each layer. The outputs of these layers serve as the inputs to a two-layer multi-layer perceptron (MLP), whose output determines the QRS complexes.

Ref. [65] proposes a hierarchical system of three neural networks. The first has two hidden layers and 54 inputs; the others have one hidden layer each, taking 20 and 4 inputs. The final model outputs the QRS location.

In [52], an encoder–decoder architecture is designed using 1D convolutional blocks on 20-s signal segments with six layers. In the encoder, a factor-2 max pooling is applied between layers, and in the decoder, factor-2 upsampling is applied, followed by a convolution. The kernel sizes are 9, 6, and 3 for two consecutive layers, and the number of filters starts at 16 and doubles every two layers, with the last layer having 64 filters. Each convolution is followed by batch normalization and a ReLU activation function. The output layer replaces the ReLU function with SoftMax.

Another encoder–decoder architecture is described in [66]. The encoder includes four convolutional layers (each with standard and dilated convolutions) followed by a bidirectional LSTM. The decoder upsamples and convolves outputs at multiple depths, which are then passed through a dense softmax layer for classification.

Finally, Ref. [36] applies an unsupervised hierarchical clustering algorithm using Euclidean distance. A sliding 1.2-s window is used to extract amplitude and slope features, and clusters labeled as QRS are refined to choose the sample with maximum absolute amplitude from each group of adjacent detections.

### 3.2. Heartbeat Rate Estimation from PPG Signals

Heartbeat detection from PPG signals typically begins with a preprocessing phase aimed at removing artifacts, followed by candidate selection and heartbeat localization. Some authors, however, bypass the candidate selection step by directly applying machine learning techniques to the preprocessed data. Table 3 provides an overview of the methods reviewed in this paper.

#### 3.2.1. PPG Preprocessing

PPG signals are often corrupted by various noise sources, most notably motion artifacts (MAs). Given that HR generally falls within the range of [40, 240] BPM, preprocessing commonly starts with bandpass filtering to isolate this frequency range. Typical filter bandwidths include [0.4, 4] Hz [75,77], or, more broadly, [0.5, 10] Hz [76].

Several strategies exist to mitigate the effect of MAs. In [72], EMD is used to select intrinsic mode functions (IMFs) with dominant frequencies in the range of [0.75, 2.5] Hz, discarding the rest. Similarly, [73] employs VMD to retain components with dominant frequencies in [0.6, 5] Hz.

Another technique, described in [71], uses a variance characterization series (VCS) to detect MAs. If motion is detected, the signal is decomposed using EMD, and a matrix of IMFs is created. Singular value decomposition (SVD) is then applied, and the VCS is re-evaluated to decide whether to apply WT-based filtering or reconstruct the signal using only the first half of the columns in the *U* matrix from the SVD.

In [77], z-score normalization is applied to the bandpass-filtered data. The discrete cosine transform (DCT) is then computed, followed by filtering with a bank of nine non-overlapping, zero-phase filters. Inverse DCT is used to reconstruct a noise-free PPG signal from the unaffected frequency components.

Another effective approach is to use an adaptive LMS filter, as in [75], where the inputs, normalized by a z-score, are a three-axis accelerometer and the contaminated PPG signal.

#### 3.2.2. Heart Rate Estimation from PPG Signal

HR can be estimated using the equation $HR=60\cdot F_{HR}$, where $F_{HR}$ is the main frequency component of the PPG signal. This frequency is often computed as the average peak frequency within the Fourier transform (FT) in the [0.4, 5] Hz range [71] or simply as the most dominant spectral component [77].

To improve reliability, some studies use principal component analysis (PCA). In one approach, PCA is applied directly to the preprocessed PPG signal [73]. In another, PCA is applied only to certain IMFs obtained through EMD [72], specifically, those with dominant frequencies in the range of [0.75, 2.5] Hz. In both cases, the $F_{HR}$ is estimated using the first principal component.

Motion-induced interference often complicates HR estimation. While accelerometer data are commonly used during preprocessing, some methods incorporate them directly into the estimation phase. For example, Ref. [74] reconstructs the HR curve from spectrograms of both the PPG and accelerometer signals. After enhancing spectral resolution using the FOCUSS algorithm [79], a combination of Gaussian and Hessian filters is used to derive the HR curve. Discontinuities caused by MAs are detected by comparing the average eigenvalues of the Hessian matrix of the PPG and accelerometer channels at suspected points. If the accelerometer value exceeds that of the PPG, the segment is deemed contaminated and corrected via interpolation between the current and next valid segments.

A different approach is found in [75], where a temporal analysis involving three stages is employed: peak detection, preliminary HR estimation, and final estimation. Initially, all positive peaks spaced at least 300 ms apart are identified. Peaks separated by less than 50% of the average interval of the last 10 valid peaks are discarded. To prevent false negatives, if two consecutive peaks are separated by more than 180% of the average interval, an intermediate peak is added. An initial HR estimate is obtained by averaging the intervals of peaks whose distances fall between 70% and 140% of the mean of the last 30. This value defines the center frequency for a 0.5 Hz wide bandpass filter, which is applied to the preprocessed PPG. The resulting signal is subtracted from the original, and the final HR is computed as 60 divided by the average inter-peak interval.

Some studies incorporate error correction mechanisms. In [77], any sudden rise in HR exceeding 5 BPM compared with the previous estimate is considered an outlier and is replaced with the average of the two preceding values.

#### 3.2.3. Machine Learning in PPG

Machine learning (ML) techniques, particularly CNNs, are increasingly employed for HR estimation from normalized PPG time series [76,78]. To reduce computational load, the data may first be decimated.

Network architecture varies across studies. In [76], a seven-layer model is used: the first three layers apply convolution and dilation, followed by a data function layer, a normalization layer, and two dense layers. In [78], the structure also comprises seven layers: the first and third ones are convolutional and normalization layers, the second and fourth ones are max-pooling layers, the fifth and sixth ones incorporate LSTM units, and the final layer is dense, outputting the estimated HR.

### 3.3. Heart Rate Variability

Although HR estimation and HRV analysis are often discussed together, they impose fundamentally different requirements on signal-processing algorithms [24]. HR estimation aims to obtain an average cardiac rate over a given observation interval and can tolerate moderate temporal imprecision, as small errors in individual beat locations tend to average out. Consequently, HR can be accurately estimated using approaches based on spectral analysis, dominant-frequency extraction, or temporally averaged measures.

In contrast, HRV analysis is defined from the sequence of beat-to-beat (NN) intervals and therefore requires precise temporal localization of individual heartbeats. Errors of only a few milliseconds in beat detection can significantly affect HRV metrics such as root mean square of successive differences (RMSSD) or standard deviation of normal-to-normal intervals (SDNN). As a result, only methods that explicitly detect individual beats and preserve the inter-beat interval series are suitable for HRV-oriented applications.

From a practical perspective, methods based on frequency-domain estimation or temporal averaging are generally sufficient for HR monitoring but are inherently unsuitable for HRV analysis, regardless of the observation window length. Conversely, time-domain and event-based detection methods enable both HR and HRV estimation but impose stricter requirements on signal quality and detection accuracy. These distinctions are particularly relevant in wearable systems, where computational efficiency must be balanced against the need for precise beat-to-beat information. For this reason, the methods used to obtain the principal frequency, mainly used in PPG and which generate an average value of HR, are not applicable to HRV, as they lack the time vector indicated above. Thus, only those techniques that temporarily locate the QRS complexes are valid for performing an HRV analysis. Therefore, all the techniques analyzed for estimating HR in ECG signals, including ML algorithms, can be used for HRV (Section 3.1), while for PPG (Section 3.2), only the method described in [75] seems suitable, since it works in the time domain by detecting peaks in the PPG signal.

### 3.4. Computational Cost and Memory Requirements

The implementation of any of the previously described techniques on wearable devices must consider both computational cost, which directly impacts energy consumption, and memory usage, which is highly constrained in these platforms. These aspects are summarized in Table 4.

#### 3.4.1. Computational Cost

The lowest energy consumption is typically achieved with algorithms exhibiting linear computational complexity, denoted by O(*N*), where *N* is the length of the entire input data or a segment thereof. Many of the discussed techniques fall into this category. For instance, algorithms that apply a linear combination of coefficients (either constant or adaptive) to the input vector—such as Savitzky–Golay filters, moving average filters, bandpass FIR/IIR filters, LMS, CSOA, EWMV, stochastic resonance, or exponential transforms—are all linear in complexity. Similarly, operations like thresholding, find peaks, difference between the maximum and minimum values, zero crossing detection, interpolation (for example, envelope filters), integration, rectification, and normalization also exhibit linear time complexity. Median filters, while traditionally presenting O(NlogN) due to sorting, can be optimized to linear complexity if the buffer is pre-sorted and samples are processed incrementally by updating only the necessary position.

The next set of algorithms in terms of computational cost includes VCS and time–frequency decomposition methods. VCS requires O(LN) operations, where *L* is the window size. Time–frequency decomposition techniques such as WT and EMD scale with O(kN) and O(kNM), respectively, where *k* is the number of decomposition levels and *M* the number of iterations. Other methods, such as FT, HT, S-transform, and DCT, have a complexity of O(NlogN), while other approaches like spectrogram, VFCDM, and VMD exhibit O(kNlogN), O(kNlogN), and O(kMNlogN), respectively.

Several of the aforementioned techniques involve matrix construction, either due to multi-channel input or because a single signal is segmented into multiple parts. For example, the Hessian and Gaussian filters—used for edge detection and smoothing in two-dimensional contexts—have computational complexities of O(NS) and O(NSL), respectively, where *N* is the number of rows (samples), *S* the number of columns (channels), and *L* the number of filter elements.

Eigenvalue-based methods like PCA have a complexity of O($N^{3}$) for square matrices, and approximately O($NS^{2}$) when applied to rectangular matrices, approaching the cost of SVD. Techniques such as non-negative matrix factorization and the FOCUSS algorithm, used in sparse signal reconstruction, are iterative processes that involve matrix multiplications. Their complexities are O(MNSP) and O($MN^{2}S$), respectively, with *M* being the number of iterations, and *S* and *P* being part of the dimensions of the matrices used in the process.

The computational cost of neural networks depends on their architecture. CNNs, for example, involve standard and/or dilated convolutional layers where filters of length *K* are applied across the input of length *I* (K<I) with stride *Z*, and expansion defined by the number of filters *F*. The overall cost is O(⌈(I−K)/Z⌉F). Other common layers in CNNs are pooling layers (average, max, etc.) that have O(⌈(I−K)/Z⌉) complexity, and normalization and activation layers (sigmoid, ReLU, etc.) both operate in linear time O(*I*). Some CNN variants incorporate attention mechanisms such as squeeze-and-excitation networks (SENets), which incurs a cost of O($IC+C^{3}$), with *C* representing the number of channels. Dense (fully connected) layers, often used in final stages, require O(IP), where *P* is the number of neurons in the layer.

More complex architectures often integrate other types of neural networks. LSTM layers, for instance, have a computational complexity of O($I^{2}U$), where *U* is the output vector size [89]. MLPs and autoencoder variants follow a dense-layer-like structure, with complexity O($IP_{1}+\sum_{i=2}^{H}P_{i-1}P_{i}+P_{H}U$), where $P_{i}$ is the number of neurons in hidden layer *i* and *H* is the total number of hidden layers.

Based on the above, the systems in [63,76], which utilize LSTM layers, exhibit a computational complexity of O($N^{3}/R^{3}$), with *R* representing a data reduction factor. The systems described in [46,67,78], which incorporate dense layers, operate at O($N^{2}/R^{2}$). The system in [65] employs an MLP architecture, resulting in O(NP) complexity, while Ref. [52] describes an encoder–decoder setup involving several convolutions and pooling, and in [66], bidirectional LSTM units and a dense output layer are also added—resulting in O($N^{3}/R^{3}+N^{2}$)

#### 3.4.2. Memory Requirements

Excluding the memory overhead associated with basic mathematical operations (logarithm, exponentials, square roots, etc.), most of the algorithms discussed in this work require memory proportional to the size of the data, that is, O(*N*) or O(NlogN). Time–frequency decomposition techniques introduce additional dependency on the number of decomposition levels *k*, leading to O(kN) for WT and EMD and O(kNlogN) for VMD.

Matrix-based techniques require memory proportional to the size of the matrices involved, typically O(NS), except for the FOCUSS algorithm, which requires O($N^{2}$) due to quadratic memory usage. For ML systems, the dominant memory cost arises from dense layers, as each input node connects to each output node. Thus, their memory footprint is of order O($N^{2}/R^{2}$).

#### 3.4.3. Evaluation of the Analyzed Algorithms

The proposed algorithms were evaluated considering the hardware constraints of typical wearable platforms such as the ESP32 and nRF5284. These devices typically operate at 64–240 MHz with RAM capacities between 256 and 520 kB. The computational complexity of the main implemented algorithm parts were analyzed in terms of arithmetic operations and required memory buffers. Unfortunately, it is not feasible to define exactly these figures for all the analyzed papers. However, we can approach a realistic scenario where we can establish a comparison among them. For instance, regarding the described algorithms using WT decomposition [48], most memory requirements must be allocated to the different decomposition levels—typically four or five. This allocation remains manageable even if the algorithm subsequently employs a median filter, for which the required memory is comparatively negligible. In this work, we assume that data will be stored in Q15 format. This format utilizes 16 bits for the fixed-point representation of numbers within the range of [−1, 1], providing sufficient precision for many physiological signals while enabling processor optimization and a reduction in RAM usage.

Table 5 represents the computational requirements of the analyzed studies for both ECG and PPG. In this regard, the ESP32 can support all the proposed solutions, as it possesses sufficient capacity to host the matrices and variables for every algorithm. The highest memory demands stem from those based on WT [39], EMD [37,45,72], VMD [73], or PCA [72,73] when processing large data samples, as well as those utilizing CNNs [63]. For the ML models, we assume that the network coefficients (weights) are stored in the ROM, which has a larger capacity, thereby freeing up RAM resources. The algorithm requiring the most RAM is [63], as it implements a 20-layer convolutional network necessitating 240 KB. Other AI algorithms, such as the one described in [65], are more memory-efficient, particularly when reducing the CNN complexity to a single layer [67]. The algorithms with the lowest demands are those based on linear filters [26,31,53,90] and some that employ more operations but with smaller window data lengths, such as that in [48], which applies the wavelet transform to 54 samples.

For wearable devices, the critical point is battery life, which depends on factors such as algorithmic complexity, sampling frequency, the processing window stride, and the specific computing capacity of the ESP32. Regarding this last parameter, according to the specifications indicated in [91], the per-core performance of the ESP32’s 32-bit RISC architecture ranges from 1.25 to 1.61 DMIPS/MHz. Assuming a clock signal of 160 MHz (the mid-range in the 80–240 MHz at which it can operate), we have a capacity of 192 MIPS–256 MIPS. Reducing the wearable’s power consumption implies that the processor must minimize its active time $T_{act}$ (%), which depends on the algorithm complexity (*Nop*), the step between consecutive processing windows (*D*) given in number of samples, and the sampling rate (*Fs*):

(1) $$T_{act}\left(\%\right)=100\frac{F_{s}\times Nop}{MIPS\times D}$$

We assume two possible scenarios. In both, *MIPS* = 192, and the *Fs* is that indicated by each respective author. Regarding the latter, since most studies utilize standard databases, we found that for ECG, the most common *Fs* is 300 Hz, while for PPG, it ranges between 25 Hz and 125 Hz. In cases where an algorithm was tested with multiple frequencies, we selected the worst-case scenario (that is, the highest sampling frequency).

Some algorithms allow for processing windows with D>1, while others do not. For instance, in the implementation of FIR filters, the filter output must be updated every time a new sample is received. However, other methods, such as those based on the fast Fourier transform, can buffer data windows before the processor enters active mode. The ESP32, along with many other modern processors, allows for data acquisition and storage in sleep mode using a ULP (Ultra-Low-Power) coprocessor. The system only transitions to active mode once the *D* samples required for the circular buffer of length N have been received.

Certain articles specify the steps between processing windows, while others do not. For the latter, we assumed the processor wakes every *D* = N/2 samples. We also analyzed the extreme case where all ECG algorithms are executed with *D* = 1 to assess their viability. Most PPG algorithms utilize an 8 s window size with a 6 s overlap; for these, the extreme case will not be analyzed.

According to the manufacturer, current consumption in active mode averages 170 mA, while in low-power mode, it is in the range of 0.1 mA. The average current ($I_{avg}$) is given by the following expression:

(2) $$I_{avg}=170\;\mathrm{mA}\frac{T_{act}}{100}+0.1\;\mathrm{mA}\left(1-\frac{T_{act}}{100}\right)$$

For the majority of the studies analyzed, the calculated power consumption would be lower than 0.2 mA, which is significantly less than the average consumption observed in scenarios involving active Wi-Fi or Bluetooth Low Energy communications.

The results demonstrate that all the evaluated algorithms are feasible for implementation. Those with the lowest energy consumption are: the Pan–Tompkins algorithm [25], those based on envelope calculation [39,56,64], filtering [33], the CSOA [55], and even one based on WT [60].

If processing windows are reduced to a step of *D* = 1 sample for ECG, it can be demonstrated that three of the algorithms become non-implementable due to the required computational load [50,61,63]. As shown in the table below, the active period for these cases exceeds 100% of the available CPU time.

## 4. General Discussion

Interest in accurately determining the HR has remained strong over the years, as it is a critical indicator of an individual’s physiological state. Beyond medical applications—particularly in assessing cardiovascular risk—HR is widely used to evaluate physical fitness during exercise and to infer psychological variables such as stress, cognitive load, or emotional state. While ECG provides richer information for diagnosing cardiac conditions, both ECG and PPG are equally effective in estimating HR. As shown in Table 1 and Table 2, the beat detection accuracy of ECG-based methods generally exceeds 99%, a value comparable to that obtained using PPG techniques [8] (Table 3).

Both technologies commonly utilize methods that combine time- and frequency-domain information, particularly in preprocessing stages. These methods have demonstrated high reliability in HR estimation, with a mean absolute error (MAE) below 3 BPM for both ECG and PPG. As a result, factors such as economic cost and user comfort become more decisive when selecting a technology, often making PPG the preferred choice.

With regard to HRV, it is necessary to detect the temporal location of heartbeats for subsequent analysis. For this reason, techniques that generate an average HR value without taking into account the location of QRS complexes are not valid. All the techniques analyzed for ECG temporarily locate heartbeats, which allows a time series necessary for this analysis to be generated. However, in PPG, most of the algorithms analyzed seek to obtain the average value of HR without taking into account the location of heartbeats, so they are not suitable. Nevertheless, it is possible to approximate their location from the PPG data. To do this, it is sufficient to determine the maximum peaks in the PPG, which correspond to the systole, so that the time difference between two consecutive maxima is assimilated to the time distance between two consecutive QRS complexes [92]. This is known as pulse rate variability (PRV), and since pulse transmission time depends on blood pressure, vascular tone, temperature, posture, and/or sympathetic activity, the result is similar to HRV but not exactly the same. However, they are highly correlated, greater that 0.97 [92] for subjects at rest, making it a good substitute for HRV when the latter is not available. Nevertheless, it appears that certain stressful situations and physical activity (motion artifacts) often affect the concordance between the two to an unacceptable degree [93], overestimating short-term variability due to the coupling between respiration and the vascular system.

ECG systems typically involve higher technical complexity and cost, requiring a bioamplifier operating at a minimum sampling rate of 170 Hz [94], along with at least two electrodes. In contrast, PPG can be acquired using an inexpensive light emitter and photoreceptor at a sampling rate as low as 50 Hz [95]. Lower sampling rates reduce both data volume and computational load. Nevertheless, despite the strong correlation between HR estimates from both methods [96], ECG offers more accurate and immediate detection, especially in individuals with underlying conditions.

Conventional ECG setups require two electrodes placed far apart on the body (for example, armpits or wrists), connected to a bioamplifier—often embedded in garments. However, such configurations raise the overall system cost. A simpler alternative is to place both electrodes on the same wrist, with one in constant contact and the other activated by touch from the opposite hand, thereby completing Einthoven’s Lead I. Though more cost-effective, this setup is ergonomically limited and unsuitable for continuous monitoring, as it requires active user participation.

Both ECG and PPG signals require preprocessing to suppress various noise sources, such as powerline interference, half-cell potential fluctuations at the skin–electrode interface, or ambient light variations. Movement artifacts, particularly problematic for PPG, cause significant signal distortion due to vibration-induced fluctuations. Many studies have therefore focused on reducing motion artifacts using three-axis accelerometer data in the preprocessing stage. While effective, this approach increases memory use and computational demands.

Energy efficiency is a key factor when implementing HR estimation algorithms on battery-powered devices. FIR/IIR, median, and adaptive filters are commonly used in preprocessing because of their linear computational complexity and relatively low energy consumption—favorable attributes for wearable devices such as smartwatches. By contrast, techniques providing joint time–frequency information typically involve higher computational costs. However, this can be justified if the resulting features are immediately usable for HR estimation (for example, through peak detection or dominant-frequency analysis), thus minimizing downstream processing.

Assuming that cost is not a constraint and continuous HR monitoring is feasible using either ECG or PPG, the choice of technique should strike a balance among energy consumption, memory requirements, and estimation accuracy. Matrix-based and ML algorithms tend to demand more resources while delivering similar or inferior accuracy compared with simpler methods. Linear-cost techniques, such as those presented in [27,31,35,71], offer a superior trade-off, achieving accuracy above 99.92% with minimal memory usage. These techniques are especially well suited for implementation on mobile platforms with limited energy and memory budgets.

Although the use of ML methods has surged recently, many such studies were excluded from our review due to misalignment with our selection criteria. Among those retained, ECG-based ML approaches achieved accuracy rates above 96.2%, while PPG-based methods reported MAE values exceeding 3.6 BPM. These figures indicate lower performance relative to conventional techniques. Moreover, the high memory demands of ML models make them impractical for direct implementation on wearables. Based on our findings, ML methods are currently not recommended for on-device HR estimation.

However, the constraints of onboard memory and battery life can be circumvented by offloading processing to an edge device, like a smartphone. In this model, the mobile device receives and processes ECG or PPG data, estimating HR externally. This approach introduces a new consideration: the energy cost of continuous data transmission, which varies depending on the communication protocol employed and warrants further investigation.

## 5. Discussion on the Evaluation of the Analyzed Algorithms

The reviewed literature indicates that the performance of heartbeat detection methods cannot be assessed solely in terms of estimation accuracy. Although all papers included are theoretically implementable on devices such as the ESP32, computational complexity, memory footprint, and energy consumption play a decisive role and often outweigh marginal accuracy improvements. To enable a systematic comparison, the analyzed methods are evaluated according to four key criteria: (1) estimation accuracy, (2) computational complexity and associated energy consumption, (3) memory requirements, and (4) suitability for real-time implementation in wearable devices.

From a comparative perspective, classical signal-processing methods and ML-based approaches exhibit fundamentally different trade-offs (Table 6). Most of the reviewed methods achieve accuracy values above 95%, with only a few exceptions, such as [42], which reports accuracy below 80%, and [78], where the MAE exceeds 10 BPM. These results indicate that a wide range of methods can provide reliable heart rate estimation, and algorithms with similar accuracy levels may exhibit differences of several orders of magnitude in terms of computational cost and energy consumption. Therefore, accuracy alone is not a sufficient criterion for selecting algorithms in wearable applications.

A key observation is that memory limitations are generally not the primary restricting factor. Even the most demanding techniques, such as CNN-based or matrix-based methods, can be implemented on modern wearable platforms due to their sufficient memory capacity, particularly when model parameters are stored in ROM. In contrast, the main limitation arises from computational load and its direct impact on energy consumption.

Classical time-domain and frequency-domain techniques consistently achieve high accuracy while maintaining linear computational complexity (O(N)), low energy consumption, and minimal memory requirements. This is due to their reduced number of operations and their ability to operate with minimal buffering requirements. These characteristics make them particularly well suited for real-time implementation in resource-constrained wearable devices, where processing time and battery lifetime are critical factors.

In contrast, matrix-based and ML approaches are designed to capture complex signal patterns but typically involve significantly higher computational complexity, ranging from $O(N^{2})$ to $O(N^{3})$, as well as increased memory demands due to model parameters and intermediate representations. Although modern embedded platforms (e.g., ESP32) and frameworks such as TensorFlow Lite (https://www.tensorflow.org/lite/, accessed on 13 April 2026). facilitate the deployment of such models by enabling parameter optimization and model compression, their energy consumption remains substantially higher due to these introducing a substantial increase in the number of operations, which directly translates into longer processor active times and, consequently, higher energy consumption, as shown in Table 5. Furthermore, the reviewed studies indicate that their accuracy is often comparable to that of simpler signal-processing methods, particularly for heart rate estimation tasks.

As a result, the performance improvements offered by ML techniques do not always justify their increased resource consumption in wearable environments. These approaches may be more appropriate in scenarios where processing can be offloaded to external devices or cloud-based systems.

A critical factor influencing energy efficiency is the processing window strategy, particularly the step size. Algorithms that allow for buffered processing can significantly reduce processor activation frequency by leveraging low-power acquisition modes. In contrast, sample-by-sample processing, as required by some filtering-based approaches, forces continuous processor activity, which may drastically increase energy consumption. This effect becomes especially relevant in high-complexity algorithms, where the processor may not be able to complete all required operations within the available time budget, as observed in [50,61,63].

Finally, the results emphasize the importance of considering system-level parameters, such as sampling frequency, processor performance, and duty cycle, when evaluating the suitability of algorithms. The proposed formulation for $T_{act}$ and $I_{avg}$ provides a useful framework for translating algorithmic complexity into realistic energy consumption estimates, enabling a more meaningful comparison across studies.

From a system-design perspective, classical signal-processing methods offer a favorable balance between performance and resource consumption. For ECG signals, QRS-based peak detection methods provide accurate and reliable beat localization while maintaining linear computational complexity and minimal memory requirements, making them well suited for long-term on-device monitoring and HRV analysis. Similarly, for PPG signals, simple peak-based or frequency-based approaches are generally sufficient for average heart rate estimation, particularly under controlled or low-motion conditions.

An additional limitation observed across several studies is the mismatch between algorithmic complexity and the actual requirements of the physiological metrics of interest. While average HR estimation can tolerate moderate temporal imprecision and may benefit from smoothing or spectral aggregation, HRV analysis imposes stricter requirements on beat-to-beat accuracy. Methods that do not explicitly preserve inter-beat interval information, regardless of their overall HR accuracy, are therefore unsuitable for HRV-oriented applications.

Thus, the main design trade-offs identified across the reviewed methods can be summarized as follows:

1. Accuracy vs. computational cost: Increasing algorithmic complexity does not necessarily result in higher accuracy, particularly in ECG-based detection, where simple peak detection methods already achieve accuracy above 99%.
2. Memory vs. real-time capability: Methods requiring large memory buffers or extensive model parameters (for example, CNN-based or matrix-based techniques) may hinder real-time processing on embedded platforms.
3. Generalization vs. implementation constraints: ML models may generalize better across datasets but are often less suitable for on-device deployment due to hardware limitations.

Overall, the findings suggest that the design of heartbeat detection algorithms for wearable devices should prioritize computational efficiency and energy-aware processing strategies over marginal improvements in accuracy. Future work should therefore focus on optimizing algorithmic structures for low-power operation and on reporting standardized metrics that reflect real-world implementation constraints.

## 6. Limitations of This Study

Several limitations of the scoping review process should be acknowledged. First, the review was restricted to studies published between 2017 and 2024, which may have excluded earlier foundational work that continues to influence current heartbeat rate estimation techniques.

Second, although predefined inclusion and exclusion criteria were applied, the selection process may still be subject to publication bias and database coverage limitations, potentially leading to the omission of relevant studies not indexed in the selected sources.

Third, as a scoping review, the objective was to map and categorize the available evidence rather than conducting a formal quality assessment or quantitative meta-analysis; therefore, the methodological rigor of individual studies was not systematically evaluated, and performance metrics were not statistically pooled.

Fourth, multi-channel techniques were excluded from the analysis. This decision was motivated by the objective of focusing on computationally efficient solutions suitable for resource-constrained devices. However, this exclusion may limit the generalizability of the results, as multi-channel approaches can improve robustness and detection accuracy.

Fifth, advanced denoising techniques were not explicitly considered. This is particularly relevant for PPG signals, where motion artifacts can significantly degrade signal quality and affect heart rate estimation performance.

Additionally, the inclusion of ML-based approaches is limited. This is mainly because many ML-based studies do not align with HR assessment. Consequently, the review may not fully capture the breadth of ML methodologies proposed for heartbeat detection.

Finally, alternative HR measurement approaches, such as camera-based methods (for example, remote photoplethysmography), were not included. The omission of these non-contact techniques narrows the scope of the study to ECG and contact-based PPG signals.

In addition, heterogeneity across the included studies—regarding datasets, signal acquisition conditions, hardware platforms, evaluation metrics, and subject populations—limits the direct comparability of results. Reported accuracy, mean absolute error, and computational requirements were often obtained under different experimental setups, which may affect generalizability to real-world wearable environments. Finally, rapid technological evolution in wearable hardware and machine learning techniques means that some conclusions, particularly regarding resource constraints, may evolve as more efficient implementations become available.

## 7. Conclusions

HR is a vital biomarker for assessing a subject’s physiological state, making accurate and efficient estimation techniques essential, especially for continuous monitoring applications. Ideally, such methods should be implementable on mobile devices like smartwatches. The techniques reviewed in this study demonstrate high accuracy; thus, the primary limitations lie in power consumption and, to a lesser extent, memory usage.

Algorithms involving matrix operations or ML models require more memory and consume more energy. Thus, although they can be implemented on wearable devices, it is recommended that data processing be performed externally to ensure efficient operation. In contrast, the remaining techniques assessed—particularly those with linear computational complexity—can be effectively executed on resource-constrained devices, offering a viable path for real-time, on-device HR estimation. Accordingly, these lightweight and efficient methods are strongly recommended for implementation in mobile health monitoring systems.

In the future, based on this information, we propose using open-source hardware platforms, such as Watchy, to overcome the typical limitations of commercial APIs, which will allow us to implement real-time algorithms for heart rate estimation while taking advantage of its ESP32 processor and ultra-low-power e-ink display; we will incorporate noise-cancellation techniques to eliminate motion artifacts and analyze optimal window lengths to optimize energy consumption.

## Figures and Tables

**Table 3:** Main features of the selected manuscripts obtained through the scoping review for PPG. Acronyms: MAE—mean absolute error; MIIP—Mimic II database from Physionet; MIP—Mimic database from Phsyionet; CBD—Capnobase database; UQVSD—University of Queens Vital Sign database; SPC—IEEE Signal Processing Cup 2015; DaLiA—PPG-DaLiA database; WESAD—Wearable Stress and Affect Detection database; B1D—Bami 1 PPG dataset; B2D—Bami 2 PPG dataset; UCIMLR—UCI Machine Learning Repository; FT—Fourier transform; EMD—empirical mode decomposition; VMD—variational mode decomposition; HMM—Hidden Markov Model; NN—neural network; FD—first derivative; SD—second derivative; PCA—principal component analysis; ^*w*^—worst case; ^*B*^—best case.

Ref.	Database	Main techniques	MAE (BPM)	Comments
[71]	MIIP	EMD; FT	1.07	Mainly focused on eliminating noise due to motion.
[72]	MIP; CBD	EMD; PCA; FT	2.8	It also extracts the respiratory rate.
[73]	MIP; CBD; UQVSD	VMD; PCA; FT	1.67 ^*w*^	
[74]	SPC; DaLiA; WESAD	Sparse spectrum reconstruction; FD; SD	2.2 ^*B*^; 6.4 ^*w*^	
[75]	SPC; DaLiA; WESAD	LMS filter	2.5 ^*B*^; 4.6 ^*w*^	
[76]	MIIP; UCIMLR	NN	3.6	
[77]	SPC; B1D; B2D	FT	1.33 ^*B*^; 1.87 ^*w*^	Mainly focused on eliminating noise due to motion.
[78]	SPC; DaLiA	NN	4.03 ^*B*^; 13.53 ^*w*^	

**Table 4:** Computational cost and memory requirements. Sorted by computational cost, from left to right and from top to bottom. Acronyms: Tech.—technique; C.C.—order of computational cost; Mem.—order of the memory requirement; Ref.—reference; L—filter length; N—data length or rows of a matrix; S—number of signals or columns of a matrix (N≥S); k—levels of decomposition; M—number of iterations; P—number of data classes. U—output vector length (U≤N); *H*—number of neurons in input layer.

Tech.	C.C.	Mem.	Ref.	Tech.	C.C.	Mem.	Ref.
FIR/IIR filter	O(*L*)	O(*L*)		LMS filter	O(*L*)	O(*L*)	[80]
Median filter	O(*L*) or O(L·log(L))	O(*L*)		Sliding window integrator	O(*L*)	O(*L*)	
Data window variance	O(*L*)	O(*L*)		Shannon energy	O(*L*)	O(*L*)	
EWMV	O(*L*)	O(*L*)		CSOA	O(*L*)	O(*L*)	[55]
Zero crossing	O(*L*)	O(*L*)		Morphology-preserving data normalization	O(*N*)	O(*N*)	
Envelope filter	O(*N*)	O(*N*)	[81]	Stochastic resonance	O(*N*)	O(*N*)	[38]
Data rectification	O(*N*)	O(*N*)		Exponential transform	O(*N*)	O(*N*)	[30]
Fixed/adapted thresholding	O(*N*)	O(*N*)		WT	O(kN)	O(kN)	[81,82]
EMD	O(kMN)	O(kN)	[81]	Fourier transform	O(NlogN)	O(*N*)	
Spectrogram	O(NlogN)	O(*N*)		Hilbert transform	O(NlogN)	O(*N*)	
S-transform	O(NlogN)	O(*N*)	[83]	Dicrete cosine transform	O(NlogN)	O(*N*)	[84]
VMD	O(kMNlogN)	O(kNlogN)	[85,86]	VFCDM	O(kNlogN)	O(kN)	[62]
VCS	O(LN)	O(*N*)		Correlation	O($L^{2}$)	O(*L*)	
2D Hessian filter	O(NS)	O(NS)		2D Gaussian filter	O(NSL)	O(NS)	
Eigenvalues	O($S^{3}$)	O($S^{2}$)	[87,88]	SVD	O($NS^{2}$)	O(NS)	[88]
PCA	O($NS^{2}$)	O(NS)	[88]	Non-negative matrix factor	O(NSP)	O(NS)	[61]
FOCUSS algorithm	O($MSN^{2}$)	O($N^{2}$)	[79]	LSTM neuronal network	O($N^{2}U$)	O($N^{2}$)	[89]
Multi-layer perceptron	O(NH)	O(NH)					

**Table 5:** Comparison table of computing resources and power consumption. The * symbol refers to the multiplication operation between numbers.

Ref.	Fs (Hz)	N	D	Op.	Fs*Op/D	$I_{\mathrm{avg}}$ (mA)	Mem. (kB)
[43]	360	2048	1024	34,816	12,240.0	0.11	28.67
[60]	250	75	37.5	375	2500.0	0.11	0.75
[25]	360	3600	1800	3600	720.0	0.11	7.20
[61]	360	1800	900	540,000	216,000.0	0.33	10.80
[44]	360	512	256	2048	2880.0	0.11	4.10
[26]	360	300	1	2768.6	99,6712.4	1.16	1.20
[27]	500	60	1	120	60,000.0	0.16	0.24
[62]	360	720	360	6834.1	6834.1	0.11	1.44
[45]	360	2000	1000	240,000	86,400.0	0.19	32.00
[28]	360	43.2	1	277.9	100,045.4	0.21	0.18
[63]	500	720	360	2,300,000	3,194,444.4	3.49	64.00
[29]	360	360	1	720	259,200.0	0.38	1.44
[30]	360	72	1	144	51,840.0	0.16	0.29
[46]	360	3600	1800	1,414,400	282,880.0	0.40	243.60
[64]	360	540	270	540	720.0	0.11	1.08
[65]	360	460.8	230.4	300,000	468,750.0	0.60	12.00
[31]	360	144	1	1176.5	423,528.9	0.55	0.58
[32]	360	55.8	1	435.4	156,730.5	0.27	3.35
[33]	360	1000	500	2000	1440.0	0.11	4.00
[66]	500	3600	1800	200,000	55,555.6	0.16	7.00
[55]	360	360	180	360	720.0	0.11	0.72
[56]	512	2560	1280	5120	2048.0	0.11	10.24
[67]	250	250	125	40,000	80,000.0	0.19	6.00
[34]	360	72	1	588.2	211,764.5	0.33	0.43
[35]	360	1024	1	11,264	4,055,040.0	4.41	4.10
[47]	1000	2000	1000	8000	80,00.0	0.11	16.00
[48]	360	54	27	526.8	7023.5	0.11	0.54
[57]	360	720	360	6834.1	6834.1	0.11	1.44
[37]	250	1024	512	153,600	75,000.0	0.18	20.48
[38]	360	250	125	1250	3600.0	0.11	2.50
[49]	360	720	360	2880	2880.0	0.11	5.76
[50]	360	3600	1800	946,129.6	189,225.9	0.30	12.96
[39]	360	2048	1024	10,240	3600.0	0.11	20.48
[40]	360	720	1	1440	518,400.0	0.65	2.88
[58]	500	500	1	4982.9	2,491,446.1	2.75	2.00
[41]	360	48	1	48	17,280.0	0.12	96.00
[90]	360	30	1	30	10,800.0	0.11	2.40
[53]	360	39	1	39	14,040.0	0.12	0.08
[51]	250	512	256	4608	4500.0	0.11	1.00
[54]	360	32	1	32	11,520.0	0.11	0.06
[68]	360	360	180	80,000	160,000.0	0.27	20.00
[59]	360	70	1	140	50,400.0	0.15	0.28
[52]	360	360	180	50,000	100,000.0	0.21	50.00
[71]	125	1000	500	69,965.8	17,491.4	0.12	10.00
[72]	125	1000	500	101,1965.8	252,991.4	0.37	34.00
[73]	125	1000	500	4,994,857.9	1,248,714.5	1.43	22.24
[74]	64	512	256	1024	256.0	0.10	1.02
[75]	64	512	256	512	128.0	0.10	1.02
[77]	100	800	400	77,150.8	19,287.7	0.12	19.20
[78]	32	256	128	100,000	25,000.0	0.13	16.00

**Table 6:** Comparative summary of HR methods in terms of accuracy, computational complexity, memory requirements, and suitability for wearable devices.

Method	Accuracy	Complexity	Memory	Wearable
Time-domain methods	Very high (ECG > 99%)	O(N)	Low	Excellent
Freq.-domain methods	Moderate (PPG MAE ∈[1, 6] BPM)	O(NlogN)	Low	Good
Matrix-based methods	Comparable	$O(N^{2}$–$N^{3})$	High	Limited
ML-based methods	Comparable/lower	$O(N^{2}$–$N^{3})$	Very high	Poor (on-device)

## Data Availability

Not applicable.

## Supplementary Materials

The following supporting information can be downloaded at: https://www.mdpi.com/article/10.3390/s26082409/s1, The complete list of the articles analyzed, the reasons for exclusion, and the “Preferred Reporting Items for Systematic Reviews and Meta-Analyses: Extension for Scoping Reviews” (PRISMA-ScR) checklist. Reference [97] is cited in the supplementary materials.

## References

[1] Molina-Cantero A.J., Merino-Monge M., Castro-García J.A., Pousada-García T., Valenzuela-Muñoz D., Gutiérrez-Párraga J., López-Álvarez S., Gómez-González I.M. (2021). A Study on Physical Exercise and General Mobility in People with Cerebral Palsy: Health through Costless Routines. Int. J. Environ. Res. Public Health.

[2] Castro-García J.A., Molina-Cantero A.J., Gómez-González I.M., Lafuente-Arroyo S., Merino-Monge M. (2022). Towards Human Stress and Activity Recognition: A Review and a First Approach Based on Low-Cost Wearables. Electronics.

[3] Carrarini C., Di Stefano V., Russo M., Dono F., Di Pietro M., Furia N., Onofrj M., Bonanni L., Faustino M., De Angelis M.V. (2022). ECG monitoring of post-stroke occurring arrhythmias: An observational study using 7-day Holter ECG. Sci. Rep..

[4] Marutani Y., Konda S., Ogasawara I., Yamasaki K., Yokoyama T., Maeshima E., Nakata K. (2022). An Experimental Feasibility Study Evaluating the Adequacy of a Sportswear-Type Wearable for Recording Exercise Intensity. Sensors.

[5] Hjortskov N., Rissén D., Blangsted A.K., Fallentin N., Lundberg U., Søgaard K. (2004). The effect of mental stress on heart rate variability and blood pressure during computer work. Eur. J. Appl. Physiol..

[6] Tamura T., Maeda Y., Sekine M., Yoshida M. (2014). Wearable photoplethysmographic sensors—Past and present. Electronics.

[7] Huszar R.J. (2017). Huszar’s ECG and 12-Lead Interpretation.

[8] Lu G., Yang F., Taylor J.A., Stein J.F. (2009). A comparison of photoplethysmography and ECG recording to analyse heart rate variability in healthy subjects. J. Med. Eng. Technol..

[9] Slaman J., Roebroeck M., van der Slot W., Twisk J., Wensink A., Stam H., van den Berg-Emons R. (2014). LEARN 2 MOVE Research Grou. Can a lifestyle intervention improve physical fitness in adolescents and young adults with spastic cerebral palsy? A randomized controlled trial. Arch. Phys. Med. Rehabil..

[10] Sparrow L., Six H., Varona L., Janin O. (2021). Validation of Affect-tag Affective and Cognitive Indicators. Front. Neuroinform..

[11] Mocny-Pachońska K., Doniec R.J., Sieciński S., Piaseczna N.J., Pachoński M., Tkacz E.J. (2021). The Relationship between Stress Levels Measured by a Questionnaire and the Data Obtained by Smart Glasses and Finger Pulse Oximeters among Polish Dental Students. Appl. Sci..

[12] Tulen J.H., Man in ’t Veld A.J., van Steenis H.G., Mechelse K. (1991). Sleep patterns and blood pressure variability in patients with pure autonomic failure. Clin. Auton. Res..

[13] Cerritelli F., Frasch M.G., Antonelli M.C., Viglione C., Vecchi S., Chiera M., Manzotti A. (2021). A Review on the Vagus Nerve and Autonomic Nervous System During Fetal Development: Searching for Critical Windows. Front. Neurosci..

[14] Ceriello A., Prattichizzo F. (2021). Variability of risk factors and diabetes complications. Cardiovasc. Diabetol..

[15] Fine J., Branan K.L., Rodriguez A.J., Boonya-Ananta T., Ajmal, Ramella-Roman J.C., McShane M.J., Coté G.L. (2021). Sources of inaccuracy in photoplethysmography for continuous cardiovascular monitoring. Biosensors.

[16] Jeng M.Y., Yeh T.M., Pai F.Y. (2020). Analyzing older adults’ perceived values of using smart bracelets by means–end chain. Healthcare.

[17] Hussain S.S., Noman F., Hussain H., Ting C.M., bin Hamid S.R.G., Sh-Hussain H., Jalil M.A., Ahmad A.Z., Rizvi S.Z.H., Kipli K. (2022). A Brief Review of Computation Techniques for ECG Signal Analysis. Proceedings of the Third International Conference on Trends in Computational and Cognitive Engineering; Lecture Notes in Networks and Systems.

[18] Tripathi P.M., Kumar A., Komaragiri R., Kumar M. (2022). A Review on Computational Methods for Denoising and Detecting ECG Signals to Detect Cardiovascular Diseases. Arch. Comput. Methods Eng..

[19] Obi A.I. (2022). An Overview of Wearable Photoplethysmographic Sensors and Various Algorithms for Tracking of Heart Rates. Eng. Proc..

[20] Scardulla F., Cosoli G., Spinsante S., Poli A., Iadarola G., Pernice R., Busacca A., Pasta S., Scalise L., D’Acquisto L. (2023). Photoplethysmograhic sensors, potential and limitations: Is it time for regulation? A comprehensive review. Measurement.

[21] Arksey H., O’Malley L. (2005). Scoping studies: Towards a methodological framework. Int. J. Soc. Res. Methodol. Theory Pract..

[22] Levac D., Colquhoun H., O’Brien K.K. (2010). Scoping studies: Advancing the methodology. Implement. Sci..

[23] Peters M., Godfrey C., Mcinerney P., Soares C., Khalil H., Parker D. (2015). Methodology for JBI Scoping Reviews. The Joanna Briggs Institute Reviewers’ Manual.

[24] Sörnmo L., Laguna P. (2005). Bioelectrical Signal Processing in Cardiac and Neurological Applications.

[25] Khadirnaikar S., Aparna P. (2017). A feasible QRS detection algorithm for arrhythmia diagnosis. Proceedings of the 2016 International Conference on Advances in Electrical, Electronic and Systems Engineering, ICAEES 2016.

[26] Nayak C., Saha S.K., Kar R., Mandal D. (2018). Automated QRS complex detection using MFO-based DFOD. IET Signal Process..

[27] Burguera A. (2019). Fast QRS Detection and ECG Compression Based on Signal Structural Analysis. IEEE J. Biomed. Health Inform..

[28] Razzaq Hussein E.A., Hassooni A.S., Al-Libawy H. (2019). Detection of electrocardiogram QRS complex based on modified adaptive threshold. Int. J. Electr. Comput. Eng..

[29] Kurniawan A., Yuniarno E.M., Setijadi E., Yusuf M., Ketut Eddy Purnama I. (2020). QVAT: QRS Complex Detection based on Variance Analysis and Adaptive Threshold for Electrocardiogram Signal. Proceedings of the Proceedings—2020 International Seminar on Intelligent Technology and Its Application: Humanification of Reliable Intelligent Systems, ISITIA 2020.

[30] Chen A., Zhang Y., Zhang M., Liu W., Chang S., Wang H., He J., Huang Q. (2020). A real time QRS detection algorithm based on ET and PD controlled threshold strategy. Sensors.

[31] Paul A., Das N., Pal S., Mitra M. (2025). Automated Detection of Cardinal Points of ECG Signal for Feature Extraction Using a Single Median Filter. J. Inst. Eng. (India) Ser. B.

[32] Xu W., Du F. (2022). A robust QRS complex detection method based on Shannon energy envelope and Hilbert transform. J. Mech. Med. Biol..

[33] Rahul J., Sora M., Sharma L.D. (2021). A novel and lightweight P, QRS, and T peaks detector using adaptive thresholding and template waveform. Comput. Biol. Med..

[34] Ma J., Wang X., Wu X., Zhao T., Li Q. (2019). Design and implementation of a novel r-peak detection algorithm. Proceedings of the Proceedings—10th International Conference on Information Technology in Medicine and Education, ITME 2019.

[35] Singh N., Deora P., Pradhan P.M. (2019). Simultaneously Concentrated PSWF-based Synchrosqueezing S-transform and its application to R peak detection in ECG signal. Proceedings of the 2019 28th IEEE International Conference on Robot and Human Interactive Communication, RO-MAN 2019.

[36] Chen H., Maharatna K. (2020). An Automatic R and T Peak Detection Method Based on the Combination of Hierarchical Clustering and Discrete Wavelet Transform. IEEE J. Biomed. Health Inform..

[37] Hadji S. (2021). R wave localization from transformed Electrocardiogram signal by EMD. Proceedings of the International Conference on Electrical, Computer and Energy Technologies (ICECET).

[38] Güngör C.B., Mercier P.P., Töreyin H. (2022). A Stochastic Resonance Electrocardiogram Enhancement Algorithm for Robust QRS Detection. IEEE J. Biomed. Health Inform..

[39] Merino-Monge M., Castro-García J.A., Lebrato-Vázquez C., Gómez-González I.M., Molina-Cantero A.J. (2023). Heartbeat detector from ECG and PPG signals based on wavelet transform and upper envelopes. Phys. Eng. Sci. Med..

[40] Zhai D., Bao X., Long X., Ru T., Zhou G. (2023). Precise detection and localization of R-peaks from ECG signals. Math. Biosci. Eng..

[41] Chen C.L., Chuang C.T. (2017). A QRS detection and R point recognition method for wearable single-lead ECG devices. Sensors.

[42] Doyen M., Ge D., Beuchée A., Carrault G., Hernández A.I. (2019). Robust, real-time generic detector based on a multi-feature probabilistic method. PLoS ONE.

[43] Rakshit M., Das S. (2017). An efficient wavelet-based automated R-peaks detection method using Hilbert transform. Biocybern. Biomed. Eng..

[44] Rodriguez V.H., Medrano C., Plaza I. (2018). A Real-Time QRS Complex Detector Based on Discrete Wavelet Transform and Adaptive Threshold as Standalone Application on ARM Microcontrollers. Proceedings of the 2018 International Conference on Biomedical Engineering Applications, ICBEA 2018—Proceedings.

[45] Hossain M.B., Bashar S.K., Walkey A.J., McManus D.D., Chon K.H. (2019). An Accurate QRS Complex and P Wave Detection in ECG Signals Using Complete Ensemble Empirical Mode Decomposition with Adaptive Noise Approach. IEEE Access.

[46] Liu W., Wang X., Gao H., Yang C., Li J., Liu C. (2020). An Octave Convolution Neural Network-based QRS Detector. Proceedings of the International Conference on Sensing, Measurement and Data Analytics in the Era of Artificial Intelligence, ICSMD 2020—Proceedings.

[47] Banerjee S. (2019). A First Derivative Based R-Peak Detection and DWT Based Beat Delineation Approach of Single Lead Electrocardiogram Signal. Proceedings of the 2019 IEEE Region 10 Symposium, TENSYMP 2019.

[48] Modak S., Taha L.Y., Abdel-Raheem E. (2020). Single Channel QRS Detection Using Wavelet and Median Denoising with Adaptive Multilevel Thresholding. Proceedings of the 2020 IEEE International Symposium on Signal Processing and Information Technology, ISSPIT 2020.

[49] Li G., Huang D., Wang L., Zhou J., Chen J., Wu K., Xu W. (2022). A new method of detecting the characteristic waves and their onset and end in electrocardiogram signals. Biomed. Signal Process. Control.

[50] Varghees V.N., Cao H., Peyrodie L. (2023). Variational Mode Decomposition-Based Simultaneous R Peak Detection and Noise Suppression for Automatic ECG Analysis. IEEE Sens. J..

[51] Park J.S., Lee S.W., Park U. (2017). R Peak Detection Method Using Wavelet Transform and Modified Shannon Energy Envelope. J. Healthc. Eng..

[52] Zahid M.U., Kiranyaz S., Ince T., Devecioglu O.C., Chowdhury M.E., Khandakar A., Tahir A., Gabbouj M. (2022). Robust R-Peak Detection in Low-Quality Holter ECGs Using 1D Convolutional Neural Network. IEEE Trans. Biomed. Eng..

[53] Pandit D., Zhang L., Liu C., Chattopadhyay S., Aslam N., Lim C.P. (2017). A lightweight QRS detector for single lead ECG signals using a max-min difference algorithm. Comput. Methods Programs Biomed..

[54] Jain S., Ahirwal M.K., Kumar A., Bajaj V., Singh G.K. (2017). QRS detection using adaptive filters: A comparative study. ISA Trans..

[55] Jain S., Kumar A., Bajaj V. (2018). QRS complex detection using cuckoo search optimization algorithm. Proceedings of the 2017 IEEE International Conference on Communication and Signal Processing, ICCSP 2017.

[56] Lee M., Park D., Dong S.Y., Youn I. (2018). A Novel R Peak Detection Method for Mobile Environments. IEEE Access.

[57] Modak S., Taha L.Y., Abdel-Raheem E. (2021). A Novel Method of QRS Detection Using Time and Amplitude Thresholds with Statistical False Peak Elimination. IEEE Access.

[58] Tien D.H., Tan M.P.N., Nam T.H., Tai T.V., Nguyen T.T.T. (2023). A Low-Complexity R-peak Detection Based on Exponential Weight Mean-Variance for Wearable ECG Devices. Proceedings of the International Conference on Advanced Technologies for Communications.

[59] Wu L., Xie X., Wang Y. (2021). ECG enhancement and r-peak detection based on window variability. Healthcare.

[60] Rekik S., Ellouze N. (2017). Enhanced and Optimal Algorithm for QRS Detection. IRBM.

[61] Guyot P., Voiriot P., Djermoune E.H., Papelier S., Lessard C., Felices M., Bastogne T. (2018). R-Peak Detection in Holter ECG Signals Using Non-Negative Matrix Factorization. Proceedings of the Computing in Cardiology.

[62] Bashar S.K., Noh Y., Walkey A.J., Mcmanus D.D., Chon K.H. (2019). VERB: VFCDM-Based Electrocardiogram Reconstruction and Beat Detection Algorithm. IEEE Access.

[63] Cai W., Hu D. (2020). QRS complex detection using novel deep learning neural networks. IEEE Access.

[64] Bae T.W., Kwon K.K. (2021). ECG PQRST complex detector and heart rate variability analysis using temporal characteristics of fiducial points. Biomed. Signal Process. Control.

[65] Belkadi M.A., Daamouche A., Melgani F. (2021). A deep neural network approach to QRS detection using autoencoders. Expert Syst. Appl..

[66] Liang X., Li L., Liu Y., Chen D., Wang X., Hu S., Wang J., Zhang H., Sun C., Liu C. (2022). ECG_SegNet: An ECG delineation model based on the encoder-decoder structure. Comput. Biol. Med..

[67] Abrishami H., Campbell M., Han C., Czosek R., Zhou X. (2018). P-QRS-T localization in ECG using deep learning. Proceedings of the 2018 IEEE EMBS International Conference on Biomedical and Health Informatics, BHI 2018.

[68] Xiang Y., Lin Z., Meng J. (2018). Automatic QRS complex detection using two-level convolutional neural network. BioMed. Eng. Online.

[69] Sahoo S., Mohanty M., Behera S., Sabut S.K. (2017). ECG beat classification using empirical mode decomposition and mixture of features. J. Med. Eng. Technol..

[70] Chen Y., Fan H., Xu B., Yan Z., Kalantidis Y., Rohrbach M., Shuicheng Y., Feng J. (2019). Drop an octave: Reducing spatial redundancy in convolutional neural networks with octave convolution. Proceedings of the IEEE International Conference on Computer Vision.

[71] Pang B., Liu M., Zhang X., Li P., Chen H. (2018). A novel approach framework based on statistics for reconstruction and heartrate estimation from PPG with heavy motion artifacts. Sci. China Inf. Sci..

[72] Motin M.A., Karmakar C.K., Palaniswami M. (2018). Ensemble empirical mode decomposition with principal component analysis: A novel approach for extracting respiratory rate and heart rate from photoplethysmographic signal. IEEE J. Biomed. Health Inform..

[73] Sharma H. (2019). Heart rate extraction from PPG signals using variational mode decomposition. Biocybern. Biomed. Eng..

[74] Zhou M., Selvaraj N. (2020). Heart Rate Monitoring using Sparse Spectral Curve Tracing. Proceedings of the Annual International Conference of the IEEE Engineering in Medicine and Biology Society, EMBS.

[75] Huang N., Selvaraj N. (2020). Robust PPG-based Ambulatory Heart Rate Tracking Algorithm. Proceedings of the Annual International Conference of the IEEE Engineering in Medicine and Biology Society, EMBS.

[76] Yen C.T., Liao C.H. (2022). Blood pressure and heart rate measurements using photoplethysmography with modified lrcn. Comput. Mater. Contin..

[77] Pankaj, Kumar A., Komaragiri R., Kumar M. (2022). Reference signal less Fourier analysis based motion artifact removal algorithm for wearable photoplethysmography devices to estimate heart rate during physical exercises. Comput. Biol. Med..

[78] Kasnesis P., Toumanidis L., Burrello A., Chatzigeorgiou C., Patrikakis C.Z. (2023). Feature-Level Cross-Attentional PPG and Motion Signal Fusion for Heart Rate Estimation. Proceedings of the Proceedings—International Computer Software and Applications Conference.

[79] Gorodnitsky I.F., Rao B.D. (1997). Sparse signal reconstruction from limited data using FOCUSS: A re-weighted minimum norm algorithm. IEEE Trans. Signal Process..

[80] Da Silveira Coelho L.F., Lovisolo L., Tcheou M.P. (2021). Adaptive filtering with reduced computational complexity using SOPOT arithmetic. IEEE Trans. Circuits Syst. I Regul. Pap..

[81] Wang Y.H., Yeh C.H., Young H.W.V., Hu K., Lo M.T. (2014). On the computational complexity of the empirical mode decomposition algorithm. Phys. A Stat. Mech. Its Appl..

[82] Latu G. (2011). Sparse data structure design for wavelet-based methods. ESAIM Proc..

[83] Brown R.A., Frayne R. (2008). A fast discrete S-transform for biomedical signal processing. Proceedings of the 2008 30th Annual International Conference of the IEEE Engineering in Medicine and Biology Society.

[84] Chen W.H., Smith C., Fralick S. (1977). A Fast Computational Algorithm for the Discrete Cosine Transform. IEEE Trans. Commun..

[85] Dragomiretskiy K., Zosso D. (2014). Variational mode decomposition. IEEE Trans. Signal Process..

[86] Zhang L., Xu F., Wang R., Huang X. (2023). Kubernetes Edge Server Resource Prediction Model Integrating Vmd and Lstm. SSRN.

[87] Pan V.Y., Chen Z.Q. (1999). The complexity of the matrix eigenproblem. Conference Proceedings of the Annual ACM Symposium on Theory of Computing.

[88] Li X., Wang S., Cai Y. (2019). Tutorial: Complexity analysis of Singular Value Decomposition and its variants. arXiv.

[89] Petrozziello A., Troiano L., Serra A., Jordanov I., Storti G., Tagliaferri R., La Rocca M. (2022). Deep learning for volatility forecasting in asset management. Soft Comput..

[90] Hamdi S., Ben Abdallah A., Bedoui M.H. (2017). Real time QRS complex detection using DFA and regular grammar. Biomed. Eng. Online.

[91] Cadence Design Systems, Inc. (2016). Xtensa LX6 Product Brief.

[92] Gil E., Orini M., Bailón R., Vergara J.M., Mainardi L., Laguna P. (2010). Photoplethysmography pulse rate variability as a surrogate measurement of heart rate variability during non-stationary conditions. Physiol. Meas..

[93] Schäfer A., Vagedes J. (2013). How accurate is pulse rate variability as an estimate of heart rate variability?: A review on studies comparing photoplethysmographic technology with an electrocardiogram. Int. J. Cardiol..

[94] Zhou Y., Lindsey B., Snyder S., Bell E., Reider L., Vignos M., Bar-Kochba E., Mousavi A., Parreira J., Hanley C. (2024). Sampling rate requirement for accurate calculation of heart rate and its variability based on the electrocardiogram. Physiol. Meas..

[95] Pelaez-Coca M.D., Hernando A., Lazaro J., Gil E. (2022). Impact of the PPG Sampling Rate in the Pulse Rate Variability Indices Evaluating Several Fiducial Points in Different Pulse Waveforms. IEEE J. Biomed. Health Inform..

[96] Luo X., Miao F., Li Y. (2012). PPG and ECG feature comparison for healthy people and hypertensive patients. Proceedings of the Proceedings—IEEE-EMBS International Conference on Biomedical and Health Informatics: Global Grand Challenge of Health Informatics, BHI 2012.

[97] Tricco A.C., Lillie E., Zarin W., O’Brien K.K., Colquhoun H., Levac D., Moher D., Peters M.D.J., Horsley T., Weeks L. (2018). PRISMA Extension for Scoping Reviews (PRISMAScR): Checklist and Explanation. Ann. Intern. Med..

