# Supplementary material for: Computational and Memory Efficiency in Heartbeat Rate Detection: A Review of ECG and PPG Techniques
Source: Sensors (Basel). 2026 Apr 14;26(8):2409. doi: 10.3390/s26082409 (PMC13120323; doi:10.3390/s26082409)
Supplement: Supplementary file 1 [file sensors-26-02409-s001.zip › sensors-4206802-supplementary.pdf]

Preferred Reporting Items for Systematic reviews and Meta-Analyses extension for Scoping Reviews (PRISMA-ScR) Checklist

SECTION	ITEM	PRISMA-ScR CHECKLIST ITEM	REPORTED ON PAGE #
**TITLE**			
Title	1	Identify the report as a scoping review.	Computational and Memory Efficiency in Heartbeat-Rate Detection: A Review of ECG and PPG Techniques
**ABSTRACT**			
Structured summary	2	Provide a structured summary that includes (as applicable): background, objectives, eligibility criteria, sources of evidence, charting methods, results, and conclusions that relate to the review questions and objectives.	**Background:** Heartbeat-rate (HR) estimation using electrocardiogram (ECG) and photoplethysmograph (PPG) signals is widely applied in clinical monitoring, sports performance assessment, and stress evaluation. With the rapid expansion of wearable devices, there is increasing interest in identifying techniques that not only provide high accuracy but are also feasible for implementation on resource-constrained platforms.<br><br>**Objectives:** This scoping review aims to map and compare heartbeat-rate detection techniques based on ECG and PPG signals, focusing on their computational cost, memory requirements, and suitability for embedded and wearable devices.<br><br>**Eligibility Criteria:** Studies published between 2017 and 2024 addressing HR estimation from ECG or PPG signals were included. Both traditional signal-processing approaches and machine learning-based methods were considered. Studies not focused on HR calculation or lacking methodological detail were excluded.<br><br>**Sources of Evidence:** A systematic search was conducted in three scientific databases (Scopus, IEEE, PubMed) in accordance with the PRISMA-ScR guidelines.<br><br>**Charting Methods:** For each study, data were extracted regarding signal type (ECG/PPG), methodological approach, reported performance metrics (e.g., accuracy, mean absolute error), computational complexity, memory requirements, and implementation context.<br><br>**Results:** 52 papers were included in this study. Linear-complexity techniques—such as FIR/IIR filters, median and adaptive filters, sliding window integration, and

St. Michael's
Inspired Care.
Inspiring Science.

SECTION	ITEM	PRISMA-ScR CHECKLIST ITEM	REPORTED ON PAGE #
			thresholding—demonstrated high accuracy (above 99.92% for ECG and below 3 BPM MAE for PPG) with minimal memory and energy demands, making them highly suitable for wearable devices. In contrast, machine learning and matrix-based methods typically required greater computational and memory resources, often without consistent performance improvements.<br><br>**Conclusions:** For wearable and embedded applications, linear-cost signal-processing techniques provide the most favorable balance between accuracy and resource efficiency. More computationally intensive methods may be better suited for edge or cloud-based implementations.
**INTRODUCTION**			
Rationale	3	Describe the rationale for the review in the context of what is already known. Explain why the review questions/objectives lend themselves to a scoping review approach.	Heartbeat-rate (HR) estimation from electrocardiogram (ECG) and photoplethysmograph (PPG) signals has been extensively studied, with numerous techniques proposed to improve detection accuracy under diverse clinical and ambulatory conditions. Existing literature largely focuses on signal-processing performance, noise reduction strategies, and, more recently, machine learning-based approaches. However, much of the available evidence emphasizes accuracy metrics without systematically addressing computational complexity, memory footprint, and energy consumption—factors that are critical for implementation on resource-constrained wearable devices such as smartwatches and fitness trackers. As wearable health technologies continue to expand, there is a growing need to evaluate HR detection methods not only in terms of diagnostic performance but also in terms of feasibility for embedded systems with limited memory capacity and battery autonomy.<br><br>The review questions and objectives of this study are particularly suited to a scoping review approach because the field encompasses a broad range of methodological frameworks, performance metrics, hardware platforms, and application contexts. The heterogeneity in study designs, evaluation protocols, and reported outcomes makes direct quantitative comparison or meta-analysis inappropriate. Instead, a

SECTION	ITEM	PRISMA-ScR CHECKLIST ITEM	REPORTED ON PAGE #
			scoping review allows for systematic mapping of the available evidence, identification of dominant methodological trends, and clarification of how different algorithmic families address implementation constraints. This approach is therefore well aligned with the objective of providing an overview of existing techniques, highlighting gaps in the literature, and informing future research directions in wearable HR monitoring systems.
Objectives	4	Provide an explicit statement of the questions and objectives being addressed with reference to their key elements (e.g., population or participants, concepts, and context) or other relevant key elements used to conceptualize the review questions and/or objectives.	How can heartbeats be detected in the ECG and PPG signals?<br><br>What computational limitations do I have? What energy limitations do I have?
**METHODS**			
Protocol and registration	5	Indicate whether a review protocol exists; state if and where it can be accessed (e.g., a Web address); and if available, provide registration information, including the registration number.	https://osf.io/6ex2r
Eligibility criteria	6	Specify characteristics of the sources of evidence used as eligibility criteria (e.g., years considered, language, and publication status), and provide a rationale.	In the first phase, duplicate results were eliminated. Then, two researchers conducted a screening process in which we discarded those: a) that did not fit our topic of interest; b) whose results were not reproducible because they did not use public or available databases; c) in which the accuracy of the technique was missing; d) that were only focused on hardware. Then, studies were selected to be included in this review after eliminating those that were not accessible, described other topics, do not use available databases, lacked details in the explanation of the techniques, used more than one channel of ECG or PPG, or did not propose a new technique for detecting QRS complexes.
Information sources*	7	Describe all information sources in the search (e.g., databases with dates of coverage and contact with authors to identify additional sources), as well as the date the most recent search was executed.	Scopus, IEEE Xplorer, PubMed
Search	8	Present the full electronic search	“ECG” AND “detect” AND “qrs” from

SECTION	ITEM	PRISMA-ScR CHECKLIST ITEM	REPORTED ON PAGE #
		strategy for at least 1 database, including any limits used, such that it could be repeated.	2017 to August 1, 2024<br><br>“PPG” AND (“hr” OR “heartbeat rate”) from 2017 to August 1, 2024
Selection of sources of evidence†	9	State the process for selecting sources of evidence (i.e., screening and eligibility) included in the scoping review.	Round 1) selection by title that fits the objective of the study; Round 2) selection by abstract that fits what is being sought; Round 3) reading of the entire document.
Data charting process‡	10	Describe the methods of charting data from the included sources of evidence (e.g., calibrated forms or forms that have been tested by the team before their use, and whether data charting was done independently or in duplicate) and any processes for obtaining and confirming data from investigators.	Three researchers assessed the suitability of the studies in each round. In case of ambiguity, the corresponding author made the final decision.
Data items	11	List and define all variables for which data were sought and any assumptions and simplifications made.	accuracy, mean absolut error, computational load, memory resources.
Critical appraisal of individual sources of evidence§	12	If done, provide a rationale for conducting a critical appraisal of included sources of evidence; describe the methods used and how this information was used in any data synthesis (if appropriate).	None
Synthesis of results	13	Describe the methods of handling and summarizing the data that were charted.	Extraction of information directly from the article, and analysis of the algorithms used to determine hardware requirements. No attempt has been made to reduce the analysis time in order to maintain scientific rigour. Conclusions based on data extracted directly from the articles and organised into summary tables in the article. Only one person has completed the final synthesis.
**RESULTS**			
Selection of sources of evidence	14	Give numbers of sources of evidence screened, assessed for eligibility, and included in the review, with reasons for exclusions at each stage, ideally using a flow diagram.	For each database, two searches were conducted, limiting results to the period between 2017 and August 1, 2024. A total of 3189 articles were retrieved: 1155 from Scopus (550 ECG-based and 605 PPG-based), 1024 from IEEE Xplore (614 ECG-based and 410 PPG-based), and 1010 from PubMed (759 ECG-based and 251 PPG-based). After removing 830 duplicate records, a total of 2359 articles remained for screening. No formal quality assessment of the

SECTION	ITEM	PRISMA-ScR CHECKLIST ITEM	REPORTED ON PAGE #
			included studies was performed.<br><br>A total of 2359 articles were analyzed. A screening process based on the title and abstract was conducted, after which 122 papers advanced to the full-text review phase. Of these, 52 were finally chosen to form part of this review after eliminating those that were not accessible (17 papers), described other topics (8 papers), do not use available databases (11 papers), lacked details in the explanation of the techniques (11 papers), used more than one channel of \gls{ECG} or \gls{PPG} (14 papers), or did not propose a new technique for detecting QRS complexes, but rather employed techniques analyzed in other articles identified in this search (9 papers).
Characteristics of sources of evidence	15	For each source of evidence, present characteristics for which data were charted and provide the citations.	a) Results were not reproducible because they did not use public or available databases; b) the accuracy of the technique; c) not focused on hardware; d) one channel of ECG or PPG; e) propose a new technique for detecting QRS complexes.
Critical appraisal within sources of evidence	16	If done, present data on critical appraisal of included sources of evidence (see item 12).	None
Results of individual sources of evidence	17	For each included source of evidence, present the relevant data that were charted that relate to the review questions and objectives.	the relevant data charted from each included study are presented to directly address the review questions and objectives. For each source of evidence, we extracted and summarized information regarding: (i) the physiological signal analyzed (ECG or PPG), (ii) the heartbeat-rate detection method employed, (iii) reported performance metrics (e.g., accuracy, mean absolute error), (iv) computational complexity, (v) memory requirements, and (vi) suitability for implementation on resource-constrained wearable devices.<br><br>These data are systematically presented in Tables 1, 2 and 3, where each study is mapped to its methodological approach and implementation characteristics. The structured presentation enables comparison across studies and facilitates the identification of dominant methodological trends, including linear-complexity signal-

SECTION	ITEM	PRISMA-ScR CHECKLIST ITEM	REPORTED ON PAGE #
			processing techniques, time–frequency methods, matrix-based algorithms, and machine learning models.<br><br>By explicitly organizing the extracted data according to performance and implementation constraints, the synthesis directly responds to the central objective of this scoping review: to evaluate heartbeat-rate detection techniques not only in terms of accuracy, but also in terms of feasibility for embedded and wearable platforms. This approach ensures transparency in the evidence-charting process.
Synthesis of results	18	Summarize and/or present the charting results as they relate to the review questions and objectives.	time-domain peak detection methods consistently provide high accuracy for ECG and reliable heart rate estimation for PPG with linear computational complexity and low memory requirements. Frequency-domain approaches are suitable for average heart rate estimation from PPG but do not preserve inter-beat intervals. Matrix-based and machine learning methods often entail higher computational cost without proportional performance gains in wearable contexts
**DISCUSSION**			
Summary of evidence	19	Summarize the main results (including an overview of concepts, themes, and types of evidence available), link to the review questions and objectives, and consider the relevance to key groups.	The main results of this review show that heartbeat-rate estimation from ECG and PPG signals can achieve very high accuracy when appropriate signal-processing techniques are selected, but their suitability for wearable implementation strongly depends on computational complexity and memory requirements. Across the 52 studies analyzed (2017–2024), two major methodological trends were identified: traditional signal-processing approaches (e.g., FIR/IIR filters, adaptive and median filters, sliding window integration, and thresholding) and machine learning or matrix-based methods. The evidence consistently indicates that linear-complexity techniques provide an optimal balance between accuracy, energy consumption, and memory footprint, achieving detection rates above 99.9% for ECG and mean absolute errors below 3 BPM for PPG. In contrast, although machine learning approaches are increasingly explored, the available evidence shows that they often require substantially greater computational and memory resources while offering comparable or even

SECTION	ITEM	PRISMA-ScR CHECKLIST ITEM	REPORTED ON PAGE #
			lower performance in wearable contexts.<br><br>These findings directly address the review objective of evaluating heartbeat-rate detection methods not only in terms of accuracy but also in terms of feasibility for resource-constrained devices. The results are particularly relevant for engineers designing embedded and wearable systems, healthcare professionals interested in reliable remote monitoring, and researchers developing mobile health solutions. Overall, the evidence supports prioritizing low-complexity, energy-efficient algorithms for on-device implementation, while reserving more demanding approaches for edge or cloud-based processing environments.
Limitations	20	Discuss the limitations of the scoping review process.	Several limitations of the scoping review process should be acknowledged. First, the review was restricted to studies published between 2017 and 2024, which may have excluded earlier foundational work that continues to influence current heartbeat-rate estimation techniques. Second, although predefined inclusion and exclusion criteria were applied, the selection process may still be subject to publication bias and database coverage limitations, potentially leading to the omission of relevant studies not indexed in the selected sources. Third, as a scoping review, the objective was to map and categorize the available evidence rather than to conduct a formal quality assessment or quantitative meta-analysis; therefore, the methodological rigor of individual studies was not systematically evaluated, and performance metrics were not statistically pooled.<br><br>In addition, heterogeneity across the included studies—regarding datasets, signal acquisition conditions, hardware platforms, evaluation metrics, and subject populations—limits the direct comparability of results. Reported accuracy, mean absolute error, and computational requirements were often obtained under different experimental setups, which may affect generalizability

SECTION	ITEM	PRISMA-ScR CHECKLIST ITEM	REPORTED ON PAGE #
			to real-world wearable environments. Finally, rapid technological evolution in wearable hardware and machine learning techniques means that some conclusions, particularly regarding resource constraints, may evolve as more efficient implementations become available.
Conclusions	21	Provide a general interpretation of the results with respect to the review questions and objectives, as well as potential implications and/or next steps.	Lightweight signal-processing techniques offer the most favorable trade-off between accuracy and efficiency for wearable implementations, whereas computationally intensive approaches are better suited for edge or cloud-based processing.
**FUNDING**			
Funding	22	Describe sources of funding for the included sources of evidence, as well as sources of funding for the scoping review. Describe the role of the funders of the scoping review.	This publication is part of the project PID2023-147508OB-I00 (BRet(IA)2), funded by 484 MICIU/AEI/ 10.13039/501100011033 and by ERDF/EU.<br><br>Funding did not influence the study.

JBI = Joanna Briggs Institute; PRISMA-ScR = Preferred Reporting Items for Systematic reviews and Meta-Analyses extension for Scoping Reviews.
* Where *sources of evidence* (see second footnote) are compiled from, such as bibliographic databases, social media platforms, and Web sites.
† A more inclusive/heterogeneous term used to account for the different types of evidence or data sources (e.g., quantitative and/or qualitative research, expert opinion, and policy documents) that may be eligible in a scoping review as opposed to only studies. This is not to be confused with *information sources* (see first footnote).
‡ The frameworks by Arksey and O'Malley (6) and Levac and colleagues (7) and the JBI guidance (4, 5) refer to the process of data extraction in a scoping review as data charting.
§ The process of systematically examining research evidence to assess its validity, results, and relevance before using it to inform a decision. This term is used for items 12 and 19 instead of "risk of bias" (which is more applicable to systematic reviews of interventions) to include and acknowledge the various sources of evidence that may be used in a scoping review (e.g., quantitative and/or qualitative research, expert opinion, and policy document).

From: Tricco AC, Lillie E, Zarin W, O'Brien KK, Colquhoun H, Levac D, et al. PRISMA Extension for Scoping Reviews (PRISMAScR): Checklist and Explanation. Ann Intern Med. 2018;169:467–473. doi: 10.7326/M18-0850.

DOCUMENT TITLE	YEAR	DOI	DATA BASE	INCLUSION/EXCLUSION	ASSESSED FULL TEXT
optimized stockwell transform for the segmentation of ecg signals	2017	10.1049/ cp.2017.0365	IEEE	off topic	no
dedicated wavelet qrs complex detection for fpga implementation	2017	10.1109/ atsip.2017.8075543	IEEE	hardware	no
deep learning based classification for healthcare data analysis system	2017	10.1109/ besc.2017.8256396	IEEE	off topic	no
integrating markov model and morphology analysis for finer classification of ventricular arrhythmia in real time	2017	10.1109/ bhi.2017.7897292	IEEE	off topic	no
differences between model-based electrocardiogram t wave features before and after haemodialysis	2017	10.1109/ biocas.2017.832508 5	IEEE	off topic	no
wearable bio-sensors bracelet for driveras health emergency detection	2017	10.1109/ biosmart.2017.8095 335	IEEE	off topic	no
android based warning system for the early detection of allergic reactions	2017	10.1109/ bsn.2017.7936027	IEEE	off topic	no
implementation of an arrhythmia detection scheme with cellular based alert framework	2017	10.1109/ calcon.2017.828077 1	IEEE	off topic	no
hamming filter design for ecg signal detection and processing using co-simulation	2017	10.1109/ ccaa.2017.8229992	IEEE	duplicate	no
design of wi-fi based ecg system	2017	10.1109/ cspc.2017.8305826	IEEE	off topic	no
ecg signal for artrial fibrillation detection	2017	10.1109/ ecace.2017.7913036	IEEE	off topic	no
ultra-low power qrs detection using adaptive thresholding based on forward search interval technique	2017	10.1109/ edssc.2017.8126486	IEEE	duplicate	no
analysis of qrs detection algorithms barely sensitive to the qrs shape	2017	10.1109/ eiconrus.2017.79106 63	IEEE	duplicate	no
pulse arrival time (pat) measurement based on arm ecg and finger ppg signals - comparison of ppg feature detection methods for pat calculation	2017	10.1109/ embc.2017.8036809	IEEE	off topic	no

disease checking method of ecg signals with variable output resolutions for wearable devices	2017	10.1109/ gcce.2017.8229385	IEEE	off topic	no
the design of wearable sleep apnea monitoring wrist watch	2017	10.1109/ healthcom.2017.821 0850	IEEE	off topic	no
a novel cardiac arrhythmia detection method relying on improved dtw method	2017	10.1109/ iaeac.2017.8054137	IEEE	off topic	no
r peak detection and feature extraction for the diagnosis of heart diseases	2017	10.1109/ icacci.2017.8126204	IEEE	off topic	no
real-time monitoring of heart conditions via electrocardiogram processing at different lifestyle situations	2017	10.1109/ ICAEE.2017.825541 6	IEEE	hardware	no
development of real-time ecg signal monitoring system for telemedicine application	2017	10.1109/ icbsii.2017.8082285	IEEE	mutichannel	no
portable ecg nalysis	2017	10.1109/ iccons.2017.825057 6	IEEE	hardware	no
ecg based algorithm for detecting ventricular arrhythmia and atrial fibrillation	2017	10.1109/ iccons.2017.825077 3	IEEE	off topic	no
mobile ecg monitoring device using wearable non contact armband	2017	10.1109/ iccpct.2017.8074335	IEEE	off topic	no
qrs complex detection using cuckoo search optimization algorithm	2017	10.1109/ iccsp.2017.8286606	IEEE	INCLUDED	yes
solar ecg acquisition system	2017	10.1109/ iceca.2017.8203595	IEEE	off topic	no
electrocardiographic signal as a biometrie feature	2017	10.1109/ iceee.2017.8108877	IEEE	off topic	no
multilead qt interval analysis algorithm based on continuous wavelet transform	2017	10.1109/ iceee2.2017.793584 2	IEEE	duplicate	no
fault detection and isolation of ecg process using kernel principal components	2017	10.1109/ icemis.2017.827308 7	IEEE	off topic	no
sleep order detection model using support vector machines and features extracted from brain ecg signals	2017	10.1109/ icici.2017.8365290	IEEE	duplicate	no

obstructive sleep apnea detection from ecg signal using neuro-fuzzy classifier	2017	10.1109/ icicict1.2017.834268 6	IEEE	duplicate	no
detect qrs complex in ecg	2017	10.1109/ iciea.2017.8283170	IEEE	duplicate	no
fetal heart abnormality detection based on subspace separation and wiener filtering	2017	10.1109/ iciinfs.2017.8300349	IEEE	fetal	no
classification of normal and abnormal ecg signals based on their pqrst intervals	2017	10.1109/ icmsc.2017.7959507	IEEE	off topic	no
study of r peaks using hrv processor in matlab simulink	2017	10.1109/ icoei.2017.8300828	IEEE	off topic	no
R - peak detection using bayesian regularization neural network	2017	10.1109/ icomicon.2017.8279 025	IEEE	duplicate	no
design of a temporay external artificial pacemaker simulator	2017	10.1109/ icoras.2017.8308061	IEEE	off topic	no
ecg signal based power aware system for obstructive sleep apnea detection	2017	10.1109/ icrteect.2017.43	IEEE	duplicate	no
an improved template-matching method for real-time detection of abnormal heartbeats	2017	10.1109/ ifuzzy.2017.8311809	IEEE	off topic	no
multiple ecg fiducial points-based random binary sequence generation for securing wireless body area networks	2017	10.1109/ jbhi.2016.2546300	IEEE	duplicate	no
qrs complex detection based on primitive	2017	10.1109/ jcn.2017.000076	IEEE	animal	no
r-peak detection using efficient technique for tachycardia detection	2017	10.1109/ mami.2017.8307877	IEEE	duplicate	no
application of savitzky-golay digital differentiator for qrs complex detection in an electrocardiographic monitoring system	2017	10.1109/ memea.2017.79858 81	IEEE	duplicate	no
fpga-based peak detection of ecg signal using histogram approach	2017	10.1109/ rise.2017.8378200	IEEE	hardware	no
development of ecg home monitoring system	2017	10.1109/ rsm.2017.8069119	IEEE	hardware	no
drowsiness prediction system for vehicle using capacity coupled electrode type non-invasive ecg measurement	2017	10.1109/ sii.2017.8279230	IEEE	off topic	no

extraction of fetal electrocardiogram from maternal electrocardiogram and classification of normal and abnormal signals	2017	10.1109/ siprocess.2017.8124572	IEEE	fetal	no
ecg signal parameters extraction using intelligent adaptive algorithm	2017	10.1109/ sta.2017.8314972	IEEE	duplicate	no
an energy efficient ecg signal processor detecting cardiovascular diseases on smartphone	2017	10.1109/ tbcas.2016.2592382	IEEE	hardware	no
energy-efficient hardware architecture of self-organizing map for ecg clustering in 65-nm cmos	2017	10.1109/ tcsii.2017.2672789	IEEE	off topic	no
analysis of sampling frequency and resolution in ecg signals	2017	10.1109/ telfor.2017.8249438	IEEE	duplicate	no
real time implementation of arrhythmia classification algorithm using statistical methods	2017	10.1109/ tima.2017.8064824	IEEE	off topic	no
keep the stress away with soda: stress detection and alleviation system	2017	10.1109/ tmscs.2017.2703613	IEEE	off topic	no
qrs complex detection in tele-health ecg recordings using modified unsw algorithm	2017	10.1109/ wispnet.2017.8299951	IEEE	duplicate	no
an efficient wavelet-based feature extraction scheme for electrocardiogram signals	2017	10.1109/ wits.2017.7934670	IEEE	duplicate	no
blinded analysis of an exercise ecg database using high frequency qrs analysis	2017	10.22489/ cinc.2017.010-254	IEEE	off topic	no
detecting ecg limb lead-wire interchanges involving the right leg lead-wire	2017	10.22489/ cinc.2017.014-061	IEEE	off topic	no
clinical performance of high frequency qrs analysis for detecting ischemia using a limited sampling rate	2017	10.22489/ cinc.2017.023-190	IEEE	off topic	no
robust automatic detection of p wave and t wave in electrocardiogram	2017	10.22489/ cinc.2017.089-407	IEEE	off topic	no
atrial fibrillation analysis for real time patient monitoring	2017	10.22489/ cinc.2017.118-272	IEEE	off topic	no
a system for electrocardiographic studies in the community	2017	10.22489/ cinc.2017.127-094	IEEE	off topic	no
atrial fibrillation detection using feature based algorithm and deep convolutional neural network	2017	10.22489/ cinc.2017.159-327	IEEE	off topic	no
cardiac arrhythmia detection from ecg combining convolutional and long short-term memory networks	2017	10.22489/ cinc.2017.161-460	IEEE	off topic	no

robust feature extraction from noisy ecg for atrial fibrillation detection	2017	10.22489/ cinc.2017.164-301	IEEE	off topic	no
arrhythmia classification from the abductive interpretation of short single-lead ecg records	2017	10.22489/ cinc.2017.166-054	IEEE	off topic	no
detection of atrial fibrillation episodes from short single lead recordings by means of ensemble learning	2017	10.22489/ cinc.2017.169-313	IEEE	off topic	no
morphology-based detection of premature ventricular contractions	2017	10.22489/ cinc.2017.211-260	IEEE	off topic	no
a four-lead real time arrhythmia analysis algorithm	2017	10.22489/ cinc.2017.214-182	IEEE	duplicate	no
qrs fragmentation index as a new discriminator for early diagnosis of heart diseases	2017	10.22489/ cinc.2017.267-258	IEEE	duplicate	no
detecting ischemic stress to the myocardium using laplacian eigenmaps and changes to conduction velocity	2017	10.22489/ cinc.2017.269-417	IEEE	off topic	no
progression towards heart failure after myocardial infarction is accompanied by a change in the spatial qrs-t angle	2017	10.22489/ cinc.2017.292-342	IEEE	duplicate	no
classification of atrial fibrillation in short-term ecg recordings using a machine learning approach and hybrid qrs detection	2017	10.22489/ cinc.2017.337-201	IEEE	off topic	no
classification of ecg recordings with neural networks based on specific morphological features and regularity of the signal	2017	10.22489/ cinc.2017.356-350	IEEE	missing accuracy	no
automatic detection of atrial fibrillation and other arrhythmias in holter ecg recordings using rhythm features and neural networks	2017	10.22489/ cinc.2017.364-057	IEEE	off topic	no
a channel-dependent algorithm for heart beats detection in ecg recordings	2017	10.23919/ cisti.2017.7975975	IEEE	duplicate	no
features extraction from impedance cardiography signal	2017	10.23919/ measurement.2017. 7983577	IEEE	off topic	no
hilbert transform based paroxysmal tachycardia detection algorithm	2017	10.23919/ mipro.2017.7973443	IEEE	off topic	no
control and signal processing software embedded in smart wristband monitor of silent atrial fibrillation	2017	10.23919/ mixdes.2017.800528 1	IEEE	off topic	no

ecg parameter extraction and classification in noisy signals	2017	10.23919/ spa.2017.8166872	IEEE	off topic	no
a novel r peak detection method for mobile environments	2018	10.1109/ access.2018.2867329	IEEE	INCLUDED	yes
severity classification of obstructive sleep apnea using only heart rate variability measures with an ensemble classifier	2018	10.1109/ bhi.2018.8333363	IEEE	off topic	no
energy-efficient ecg compression in wearable body sensor network by leveraging empirical mode decomposition	2018	10.1109/ bhi.2018.8333391	IEEE	off topic	no
p-qrs-t localization in ecg using deep learning	2018	10.1109/ bhi.2018.8333406	IEEE	INCLUDED	yes
a tree-search method for single-channel fetal qrs complexes detection in fetal heart rate monitoring	2018	10.1109/ bibm.2018.8621262	IEEE	fetal	no
detecting ecg heartbeat abnormalities using artificial neural networks	2018	10.1109/ bigdata.2018.8622623	IEEE	off topic	no
measuring fine-grained heart-rate using a flexible wearable sensor in the presence of noise	2018	10.1109/ bsn.2018.8329683	IEEE	duplicate	no
detection and parameter estimation of r peaks in ecg signal using optimization algorithm	2018	10.1109/ candarw.2018.00039	IEEE	missing accuracy	no
r-peak based arrhythmia detection using hilbert transform and principal component analysis	2018	10.1109/ cipech.2018.8724191	IEEE	INCLUDED	yes
multi-dynamics analysis of qrs complex for atrial fibrillation diagnosis	2018	10.1109/ codit.2018.8394935	IEEE	duplicate	no
inter-patient ecg classification using deep convolutional neural networks	2018	10.1109/ dsd.2018.00077	IEEE	off topic	no
comparative study on heart rate variability analysis for atrial fibrillation detection in short single-lead ecg recordings	2018	10.1109/ embc.2018.8512345	IEEE	duplicate	no
fully disposable wireless patch sensor for continuous remote patient monitoring	2018	10.1109/ embc.2018.8512569	IEEE	duplicate	no
qrs detection and measurement method of ecg paper based on convolutional neural networks	2018	10.1109/ embc.2018.8513132	IEEE	duplicate	no

detecting cardiac activity by capacitive electrodes from a single point on the wrist	2018	10.1109/ embc.2018.8513339	IEEE	hardware	no
a modified mask for continuous cardiac monitoring during positive airway pressure therapy	2018	10.1109/ embc.2018.8513399	IEEE	off topic	no
r-r interval outlier exclusion method based on statistical ecg values targeting hrv analysis using wearable ecg devices	2018	10.1109/ embc.2018.8513452	IEEE	off topic	no
a fast principal component analysis method for calculating the ecg derived respiration	2018	10.1109/ embc.2018.8513495	IEEE	off topic	no
detection of r peaks and rr intervals in electrocardiogram print-outs using wavelet transforms and hough transforms	2018	10.1109/ hnicem.2018.8666425	IEEE	inaccessible dataset	no
multi lead fetal qrs detection with principal component analysis	2018	10.1109/ i2ct.2018.8529322	IEEE	fetal	no
cardiac arrhythmia detection through ecg signals	2018	10.1109/ i2ct42659.2018.9057836	IEEE	duplicate	no
development of heartbeat measures and arrythmia premonitory based on time sampling	2018	10.1109/ iac.2018.8780429	IEEE	off topic	no
cardiac arrhythmia detection from single-lead ecg using cnn and lstm assisted by oversampling	2018	10.1109/ icacci.2018.8554541	IEEE	off topic	no
iot-enabled heart monitoring device with signal de-noising and segmentation using discrete wavelet transform	2018	10.1109/ icarcv.2018.8581315	IEEE	duplicate	no
innovative r peak detecting algorithm using enhanced artificial bee colony	2018	10.1109/ icasi.2018.8394317	IEEE	does not introduce a new technique	no
a real-time qrs complex detector based on discrete wavelet transform and adaptive threshold as standalone application on arm microcontrollers	2018	10.1109/ icbea.2018.8471741	IEEE	duplicate	no
a low-power current-mode analog qrs-detection circuit for wearable ecg sensors	2018	10.1109/ icbme.2018.8703577	IEEE	hardware	no
locating heartbeats from electrocardiograms and other correlated signals	2018	10.1109/ iccia.2018.00032	IEEE	mutichannel	no
derivative-based peak detection algorithm for ppa waveforms	2018	10.1109/ iccic.2018.8782379	IEEE	duplicate	no

physiological detection of anxiety	2018	10.1109/ iccsdet.2018.882116 2	IEEE	off topic	no
real time ecg acquisition and fpga based qrs detection	2018	10.1109/ iccubea.2018.86977 10	IEEE	duplicate	no
a low complexity algorithm for detection of three types of cardiac arrhythmia	2018	10.1109/ iceeccot43722.2018. 9001502	IEEE	off topic	no
adaptive noise reduction of multichannel signals obtained by capacitive ecg sensors	2018	10.1109/ icfsp.2018.8552062	IEEE	duplicate	no
fetal qrs detection based on convolutional neural networks in noninvasive fetal electrocardiogram	2018	10.1109/ icfsp.2018.8552074	IEEE	duplicate	no
a novel approach in determining the vitals in the ecg, scg and respiratory signals	2018	10.1109/ icict43934.2018.903 4291	IEEE	off topic	no
monitoring and warning system for the elderly: a case study of cardiac arrhythmias	2018	10.1109/ict- ispc.2018.8523932	IEEE	off topic	no
autoencoder neural networks for outlier correction in ecg- based biometric identification	2018	10.1109/idaacs- sws.2018.8525836	IEEE	off topic	no
pc-based detection of ecg signals, decomposition and analysis	2018	10.1109/ idap.2018.8620730	IEEE	off topic	no
arrhythmia classification using dwt-coefficient energy ratios	2018	10.1109/ iecbes.2018.862672 6	IEEE	off topic	no
smart weighing scale with feet-sampled ecg	2018	10.1109/ iecon.2018.8591487	IEEE	off topic	no
an application of zero cross qrs detection algorithm of ecg signals with various subject conditions	2018	10.1109/ incae.2018.8579419	IEEE	duplicate	no
smart ecg holter monitoring system using smartphone	2018	10.1109/ iotais.2018.8600891	IEEE	duplicate	no
the limb leads ecg signal analysis in inferior myocardial infarction patients by rule base	2018	10.1109/isai- nlp.2018.8692998	IEEE	duplicate	no
electrocardiogram fiducial points detection for health care systems	2018	10.1109/ iscc.2018.8538710	IEEE	off topic	no
a wearable device for real-time ecg monitoring and cardiovascular arrhythmia detection for resource constrained regions	2018	10.1109/ ised.2018.8704072	IEEE	off topic	no

a humans' status detection scheme for industrial safety	2018	10.1109/ isie.2018.8433647	IEEE	off topic	no
garma modeling of ecg and classification of arrhythmia	2018	10.1109/ isms.2018.00015	IEEE	off topic	no
an enhanced random forest for cardiac diseases identification based on ecg signal	2018	10.1109/ iwcmc.2018.8450361	IEEE	off topic	no
a modular low-complexity ecg delineation algorithm for real-time embedded systems	2018	10.1109/ jbhi.2017.2671443	IEEE	off topic	no
automated ecg noise detection and classification system for unsupervised healthcare monitoring	2018	10.1109/ jbhi.2017.2686436	IEEE	off topic	no
a real-time qrs detection method based on phase portraits and box-scoring calculation	2018	10.1109/ jsen.2018.2812792	IEEE	duplicate	no
design and characterization of screen-printed textile electrodes for ecg monitoring	2018	10.1109/ jsen.2018.2819202	IEEE	hardware	no
qrs complex detection and measurement algorithms for multichannel ecgs in cardiac resynchronization therapy patients	2018	10.1109/ jtehm.2018.2844195	IEEE	duplicate	no
new adaptive thresholding-based ecg r-peak detection technique	2018	10.1109/ mecbme.2018.8402423	IEEE	does not introduce a new technique	yes
matlab based gui for ecg arrhythmia detection using pan-tompkin algorithm	2018	10.1109/ pdgc.2018.8745865	IEEE	duplicate	no
fast detection of p, q, s and t waves from normal ecg signals using local context windows	2018	10.1109/ rcar.2018.8621824	IEEE	duplicate	no
a novel framework for prediction and detection of myocardial ischemia with single lead ecg and pat	2018	10.1109/ rice.2018.8509036	IEEE	off topic	no
i-nxgevita: iomt based ubiquitous health monitoring system using deep neural networks	2018	10.1109/ rteict42901.2018.9012640	IEEE	off topic	no
partition of medical signals with a two band adaptive filter bank	2018	10.1109/ siu.2018.8404160	IEEE	duplicate	no
automatic identification of abnormalities in 12-lead ecgs using expert features and convolutional neural networks	2018	10.1109/ snsp.2018.00038	IEEE	mutichannel	no
parameter extraction of ecg using labview	2018	10.1109/ ssd.2018.8570391	IEEE	duplicate	no

a real-time qrs detection system with pr/rt interval and st segment measurements for wearable ecg sensors using parallel delta modulators	2018	10.1109/ tbcas.2018.2823275	IEEE	duplicate	no
robust methods for automated selection of cardiac signals after blind source separation	2018	10.1109/ tbme.2017.2788701	IEEE	off topic	no
a personalized point-of-care platform for real-time ecg monitoring	2018	10.1109/ tce.2018.2877481	IEEE	off topic	no
embedded emotion recognition system based on electrocardiogram attributes	2018	10.1109/ tsp.2018.8441234	IEEE	off topic	no
spiral complex movements of the heart wall at the beginning of myocardial contraction detected by high frame speckle tracking	2018	10.1109/ ultsym.2018.857998 8	IEEE	off topic	no
detection of cardiac events in echocardiography using 3d convolutional recurrent neural networks	2018	10.1109/ ultsym.2018.858013 7	IEEE	off topic	no
heartbeat classification in wearables using multi-layer perceptron and time-frequency joint distribution of ecg	2018	10.1145/3278576.32 78598	IEEE	off topic	no
detecting “strict” left bundle branch block from 12-lead electrocardiogram using support vector machine classification and derivative analysis	2018	10.22489/ cinc.2018.030	IEEE	duplicate	no
a multilead fusion based qrs complex detection method on 12-lead electrocardiogram signals	2018	10.22489/ cinc.2018.082	IEEE	mutichannel	no
p-wave analysis in atrial fibrillation detection using a neural network clustering algorithm	2018	10.22489/ cinc.2018.087	IEEE	off topic	no
serial ecg analysis: absolute rather than signed changes in the spatial qrs-t angle should be used to detect emerging cardiac pathology	2018	10.22489/ cinc.2018.099	IEEE	duplicate	no
a method for removing pacing artifacts from ultra-high-frequency electrocardiograms	2018	10.22489/ cinc.2018.106	IEEE	denoising	no
risk assessment of all-cause mortality in icd patients using a novel qrs fragmentation score	2018	10.22489/ cinc.2018.115	IEEE	off topic	no
r-peak detection in holter ecg signals using non-negative matrix factorization	2018	10.22489/ cinc.2018.123	IEEE	duplicate	no
a wavelet-based approach for automatic diagnosis of strict left bundle branch block	2018	10.22489/ cinc.2018.154	IEEE	off topic	no
cardiac fibrosis detection applying machine learning techniques to standard 12-lead ecg	2018	10.22489/ cinc.2018.174	IEEE	mutichannel	no

a robust detection method of atrial fibrillation	2018	10.22489/ cinc.2018.268	IEEE	off topic	no
detecting flutter waves in the electrocardiogram using generalized likelihood ratio test	2018	10.22489/ cinc.2018.375	IEEE	off topic	no
removal of movement artefact for mobile eeg analysis in sports exercises	2019	10.1109/ access.2018.289033 5	IEEE	denoising	no
verb: vfcdm-based electrocardiogram reconstruction and beat detection algorithm	2019	10.1109/ access.2019.289409 2	IEEE	duplicate	no
detection of fetal ecg r wave from single-lead abdominal ecg using a combination of rr time-series smoothing and template-matching approach	2019	10.1109/ access.2019.291782 6	IEEE	duplicate	no
a novel deep arrhythmia-diagnosis network for atrial fibrillation classification using electrocardiogram signals	2019	10.1109/ access.2019.291879 2	IEEE	duplicate	no
an automated strategy for early risk identification of sudden cardiac death by using machine learning approach on measurable arrhythmic risk markers	2019	10.1109/ access.2019.292584 7	IEEE	off topic	no
noise detection in electrocardiogram signals for intensive care unit patients	2019	10.1109/ access.2019.292619 9	IEEE	duplicate	no
impact of ecg dataset diversity on generalization of cnn model for detecting qrs complex	2019	10.1109/ access.2019.292772 6	IEEE	off topic	no
preprocessing method for performance enhancement in cnn-based stemi detection from 12-lead ecg	2019	10.1109/ access.2019.293077 0	IEEE	mutichannel	no
interpretability analysis of heartbeat classification based on heartbeat activity's global sequence features and bilstm-attention neural network	2019	10.1109/ access.2019.293347 3	IEEE	off topic	no
an accurate qrs complex and p wave detection in ecg signals using complete ensemble empirical mode decomposition with adaptive noise approach	2019	10.1109/ access.2019.293994 3	IEEE	duplicate	no
a new automatic identification method of heart failure using improved support vector machine based on duality optimization technique	2019	10.1109/ access.2019.294552 7	IEEE	off topic	no

inter-patient cnn-lstm for qrs complex detection in noisy ecg signals	2019	10.1109/ access.2019.2955738	IEEE	duplicate	no
a novel method to detect multiple arrhythmias based on time-frequency analysis and convolutional neural networks	2019	10.1109/ access.2019.2956050	IEEE	off topic	no
portable real time ecg monitor and disease diagnostics	2019	10.1109/ becithcon48839.2019.9063172	IEEE	duplicate	no
simultaneous multiple features tracking of beats: a representation learning approach to reduce false alarm rate in icus	2019	10.1109/ bibm47256.2019.8983408	IEEE	off topic	no
a high-precise arrhythmia detection method based on biorthogonal wavelet and fully connected neural network	2019	10.1109/ bigdataservice.2019.00056	IEEE	off topic	no
an ecg processor for the detection of eight cardiac arrhythmias with minimum false alarms	2019	10.1109/ biocas.2019.8919053	IEEE	off topic	no
cuff-less methods for blood pressure measurement	2019	10.1109/ cac48633.2019.8997340	IEEE	off topic	no
low-energy ecg processing for accurate features' extraction in wireless body sensor networks	2019	10.1109/ cais.2019.8769544	IEEE	off topic	no
poster abstract: automated detection of the onset of ventricular depolarization in challenging clinical ecg data	2019	10.1109/ chase48038.2019.00010	IEEE	off topic	no
wearable ecg for real time complex p-qrs-t detection and classification of various arrhythmias	2019	10.1109/ comsnets.2019.8711218	IEEE	duplicate	no
ecg signal analysis using 2-d image classification with convolutional neural network	2019	10.1109/ csci49370.2019.00180	IEEE	off topic	no
basic study to reduce the artifact in brain activity measuring data by using auto-regressive model	2019	10.1109/ csci49370.2019.00194	IEEE	off topic	no
automatic qrs onset detection of ecg signal using secant line slope formula	2019	10.1109/ cspa.2019.8695982	IEEE	duplicate	no

wavelet-based signal quality assessment: noise detection by temporal feature and heuristics-based	2019	10.1109/ cyberneticscom.2019.8875655	IEEE	off topic	no
development of arrhythmia classification system for personal cardiac monitor in thailand	2019	10.1109/ eltech.2019.8839615	IEEE	duplicate	no
comparing different methods of hand-crafted hrv, edr and cpc features for sleep apnoea detection	2019	10.1109/ embc.2019.8856779	IEEE	duplicate	no
a deep learning method to detect atrial fibrillation based on continuous wavelet transform	2019	10.1109/ embc.2019.8856834	IEEE	off topic	no
a novel detection method of bundle branch block from multi-lead ecg	2019	10.1109/ embc.2019.8857485	IEEE	mutichannel	no
sleep arousal and sudden changes in cardiac qt interval	2019	10.1109/ embc.2019.8857651	IEEE	off topic	no
is there an optimal localization of cardio-microphone sensors for phonocardiogram analysis?	2019	10.1109/ embc.2019.8857681	IEEE	off topic	no
ejection wave segmentation for contact-free heart rate estimation from ballistocardiographic signals	2019	10.1109/ embc.2019.8857731	IEEE	off topic	no
convolutional recurrent neural networks to characterize the circulation component in the thoracic impedance during out-of-hospital cardiac arrest	2019	10.1109/ embc.2019.8857758	IEEE	off topic	no
portable system for real time detection of p, qrs and t waves from ecg signals	2019	10.1109/ i2ct45611.2019.9033666	IEEE	duplicate	no
harmonic decomposition of ecg signal using analytic signal matched filter bank	2019	10.1109/ icaccp.2019.8882983	IEEE	off topic	no
envisaging ventricular arrhythmia from an ecg by using machine learning algorithm	2019	10.1109/ icaccs.2019.8728525	IEEE	duplicate	no
a survey of algorithms for feature extraction and feature classification methods	2019	10.1109/ icactm.2019.8776804	IEEE	off topic	no
efficient communication overhead reduction using polygonal approximation-based ecg signal compression	2019	10.1109/ icaiic.2019.8668974	IEEE	off topic	no
comparative analysis of machine learning algorithms along with classifiers for af detection using a scale	2019	10.1109/ icaiic.2019.8669084	IEEE	off topic	no

improvement of ecg based personal identification performance in different bathtub water temperature by cnn	2019	10.1109/ icawst.2019.8923503	IEEE	off topic	no
ecg automatic identification method based on bp neural	2019	10.1109/ ICCCE48422.2019.9010793	IEEE	off topic	no
a modern cardiac monitoring system based on wireless sensing for human health care	2019	10.1109/ icces45898.2019.9002563	IEEE	off topic	no
ecg signal processing using classifier to analyses cardiovascular disease	2019	10.1109/ iccmc.2019.8819777	IEEE	off topic	no
ecg signals denoising and features extraction by applying ufir smoothing with optimal q-lag in the state space	2019	10.1109/ iceee.2019.8884556	IEEE	off topic	no
ecg arrhythmia classification based on fuzzy cognitive maps	2019	10.1109/ iceee.2019.8884560	IEEE	off topic	no
vhdl module for the r wave detection in real time using continuous wavelet transform	2019	10.1109/ iceee.2019.8884584	IEEE	hardware	no
analysis of different heart rate monitoring and pre-processing techniques for ecg	2019	10.1109/ icnte44896.2019.8946042	IEEE	duplicate	no
detection of heart rate through speech using mel frequency cepstrum coefficients	2019	10.1109/ icoei.2019.8862611	IEEE	off topic	no
detection of waves in ecg for arrhythmia classification	2019	10.1109/ icoei.2019.8862653	IEEE	off topic	no
classification of ecg signal using machine learning techniques	2019	10.1109/ icpedc47771.2019.9036613	IEEE	does not introduce a new technique	no
a comprehensive review on accurate qrs and t wave detection techniques for confirming cardiac abnormalities	2019	10.1109/ icraecc43874.2019.8995091	IEEE	review	no
design of a biosignal based stress detection system using machine learning techniques	2019	10.1109/ icrest.2019.8644259	IEEE	off topic	no
extraction of p and t waves from electrocardiogram signals with modified hamilton algorithm	2019	10.1109/ icsecc.2019.8907016	IEEE	duplicate	no

the influence of parameter selection for rényi phase permutation entropy on abnormal change detection	2019	10.1109/icsidp47821.2019.9172821	IEEE	off topic	no
classification of short-time single-lead ecg recordings using deep residual cnn	2019	10.1109/ictcs.2019.8923079	IEEE	inaccessible dataset	no
fetal arrhythmia detection using fetal ecg signal	2019	10.1109/ictp48844.2019.9041789	IEEE	fetal	no
arrhythmia classification system using deep neural network	2019	10.1109/icufn.2019.8805913	IEEE	off topic	no
an ecg-ppg wearable device for real time detection of various arrhythmic cardiovascular diseases	2019	10.1109/ised48680.2019.9096223	IEEE	off topic	no
an internet of things (iot) management system for improving homecare - a case study	2019	10.1109/isncc.2019.8909186	IEEE	off topic	no
design of cardiac status indicator and r-r interval adjustment circuits	2019	10.1109/isocc47750.2019.9027742	IEEE	off topic	no
ecg signal processing and analysis for accurate features extraction	2019	10.1109/isspit47144.2019.9001872	IEEE	off topic	no
design and implementation of a novel r-peak detection algorithm	2019	10.1109/itme.2019.00035	IEEE	INCLUDED	yes
fast qrs detection and ecg compression based on signal structural analysis	2019	10.1109/jbhi.2018.2792404	IEEE	duplicate	no
noncontact in-bed measurements of physiological and behavioral signals using an integrated fabric-sheet sensing scheme	2019	10.1109/jbhi.2018.2825020	IEEE	off topic	no
towards end-to-end ecg classification with raw signal extraction and deep neural networks	2019	10.1109/jbhi.2018.2871510	IEEE	duplicate	no
a machine-learning approach for detection and quantification of qrs fragmentation	2019	10.1109/jbhi.2018.2878492	IEEE	duplicate	no
bayesian real-time qrs complex detector for healthcare system	2019	10.1109/jiot.2019.2903530	IEEE	duplicate	no
lossless and lossy direct compression design with multi-signal symptom detection for low-temperature wearable devices	2019	10.1109/jsen.2018.2877430	IEEE	mutichannel	no

embedded solution for atrial fibrillation detection using smart wireless body sensors	2019	10.1109/ jsen.2019.2906238	IEEE	off topic	no
high precision digitization of paper-based ecg records: a step toward machine learning	2019	10.1109/ jtehm.2019.2949784	IEEE	off topic	no
securing health-related data transmission using ecg and named data networks	2019	10.1109/ lanman.2019.88469 93	IEEE	off topic	no
usefulness of adaptive correlation filter for detecting qrs waves from noisy electrocardiograms	2019	10.1109/ lifetech.2019.888399 3	IEEE	denoising	no
ecgdeepnet: a deep learning approach for classifying ecg beats	2019	10.1109/ ritapp.2019.8932850	IEEE	off topic	no
simultaneously concentrated pswf-based synchrosqueezing s-transform and its application to r peak detection in ecg signal	2019	10.1109/ro-man46459.2019.895 6391	IEEE	INCLUDED	yes
versatile detector of pseudo-periodic patterns	2019	10.1109/ rpic.2019.8882132	IEEE	off topic	no
algorithmic and software analysis and processing of ecg signals	2019	10.1109/ sibircon48586.2019. 8958424	IEEE	inaccessible dataset	yes
an efficient and robust digital fractional order differentiator based ecg pre-processor design for qrs detection	2019	10.1109/ tbcas.2019.2916676	IEEE	duplicate	no
nfc-powered flexible chest patch for fast assessment of cardiac, hemodynamic, and endocrine parameters	2019	10.1109/ tbcas.2019.2956810	IEEE	off topic	no
robust heartbeat detection from multimodal data via cnn-based generalizable information fusion	2019	10.1109/ tbme.2018.2854899	IEEE	mutichannel	no
catheter treatment of ventricular tachycardia: a reference-less pace-mapping method to identify ablation targets	2019	10.1109/ tbme.2019.2903631	IEEE	off topic	no
robust fetal heart beat detection via r-peak intervals distribution	2019	10.1109/ tbme.2019.2904014	IEEE	fetal	no
radio-frequency passive identifier for biomedical applications	2019	10.1109/ telsiks46999.2019.9 002012	IEEE	off topic	no
automatic detection of cardiac arrhythmias using ensemble learning	2019	10.1109/ tencon.2019.892934 8	IEEE	off topic	no

automated noise detection and classification for unsupervised ecg analysis systems using ceemd and wavelet packet decomposition	2019	10.1109/ tencon.2019.892945 0	IEEE	off topic	no
a first derivative based r-peak detection and dwt based beat delineation approach of single lead electrocardiogram signal	2019	10.1109/ tensymp46218.2019. 8971094	IEEE	INCLUDED	yes
improved arrhythmia detection from electrocardiogram	2019	10.1109/ tensymp46218.2019. 8971240	IEEE	off topic	no
1d convolutional neural network for detecting ventricular heartbeats	2019	10.1109/ tla.2019.9011541	IEEE	off topic	no
fiducial ecg-based biometry: comparison of classifiers and dimensionality reduction methods	2019	10.1109/ tsp.2019.8768891	IEEE	off topic	no
speeds of contraction responses propagating along septum at pre-ejection period are different between radial and longitudinal directions	2019	10.1109/ ultsym.2019.892599 0	IEEE	off topic	no
development of methods and algorithms for determining physiological parameters of the patient with the help of the biomodule	2019	10.1109/ usbereit.2019.87365 66	IEEE	off topic	no
processing of synchronous recordings of surface ecg and intracardiac potentials for diagnostics of dangerous heart rate disturbances	2019	10.1109/ usbereit.2019.87366 73	IEEE	off topic	no
a body area network for ubiquitous driver stress monitoring based on ecg signal	2019	10.1109/ wimob.2019.892346 8	IEEE	off topic	no
heart rate variability monitoring using a wearable armband	2019	10.22489/ cinc.2019.059	IEEE	off topic	no
detection of first-degree atrioventricular block on variable-length electrocardiogram via a multimodal deep learning method	2019	10.22489/ cinc.2019.194	IEEE	off topic	no
a highly-reliable full-automatic system for analyzing ecg waveforms in real time applications	2019	10.22489/ cinc.2019.224	IEEE	missing technical details	no
an efficient instantaneous ecg delineation algorithm	2019	10.22489/ cinc.2019.227	IEEE	off topic	no
a single ecg lead-based oscillation index for the quantification of periodic breathing in severe heart failure patients	2019	10.23919/ eusipco.2019.89027 14	IEEE	off topic	no

application of svd for removing motion artifacts from the measurements of a wireless electrocardiogram	2019	10.23919/fusion43075.2019.9011419	IEEE	denoising	no
a 17.7-pj/cycle ecg processor for arrhythmia detection with high immunity to power line interference and baseline drift	2020	10.1109/a-sscc48613.2020.9336104	IEEE	hardware	no
a comparison between novel fpga-based pad monitoring system using ballistocardiography and the conventional systems for synchronization and gating of cmri at 3 tesla: a pilot study	2020	10.1109/access.2019.2963221	IEEE	hardware	no
a crucial wave detection and delineation method for twelve-lead ecg signals	2020	10.1109/access.2020.2965334	IEEE	duplicate	no
robust qrs detection using high-resolution wavelet packet decomposition and time-attention convolutional neural network	2020	10.1109/access.2020.2967775	IEEE	duplicate	no
ensemble networks for user recognition in various situations based on electrocardiogram	2020	10.1109/access.2020.2975258	IEEE	off topic	no
arrhythmia recognition and classification using combined parametric and visual pattern features of ecg morphology	2020	10.1109/access.2020.2979256	IEEE	duplicate	no
health monitoring of human multiple physiological parameters based on wireless remote medical system	2020	10.1109/access.2020.2987058	IEEE	duplicate	no
qrs complex detection using novel deep learning neural networks	2020	10.1109/access.2020.2997473	IEEE	duplicate	no
detecting noisy ecg qrs complexes using waveletcnn autoencoder and convlstm	2020	10.1109/access.2020.3012904	IEEE	duplicate	no
stages-based ecg signal analysis from traditional signal processing to machine learning approaches: a survey	2020	10.1109/access.2020.3026968	IEEE	duplicate	no
the cardiodynamicsgram based early detection of myocardial ischemia using the lempel-ziv complexity	2020	10.1109/access.2020.3038210	IEEE	off topic	no

evaluating deep learning algorithms for real-time arrhythmia detection	2020	10.1109/ bdcat50828.2020.00 022	IEEE	off topic	no
improving ecg classification interpretability using saliency maps	2020	10.1109/ bibe50027.2020.001 14	IEEE	off topic	no
ecg data mining using sas	2020	10.1109/ cbi49978.2020.1006 1	IEEE	off topic	no
optimized pan-tompkins based heartbeat detection algorithms	2020	10.1109/ ccdc49329.2020.916 4736	IEEE	does not introduce a new technique	yes
automatic qrs detection and segmentation using short time fourier transform and feature fusion	2020	10.1109/ ccece47787.2020.92 55676	IEEE	mutichannel	no
r peak detection in ecg signals using chebfun	2020	10.1109/ ccem50674.2020.00 023	IEEE	missing technical details	no
comparison of 10 qrs detection methods for heart beat detection on portable ecg systems	2020	10.1109/ cenim51130.2020.92 97903	IEEE	duplicate	no
arrhythmia classification on electrocardiogram signal using convolution neural network based on frequency spectrum	2020	10.1109/ cenim51130.2020.92 97997	IEEE	off topic	no
a 151nw second-order ternary delta modulator for ecg slope variation measurement with baseline wandering resilience	2020	10.1109/ cicc48029.2020.907 5953	IEEE	hardware	no
detection of af and i-avb combined with rr interval and p wave	2020	10.1109/cisp-bmei51763.2020.92 63514	IEEE	off topic	no
an automatic detection of arrhythmia disease diagnosis system based on artificial neural network and support vector machine	2020	10.1109/ compe49325.2020.9 200140	IEEE	duplicate	no
characterization of a low cost, automated and field deployable 2-lead myocardial infarction detection system	2020	10.1109/ comsnets48256.202 0.9027451	IEEE	mutichannel	no

portable ecg device for remote monitoring and detection of onset of arrhythmia	2020	10.1109/conecct50063.2020.9198658	IEEE	off topic	no
fold electrocardiogram into a fingerprint	2020	10.1109/cvprw50498.2020.00422	IEEE	off topic	no
machine learning based decision support system for atrial fibrillation detection using electrocardiogram	2020	10.1109/discover50404.2020.9278124	IEEE	duplicate	no
ecg signal processing for early detection of atrial and ventricular fibrillation based on r-r interval	2020	10.1109/eeccis49483.2020.9263454	IEEE	off topic	no
classification of aortic stenosis using ecg by deep learning and its analysis using grad-cam	2020	10.1109/embc44109.2020.9175151	IEEE	off topic	no
preliminary results on density poincare plot based atrial fibrillation detection from premature atrial/ventricular contractions	2020	10.1109/embc44109.2020.9175216	IEEE	off topic	no
transfer learning for detection of atrial fibrillation in deterministic compressive sensed ecg	2020	10.1109/embc44109.2020.9175813	IEEE	off topic	no
an innovative hybrid approach for detection of pacemaker pulses at low sampling frequency	2020	10.1109/embc44109.2020.9176390	IEEE	off topic	no
development of cloud computing algorithm for arrhythmia detection	2020	10.1109/fortei-icee50915.2020.9249808	IEEE	off topic	no
multi-stage detection of atrial fibrillation in compressively sensed electrocardiogram	2020	10.1109/i2mtc43012.2020.9128396	IEEE	off topic	no
p wave detection in electrocardiogram based on wavelet transform and differential correction	2020	10.1109/icaci49185.2020.9177647	IEEE	off topic	no
noise reduction method based on autocorrelation for threshold-based heartbeat detection	2020	10.1109/icamechs49982.2020.9310147	IEEE	denoising	no

extracting fetal heart rate from abdominal ecgs based on fast multivariate empirical mode decomposition	2020	10.1109/icarcv50220.2020.9305481	IEEE	fetal	no
fractional fourier transform based qrs complex detection in ecg signal	2020	10.1109/icassp40776.2020.9052939	IEEE	off topic	no
retracted: deep neural network models for detection of arrhythmia based on electrocardiogram reports	2020	10.1109/icccnt49239.2020.9225534	IEEE	off topic	no
development of algorithm for extraction of fetal from maternal ecg on benchmark database and prototype development for acquisition	2020	10.1109/iccmc48092.2020.iccmc-00059	IEEE	fetal	no
a decision tree optimised svm model for stress detection using biosignals	2020	10.1109/iccsp48568.2020.9182043	IEEE	off topic	no
r - peak detection using altered pan-tompkins algorithm	2020	10.1109/iccsp48568.2020.9182298	IEEE	duplicate	no
identifying and remove the mislabeled training sample of ecg signals using ensemble learning and iterative	2020	10.1109/iccss52145.2020.9336904	IEEE	off topic	no
detection of myocardial infarction from ecg signal through combining cnn and bi-lstm	2020	10.1109/icece51571.2020.9393090	IEEE	off topic	no
detection of a fib and its classification using svm	2020	10.1109/icimia48430.2020.9074888	IEEE	off topic	no
methodology for processing and analysis of diagnostic indicators electrocardiogram based on labview	2020	10.1109/icisct50599.2020.9351507	IEEE	off topic	no
a novel non invasive myocardial infarction vigilant system by using raspberry pi	2020	10.1109/iciss49785.2020.9315917	IEEE	off topic	no
prediction of coronary artery disease using electrocardiography: a machine learning approach	2020	10.1109/icmlc51923.2020.9469585	IEEE	off topic	no

premature ventricular contractions classification using machine learning approach	2020	10.1109/icosec49089.2020.9215290	IEEE	off topic	no
an octave convolution neural network-based qrs detector	2020	10.1109/icsmd50554.2020.9261658	IEEE	duplicate	no
diagnosis of fetal arrhythmia using jade algorithm	2020	10.1109/icsss49621.2020.9202124	IEEE	fetal	no
classification of p-wave morphology using new local distance transform and random forests	2020	10.1109/icst50505.2020.9732811	IEEE	off topic	no
a new methodology for classifying qrs morphology in ecg signals	2020	10.1109/ijcnn48605.2020.9206707	IEEE	duplicate	no
novel method for detection of electrocardiogram r waves using amplitude modulation	2020	10.1109/incet49848.2020.9154052	IEEE	missing technical details	yes
improved algorithm for spectrum-based r wave detection	2020	10.1109/inocon50539.2020.9298444	IEEE	missing technical details	no
mri-based characterization of left ventricle dyssynchrony with correlation to crt outcomes	2020	10.1109/isbi45749.2020.9098519	IEEE	off topic	no
asic implementation of a pre-trained neural network for ecg feature extraction	2020	10.1109/iscas45731.2020.9180703	IEEE	off topic	no
qvat: qrs complex detection based on variance analysis and adaptive threshold for electrocardiogram signal	2020	10.1109/isitia49792.2020.9163784	IEEE	duplicate	no
single channel qrs detection using wavelet and median denoising with adaptive multilevel thresholding	2020	10.1109/isspit51521.2020.9408699	IEEE	INCLUDED	yes
design and implementation of a novel real time p-qrs-t waves detection algorithm	2020	10.1109/itnec48623.2020.9084924	IEEE	duplicate	no

heart arrhythmia detection & monitoring using machine learning & ecg wearable device	2020	10.1109/ itt51279.2020.9320881	IEEE	off topic	no
a new wearable ecg monitor evaluation and experimental analysis: proof of concept	2020	10.1109/ iwcmc48107.2020.9148191	IEEE	off topic	no
separation of fetal-ecg from single-channel abdominal ecg using activation scaled non-negative matrix factorization	2020	10.1109/ jbhi.2019.2920356	IEEE	fetal	no
an automatic r and t peak detection method based on the combination of hierarchical clustering and discrete wavelet transform	2020	10.1109/ jbhi.2020.2973982	IEEE	INCLUDED	yes
influence of capacitive coupling on high-fidelity non-contact ecg measurement	2020	10.1109/ jsen.2020.2986723	IEEE	off topic	no
a batteryless motion-adaptive heartbeat detection system-on-chip powered by human body heat	2020	10.1109/ jssc.2020.3013789	IEEE	hardware	no
deterministic compressed domain analysis of multi-channel ecg measurements	2020	10.1109/ memea49120.2020.9137252	IEEE	mutichannel	no
a hybrid deep model for automatic arrhythmia classification based on lstm recurrent networks	2020	10.1109/ memea49120.2020.9137328	IEEE	off topic	no
a fusion based classification of normal, arrhythmia and congestive heart failure in ecg	2020	10.1109/ ncc48643.2020.9056095	IEEE	off topic	no
towards a real-time cognitive load assessment system for industrial human-robot cooperation	2020	10.1109/ro-man47096.2020.9223531	IEEE	off topic	no
a dry electrode-based ecg sensor with motion artifacts cancellation and signal analysis for heart irregularity detection	2020	10.1109/ sensors47125.2020.9278739	IEEE	off topic	no
design of touch ecg detection system based on stm32 and android mobile phone	2020	10.1109/ siitme50350.2020.9292151	IEEE	hardware	no
application of machine learning on ecg signal classification using morphological features	2020	10.1109/ tensymp50017.2020.9230780	IEEE	off topic	yes

fully automated annotation of seismocardiogram for noninvasive vital sign measurements	2020	10.1109/ tim.2019.2908511	IEEE	off topic	no
portable ecg monitoring device design based on arduino	2020	10.1109/ tiptekno50054.2020. 9299238	IEEE	hardware	no
doppler cardiogram: a remote detection of human heart activities	2020	10.1109/ tmtt.2019.2948844	IEEE	duplicate	no
an improved iterative pace-mapping algorithm to detect the origin of premature ventricular contractions	2020	10.22489/ cinc.2020.062	IEEE	duplicate	no
automated identification of paced beats in holter ecg	2020	10.22489/ cinc.2020.067	IEEE	off topic	no
multi-class classification of pathologies found on short ecg signals	2020	10.22489/ cinc.2020.071	IEEE	off topic	no
the electro-anatomical pathway for normal and abnormal ecgs in covid patients	2020	10.22489/ cinc.2020.096	IEEE	duplicate	no
moving dipole determination from 12-lead ecgs can improve detection of acute myocardial ischemia	2020	10.22489/ cinc.2020.115	IEEE	duplicate	no
arrhythmia detection based on patient-specific normal ecgs using deep learning	2020	10.22489/ cinc.2020.137	IEEE	off topic	no
cardiac pathologies detection and classification in 12-lead ecg	2020	10.22489/ cinc.2020.171	IEEE	mutichannel	no
an ecg-based system for respiratory rate estimation tested on a wearable armband during daily life	2020	10.22489/ cinc.2020.251	IEEE	off topic	no
building normal ecg models to detect any arrhythmias using deep learning	2020	10.22489/ cinc.2020.344	IEEE	off topic	no
interpretability analysis of machine learning algorithms in the detection of st-elevation myocardial infarction	2020	10.22489/ cinc.2020.403	IEEE	duplicate	no
ecg morphological decomposition for automatic rhythm identification	2020	10.22489/ cinc.2020.435	IEEE	mutichannel	no
denoise modelling of ecg signals for analog front end	2020	10.23919/ indiacom49435.2020 .9083724	IEEE	off topic	no
detection of uninterpretable ecg signal segments	2020	10.23919/ mipro48935.2020.92 45427	IEEE	duplicate	no

a survey on machine learning approaches to ecg processing	2020	10.23919/ spa50552.2020.924 1283	IEEE	off topic	no
a novel method of qrs detection using time and amplitude thresholds with statistical false peak elimination	2021	10.1109/ access.2021.306717 9	IEEE	INCLUDED	yes
a novel approach for heart ventricular and atrial abnormalities detection via an ensemble classification algorithm based on ecg morphological features	2021	10.1109/ access.2021.307127 3	IEEE	off topic	no
efficient template cluster generation for real-time abnormal beat detection in lightweight embedded ecg acquisition devices	2021	10.1109/ access.2021.307762 8	IEEE	off topic	no
a novel machine learning approach to classify and detect atrial fibrillation using optimized implantable electrocardiogram sensor	2021	10.1109/ access.2021.312336 7	IEEE	duplicate	no
an iot enabled health monitoring kit using non-invasive health parameters	2021	10.1109/ acmi53878.2021.952 8227	IEEE	off topic	no
remote monitoring of heart rate and ecg signal using esp32	2021	10.1109/ aemcse51986.2021. 00127	IEEE	hardware	no
an ecg automatic detection system with baseline drift removal based on sg filter	2021	10.1109/ asicon52560.2021.9 620414	IEEE	missing assessment information	yes
tr-index: semantic characterization for non-invasive fetal ecg signal quality assessment	2021	10.1109/ bibm52615.2021.96 69718	IEEE	fetal	no
assessment of potential primary and recurrent ischemic stroke by detecting atrial fibrillation using 1d-cnn and cha2ds2-va score	2021	10.1109/ bibm52615.2021.96 69885	IEEE	off topic	no
iot and big data for ecg signal classification - a quick decision system	2021	10.1109/ c2i454156.2021.968 9420	IEEE	off topic	no
spatio temporal filtering of multi-lead ecg signals for atrial arrhythmia classification	2021	10.1109/ci- ibbi54220.2021.9626 098	IEEE	mutichannel	no

a low cost and portable electrocardiogram (ecg) for predicting arrhythmia	2021	10.1109/compe53109.2021.9751727	IEEE	off topic	no
identification of arrhythmia using ecg signal patterns	2021	10.1109/compe53109.2021.9752348	IEEE	off topic	no
detection and identification of irregularities in human heart rate	2021	10.1109/conit51480.2021.9498296	IEEE	off topic	no
matlab based ecg signal analysis	2021	10.1109/conit51480.2021.9498423	IEEE	off topic	no
preprocessing of electrocardiogram techniques – a review	2021	10.1109/ehb52898.2021.9657732	IEEE	review	no
approxbiowear: approximating additions for efficient biomedical wearable computing at the edge	2021	10.1109/embc46164.2021.9630165	IEEE	duplicate	no
fetal heart rate detection using first derivative of ecg waveform and multiple weighting functions	2021	10.1109/embc46164.2021.9630268	IEEE	fetal	no
multistage pruning of cnn based ecg classifiers for edge devices	2021	10.1109/embc46164.2021.9630588	IEEE	off topic	no
ecg dry-electrode 3d printing and signal quality considerations	2021	10.1109/embc46164.2021.9630599	IEEE	duplicate	no
ecg-based biometric recognition without qrs segmentation: a deep learning-based approach	2021	10.1109/embc46164.2021.9630899	IEEE	duplicate	no
simulating cardiac disorders with a lumped parameter synergistic model	2021	10.1109/embc46164.2021.9631073	IEEE	off topic	no
detection of atrial fibrillation in electrocardiogram signals using machine learning	2021	10.1109/gcat52182.2021.9587664	IEEE	off topic	no

ecg processing algorithm in the qrs complex	2021	10.1109/icaacca51523.2021.9465280	IEEE	duplicate	no
revisiting derivative based methods on qrs detections from an ecg signal	2021	10.1109/icaeca52838.2021.9675580	IEEE	duplicate	no
ecg signal noises versus filters for signal quality improvement	2021	10.1109/icaect49130.2021.9392621	IEEE	off topic	no
a wireless and wearable system for fetal heart rate monitoring	2021	10.1109/icaml54311.2021.00091	IEEE	fetal	no
enhancing driver drowsiness detection for data acquisition stage using electrocardiogram	2021	10.1109/iccce50029.2021.9467209	IEEE	off topic	no
implementation of a novel intelligent drowsiness detection warning system	2021	10.1109/icce-tw52618.2021.9602872	IEEE	off topic	no
ecg classification and arrhythmia detection using wavelet transform and convolutional neural network	2021	10.1109/iccisc52257.2021.9485012	IEEE	off topic	no
performance analysis of various machine learning techniques for classifying cardiac arrhythmia - survey	2021	10.1109/iceca52323.2021.9675872	IEEE	off topic	no
r wave localization from transformed electrocardiogram signal by emd	2021	10.1109/icecet52533.2021.9698708	IEEE	INCLUDED	yes
computer aided atrial fibrillation detection from the statistical attributes of ecg signal	2021	10.1109/icecit54077.2021.9641198	IEEE	duplicate	no
diagnosis of left ventricular hypertrophy from ecg signals based on ccs methodology using svm	2021	10.1109/iceeict53905.2021.9667877	IEEE	off topic	no
an ecg based arrhythmia detector with an emergency alert system	2021	10.1109/icesc51422.2021.9532690	IEEE	off topic	no

ecg diagnosis device based on machine learning	2021	10.1109/icesit53460.2021.9697057	IEEE	hardware	no
design of ecg signal detection circuit based on pacing pulse suppression	2021	10.1109/icetci53161.2021.9563387	IEEE	hardware	no
p-wave detection using a parallel convolutional neural network in electrocardiogram	2021	10.1109/icicsp54369.2021.9611986	IEEE	off topic	no
filtering the ecg signal towards heart attack detection using motion artifact removal technique	2021	10.1109/icicv50876.2021.9388515	IEEE	off topic	no
ecg arrhythmia classification using 1d cnn leveraging the resampling technique and gaussian mixture model	2021	10.1109/icievicivpr52578.2021.9564201	IEEE	off topic	no
deep learning for morphological arrhythmia classification in encoded ecg signal	2021	10.1109/icmla52953.2021.00096	IEEE	off topic	no
design of a low-power ecg detecting circuit	2021	10.1109/icmsp53480.2021.9513348	IEEE	hardware	no
machine learning and iot based solutions for detection of arrhythmia using ecg signals	2021	10.1109/icoei51242.2021.9452907	IEEE	off topic	no
early detection of cardiac arrhythmia disease using machine learning and iot technologies	2021	10.1109/icosec51865.2021.9591884	IEEE	off topic	no
r-peak detection for ecg biomedical monitoring	2021	10.1109/icpics52425.2021.9524155	IEEE	duplicate	no
automated cardiac condition diagnosis using ai based ecg analysis system for school children	2021	10.1109/icscc51209.2021.9528098	IEEE	off topic	no
efficient ecg analysis with high f1 score and low computation complexity	2021	10.1109/idaacs53288.2021.9660931	IEEE	off topic	no

study on monitoring respiratory frequency by smart phone based on photoelectric volume pulse wave method	2021	10.1109/ ipec51340.2021.9421320	IEEE	off topic	no
a 2.52 µα wearable single lead ternary neural network based cardiac arrhythmia detection processor	2021	10.1109/ iscas51556.2021.9401054	IEEE	hardware	no
wearable devices acquired ecg signals detection method using 1d convolutional neural network	2021	10.1109/ ismict51748.2021.9434935	IEEE	off topic	no
fpga based ecg denoising: current status and future technologies	2021	10.1109/ ispcc53510.2021.9609422	IEEE	hardware	no
real-time cnn based st depression episode detection using single-lead ecg	2021	10.1109/ isqed51717.2021.9424275	IEEE	off topic	no
input feature selection in ecg signal data modelling using long short term memory	2021	10.1109/ isriti54043.2021.9702810	IEEE	off topic	no
automatic detection of qrs complexes using dual channels based on u-net and bidirectional long short-term memory	2021	10.1109/ jbhi.2020.3018563	IEEE	mutichannel	yes
machine learning for real-time heart disease prediction	2021	10.1109/ jbhi.2021.3066347	IEEE	duplicate	no
hardware emulation of a biorthogonal wavelet transform-based heart rate monitoring device	2021	10.1109/ jsen.2020.3034742	IEEE	hardware	no
a wearable wireless sensor system using machine learning classification to detect arrhythmia	2021	10.1109/ jsen.2021.3062395	IEEE	off topic	no
wearable physiological multi-vital sign monitoring system with medical standard	2021	10.1109/ jsen.2021.3123084	IEEE	mutichannel	no
automated detection of posterior myocardial infarction from vectorcardiogram signals using fourier–bessel series expansion based empirical wavelet transform	2021	10.1109/ lsens.2021.3070142	IEEE	off topic	no
automated biosignal quality analysis of electrocardiograms	2021	10.1109/ mim.2021.9400951	IEEE	off topic	no
ecg abnormality classification and analysis with svm classifier	2021	10.1109/ mysurucon52639.2021.9641722	IEEE	off topic	no

the way of ecg signal obtaining from the respiratory wave by savitzky-golay filtration	2021	10.1109/ nanofim54124.2021. 9737356	IEEE	off topic	no
real time heart beat monitoring with labview	2021	10.1109/ ocit53463.2021.0001 4	IEEE	hardware	no
deep learning based atrial fibrillation detection using effective denoising methods and dimensionality reduction techniques	2021	10.1109/r10- htc53172.2021.9641 550	IEEE	off topic	no
classification of heart disease from ecg signals using machine learning	2021	10.1109/ rteict52294.2021.95 73659	IEEE	off topic	no
false positives avoidance in pulse detection from ecg and ppg sensor signals	2021	10.1109/ sensors47087.2021. 9639525	IEEE	off topic	no
development of a flexible wireless mwcnts-based ecg monitoring device	2021	10.1109/ sensors47087.2021. 9639596	IEEE	hardware	no
hardware-assisted and deep-learning techniques for low-power detection of cardiovascular abnormalities in smart wearables	2021	10.1109/ smartiot52359.2021. 00031	IEEE	hardware	no
a study on selection of hrv-based features for different classifiers in atrial fibrillation detection	2021	10.1109/ spsympo51155.2020 .9593769	IEEE	off topic	no
an ecg delineation and arrhythmia classification system using slope variation measurement by ternary second-order delta modulators for wearable ecg sensors	2021	10.1109/ tbcas.2021.3113665	IEEE	duplicate	no
fixed-point nlms and ipnlms vlsi architectures for accurate fecg and fhr processing	2021	10.1109/ tbcas.2021.3120237	IEEE	hardware	no
approximate pruned and truncated haar discrete wavelet transform vlsi hardware for energy-efficient ecg signal processing	2021	10.1109/ tcsi.2021.3057584	IEEE	hardware	no
r-peak detection from ecg signals using fractal based mathematical morphological operators	2021	10.1109/ tencon54134.2021.9 707247	IEEE	duplicate	no
ventricular arrhythmia classification and interpretation using residual neural network with guided backpropagation	2021	10.1109/ tencon54134.2021.9 707469	IEEE	off topic	no

detection of atrial fibrillation in compressively sensed electrocardiogram measurements	2021	10.1109/ tim.2020.3027930	IEEE	off topic	no
embedded algorithm for qrs detection based on signal shape	2021	10.1109/ tim.2021.3051412	IEEE	hardware	no
an automatic algorithm for p/t-wave detection based on auxiliary waveform	2021	10.1109/ trustcom53373.2021 .00214	IEEE	off topic	no
fpga implementation of iot-based health monitoring system	2021	10.1109/ tssa52866.2021.976 8261	IEEE	duplicate	no
study of the ventricular late potentials characteristics of the patients with heart conducting ways abnormality	2021	10.1109/ usbereit51232.2021. 9455036	IEEE	off topic	no
semi-supervised learning for ecg classification	2021	10.23919/ cinc53138.2021.966 2693	IEEE	duplicate	no
improving the microvolt t-wave alternans peak by changing the t-wave search window duration	2021	10.23919/ cinc53138.2021.966 2708	IEEE	off topic	no
qrs slopes for potassium and calcium monitoring in end-stage renal disease patients	2021	10.23919/ cinc53138.2021.966 2720	IEEE	duplicate	no
guinea pig ecg changes under the effect of new drug candidate tp28b	2021	10.23919/ cinc53138.2021.966 2793	IEEE	animal	no
wavelet transform based detection of the first-degree atrioventricular block	2021	10.23919/ cinc53138.2021.966 2877	IEEE	duplicate	no
a novel method for the detection of qrs complex using vectorcardiographic octants	2021	10.23919/ cinc53138.2021.966 2893	IEEE	duplicate	no
evaluating pauses in holter ecg signals	2021	10.23919/ cinc53138.2021.966 2914	IEEE	off topic	no
vdi vision - analysis of ventricular electrical dyssynchrony in real-time	2021	10.23919/ cinc53138.2021.966 2916	IEEE	off topic	no

uncovering electromechanical uncoupling in subclinical pathogenic mutation carriers and arrhythmogenic cardiomyopathy patients	2021	10.23919/cinc53138.2021.9662949	IEEE	duplicate	no
detection of obstructive sleep apnoea by ecg signals using deep learning architectures	2021	10.23919/eusipco47968.2020.9287360	IEEE	off topic	no
a method to detect ventricular fibrillation in electrocardiograms	2021	10.23919/mipro52101.2021.9596913	IEEE	off topic	no
a deep learning approach for the classification of arrhythmias in ecg signal	2021		IEEE	off topic	no
ai-based identification and prediction of cardiac disorders	2022	10.1002/9781119819165.ch8	IEEE	off topic	no
autoencoder and machine learning method for myocardial infarction (mi) detection application	2022	10.1049/icp.2023.0377	IEEE	off topic	no
detection of atrial fibrillation by custom designed convolutional neural network: a medical viewpoint	2022	10.1109/asyu56188.2022.9925304	IEEE	off topic	no
machine learning-based detection of in-utero fetal presentation from non-invasive fetal ecg	2022	10.1109/bhi56158.2022.9926804	IEEE	fetal	no
a lightweight r peak detection algorithm for noisy ecg signals	2022	10.1109/bibe55377.2022.00060	IEEE	missing technical details	no
pan-tompkins++: a robust approach to detect r-peaks in ecg signals	2022	10.1109/bibm55620.2022.9995552	IEEE	does not introduce a new technique	yes
accurately identifying coronary atherosclerotic heart disease through merged beats of electrocardiogram	2022	10.1109/bibm55620.2022.9995602	IEEE	off topic	no
automatic swt based qrs detection using weighted subbands and shannon energy peak amplification for ecg signal analysis devices	2022	10.1109/ccip57447.2022.10058632	IEEE	missing technical details	yes
ecg signal feature extraction and waveform representation	2022	10.1109/cict56698.2022.9997920	IEEE	off topic	no

intelligent detection of hypokalemia based on 12-lead ecg using two-stream deep learning model	2022	10.1109/cisp-bmei56279.2022.9980196	IEEE	mutichannel	no
ecg noise removal using fcn dae method	2022	10.1109/conit55038.2022.9847756	IEEE	off topic	no
population based approach for detection myocardial scar based on 12-lead ecg: simulation study	2022	10.1109/csgb56354.2022.9865264	IEEE	mutichannel	no
deep learning assisted tool for atrial fibrillation detection using rr intervals	2022	10.1109/csi54720.2022.9924134	IEEE	off topic	no
simple and effective signal processing pinpointing subtle premature ventricular contractions inferred from increasing physical effort	2022	10.1109/csndsp54353.2022.9907943	IEEE	off topic	no
qt-stnet: a spatial and temporal network combined with qt segment for mi detection and location	2022	10.1109/cyberc55534.2022.00038	IEEE	off topic	no
machine learning algorithms for atrioventricular conduction defects prediction using ecg: a comparative study	2022	10.1109/delcon54057.2022.9753488	IEEE	off topic	no
premature ventricular contraction detection algorithm based on robust feature extraction	2022	10.1109/ecice55674.2022.10042957	IEEE	off topic	no
fetal heart rate extraction from abdominal electrocardiography recordings based on wavelet transform and adaptive threshold algorithm	2022	10.1109/ehb55594.2022.9991339	IEEE	fetal	no
an algorithm for locating reference points of cardiac motion signals based on non-contact	2022	10.1109/eiect58010.2022.00088	IEEE	off topic	no
detecting obstructive apnea episodes using dynamic bayesian networks and ecg-based time-series	2022	10.1109/embc48229.2022.9870930	IEEE	off topic	no
a new multimodal device for atrial fibrillation detection: ecg quality analysis in healthy volunteers	2022	10.1109/embc48229.2022.9871207	IEEE	off topic	no

cnn-based two step r peak detection method: combining segmentation and regression	2022	10.1109/embc48229.2022.9871227	IEEE	duplicate	no
heartbeat detection in seismocardiograms with semantic segmentation	2022	10.1109/embc48229.2022.9871477	IEEE	duplicate	no
threshold-based anxiety detection algorithm through ecg and gsr signals	2022	10.1109/etcm56276.2022.9935706	IEEE	off topic	no
lmau-net: a local mask attention convolutional neural network for qrs wave detection	2022	10.1109/hdis56859.2022.9991510	IEEE	off topic	no
analysis of arrhythmia classification on ecg dataset	2022	10.1109/i2ct54291.2022.9825052	IEEE	off topic	no
using the gan method, analysis several characteristics of the ecg signal in order to detect cardiac arrhythmia	2022	10.1109/i2ct54291.2022.9825337	IEEE	off topic	no
classification of ventricular arrhythmia using machine learning and deep learning techniques	2022	10.1109/i4c57141.2022.10057825	IEEE	off topic	no
complexity comparison of empirical mode decomposition and wavelet decomposition methods in the detection of ventricular late potential	2022	10.1109/iatmsi56455.2022.10119348	IEEE	off topic	no
classification and segmentation of electrocardiogram signals using machine learning approach	2022	10.1109/ic3sis54991.2022.9885654	IEEE	off topic	no
application of improved differential algorithm in wearable device ecg monitoring	2022	10.1109/iccsi55536.2022.9970596	IEEE	off topic	no
analysis of electrocardiographic signals using a new method based on the principles of wavelet processing and gauss-hermit functions	2022	10.1109/icct56057.2022.9976684	IEEE	inaccessible dataset	no
wearable ecg devices: a state of the art about circuit, software and application	2022	10.1109/icedcs57360.2022.00099	IEEE	off topic	no

automatic detection of fetal qrs complex using time-frequency image based features and deep learning architecture	2022	10.1109/icesc54411.2022.9885294	IEEE	fetal	no
classification of ecg signal using cnn algorithm	2022	10.1109/icesic53714.2022.9783598	IEEE	off topic	no
automated prediction and occurence of tachyarrhythmia using deep learning techniques	2022	10.1109/icicict54557.2022.9917672	IEEE	off topic	no
signal quality evaluation and processing for qrs detection in ecg based smart healthcare systems	2022	10.1109/ickecs56523.2022.10060422	IEEE	off topic	no
on the generalizability of ecg-based stress detection models	2022	10.1109/icmla55696.2022.00090	IEEE	off topic	no
machine learning approach for estimation of high acquity ecg monitoring system	2022	10.1109/iconsip49665.2022.10007521	IEEE	inaccessible dataset	no
amplitude and frequency based evaluations for algorithm development of premature ventricular contraction detection system	2022	10.1109/icoris56080.2022.10031562	IEEE	duplicate	no
efficient prediction of heart diseases by using machine learning classifiers	2022	10.1109/ictacs56270.2022.9988582	IEEE	off topic	no
atrial fibrillation classification and prediction explanation using transformer neural network	2022	10.1109/ijcnn55064.2022.9892286	IEEE	off topic	no
data extraction from an electrocardiogram signal and qrs peak detection employing wavelet-based approach	2022	10.1109/incet54531.2022.9824913	IEEE	duplicate	no
monitoring cardiac activity by detecting subtle head movements using mems technology	2022	10.1109/inertial53425.2022.9787725	IEEE	off topic	no
biometric individual authentication system using high performance ecg fiducial features	2022	10.1109/isia55826.2022.9993496	IEEE	off topic	no

real-time detection of premature ventricular contraction using discrete wavelet transform	2022	10.1109/ isitia56226.2022.985 5271	IEEE	off topic	no
on the framework of cardiac arrhythmia characterization using morphological and statistical features	2022	10.1109/ ispa54004.2022.978 6291	IEEE	off topic	no
identification of morphological patterns for the detection of premature ventricular contractions	2022	10.1109/ iv56949.2022.00071	IEEE	off topic	no
classification of ecg signals for detecting coronary heart diseases using deep transfer learning techniques	2022	10.1109/jac-ecc56395.2022.1004 3970	IEEE	off topic	no
a stochastic resonance electrocardiogram enhancement algorithm for robust qrs detection	2022	10.1109/ jbhi.2022.3178109	IEEE	INCLUDED	yes
robust arrhythmia classification based on qrs detection and a compact 1d-cnn for wearable ecg devices	2022	10.1109/ jbhi.2022.3207456	IEEE	off topic	no
a multimodal data fusion technique for heartbeat detection in wearable iot sensors	2022	10.1109/ jiot.2021.3093112	IEEE	mutichannel	no
a near-sensor ecg delineation and arrhythmia classification system	2022	10.1109/ jsen.2022.3183136	IEEE	off topic	no
a real-time tunable ecg noise-aware system for iot-enabled devices	2022	10.1109/ jsen.2022.3211318	IEEE	missing accuracy	no
adaptive r-peak detector in extreme noise using emd selective analyzer	2022	10.1109/ memea54994.2022. 9856531	IEEE	duplicate	no
deepecg :building an efficient framework for automatic arrhythmia classification model	2022	10.1109/ miucc55081.2022.97 81646	IEEE	off topic	no
hardware emulation of ecg sensors for biomedical embedded system design and teaching	2022	10.1109/ mysurucon55714.20 22.9972545	IEEE	hardware	no
ecg r-r interval automatic detection using iot	2022	10.1109/ nkcon56289.2022.10 126731	IEEE	duplicate	no
classification of ecg arrhythmia using artificial intelligence techniques (rbf and svm)	2022	10.1109/ pais56586.2022.994 6873	IEEE	off topic	no

ecg characteristic detection using densenet based on attention mechanism and feature pyramid	2022	10.1109/prml56267.2022.9882215	IEEE	off topic	no
an area and power efficient vlsi architecture to detect obstructive sleep apnea for wearable devices	2022	10.1109/radioelektronika54537.2022.9764917	IEEE	duplicate	no
characteristics of pulse waveform in chronic atrial fibrillation: simultaneous comparison with ecg	2022	10.1109/scisisis55246.2022.10001913	IEEE	off topic	no
detection algorithm of qrs wave and r peak based on adaptive energy segmentation	2022	10.1109/sdpc55702.2022.9915890	IEEE	missing technical details	no
effects of geometry on performances of optically unobtrusive zeolite-based electrodes	2022	10.1109/sensors52175.2022.9967325	IEEE	off topic	no
common spatial pattern with deep learning for fetal heart rate monitoring	2022	10.1109/sips55645.2022.9919257	IEEE	duplicate	no
support vector machine for heart beats classification based on robust filtering	2022	10.1109/ssd54932.2022.9955703	IEEE	missing accuracy	yes
ecg arrhythmia classification on an ultra-low-power microcontroller	2022	10.1109/tbcas.2022.3182159	IEEE	hardware	no
a 746 nw ecg processor asic based on ternary neural network	2022	10.1109/tbcas.2022.3196059	IEEE	duplicate	no
robust r-peak detection in low-quality holter ecgs using 1d convolutional neural network	2022	10.1109/tbme.2021.3088218	IEEE	INCLUDED	yes
from continuous ecg signals to extracted features for machine learning models and arrhythmia annotations	2022	10.1109/telfor56187.2022.9983663	IEEE	off topic	no
pa²net: period-aware attention network for robust fetal ecg detection	2022	10.1109/tim.2022.3189742	IEEE	fetal	no
interpretation and classification of arrhythmia using deep convolutional network	2022	10.1109/tim.2022.3204316	IEEE	off topic	no
automatic analysis of sleep apnea using dnn andhrv features	2022	10.1109/wiecon-ece57977.2022.10150724	IEEE	off topic	no

cardiac monitoring with novel low power sensors measuring upper thoracic electrostatic charge variation for long lasting wearable devices	2022	10.1109/ wimob55322.2022.9 941545	IEEE	off topic	no
arrhythmia detection using spiking variable projection neural networks	2022	10.22489/ cinc.2022.049	IEEE	off topic	no
decision tree-based model for signal quality scanning in wearable ecg	2022	10.22489/ cinc.2022.121	IEEE	duplicate	no
relationship between ecg-pattern of depolarization abnormalities and an mildly reduced ejection fraction	2022	10.22489/ cinc.2022.204	IEEE	off topic	no
automated algorithm for qrs detection in cardiac arrest patients with pea	2022	10.22489/ cinc.2022.270	IEEE	duplicate	no
ai-enabled ecg combined with dry electrode sensors for population-based screening of atrial fibrillation	2022	10.22489/ cinc.2022.312	IEEE	off topic	no
cer-s, an ecg platform for the management of continuous ecg recordings and databases	2022	10.22489/ cinc.2022.336	IEEE	off topic	no
a novel deep learning-based approach for sleep apnea detection using single-lead ecg signals	2022	10.23919/ apsipaasc55919.202 2.9979890	IEEE	off topic	no
detecting ventricular beats with machine learning models	2022	10.23919/ mipro55190.2022.98 03758	IEEE	off topic	no
ieee draft standard for health informatics--personal health device communication part 10406: device specialization--basic electrocardiograph (ecg) (1- to 3-lead ecg)	2022		IEEE	duplicate	no
ieee draft standard for health informatics--personal health device communication part 10406: device specialization--basic electrocardiograph (ecg) (1- to 3-lead ecg)	2022		IEEE	duplicate	no
criteria decision analysis based cardiovascular diseases classifier for drunk driver detection	2023	10.1002/978111979 0402.ch18	IEEE	off topic	no
cardiac arrhythmia detection using convolutional neural network	2023	10.1049/ icp.2023.2863	IEEE	off topic	no
practical r-r interval editing for heart rate variability analysis using single-channel wearable ecg devices	2023	10.1109/ access.2023.325393 3	IEEE	off topic	yes

sudden cardiac death detection by using an hybrid method based on twa and dictionary learning: a data experimentation	2023	10.1109/access.2023.3277396	IEEE	off topic	no
extracting fetal ecg signals through a hybrid technique utilizing two wavelet-based denoising algorithms	2023	10.1109/access.2023.3308409	IEEE	duplicate	no
an adaptive sleep apnea detection model using multi cascaded atrous-based deep learning schemes with hybrid artificial humming bird pity beetle algorithm	2023	10.1109/access.2023.3319452	IEEE	off topic	no
wireless healthcare monitoring system for heart diseases classification using efficient ecg-based wave modeling and machine learning techniques	2023	10.1109/actea58025.2023.10193969	IEEE	off topic	no
activity monitoring and alert system for elderly people in smart homes	2023	10.1109/aisp57993.2023.10134876	IEEE	off topic	no
a low-complexity r-peak detection based on exponential weight mean-variance for wearable ecg devices	2023	10.1109/atc58710.2023.10318901	IEEE	duplicate	no
unraveling the potential of heart rate variability for type 1 diabetes prediction	2023	10.1109/bats59463.2023.10303132	IEEE	off topic	no
fast r-peak detection from compressed ecg sensing measurements without reconstruction for energy-constrained cardiac health monitoring	2023	10.1109/biosmart58455.2023.10162031	IEEE	does not introduce a new technique	no
high-precision rhythm-based detection of atrial fibrillation and frequent premature contractions using extreme gradient boosting	2023	10.1109/bmeicon60347.2023.10322038	IEEE	duplicate	no
ecg heartbeat signal classification and detection of cardiac abnormalities using deep learning	2023	10.1109/ccpis59145.2023.10291586	IEEE	duplicate	no
a 2.67gω 454nvrms 14.9μw dry-electrode enabled ecg-on-chip with arrhythmia detection	2023	10.1109/cicc57935.2023.10121299	IEEE	hardware	no
high quality ecg dataset based on mit-bih recordings for improved heartbeats classification	2023	10.1109/coins57856.2023.10189299	IEEE	duplicate	no

a comparative study on wavelet transform-based algorithm for calculating heart rate from ballistocardiography	2023	10.1109/comsnets56262.2023.10041401	IEEE	off topic	no
electrocardiogram detection system of autistic children based on ad8232 for healthcare	2023	10.1109/cosite60233.2023.10250117	IEEE	off topic	no
statistical analysis of cross-correlation index for identifying abnormal ecg signals	2023	10.1109/csci62032.2023.00234	IEEE	off topic	no
anxiogram: unmasking anxiety with iot- enhanced ecg	2023	10.1109/csitss60515.2023.10334100	IEEE	off topic	no
techniques and methods for anomalies detection in ecg as a support for medical decision in healthcare: a review	2023	10.1109/dasa59624.2023.10286626	IEEE	off topic	no
memory classifiers for robust ecg classification against physiological noise	2023	10.1109/embc40787.2023.10339980	IEEE	off topic	no
pulsation artifact removal from intra-operatively recorded local field potentials using sparse signal processing and data-specific dictionary	2023	10.1109/embc40787.2023.10340160	IEEE	duplicate	no
new features for the detection of fetal qrs complexes in non-invasive fetal electrocardiograms	2023	10.1109/embc40787.2023.10340399	IEEE	duplicate	no
accurate ventricular tachyarrhytmia beats detection on low sample rate ecg patch signals using 1d u-net	2023	10.1109/embc40787.2023.10340592	IEEE	off topic	no
atrial fibrillation and atrial flutter detection using electrocardiomatrix technique from ecg signal	2023	10.1109/i2ct57861.2023.10126168	IEEE	off topic	no
low-cost ecg monitoring system with classification using deep learning	2023	10.1109/icaeccs56710.2023.10104707	IEEE	off topic	no
performance evaluation of classifiers for ecg signal analysis	2023	10.1109/icaia57370.2023.10169512	IEEE	off topic	no

learning techniques of ecg arrhythmia classification: a review	2023	10.1109/ icccee55951.2023.10424554	IEEE	off topic	no
a novel geometric visualization of dual-lead ecg signals for beat-by-beat arrhythmia detection with a 2d cnn deep learning classifier	2023	10.1109/ iccit60459.2023.10441002	IEEE	mutichannel	no
deep learning-based heartbeat classification of 12-lead ecg time series signal	2023	10.1109/ icdabi60145.2023.10629531	IEEE	mutichannel	no
denoising ecg signals and their analysis using hybrid deep learning model	2023	10.1109/ icdt57929.2023.10150811	IEEE	off topic	no
a review on analysis of cardiac arrhythmia from heart beat classification	2023	10.1109/ icears56392.2023.10085295	IEEE	review	no
meticulous ecg - a continuous monitoring and early detection system of arrhythmia detection	2023	10.1109/ iceca58529.2023.10395043	IEEE	off topic	no
lab view based design of arrhythmia detection system	2023	10.1109/ iceca58529.2023.10395584	IEEE	off topic	no
exploring the feasibilities of applying min-max threshold analysis with machine learning techniques for categorization of x-wave in ecg signal	2023	10.1109/ icecaa58104.2023.10212176	IEEE	off topic	no
detection and analysis of cardiac arrhythmias from heartbeat classification	2023	10.1109/ iceeict56924.2023.10156983	IEEE	off topic	no
detection of cardiac arrhythmia using machine learning	2023	10.1109/ icimia60377.2023.10425867	IEEE	off topic	no
impact of linearization in abdominal ecg for non-causal filtering structure in fetal ecg extraction	2023	10.1109/ icoei56765.2023.10125877	IEEE	off topic	no
a deep learning based approach for cardiovascular monitoring using seismocardiogram signals	2023	10.1109/ icpeev58650.2023.10391941	IEEE	off topic	no

ecg dimensionality reduction using pca and feature abstraction expending ica built with power spectral estimation	2023	10.1109/icsc60394.2023.10441071	IEEE	off topic	no
obstructive sleep apnea detection from ecg signals with deep learning	2023	10.1109/icscss57650.2023.10169207	IEEE	off topic	no
prediction of ecg cardiac abnormality signal using supervised prediction model	2023	10.1109/icspc59664.2023.10420227	IEEE	duplicate	no
kpca and svr-based cardiac arrhythmia classification on electrocardiography waves	2023	10.1109/icssit55814.2023.10061047	IEEE	off topic	no
performance analysis of fir filters using high speed vlsi adders for fecg monitoring	2023	10.1109/icsss58085.2023.10407053	IEEE	off topic	no
machine learning methods for ecg arrhythmia beat classification	2023	10.1109/icuis60567.2023.00017	IEEE	off topic	no
detecting arrhythmia heart condition using supervised machine learning algorithms	2023	10.1109/ieeeconf58110.2023.10520581	IEEE	off topic	no
a portable and mobile systems for identifying cardiac arrhythmia using naïve bayes	2023	10.1109/ies59143.2023.10242509	IEEE	off topic	no
multi-resolution wavelet investigation approach for abstraction of electrocardiogram (ecg) characteristic features	2023	10.1109/ihcsp56702.2023.10127117	IEEE	mutichannel	no
effective delineation of ecg signals for operative neural disease recognition and classification	2023	10.1109/ihcsp56702.2023.10127144	IEEE	off topic	no
depression analysis based on eeg and ecg signals	2023	10.1109/incet57972.2023.10170067	IEEE	duplicate	no
a comprehensive review on monitoring sensors for cardiovascular disease prevention and management	2023	10.1109/iraset57153.2023.10152939	IEEE	review	no

research on qrs wave detection algorithm of ecg system	2023	10.1109/itnec56291.2023.10082483	IEEE	duplicate	no
non-contact estimation of cardiac inter-beat interval and heart rate variability using time-frequency domain analysis for cw radar	2023	10.1109/jerm.2023.3326562	IEEE	off topic	no
iomt-enabled stress monitoring in a virtual reality environment and at home	2023	10.1109/jiot.2023.3240099	IEEE	off topic	no
an iot-based noncontact ecg system: sole of the feet/hands palm	2023	10.1109/jiot.2023.3283037	IEEE	off topic	no
variational mode decomposition-based simultaneous r peak detection and noise suppression for automatic ecg analysis	2023	10.1109/jsen.2023.3257332	IEEE	duplicate	no
higher order time-frequency domain tensor-based method for the detection of atrial abnormalities using 12-lead ecg signals	2023	10.1109/lsens.2023.3296347	IEEE	mutichannel	no
an electrocardiogram augmentation method for automatic detection of st-segment elevation myocardial infarction and culprit vessel	2023	10.1109/medai59581.2023.00055	IEEE	off topic	no
ecg wave segmentation algorithm for complete p-qrs-t detection	2023	10.1109/memea57477.2023.10171894	IEEE	duplicate	no
design and evaluation of a wearable single-lead ecg for continuous monitoring	2023	10.1109/metroxraine58569.2023.10405813	IEEE	duplicate	no
detection of life-threatening malignant cardiac arrhythmias using machine learning methods	2023	10.1109/mi-sta57575.2023.10169565	IEEE	off topic	no
tensor-domain machine learning based cardiac diseases detection using 12-lead ecg	2023	10.1109/ncc56989.2023.10068027	IEEE	duplicate	no
understanding the impacts of ecg signal processing techniques on recognition of cardiovascular diseases	2023	10.1109/r10-htc57504.2023.10461836	IEEE	off topic	no
acquiring favorable ecg signal from low-cost devices	2023	10.1109/sami58000.2023.10044482	IEEE	off topic	no

resource-constrained device characterization for detecting sleep apnea using machine learning	2023	10.1109/smartnets58706.2023.10216211	IEEE	off topic	no
heartbeat classification of arrhythmia using hybrid features extraction techniques	2023	10.1109/ssd58187.2023.10411288	IEEE	off topic	no
evaluation and categorization of ecg signal for arrhythmia detection with cnn - alexnet	2023	10.1109/stcr59085.2023.10396972	IEEE	off topic	no
a 2.2 nw analog electrocardiogram processor based on stochastic resonance achieving a 99.94% qrs complex detection sensitivity	2023	10.1109/tbcas.2023.3235786	IEEE	duplicate	no
second-order level-crossing sampling analog to digital converter for electrocardiogram delineation and premature ventricular contraction detection	2023	10.1109/tbcas.2023.3296529	IEEE	duplicate	no
adaptive r-peak detection on wearable ecg sensors for high-intensity exercise	2023	10.1109/tbme.2022.3205304	IEEE	duplicate	no
identifying electrocardiogram abnormalities using a handcrafted-rule-enhanced neural network	2023	10.1109/tcbb.2022.3140785	IEEE	off topic	no
minsc: a vlsi architecture for myocardial infarction stages classifier for wearable healthcare applications	2023	10.1109/tcsii.2022.3222738	IEEE	hardware	no
ecg signals segmentation using deep spatiotemporal feature fusion u-net for qrs complexes and r-peak detection	2023	10.1109/tim.2023.3241997	IEEE	duplicate	no
detection of premature ventricular contractions using machine learning	2023	10.1109/tiptekno59875.2023.10359234	IEEE	off topic	no
efficient and real-time approach for pqrst and atrial fibrillation detection of ecg signal	2023	10.1109/tsp59544.2023.10197821	IEEE	inaccessible dataset	no
cardiaware: a novel ecg-based deep neural network algorithm for early detection of cardiac conditions	2023	10.1109/urtc60662.2023.10535038	IEEE	off topic	no
pattern recognition of ecg signal to detect heart disorders	2023	10.1109/vitecon58111.2023.10157257	IEEE	duplicate	no

ultra-low power non-uniform sar adc based ecg detector for early detection of cardiovascular diseases	2023	10.1109/vlsid57277.2023.00032	IEEE	hardware	no
deep learning-based signal quality assessment in wearable ecg monitoring	2023	10.22489/cinc.2023.017	IEEE	off topic	no
an ensemble of machine learning models for multilabel classification of cardiovascular diseases by ecgs	2023	10.22489/cinc.2023.258	IEEE	duplicate	no
characterising rr intervals in atrial fibrillation detected through screening	2023	10.22489/cinc.2023.270	IEEE	off topic	no
extraction of fetal electrocardiogram by combining deep learning and svd-ica-nmf methods	2023	10.26599/bdma.2022.9020035	IEEE	fetal	no
ieee standard for health informatics--device interoperability part 10406: personal health device communication--device specialization--basic electrocardiography (ecg) (1- to 3-lead ecg) - redline	2023		IEEE	off topic	no
detection of events	2024	10.1002/9781119825883.ch4	IEEE	off topic	no
a novel ecg signal quality index method based on skewness-modwt analysis	2024	10.1109/access.2024.3401708	IEEE	off topic	no
detecting sinus bradycardia from ecg signals using signal processing and machine learning	2024	10.1109/aimhc59811.2024.00016	IEEE	off topic	no
detection of potential hypertension with pan tompkins extraction and naive bayes classifier methods	2024	10.1109/aims61812.2024.10513252	IEEE	off topic	no
detecting arrhythmias using ecg signals with machine learning techniques	2024	10.1109/hora61326.2024.10550907	IEEE	off topic	no
developing deep learning model for abnormal heartbeat detection in electrocardiography signals	2024	10.1109/idciot59759.2024.10467337	IEEE	off topic	no
a peak-detector-based ultra low power ecg asic for early detection of cardio-vascular diseases	2024	10.1109/iscas58744.2024.10558031	IEEE	hardware	no
mddbranchnet: a deep learning model for detecting major depressive disorder using ecg signal	2024	10.1109/jbhi.2024.3390847	IEEE	off topic	no

computational paradigms for heart arrhythmia detection: leveraging neural networks	2024	10.1109/sami60510.2024.10432860	IEEE	off topic	no
hybrid amplitude ordinal partition networks for ecg morphology discrimination: an application to pvc recognition	2024	10.1109/tim.2024.3400307	IEEE	off topic	no
driver drowsiness detection using r-r interval of electrocardiogram and self-attention autoencoder	2024	10.1109/tiv.2023.3308575	IEEE	off topic	no
delta wave detection method of wolff-parkins on-white syndrome based on electrocardiogram	2024	10.1109/tsp63128.2024.10605767	IEEE	off topic	no
[a heart rate detection method for wearable electrocardiogram with the presence of motion interference]	2021	10.7507/1001-5515.202011011	PUBMED	not accessible	no
[a novel and fast p wave detection algorithm in ecg signals]	2017		PUBMED	off topic	no
[assessment of myocardial electrical dissynchrony by noninvasive activation mapping and its role in achieving the success of cardiac resynchronization]	2019	10.18087/cardio.2613	PUBMED	off topic	no
[automatic detection of ventricular and supraventricular wide qrs arrhythmias using complex of morphological criteria and algorithms]	2019	10.18087/cardio.2659	PUBMED	language not supported	no
[design and implementation of portable abnormal ecg signal analysis instrument based on feature classifcation]	2018	10.3969/j.issn.1671-7104.2018.02.006	PUBMED	off topic	no
[detection of inferior myocardial infarction based on morphological characteristics]	2021	10.7507/1001-5515.202001027	PUBMED	off topic	no
[ecg abnormalities in athletes as compare to healthy subjects]	2020		PUBMED	off topic	no
[esophageal-ecg in the emergency department]	2024		PUBMED	duplicate	no
[experimental study of mitochondrion-targeted small molecule ir-61 ameliorated exhaustive exercise-induced cardiac injury in rats]	2022	10.12047/j.cjap.6260.2022.093	PUBMED	off topic	no
[fatal flecainide intoxication in a 17-year-old girl]	2018	10.1007/s00101-018-0434-5	PUBMED	off topic	no
[features of vectorcardiograms in patients with hypertension complicated by chronic heart failure with reduced left ventricle ejection fraction]	2022	10.26442/00403660.2022.09.201843	PUBMED	off topic	no

[research on the detection algorithm of electrocardiogram characteristic wave based on energy segmentation and stationary wavelet transform]	2021	10.7507/1001-5515.202002038	PUBMED	duplicate	no
3.6 mw active-electrode ecg/eti sensor system using wideband low-noise instrumentation amplifier and high impedance balanced current driver	2023	10.3390/s23052536	PUBMED	duplicate	no
72 hour holter monitoring, 7 day holter monitoring, and 30 day intermittent patient-activated heart rhythm recording in detecting arrhythmias in cryptogenic stroke patients free from arrhythmia in a screening 24 h holter	2020	10.1515/med-2020-0203	PUBMED	off topic	no
a 1.2nw analog electrocardiogram processor achieving a 99.63% qrs complex detection sensitivity	2021	10.1109/tbcas.2021.3092729	PUBMED	hardware	no
a 2.2 nw analog electrocardiogram processor based on stochastic resonance achieving a 99.94% qrs complex detection sensitivity	2023	10.1109/tbcas.2023.3235786	PUBMED	duplicate	no
a 2.66 µw clinician-like cardiac arrhythmia watchdog based on p-qrs-t for wearable applications	2022	10.1109/tbcas.2022.3184971	PUBMED	hardware	no
a 410-nw efficient qrs processor for mobile ecg monitoring in 0.18-µm cmos	2017	10.1109/tbcas.2017.2731797	PUBMED	off topic	no
a 746 nw ecg processor asic based on ternary neural network	2022	10.1109/tbcas.2022.3196059	PUBMED	duplicate	no
a case of a complete atrioventricular canal defect in a ferret	2021	10.1186/s12917-020-02736-2	PUBMED	off topic	no
a case of multiple cardiovascular and tracheal anomalies presented with wolff-parkinson-white syndrome in a middle-aged adult	2017	10.3346/jkms.2017.32.12.2069	PUBMED	off topic	no
a case report of flecainide toxicity with review of literature	2022	10.7759/cureus.22261	PUBMED	off topic	no
a clustering-based method for single-channel fetal heart rate monitoring	2018	10.1371/journal.pone.0199308	PUBMED	fetal	no
a comparative study of the validity and reliability of two wireless telemetry electrocardiogram devices in the emergency medicine department	2019	10.12659/msm.913299	PUBMED	off topic	no
a comparative study on neural networks for paroxysmal atrial fibrillation events detection from electrocardiography	2022	10.1016/j.jelectrocard.2022.10.002	PUBMED	off topic	no

a comparison of ecg-based home monitoring devices in adults with chd	2023	10.1017/s1047951122002244	PUBMED	off topic	no
a comparison of manual electrocardiographic interval and waveform analysis in lead 1 of 12-lead ecg and apple watch ecg: a validation study	2020	10.1016/j.cvdhj.2020.07.002	PUBMED	multichannel	no
a comparison of methods to suppress electrocardiographic artifacts in local field potential recordings	2023	10.1016/j.clinph.2022.11.011	PUBMED	denoising	no
a comprehensive comparison of six publicly available algorithms for localization of qrs complex on electrocardiograph	2023	10.1109/embc40787.2023.10340013	PUBMED	off topic	no
a comprehensive review on the electrocardiographic manifestations of cardiac sarcoidosis: patterns and prognosis	2024	10.1007/s11886-024-02088-5	PUBMED	review	no
a computational investigation into rate-dependant vectorcardiogram changes due to specific fibrosis patterns in non-ischæmic dilated cardiomyopathy	2020	10.1016/j.compbiomed.2020.103895	PUBMED	off topic	no
a convolutional neural network for ecg annotation as the basis for classification of cardiac rhythms	2018	10.1088/1361-6579/aae304	PUBMED	off topic	no
a deep learning algorithm to detect anaemia with ecgs: a retrospective, multicentre study	2020	10.1016/s2589-7500(20)30108-4	PUBMED	duplicate	no
a deep learning approach for fetal qrs complex detection	2018	10.1088/1361-6579/aab297	PUBMED	fetal	no
a deep learning approach identifies new ecg features in congenital long qt syndrome	2022	10.1186/s12916-022-02350-z	PUBMED	off topic	no
a deep learning architecture using 3d vectorcardiogram to detect r-peaks in ecg with enhanced precision	2023	10.3390/s23042288	PUBMED	duplicate	no
a delayed diagnosis of catecholaminergic polymorphic ventricular tachycardia with a mutant of ryr2 at c.7580t>g for 6 years in a 9-year-old child	2018	10.1097/md.0000000000010368	PUBMED	off topic	no
a digital electrocardiographic system for assessing myocardial electrical instability: principles and applications	2021	10.17691/stm2020.12.6.02	PUBMED	off topic	no
a fatal case of myocarditis following myositis induced by pembrolizumab treatment for metastatic upper urinary tract urothelial carcinoma	2020	10.1536/ihj.20-162	PUBMED	off topic	no

a graph-constrained changepoint detection approach for ecg segmentation	2020	10.1109/embc44109.2020.9175333	PUBMED	off topic	no
a greedy graph search algorithm based on changepoint analysis for automatic qrs complex detection	2021	10.1016/j.compbiomed.2021.104208	PUBMED	duplicate	no
a high accuracy & ultra-low power ecg-derived respiration estimation processor for wearable respiration monitoring sensor	2022	10.3390/bios12080665	PUBMED	off topic	no
a high precision real-time premature ventricular contraction assessment method based on the complex feature set	2019	10.1007/s10916-019-1443-x	PUBMED	off topic	no
a hybrid lossless ecg compressor with morphological detection and qrs prediction	2024	10.1109/embc53108.2024.10782467	PUBMED	multichannel	no
a left ventricular lead implantation at the latest site based on four-dimensional computed tomography: a case report	2020	10.1093/ehjcr/ytaa033	PUBMED	off topic	no
a lightweight qrs detector for single lead ecg signals using a max-min difference algorithm	2017	10.1016/j.cmpb.2017.02.028	PUBMED	INCLUDED	yes
a machine learning algorithm for electrocardiographic fqrs quantification validated on multi-center data	2022	10.1038/s41598-022-10452-0	PUBMED	off topic	no
a machine learning approach for the detection of qrs complexes in electrocardiogram (ecg) using discrete wavelet transform (dwt) algorithm	2022	10.1155/2022/9023478	PUBMED	off topic	no
a machine-learning approach for detection and quantification of qrs fragmentation	2019	10.1109/jbhi.2018.2878492	PUBMED	duplicate	no
a method for assessing and monitoring consistency of nonclinical ecg analysis	2022	10.1016/j.vascn.2022.107189	PUBMED	off topic	no
a method to minimise the impact of ecg marker inaccuracies on the spatial qrs-t angle: evaluation on 1,512 manually annotated ecgs	2021	10.1016/j.bspc.2020.102305	PUBMED	off topic	no
a modular low-complexity ecg delineation algorithm for real-time embedded systems	2018	10.1109/jbhi.2017.2671443	PUBMED	duplicate	no
a morphology based deep learning model for atrial fibrillation detection using single cycle electrocardiographic samples	2020	10.1016/j.ijcard.2020.04.046	PUBMED	off topic	no

a nanowatt real-time cardiac autonomic neuropathy detector	2018	10.1109/tbcas.2018.2833624	PUBMED	off topic	no
a new algorithm for fetal heart rate detection: fractional order calculus approach	2023	10.1016/j.medengphy.2023.104007	PUBMED	fetal	no
a new automatic qt-interval measurement method for wireless ecg monitoring system using smartphone	2021	10.31661/jbpe.v0i0.1912-1017	PUBMED	off topic	yes
a new bipolar precordial electrocardiographic lead configuration for specific evaluation of atrial depolarization in healthy dogs	2023	10.2460/ajvr.23.07.0151	PUBMED	off topic	no
a new database with annotations of p waves in ecgs with various types of arrhythmias	2022	10.1088/1361-6579/ac944e	PUBMED	off topic	no
a new deep learning algorithm of 12-lead electrocardiogram for identifying atrial fibrillation during sinus rhythm	2021	10.1038/s41598-021-92172-5	PUBMED	duplicate	no
a new deep learning method with self-supervised learning for delineation of the electrocardiogram	2022	10.3390/e24121828	PUBMED	duplicate	no
a new method for qrs complex detection in multichannel ecg: application to self-monitoring of fetal health	2017	10.1016/j.compbiomed.2016.04.008	PUBMED	multichannel	no
a new method for qrs detection in ecg signals using qrs-preserving filtering techniques	2018	10.1515/bmt-2016-0072	PUBMED	denoising	no
a new qrs detector stress test combining temporal jitter and f-score (jf) reveals significant performance differences amongst popular detectors	2024	10.1371/journal.pone.0309739	PUBMED	off topic	no
a new use for maximum deflection index: detection of intraventricular dyssynchrony	2017	10.1016/j.jelectrocard.2016.12.001	PUBMED	off topic	no
a new wavelet-based ecg delineator for the evaluation of the ventricular innervation	2017	10.1109/jtehm.2017.2722998	PUBMED	off topic	no
a novel algorithm based on ensemble empirical mode decomposition for non-invasive fetal ecg extraction	2021	10.1371/journal.pone.0256154	PUBMED	fetal	no
a novel algorithm for improving the prehospital diagnostic accuracy of st-segment elevation myocardial infarction	2024	10.1017/s1049023x23006635	PUBMED	off topic	no

a novel and lightweight p, qrs, and t peaks detector using adaptive thresholding and template waveform	2021	10.1016/ j.compbiomed.2021.104307	PUBMED	duplicate	no
a novel approach to the extraction of fetal electrocardiogram based on empirical mode decomposition and correlation analysis	2017	10.1007/s13246-017-0560-4	PUBMED	duplicate	no
a novel cluster-based method for single-channel fetal electrocardiogram detection	2021	10.1109/ embc46164.2021.9629848	PUBMED	fetal	no
a novel deep learning package for electrocardiography research	2022	10.1088/1361-6579/ac9451	PUBMED	not accessible	no
a novel ecg compression algorithm using pulse-width modulation integrated quantization for low-power real-time monitoring	2024	10.1038/s41598-024-68022-5	PUBMED	off topic	no
a novel ecg signal classification method using dea-elm	2020	10.1016/ j.mehy.2019.109515	PUBMED	off topic	no
a novel irbf-rvm model for diagnosis of atrial fibrillation	2019	10.1016/ j.cmpb.2019.05.028	PUBMED	off topic	no
a novel modular fetal ecg stan and hrv analysis: towards robust hypoxia detection	2019	10.3233/thc-181375	PUBMED	duplicate	no
a novel quantitative electrocardiography strategy reveals the electroinhibitory effect of tamoxifen on the mouse heart	2023	10.1007/s12265-023-10395-5	PUBMED	off topic	no
a novel sensor-array system for contactless electrocardiogram acquisition	2020	10.1109/ embc44109.2020.9175666	PUBMED	off topic	no
a novel short-term event extraction algorithm for biomedical signals	2018	10.1109/ tbme.2017.2718179	PUBMED	off topic	no
a novel technique for fetal ecg extraction using single-channel abdominal recording	2017	10.3390/s17030457	PUBMED	fetal	no
a novel wearable device for continuous ambulatory ecg recording: proof of concept and assessment of signal quality	2019	10.3390/ bios9010017	PUBMED	duplicate	no
a population-wide study of electrocardiographic (ecg) norms and the effect of demographic and anthropometric factors on selected ecg characteristics in young, southeast asian males-results from the singapore armed forces ecg (safe) study	2019	10.1111/anec.12634	PUBMED	off topic	no

a qrs detection and r point recognition method for wearable single-lead ecg devices	2017	10.3390/s17091969	PUBMED	INCLUDED	yes
a real time qrs detection algorithm based on et and pd controlled threshold strategy	2020	10.3390/s20144003	PUBMED	duplicate	no
a real-time arrhythmia heartbeats classification algorithm using parallel delta modulations and rotated linear-kernel support vector machines	2020	10.1109/ tbme.2019.2926104	PUBMED	off topic	no
a real-time qrs detection system with pr/rt interval and st segment measurements for wearable ecg sensors using parallel delta modulators	2018	10.1109/ tbcas.2018.2823275	PUBMED	duplicate	no
a review on atrial fibrillation detection from ambulatory ecg	2024	10.1109/ tbme.2023.3321792	PUBMED	review	no
a robust ecg denoising technique using variable frequency complex demodulation	2021	10.1016/ j.cmpb.2020.105856	PUBMED	denoising	no
a short p-wave duration is associated with incident heart failure in the elderly: a 15 years follow-up cohort study	2022	10.11909/ j.issn.1671-5411.2022.09.008	PUBMED	off topic	no
a signal quality assessment method for fetal qrs complexes detection	2023	10.3934/ mbe.2023344	PUBMED	fetal	no
a significance of school screening electrocardiogram in the patients with ventricular noncompaction	2020	10.1007/s00380-020-01571-7	PUBMED	off topic	no
a simple and effective deep neural network based qrs complex detection method on ecg signal	2024	10.3389/ fphys.2024.1384356	PUBMED	multichannel	yes
a stochastic resonance electrocardiogram enhancement algorithm for robust qrs detection	2022	10.1109/ jbhi.2022.3178109	PUBMED	duplicate	no
a systematic review of cardiac time intervals utilising non-invasive fetal electrocardiogram in normal fetuses	2018	10.1186/s12884-018-2006-8	PUBMED	fetal	no
a telediagnosis assistance system for multiple-lead electrocardiography	2021	10.1007/s13246-021-00996-2	PUBMED	off topic	no
a universal, high-performance ecg signal processing engine to reduce clinical burden	2022	10.1111/anec.12993	PUBMED	off topic	no
a visually interpretable detection method combines 3-d ecg with a multi-vgg neural network for myocardial infarction identification	2022	10.1016/ j.cmpb.2022.106762	PUBMED	off topic	no
a watch-type electrocardiography is a reliable tool for detecting paroxysmal cardiac arrhythmias	2022	10.3390/ jcm11123333	PUBMED	duplicate	no

abnormal blood lipid and electrocardiogram characteristics in common mental disorders	2023	10.1186/s12888-023-04965-9	PUBMED	duplicate	no
abnormal conduction zone detected by isochronal late activation mapping accurately identifies the potential atrial substrate and predicts the atrial fibrillation ablation outcome after pulmonary vein isolation	2023	10.1161/circep.122.011149	PUBMED	off topic	no
accuracy and usability of single-lead ecg from smartphones - a clinical study	2019	10.1016/j.ipej.2019.02.006	PUBMED	off topic	no
accuracy of ecg indices for diagnosis of left ventricular hypertrophy in people >65 years: results from the actife study	2017	10.1007/s40520-016-0667-6	PUBMED	off topic	no
accurate localization and catheter ablation of superoparaseptal accessory pathways	2018	10.1016/j.hrthm.2017.12.025	PUBMED	off topic	no
acquisition of electrocardiogram signals during magnetic resonance imaging	2017	10.1088/1361-6579/aa6e8c	PUBMED	off topic	no
acute high level noise exposure can cause physiological dysfunction in macaque monkeys: insight on the medical protection for special working environmental personnel	2021	10.3390/healthcare9070840	PUBMED	duplicate	no
acute osimertinib exposure induces electrocardiac changes by synchronously inhibiting the currents of cardiac ion channels	2023	10.3389/fphar.2023.1177003	PUBMED	duplicate	no
adaptive fourier decomposition based r-peak detection for noisy ecg signals	2017	10.1109/embc.2017.8037611	PUBMED	missing accuracy	yes
adaptive noise reduction algorithm to improve r peak detection in ecg measured by capacitive ecg sensors	2018	10.3390/s18072086	PUBMED	off topic	no
adaptive r-peak detection on wearable ecg sensors for high-intensity exercise	2023	10.1109/tbme.2022.3205304	PUBMED	duplicate	no
adaptive step size lms improves ecg detection during mri at 1.5 and 3 t	2017	10.1007/s10334-017-0638-8	PUBMED	off topic	no
advanced time-frequency methods for ecg waves recognition	2023	10.3390/diagnostics13020308	PUBMED	duplicate	no
aecg-decompnet: abdominal ecg signal decomposition through deep-learning model	2021	10.1088/1361-6579/abedc1	PUBMED	off topic	no
ai-enabled detection of qrs fragmentation from 12-lead electrocardiogram and its clinical relevance for predicting malignant arrhythmia onset	2024	10.3389/fcvm.2024.1464303	PUBMED	multichannel	no

aiding gastrointestinal diagnostic laboratory by designing a device for the non invasive detection of peptic ulcer	2023	10.1177/09544119231184111	PUBMED	off topic	no
aiding the detection of qrs complex in ecg signals by detecting s peaks independently	2018	10.1007/s13239-018-0355-0	PUBMED	off topic	no
alexander fractional differential window filter for ecg denoising	2018	10.1007/s13246-018-0642-y	PUBMED	off topic	no
algorithm for mobile platform-based real-time qrs detection	2023	10.3390/s23031625	PUBMED	missing dataset information	yes
amyloid burden correlates with electrocardiographic findings in patients with cardiac amyloidosis-insights from histology and cardiac magnetic resonance imaging	2024	10.3390/jcm13020368	PUBMED	off topic	no
amyloid transthyretin cardiomyopathy in elderly patients with aortic stenosis undergoing transcatheter aortic valve implantation	2023	10.1161/jaha.123.030271	PUBMED	off topic	no
an accurate qrs complex and p wave detection in ecg signals using complete ensemble empirical mode decomposition approach	2019	10.1109/access.2019.2939943	PUBMED	duplicate	no
an adaptive median filter based on sampling rate for r-peak detection and major-arrhythmia analysis	2020	10.3390/s20216144	PUBMED	denoising	yes
an adaptive qrs detection algorithm for ultra-long-term ecg recordings	2020	10.1016/j.jelectrocard.2020.02.016	PUBMED	missing technical details	yes
an analysis method for wearable electrocardiogram measurement based on non-orthogonal complex wavelet expansion	2017	10.1109/embc.2017.8037726	PUBMED	inaccessible dataset	no
an analysis of the effects of noisy electrocardiogram signal on heartbeat detection performance	2020	10.3390/bioengineering7020053	PUBMED	duplicate	no
an arrhythmia classification approach via deep learning using single-lead ecg without qrs wave detection	2024	10.1016/j.heliyon.2024.e27200	PUBMED	duplicate	no
an artifact-resistant feature sknaer for quantifying the burst of skin sympathetic nerve activity signal	2022	10.3390/bios12050355	PUBMED	off topic	no
an automatic r and t peak detection method based on the combination of hierarchical clustering and discrete wavelet transform	2020	10.1109/jbhi.2020.2973982	PUBMED	duplicate	no

an autopsy case of sudden unexpected death of a young adult with progressive intraventricular conduction delay	2022	10.1016/ j.prp.2022.154226	PUBMED	off topic	no
an ecg delineation and arrhythmia classification system using slope variation measurement by ternary second-order delta modulators for wearable ecg sensors	2021	10.1109/ tbcas.2021.3113665	PUBMED	duplicate	no
an efficient and robust deep learning method with 1-d octave convolution to extract fetal electrocardiogram	2020	10.3390/s20133757	PUBMED	duplicate	no
an efficient and robust digital fractional order differentiator based ecg pre-processor design for qrs detection	2019	10.1109/ tbcas.2019.2916676	PUBMED	duplicate	no
an efficient teager energy operator-based automated qrs complex detection	2018	10.1155/2018/8360475	PUBMED	missing technical details	yes
an enhanced t-wave delineation method using phasor transform in the electrocardiogram	2021	10.1088/2057-1976/ac0502	PUBMED	duplicate	no
an improved real-time r-wave detection efficient algorithm in exercise ecg signal analysis	2020	10.1155/2020/8868685	PUBMED	multichannel	yes
an improved sliding window area method for t wave detection	2019	10.1155/2019/3130527	PUBMED	off topic	no
an innovative method based on shannon energy envelope and summit navigation for detecting r peaks of noise stress test signals	2021	10.1016/ j.jelectrocard.2020.12.012	PUBMED	does not introduce a new technique	yes
an intriguing case of regular rr and qrs alternans during idiopathic left posterior fascicle ventricular tachycardia	2020	10.1016/ j.jelectrocard.2020.07.022	PUBMED	off topic	no
an objective tool for quantifying atrial fibrillation substrate in rats	2023	10.1152/ ajpheart.00728.2022	PUBMED	duplicate	no
analysis of cardiac vibration signals acquired from a novel implant placed on the gastric fundus	2021	10.3389/ fphys.2021.748367	PUBMED	off topic	no
analysis of normal electrocardiographic patterns in mandarin ducks (aix galericulata)	2021	10.5455/ ovj.2021.v11.i4.29	PUBMED	animal	no
analysis of screening electrocardiogram for the subcutaneous defibrillator in adults with congenital heart disease	2017	10.1007/s00246-017-1635-4	PUBMED	off topic	no
analysis of the determining factors of detectable p-wave and amplitude of qrs complex sensed by implantable loop recorder	2021	10.1002/joa3.12582	PUBMED	off topic	no

analyzing long-term electrocardiography recordings to detect arrhythmias in mice	2021	10.3791/62386	PUBMED	duplicate	no
annotated real and synthetic datasets for non-invasive foetal electrocardiography post-processing benchmarking	2020	10.1016/ j.dib.2020.106399	PUBMED	off topic	no
appearance of qrs fragmentation late after mustard/senning repair is associated with adverse outcome	2017	10.1136/ heartjnl-2016-310512	PUBMED	off topic	no
approxbiowear: approximating additions for efficient biomedical wearable computing at the edge	2021	10.1109/ embc46164.2021.9630165	PUBMED	duplicate	no
are tp-e interval and qt dispersion values important in children with coeliac disease?	2023	10.1017/ s1047951122003213	PUBMED	off topic	no
area efficient folded undecimator based ecg detector	2021	10.1038/s41598-021-82231-2	PUBMED	off topic	no
armband sensors location assessment for left arm-ecg bipolar leads waveform components discovery tendencies around the muac line	2022	10.3390/s22197240	PUBMED	off topic	no
arrhythmia detection using deep convolutional neural network with long duration ecg signals	2018	10.1016/ j.compbiomed.2018.09.009	PUBMED	off topic	no
arrhythmia recognition and classification using ecg morphology and segment feature analysis	2019	10.1109/ tcbb.2018.2846611	PUBMED	does not introduce a new technique	no
arrhythmia spectrum and outcome in children with myocarditis	2021	10.4103/ apc.apc_207_20	PUBMED	off topic	no
artificial intelligence assessment for early detection and prediction of renal impairment using electrocardiography	2022	10.1007/s11255-022-03165-w	PUBMED	duplicate	no
artificial intelligence assessment for early detection of heart failure with preserved ejection fraction based on electrocardiographic features	2020	10.1093/ehjdh/ ztaa015	PUBMED	off topic	no
artificial intelligence for detecting electrolyte imbalance using electrocardiography	2021	10.1111/anec.12839	PUBMED	off topic	no
artificial intelligence for detecting mitral regurgitation using electrocardiography	2020	10.1016/ j.jelectrocard.2020.02.008	PUBMED	off topic	no

artificial intelligence for early prediction of pulmonary hypertension using electrocardiography	2020	10.1016/ j.healun.2020.04.009	PUBMED	off topic	no
artificial intelligence-based electrocardiographic biomarker for outcome prediction in patients with acute heart failure: prospective cohort study	2024	10.2196/52139	PUBMED	duplicate	no
artificial intelligence-based identification of left ventricular systolic dysfunction from 12-lead electrocardiograms: external validation and advanced application of an existing model	2023	10.1093/ehjdh/ ztad081	PUBMED	duplicate	no
assessing cardiac safety among clients receiving methadone as part of opioid agonist maintenance therapy (oamt) in durban, south africa	2023	10.1080/10550887.2 022.2063640	PUBMED	off topic	no
assessment of automatic strategies for combining qrs detections by multiple algorithms in multiple leads	2019	10.1088/1361- 6579/ab553a	PUBMED	multichannel	no
assessment of electrical dyssynchrony in cardiac resynchronization therapy: 12-lead electrocardiogram vs. 96-lead body surface map	2023	10.1093/europace/ euac159	PUBMED	off topic	no
association between complete right bundle branch block and atrial fibrillation development	2022	10.1111/anec.12966	PUBMED	off topic	no
association between fragmented qrs complexes and left-ventricular dysfunction in anabolic androgenic steroid users	2020	10.1080/00015385.2 019.1682339	PUBMED	off topic	no
association between left bundle branch block and ventricular septal mid-wall fibrosis in patients with preserved left ventricular ejection fraction	2024	10.1016/ j.jelectrocard.2024.0 1.002	PUBMED	off topic	no
association between t wave morphology parameters and abnormal cardiac spect imaging	2022	10.1016/ j.jelectrocard.2021.1 2.003	PUBMED	off topic	no
association between urinary cadmium and qrs|t angle among adults in the united states	2018	10.1097/ jom.0000000000001 382	PUBMED	off topic	no
association between uterine leiomyoma and fragmented qrs waves: a prospective case-control study	2024	10.1590/1806- 9282.20231359	PUBMED	off topic	no

association of ecg parameters with late gadolinium enhancement and outcome in patients with clinical suspicion of acute or subacute myocarditis referred for cmr imaging	2020	10.1371/ journal.pone.0227134	PUBMED	off topic	no
association of fragmented qrs with left atrial scarring in patients with persistent atrial fibrillation undergoing radiofrequency catheter ablation	2020	10.1016/ j.hrthm.2019.09.010	PUBMED	off topic	no
association of frequent premature ventricular complex >10% and stroke-like symptoms without a prior diagnosis of stroke or transient ischemic attack	2018	10.1016/ j.ijcha.2018.05.001	PUBMED	off topic	no
associations of cardiac ventricular repolarization with serum adhesion molecules and cognitive function in older adults: the mind-china study	2023	10.3233/jad-220874	PUBMED	off topic	no
asymptomatic idiopathic belhassen ventricular tachycardia in a neonate detected using 'smart sock' wearable smartphone-enabled cardiac monitoring	2020	10.12659/ ajcr.921092	PUBMED	off topic	no
atrial fibrillation detection on compressed sensed ecg	2017	10.1088/1361-6579/aa7652	PUBMED	off topic	no
atrial fibrillation detection with an analog smartwatch: prospective clinical study and algorithm validation	2022	10.2196/37280	PUBMED	duplicate	no
atrial fibrillation detection with signal decomposition and dilated residual neural network	2023	10.1088/1361-6579/acfa61	PUBMED	off topic	no
augmentation of electrocardiographic qrs r-amplitude precedes radiocontrast-induced hypotension during mobile computed tomography scanning	2019	10.3390/jcm8040505	PUBMED	off topic	no
autodetection of j wave based on random forest with synchrosqueezed wavelet transform	2018	10.1155/2018/1315357	PUBMED	off topic	no
automated detection of premature ventricular contraction in ecg signals using enhanced template matching algorithm	2020	10.1088/2057-1976/ab6995	PUBMED	off topic	no
automated diagnosis of atrial fibrillation using ecg component-aware transformer	2022	10.1016/ j.compbiomed.2022.106115	PUBMED	off topic	no
automated electrocardiogram signal quality assessment based on fourier analysis and template matching	2023	10.1007/s10877-022-00948-5	PUBMED	off topic	no

automated interpretable detection of myocardial infarction fusing energy entropy and morphological features	2019	10.1016/ j.cmpb.2019.03.012	PUBMED	duplicate	no
automated quantification of abnormal qrs peaks from high-resolution ecgs predicts late ventricular arrhythmias in hypertrophic cardiomyopathy: a 5-year prospective multicenter study	2022	10.1161/ jaha.122.026025	PUBMED	off topic	no
automatic detection of arrhythmia from imbalanced ecg database using cnn model with smote	2019	10.1007/s13246-019-00815-9	PUBMED	duplicate	no
automatic detection of qrs complexes using dual channels based on u-net and bidirectional long short-term memory	2021	10.1109/ jbhi.2020.3018563	PUBMED	duplicate	no
automatic detection of short-term atrial fibrillation segments based on frequency slice wavelet transform and machine learning techniques	2021	10.3390/s21165302	PUBMED	off topic	no
automatic diagnosis of strict left bundle branch block using a wavelet-based approach	2019	10.1371/ journal.pone.0212971	PUBMED	off topic	no
automatic digital ecg signal extraction and normal qrs recognition from real scene ecg images	2020	10.1016/ j.cmpb.2019.105254	PUBMED	off topic	no
automatic qrs complex detection using two-level convolutional neural network	2018	10.1186/s12938-018-0441-4	PUBMED	INCLUDED	yes
automatic signal quality assessment of raw trans-abdominal biopotential recordings for non-invasive fetal electrocardiography	2023	10.3389/ fbioe.2023.1059119	PUBMED	fetal	no
automatically computed ecg algorithm for the quantification of myocardial scar and the prediction of mortality	2018	10.1007/s00392-018-1253-z	PUBMED	off topic	no
beat-level interpretation of intra-patient paradigm based on object detection	2022	10.3389/ fcvm.2022.857019	PUBMED	off topic	no
benchmarking open-source algorithms for qrs detection and rri editing in textile electrocardiography	2024	10.1109/ embc53108.2024.10782192	PUBMED	off topic	no
biomarkers of pre-existing risk of torsade de pointes under sotalol treatment	2020	10.1016/ j.jelectrocard.2020.04.011	PUBMED	off topic	no
blunt cardiac injury due to trauma associated with snowboarding: a case report	2017	10.1186/s13256-017-1242-2	PUBMED	off topic	no

body surface potential mapping detects early disease onset in plakophilin-2-pathogenic variant carriers	2023	10.1093/europace/euad197	PUBMED	duplicate	no
bradyarrhythmias detected by extended rhythm recording in patients undergoing transcatheter aortic valve replacement (brady-tavr study)	2022	10.1016/j.hrthm.2021.11.020	PUBMED	off topic	no
broadband electrocardiogram acquisition for improved suppression of mri gradient artifacts	2020	10.1088/1361-6579/ab7b8e	PUBMED	off topic	no
can heart rate variability parameters derived by a heart rate monitor differentiate between atrial fibrillation and sinus rhythm?	2018	10.1186/s12917-018-1650-6	PUBMED	off topic	no
cardiac effects of 5f-cumyl-pegaclone	2024	10.1007/s00414-023-03146-3	PUBMED	off topic	no
cardiac electrical dyssynchrony is accurately detected by noninvasive electrocardiographic imaging	2018	10.1016/j.hrthm.2018.02.024	PUBMED	off topic	no
cardiac muscle-restricted partial loss of nos1ap expression has limited but significant impact on electrocardiographic features	2023	10.1093/g3journal/jkad208	PUBMED	off topic	no
cardiac resynchronization therapy reduces ventricular arrhythmias in primary but not secondary prophylactic implantable cardioverter defibrillator patients: insight from the resynchronization in ambulatory heart failure trial	2017	10.1161/circep.116.004875	PUBMED	off topic	no
cardioprotective role of gts-21 by attenuating the tlr4/nf-κb pathway in streptozotocin-induced diabetic cardiomyopathy in rats	2021	10.1007/s00210-020-01957-4	PUBMED	off topic	no
case report: efficacy analysis of radiofrequency catheter ablation combined with atrial appendage resection for atrial tachycardia originating from the atrial appendage in children	2022	10.3389/fcvm.2022.990325	PUBMED	off topic	no
case report: left bundle branch pacing guided by real-time monitoring of current of injury and electrocardiography	2022	10.3389/fcvm.2022.1025620	PUBMED	off topic	no
catheter ablation of premature ventricular contractions originating from aortic sinus cusps in a patient with dextrocardia and situs solitus: a case report	2017	10.1097/md.0000000000008947	PUBMED	off topic	no
changes in paced signals may predict in-hospital cardiac arrest	2018	10.1111/pace.13223	PUBMED	off topic	no

characteristic electrocardiography findings of covid-19 patients	2021	10.34172/aim.2021.103	PUBMED	off topic	no
characterization of a high throughput human stem cell cardiomyocyte assay to predict drug-induced changes in clinical electrocardiogram parameters	2021	10.1016/j.ejphar.2021.174584	PUBMED	off topic	no
characterization of microminipigs as an in vivo experimental model for cardiac safety pharmacology	2017	10.1016/j.jphs.2017.02.002	PUBMED	off topic	no
characterization of rheumatic heart disease from electrocardiogram recordings	2023	10.1088/1361-6579/aca6cb	PUBMED	off topic	no
characterization of the electrocardiogram of microminipig in comparison with that of clawn miniature swine: impacts of miniaturization of body size on electrocardiographic indices	2023	10.1292/jvms.23-0049	PUBMED	off topic	no
characterizing the location and extent of myocardial infarctions with inverse ecg modeling and spatiotemporal regularization	2018	10.1109/jbhi.2017.2768534	PUBMED	off topic	no
characterizing the transient electrocardiographic signature of ischemic stress using laplacian eigenmaps for dimensionality reduction	2020	10.1016/j.compbiomed.2020.104059	PUBMED	duplicate	no
chronic volume overload caused by abdominal aorto-venocaval shunt provides arrhythmogenic substrates in the rat atrium	2022	10.1248/bpb.b22-00031	PUBMED	off topic	no
cineecg for visualization of changes in ventricular electrical activity during ischemia	2024	10.1016/j.jelectrocard.2024.01.007	PUBMED	duplicate	no
cineecg: a novel method to image the average activation sequence in the heart from the 12-lead ecg	2022	10.1016/j.compbiomed.2021.105128	PUBMED	duplicate	no
classical heart rate variability and nonlinear heart rate analysis in mice under napentobarbital and ketamine/xylazine anesthesia	2022	10.55730/1300-0144.5383	PUBMED	animal	no
classification of ischemic and non-ischemic cardiac events in holter recordings based on the continuous wavelet transform	2020	10.1007/s11517-020-02134-8	PUBMED	duplicate	no
classification of qrs complexes to detect premature ventricular contraction using machine learning techniques	2022	10.1371/journal.pone.0268555	PUBMED	duplicate	no

clinical correlates and outcome of the patterns of premature ventricular beats in olympic athletes: a long-term follow-up study	2021	10.1177/2047487320928452	PUBMED	off topic	no
clinical significance of 12 lead ecg changes in patients undergoing pericardiocentesis for cardiac tamponade	2021	10.1080/00015385.2019.1700336	PUBMED	off topic	no
clinical significance of myocardial scar in patients with frequent premature ventricular complexes undergoing catheter ablation	2021	10.1016/ j.hrthm.2020.07.030	PUBMED	off topic	no
clinical utility of the electrocardiographic p-wave axis in patients with chronic obstructive pulmonary disease	2022	10.1159/000519668	PUBMED	off topic	no
clinical validation and evaluation of a novel six-lead handheld electrocardiogram recorder compared to the 12-lead electrocardiogram in unselected cardiology patients (evalecg cardio)	2021	10.1093/ehjdh/ ztab083	PUBMED	off topic	no
clinical validation of a smartphone-based handheld ecg device: a validation study	2022	10.1097/ hpc.0000000000000303	PUBMED	off topic	no
clinical validation of automated corrected qt-interval measurements from a single lead electrocardiogram using a novel smartwatch	2022	10.3389/ fcvm.2022.906079	PUBMED	off topic	no
cnn-based two step r peak detection method: combining segmentation and regression	2022	10.1109/ embc48229.2022.9871227	PUBMED	duplicate	no
combined cardiac and respiratory monitoring from a single signal: a case study employing the fantasia database	2023	10.3390/s23177401	PUBMED	duplicate	no
comparative study on heart rate variability analysis for atrial fibrillation detection in short single-lead ecg recordings	2018	10.1109/ embc.2018.8512345	PUBMED	duplicate	no
comparing different methods of hand-crafted hrv, edr and cpc features for sleep apnoea detection	2019	10.1109/ embc.2019.8856779	PUBMED	duplicate	no
comparison between smartphone electrocardiography and standard three-lead base apex electrocardiography in healthy horses	2020	10.1136/vr.105759	PUBMED	animal	no
comparison of different electrocardiography with vectorcardiography transformations	2019	10.3390/s19143072	PUBMED	off topic	no

comparison of electrocardiograms (ecg) waveforms and centralized ecg measurements between a simple 6-lead mobile ecg device and a standard 12-lead ecg	2021	10.1111/anec.12872	PUBMED	multichannel	no
comparison of single- and multi-reference qrd-rls adaptive filter for non-invasive fetal electrocardiography	2019	10.1109/embc.2019.8856824	PUBMED	fetal	no
comparison of the integral indices of the vectorcardiogram with the data of echocardiography in patients with idiopathic and chronic thromboembolic pulmonary hypertension	2019	10.26442/00403660.2019.03.000043	PUBMED	off topic	no
comparison of three ecg machines for electrocardiography in green iguanas (iguana iguana)	2021	10.17221/39/2020-vetmed	PUBMED	animal	no
complete right bundle branch block and qrs-t discordance can be the initial clue to detect s-icd ineligibility	2017	10.1016/j.jjcc.2016.11.014	PUBMED	duplicate	no
contactless capacitive electrocardiography using hybrid flexible printed electrodes	2020	10.3390/s20185156	PUBMED	off topic	no
continuous ecg monitoring in patients with acute coronary syndrome or heart failure: easi versus gold standard	2018	10.1177/1054773817704653	PUBMED	off topic	no
continuous heart rate monitoring for automatic detection of life-threatening arrhythmias with novel bio-sensing technology	2021	10.3389/fcvm.2021.707621	PUBMED	off topic	no
coronary sinus lead delay index for optimization of coronary sinus lead placement	2018	10.1111/anec.12454	PUBMED	off topic	no
correction of the qrs duration for heart rate	2019	10.1016/j.jelectrocard.2019.02.005	PUBMED	off topic	no
correlation between fragmented qrs and ventricular function from cardiac magnetic resonance in patients with repaired tetralogy of fallot	2021	10.1007/s00246-021-02655-5	PUBMED	off topic	no
correlation of qrs duration with myocardial blush grade as a marker of myocardial reperfusion in primary percutaneous coronary intervention	2018	10.1016/j.ihj.2018.10.412	PUBMED	off topic	no
covid-19 survivors may exhibit deterioration in frontal plane qrs-t angle and other electrocardiogram parameters	2022	10.26355/eurrev_202209_29791	PUBMED	off topic	no

crispr -mediated expression of the fetal scn5a isoform in adult mice causes conduction defects and arrhythmias	2018	10.1161/ jaha.118.010393	PUBMED	fetal	no
cryoballoon ablation induced hyperkalemia due to possible cold agglutinin disease	2019	10.2169/ internalmedicine.2888-19	PUBMED	off topic	no
current clinical practice of cardiac resynchronization therapy in turkey: reflections from cardiac resynchronization therapy survey-ii	2020	10.14744/ anatoljcardiol.2020.02680	PUBMED	duplicate	no
dapagliflozin improves diabetic cardiomyopathy by modulating the akt/mtor signaling pathway	2022	10.1155/2022/9687345	PUBMED	off topic	no
daphnetin preconditioning decreases cardiac injury and susceptibility to ventricular arrhythmia following ischaemia-reperfusion through the tlr4/myd88/nf-κb signalling pathway	2021	10.1159/000513631	PUBMED	off topic	no
data-driven separation and estimation of atrial dynamics in very high-dimensional electrocardiograms from epilepsy patients	2019	10.1109/ embc.2019.8856494	PUBMED	off topic	no
decreased cardiac reserve in asymptomatic patients after arterial switch operation for transposition of the great arteries	2023	10.1016/ j.ijcard.2023.131153	PUBMED	off topic	no
deep learning in the cross-time frequency domain for sleep staging from a single-lead electrocardiogram	2018	10.1088/1361-6579/aaf339	PUBMED	off topic	no
deep learning model to detect significant aortic regurgitation using electrocardiography	2022	10.1016/ j.jjcc.2021.08.029	PUBMED	duplicate	no
deep learning techniques in the classification of ecg signals using r-peak detection based on the ptb-xl dataset	2021	10.3390/s21248174	PUBMED	off topic	no
deep learning-based long-term risk evaluation of incident type 2 diabetes using electrocardiogram in a non-diabetic population: a retrospective, multicentre study	2024	10.1016/ j.eclinm.2024.102445	PUBMED	off topic	no
deep learning-mediated prediction of concealed accessory pathway based on sinus rhythmic electrocardiograms	2023	10.1111/anec.13072	PUBMED	duplicate	no
deep-learning model for screening sepsis using electrocardiography	2021	10.1186/s13049-021-00953-8	PUBMED	off topic	no

deep-learning-based estimation of the spatial qrs-t angle from reduced-lead ecgs	2022	10.3390/s22145414	PUBMED	off topic	no
defining electrocardiographic criteria to differentiate non-type 1 brugada ecg variants from normal incomplete rbbb patterns in the young scd-sos cohort	2022	10.1111/jce.15615	PUBMED	off topic	no
delayed ventricular repolarization and sodium channel current modification in a mouse model of rett syndrome	2022	10.3390/ ijms23105735	PUBMED	off topic	no
delineation of 12-lead ecg representative beats using convolutional encoder-decoders with residual and recurrent connections	2024	10.3390/s24144645	PUBMED	multichannel	no
delineation of the electrocardiogram with a mixed-quality-annotations dataset using convolutional neural networks	2021	10.1038/s41598-020-79512-7	PUBMED	off topic	no
denoising wearable armband ecg data using the variable frequency complex demodulation technique	2020	10.1109/ embc44109.2020.9175665	PUBMED	off topic	no
design of a biorthogonal wavelet transform based r-peak detection and data compression scheme for implantable cardiac pacemaker systems	2018	10.1007/s10916-018-0953-2	PUBMED	duplicate	no
design of high performance qrs complex detector for wearable healthcare devices using biorthogonal spline wavelet transform	2018	10.1016/ j.isatra.2018.08.002	PUBMED	missing technical details	yes
designing an optimum and reduced order filter for efficient ecg qrs peak detection and classification of arrhythmia data	2021	10.1155/2021/6542290	PUBMED	inaccessible dataset	no
detecting hypoglycemia-induced electrocardiogram changes in a rodent model of type 1 diabetes using shape-based clustering	2023	10.1371/ journal.pone.0284622	PUBMED	off topic	no
detecting ischemic stress to the myocardium using laplacian eigenmaps and changes to conduction velocity	2017	10.22489/ cinc.2017.269-417	PUBMED	duplicate	no
detection and classification of atrial and ventricular cardiovascular diseases to improve the cardiac health literacy for resource constrained regions	2023	10.1049/htl2.12043	PUBMED	off topic	no
detection and classification of myocardial infarction with support vector machine classifier using grasshopper optimization algorithm	2021	10.4103/ jmss.jmss_24_20	PUBMED	duplicate	no

detection of abnormal left ventricular geometry in patients without cardiovascular disease through machine learning: an ecg-based approach	2021	10.1111/jch.14200	PUBMED	duplicate	no
detection of acute myocardial infarction in a pig model using the san-atrial-avn-his (saah) electrocardiogram (ecg), model phs-a10, an automated and integrated signals recognition system	2018	10.12659/msm.905961	PUBMED	off topic	no
detection of apnea bradycardia from ecg signals of preterm infants using layered hidden markov model	2021	10.1007/s10439-021-02732-z	PUBMED	duplicate	no
detection of arrhythmogenic substrate within qrs complex in patients with cardiac sarcoidosis using wavelet-transformed ecg	2020	10.1007/s00380-020-01584-2	PUBMED	off topic	no
detection of atrial fibrillation episodes based on 3d algebraic relationships between cardiac intervals	2022	10.3390/diagnostics12122919	PUBMED	duplicate	no
detection of atrial fibrillation from rr intervals and pqrst morphology using a neural network ensemble	2018	10.1109/embc.2018.8513496	PUBMED	off topic	no
detection of cardiac involvement in pulmonary sarcoidosis using high-resolution holter electrocardiogram	2021	10.1002/joa3.12501	PUBMED	off topic	no
detection of myocardial infarction from multi-lead ecg using dual-q tunable q-factor wavelet transform	2019	10.1109/embc.2019.8857775	PUBMED	multichannel	no
detection of the complete ecg waveform with woven textile electrodes	2021	10.3390/bios11090331	PUBMED	off topic	no
development and validation of a deep-learning model to detect crp level from the electrocardiogram	2022	10.3389/fphys.2022.864747	PUBMED	duplicate	no
development and validation of a prediction model and score for transthyretin cardiac amyloidosis diagnosis: t-amylo	2023	10.1016/j.jcmg.2023.05.002	PUBMED	off topic	no
development and validation of an algorithm for the digitization of ecg paper images	2022	10.3390/s22197138	PUBMED	duplicate	no
development of robust, fast and efficient qrs complex detector: a methodological review	2018	10.1007/s13246-018-0670-7	PUBMED	review	no
diagnosis of atrial fibrillation based on ai-detected anomalies of ecg segments	2023	10.1016/j.heliyon.2023.e23597	PUBMED	off topic	no

diagnostic ability of peguero-lo presti electrocardiographic left ventricular hypertrophy criterion in severe aortic stenosis	2021	10.3390/ jcm10132864	PUBMED	off topic	no
diagnostic accuracy of computer aided electrocardiogram analysis in dogs	2021	10.1111/jsap.13267	PUBMED	animal	no
diagnostic accuracy of high sensitivity troponin and association of electrocardiogram findings for mortality in syncope patients: a retrospective cohort study	2023	10.1097/ md.0000000000034 064	PUBMED	off topic	no
diagnostic accuracy of the electrocardiographic decision support - myocardial ischaemia (eds-mi) algorithm in detection of acute coronary occlusion	2020	10.1177/204887261 8768081	PUBMED	off topic	no
diagnostic and prognostic value of low qrs voltages in cardiomyopathies: old but gold	2022	10.1093/eurjpc/ zwaa027	PUBMED	off topic	no
diagnostic interpretation of non-uniformly sampled electrocardiogram	2021	10.3390/s21092969	PUBMED	off topic	no
diagnostic value of qrs and s wave variation in patients with suspicion of acute pulmonary embolism	2018	10.1016/ j.ajem.2018.03.074	PUBMED	off topic	no
diaphragmatic cmap monitoring during cryoballoon procedures: surface vs. hepatic recording comparison and limitations of this approach	2022	10.3389/ fcvm.2022.814026	PUBMED	off topic	no
differential genome-wide associated variants and enriched pathways of ecg parameters among people with versus without hiv	2023	10.1097/ qad.0000000000003 647	PUBMED	off topic	no
diffuse myocardial fibrosis reduces electrocardiographic voltage measures of left ventricular hypertrophy independent of left ventricular mass	2017	10.1161/ jaha.116.003795	PUBMED	off topic	no
diphenhydramine overdose detected early by integration of toxidrome and electrocardiography and treated with venoarterial extracorporeal membrane oxygenation: a case report	2023	10.1177/030006052 31205449	PUBMED	off topic	no
discovering and visualizing disease-specific electrocardiogram features using deep learning: proof-of-concept in phospholamban gene mutation carriers	2021	10.1161/ circep.120.009056	PUBMED	off topic	no
distinct features of probands with early repolarization and brugada syndromes carrying scn5a pathogenic variants	2021	10.1016/ j.jacc.2021.08.024	PUBMED	off topic	no

do we need new electrocardiographic criteria for left ventricular hypertrophy? the case of the peguero-lo presti criterion. a narrative review	2024	10.1093/ajh/hpad117	PUBMED	off topic	no
drug-induced qt prolongation: concordance of preclinical anesthetized canine model in relation to published clinical observations for ten cipa drugs	2020	10.1016/j.vascn.2020.106871	PUBMED	off topic	no
dual-chamber icd for left bundle branch area pacing: the cardiac resynchronization and arrhythmia sensing via the left bundle (cross-left) pilot study	2023	10.1007/s10840-022-01342-6	PUBMED	off topic	no
dynamic changes in the signal-averaged electrocardiogram are associated with the long-term outcomes after ablation of ischemic ventricular tachycardia	2021	10.1007/s10840-020-00708-y	PUBMED	duplicate	no
dyspnoe, cough and fever in 80-year old patient with chronic heart failure	2019		PUBMED	off topic	no
early detection of iron overload cardiomyopathy in transfusion dependent thalassemia patients in sulaimaniyah city, iraq	2022	10.2147/tcrm.s354574	PUBMED	off topic	no
early detection of low qrs voltage and its association with mortality in patients with sepsis	2024	10.1038/s41598-024-66612-x	PUBMED	off topic	no
early detection of myocardial ischemia in resting ecg: analysis by hht	2023	10.1186/s12938-023-01089-9	PUBMED	duplicate	no
early detection of organ involvement in fabry disease by biomarker assessment in conjunction with lge cardiac mri: results from the sophia study	2019	10.1016/j.ymgme.2018.11.005	PUBMED	off topic	no
early identification of severe immune checkpoint inhibitor associated myocarditis: from an electrocardiographic perspective	2024	10.1002/cam4.7460	PUBMED	off topic	no
early repolarization pattern on ecg recorded before the acute coronary event does not predict ventricular fibrillation during st-elevation myocardial infarction	2020	10.1016/j.hrthm.2019.11.011	PUBMED	off topic	no
ecg alarms during left ventricular assist device (lvad) therapy in the icu	2021	10.1016/j.hrtlng.2021.03.080	PUBMED	off topic	no
ecg beat classification using empirical mode decomposition and mixture of features	2017	10.1080/03091902.2017.1394386	PUBMED	duplicate	no
ecg changes during adult life in fabry disease: results from a large longitudinal cohort study	2023	10.3390/diagnostics13030354	PUBMED	duplicate	no

ecg classification using orthogonal matching pursuit and machine learning	2022	10.3390/s22134960	PUBMED	multichannel	no
ecg delineation using a piecewise gaussian derivative model with parameters estimated from scale-dependent algebraic expressions	2019	10.1109/embc.2019.8856523	PUBMED	off topic	no
ecg dry-electrode 3d printing and signal quality considerations	2021	10.1109/embc46164.2021.9630599	PUBMED	duplicate	no
ecg enhancement and r-peak detection based on window variability	2021	10.3390/healthcare9020227	PUBMED	INCLUDED	yes
ecg features and methods for automatic classification of ventricular premature and ischemic heartbeats: a comprehensive experimental study	2017	10.1038/s41598-017-10942-6	PUBMED	off topic	no
ecg in left ventricular hypertrophy: a change in paradigm from assessing left ventricular mass to its electrophysiological properties	2022	10.1016/j.jelectrocard.2022.06.002	PUBMED	off topic	no
ecg in the clinical and prognostic evaluation of patients with pulmonary arterial hypertension: an underestimated value	2022	10.1177/17534666221087846	PUBMED	duplicate	no
ecg indices poorly predict left ventricular hypertrophy and are applicable only in individuals with low cardiovascular risk	2020	10.3390/jcm9051364	PUBMED	duplicate	no
ecg language processing (elp): a new technique to analyze ecg signals	2021	10.1016/j.cmpb.2021.105959	PUBMED	off topic	no
ecg marker evaluation for the machine-learning-based classification of acute and chronic phases of trypanosoma cruzi infection in a murine model	2023	10.3390/tropicalmed8030157	PUBMED	duplicate	no
ecg multilead qt interval estimation using support vector machines	2019	10.1155/2019/6371871	PUBMED	multichannel	no
ecg performance in simultaneous recordings of five wearable devices using a new morphological noise-to-signal index and smith-waterman-based rr interval comparisons	2022	10.1371/journal.pone.0274994	PUBMED	duplicate	no
ecg segmentation algorithm based on bidirectional hidden semi-markov model	2022	10.1016/j.compbiomed.2022.106081	PUBMED	multichannel	yes
ecg signal analysis using modified s-transform	2017	10.1049/htl.2016.0078	PUBMED	multichannel	yes

ecg signal classification for the detection of cardiac arrhythmias using a convolutional recurrent neural network	2018	10.1088/1361-6579/aad9ed	PUBMED	duplicate	no
ecg signal denoising and features extraction using unbiased fir smoothing	2019	10.1155/2019/2608547	PUBMED	duplicate	no
ecg_segnet: an ecg delineation model based on the encoder-decoder structure	2022	10.1016/j.compbiomed.2022.105445	PUBMED	duplicate	no
ecg-based biometric recognition without qrs segmentation: a deep learning-based approach	2021	10.1109/embc46164.2021.9630899	PUBMED	duplicate	no
ecg-based identity validation during bathing in different water temperature(*)	2020	10.1109/embc44109.2020.9176107	PUBMED	off topic	no
ecg-based score estimates the probability to detect fabry disease cardiac involvement	2021	10.1016/j.ijcard.2021.07.022	PUBMED	duplicate	no
ecg-derived respiration based on iterated hilbert transform and hilbert vibration decomposition	2018	10.1007/s13246-018-0640-0	PUBMED	off topic	no
effect of bone marrow-derived mesenchymal stem cells on ischaemic-reperfused hearts in adult rats with established chronic kidney disease	2019	10.15283/ijsc18114	PUBMED	duplicate	no
effect of diurnal variations in the qrs complex and t waves on the eligibility for subcutaneous implantable cardioverter-defibrillators	2019	10.1016/j.hrthm.2019.01.004	PUBMED	off topic	no
effect of hunger strike on electrocardiographic parameters	2019	10.5543/tkda.2018.57794	PUBMED	off topic	no
effect of increased left ventricle mass on ischemia assessment in electrocardiographic signals: rabbit isolated heart study	2017	10.1186/s12872-017-0652-9	PUBMED	off topic	no
effect of the recording condition on the quality of a single-lead electrocardiogram	2022	10.1007/s00380-021-01991-z	PUBMED	off topic	no
effects of baroreflex activation therapy on cardiac function and morphology	2024	10.1002/ehf2.14940	PUBMED	off topic	no
effects of different dosages esketamine on cardiac conduction and heterogeneity of cx43: the epicardial mapping in guinea pigs	2022	10.21037/atm-22-2614	PUBMED	off topic	no

effects of propofol on ventricular repolarization and incidence of malignant arrhythmias in adults	2018	10.1016/ j.jelectrocard.2017.1 1.003	PUBMED	off topic	no
efficient ecg compression and qrs detection for e-health applications	2017	10.1038/s41598-017-00540-x	PUBMED	does not introduce a new technique	no
efficient fiducial point detection of ecg qrs complex based on polygonal approximation	2018	10.3390/s18124502	PUBMED	off topic	no
efficient qrs complex detection algorithm based on fast fourier transform	2018	10.1007/s13534-018-0087-y	PUBMED	denoising	no
electrical and mechanical dyssynchrony in patients with right bundle branch block	2020	10.1007/s12350-018-1418-1	PUBMED	off topic	no
electrical approach to improve left ventricular activation during right ventricle stimulation	2017		PUBMED	off topic	no
electrocardiogram analysis in anderson-fabry disease: a valuable tool for progressive phenotypic expression tracking	2023	10.3389/ fcvm.2023.1184361	PUBMED	off topic	no
electrocardiogram and phonocardiogram monitoring system for cardiac auscultation	2019	10.1109/ tbcas.2019.2947694	PUBMED	off topic	no
electrocardiogram characteristics and prognostic value in light-chain amyloidosis: a comparison with cardiac magnetic resonance imaging	2021	10.3389/ fcvm.2021.751422	PUBMED	off topic	no
electrocardiogram derived respiratory rate using a wearable armband	2021	10.1109/ tbme.2020.3004730	PUBMED	off topic	no
electrocardiogram fiducial point detector using a bilateral filter and symmetrical point-filter structure	2021	10.3390/ ijerph182010792	PUBMED	off topic	no
electrocardiogram machine learning for detection of cardiovascular disease in african americans: the jackson heart study	2021	10.1093/ehjdh/ ztab003	PUBMED	off topic	no
electrocardiogram signal classification in the diagnosis of heart disease based on rbf neural network	2022	10.1155/2022/92512 25	PUBMED	duplicate	no
electrocardiographic abnormalities are frequently detected in healthy adult borzoi with a normal echocardiogram	2024	10.2460/ javma.24.04.0247	PUBMED	off topic	no
electrocardiographic abnormalities in patients with cardiomyopathies	2024	10.1007/s10741-023-10358-7	PUBMED	duplicate	no
electrocardiographic alterations in patients with chronic obstructive pulmonary disease	2022	10.4330/ wjc.v14.i3.187	PUBMED	off topic	no

electrocardiographic and autonomic nervous system changes after changes in the posture of children and adolescents with duchenne muscular dystrophy	2024	10.36660/ abc.20230483	PUBMED	duplicate	no
electrocardiographic and electrophysiological characteristics of fasciculoventricular fibers in children	2022	10.1111/pace.14568	PUBMED	off topic	no
electrocardiographic and histopathological characterizations of diabetic cardiomyopathy in rats	2022	10.1007/s11356-021-17831-6	PUBMED	off topic	no
electrocardiographic biomarkers to predict atrial fibrillation in sinus rhythm electrocardiograms	2021	10.1136/ heartjnl-2021-319120	PUBMED	off topic	no
electrocardiographic changes associated with epilepsy beyond heart rate and their utilization in future seizure detection and forecasting methods	2020	10.1016/ j.clinph.2020.01.007	PUBMED	off topic	no
electrocardiographic changes during initiation of lithium augmentation of antidepressant pharmacotherapy	2022	10.1097/ jcp.0000000000000014 96	PUBMED	duplicate	no
electrocardiographic changes in newborns from mothers with metabolic syndrome	2021		PUBMED	off topic	no
electrocardiographic characteristics of bladder cancer patients receiving preoperative chemotherapy combined with immunotherapy	2024	10.1111/anec.13107	PUBMED	duplicate	no
electrocardiographic characteristics of breast cancer patients treated with chemotherapy	2020	10.1155/2020/66785 03	PUBMED	duplicate	no
electrocardiographic characteristics of diffuse large b-cell lymphoma patients treated with anthracycline-based chemotherapy	2020	10.1016/ j.jelectrocard.2020.0 4.024	PUBMED	duplicate	no
electrocardiographic characteristics of pediatric and adolescent football players	2023	10.1016/ j.smhs.2023.12.004	PUBMED	off topic	no
electrocardiographic correlates of acute allograft rejection among heart transplant recipients	2018	10.4037/ ajcc2018862	PUBMED	off topic	no
electrocardiographic detection of left ventricular hypertrophy; adding body mass index and spatial qrs-t angle: a cross-sectional study	2019	10.1007/s40119-019-00151-9	PUBMED	off topic	no
electrocardiographic features of 431 consecutive, critically ill covid-19 patients: an insight into the mechanisms of cardiac involvement	2020	10.1093/europace/ euaa258	PUBMED	off topic	no

electrocardiographic findings in human immunodeficiency virus-infected children in benin city, nigeria	2020	10.4103/npmj.npmj_92_20	PUBMED	off topic	no
electrocardiographic findings in true acute left main coronary total occlusion a subanalisys from atolma registry	2021	10.1016/j.jelectrocard.2021.07.017	PUBMED	off topic	no
electrocardiographic interpretation in athletes	2021	10.23736/s2724-5683.20.05331-1	PUBMED	off topic	no
electrocardiographic manifestations in paediatric wilson disease	2018		PUBMED	off topic	no
electrocardiographic modifications and cardiac involvement in covid-19 patients: results from an italian cohort	2021	10.2459/jcm.0000000000001166	PUBMED	duplicate	no
electrocardiographic patterns in biventricular pacing delivered by second-generation cardiac resynchronization devices	2018	10.1016/j.ipej.2017.10.007	PUBMED	off topic	no
electrocardiographic patterns of depolarization abnormalities help to identify reduced left ventricular ejection fraction	2022	10.3390/diagnostics12082020	PUBMED	off topic	no
electrocardiographic predictors of heart failure with reduced versus preserved ejection fraction: the multi-ethnic study of atherosclerosis	2017	10.1161/jaha.117.006023	PUBMED	off topic	no
electrocardiographic predictors of left ventricular scar in athletes with right bundle branch block premature ventricular beats	2024	10.1093/eurjpc/zwae010	PUBMED	off topic	no
electrocardiographic predictors of myocardial fibrosis and apical hypertrophic cardiomyopathy	2019	10.1111/anec.12612	PUBMED	duplicate	no
electrocardiographic strain pattern is associated with left ventricular concentric remodeling, scar, and mortality over 10 years: the multi-ethnic study of atherosclerosis	2017	10.1161/jaha.117.006624	PUBMED	duplicate	no
electrocardiographic temporo-spatial assessment of depolarization and repolarization changes after epicardial arrhythmogenic substrate ablation in brugada syndrome	2023	10.1093/ehjdh/ztad050	PUBMED	off topic	no
electrocardiography is unreliable to detect potential lethal hyperkalemia in patients with non-dialysis chronic kidney disease	2022	10.1007/s00246-022-02826-y	PUBMED	duplicate	no

electrocardiography is useful to predict postoperative ventricular arrhythmia in patients undergoing cardiac surgery: a retrospective study	2022	10.3389/fphys.2022.873821	PUBMED	off topic	no
electrographic lead i and v(5) monitoring could have detected a missed left-side pneumothorax intraoperatively	2023	10.1111/anec.13017	PUBMED	duplicate	no
electrophysiological and therapeutic effects of amiodarone in patients with preexcited atrial fibrillation	2022	10.4103/jrms.jrms_91_22	PUBMED	off topic	no
electrophysiological properties and heart rate variability of patients with thalassemia major in jakarta, indonesia	2023	10.1371/journal.pone.0280401	PUBMED	off topic	no
emerging ecg methods for acute coronary syndrome detection: recommendations & future opportunities	2022	10.1016/j.jelectrocard.2022.08.003	PUBMED	duplicate	no
enabling heart self-monitoring for all and for aal-portable device within a complete telemedicine system	2019	10.3390/s19183969	PUBMED	off topic	no
end-to-end premature ventricular contraction detection using deep neural networks	2023	10.3390/s23208573	PUBMED	off topic	no
endocardial pacing compared to epicardial left ventricle pacing and right ventricle pacing: a single-center long-term experience in a pediatric population	2024	10.1016/j.ipej.2023.11.003	PUBMED	off topic	no
entropy information of cardiorespiratory dynamics in neonates during sleep	2017	10.3390/e19050225	PUBMED	duplicate	no
environmental pollutant pre- and polyfluoroalkyl substances are associated with electrocardiogram parameters disorder in adults	2023	10.1016/j.jhazmat.2023.131832	PUBMED	off topic	no
epsilon wave masked by st-segment elevation after cardioversion from sustained ventricular tachycardia: an exceptional manifestation of arrhythmogenic right ventricular dysplasia/cardiomyopathy	2022	10.1016/j.jemermed.2021.10.033	PUBMED	off topic	no
estimation of pq distance dispersion for atrial fibrillation detection	2021	10.1016/j.cmpb.2021.106167	PUBMED	duplicate	no
evaluation of 12-lead electrocardiogram at 0.55t for improved cardiac monitoring in magnetic resonance imaging	2024	10.1016/j.jocmr.2024.101009	PUBMED	multichannel	no
evaluation of a novel precordial lead system for the electrocardiographic diagnosis of right ventricular enlargement in dogs	2022	10.3390/vetsci9080399	PUBMED	off topic	no

evaluation of index of cardiac electrophysiological balance in covid-19 patients	2021	10.4149/bll_2021_096	PUBMED	off topic	no
evaluation of the tp-e interval and tp-e/qtc ratio in patients with benign paroxysmal positional vertigo in the emergency department compared with the normal population	2020	10.1016/j.jelectrocard.2019.11.002	PUBMED	duplicate	no
evolution of single-lead ecg for stemi detection using a deep learning approach	2022	10.1016/j.ijcard.2021.11.039	PUBMED	off topic	no
exploratory data analysis based efficient qrs-complex detection technique with minimal computational load	2020	10.1007/s13246-020-00906-y	PUBMED	fetal	yes
extraction of foetal ecg from abdominal ecg by nonlinear transformation and estimations	2019	10.1016/j.cmpb.2019.04.022	PUBMED	fetal	no
f-wave extraction from single-lead electrocardiogram signals with atrial fibrillation by utilizing an optimized resonance-based signal decomposition method	2022	10.3390/e24060812	PUBMED	off topic	no
failure of intracardiac pacing after fatal propafenone overdose: a case report	2018	10.1016/j.jemermed.2017.12.021	PUBMED	off topic	no
false alarm reduction in self-care by personalized automatic detection of ecg electrode cable interchanges	2020	10.1155/2020/9175673	PUBMED	off topic	no
false negative ecg device results may increase the risk of adverse events in clinical oncology trials	2022	10.1007/s43441-022-00405-0	PUBMED	duplicate	no
fast qrs detection and ecg compression based on signal structural analysis	2019	10.1109/jbhi.2018.2792404	PUBMED	duplicate	no
feasibility analysis of ecg-based ph estimation for asphyxia detection in neonates	2024	10.3390/s24113357	PUBMED	off topic	no
feasibility of a novel ecg electrode placement method in newborn infants	2022	10.1159/000521530	PUBMED	duplicate	no
feasibility of non-invasive fetal electrocardiographic interval measurement in the outpatient clinical setting	2019	10.1007/s00246-019-02128-w	PUBMED	fetal	no
feature extraction of ecg signal	2018	10.1080/03091902.2018.1492039	PUBMED	not accessible	no
fetal arrhythmias: prenatal evaluation and intrauterine therapeutics	2020	10.1186/s13052-020-0785-9	PUBMED	fetal	no

fetal cardiac time intervals in healthy pregnancies - an observational study by fetal ecg (monica healthcare system)	2018	10.1515/jpm-2017-0003	PUBMED	duplicate	no
fetal ecg extraction from maternal ecg using attention-based cyclegan	2022	10.1109/jbhi.2021.3111873	PUBMED	fetal	no
fetal electrocardiogram extraction and analysis using adaptive noise cancellation and wavelet transformation techniques	2017	10.1007/s10916-017-0868-3	PUBMED	fetal	no
fetal electrocardiogram extraction using dual-path source separation of single-channel non-invasive abdominal recordings	2023	10.1109/tbme.2022.3189617	PUBMED	fetal	no
fetal electrocardiogram signal extraction based on fast independent component analysis and singular value decomposition	2022	10.3390/s22103705	PUBMED	duplicate	no
fetal qrs detection in noninvasive abdominal electrocardiograms using principal component analysis and discrete wavelet transforms with signal quality estimation	2021	10.31661/jbpe.v0i0.397	PUBMED	fetal	no
filamin-c variant-associated cardiomyopathy: a pooled analysis of individual patient data to evaluate the clinical profile and risk of sudden cardiac death	2022	10.1016/j.hrthm.2021.09.029	PUBMED	off topic	no
filtering of ecg signals distorted by magnetic field gradients during mri using non-linear filters and higher-order statistics	2018	10.1515/bmt-2016-0232	PUBMED	off topic	no
first description of the burmeister porpoise (phocoena spinipinnis) electrocardiogram	2022	10.1002/jez.2641	PUBMED	off topic	no
four-dimensional speckle tracking echocardiography and fragmented qrs in detection of early left ventricular systolic dysfunction in patients with subclinical hyperthyroidism	2023	10.1002/jcu.23459	PUBMED	off topic	no
fragmentation of the qrs complex is associated with right ventricular dilatation and mortality in critically unwell coronavirus disease 2019 patients	2024	10.14744/anatoljcardiol.2024.3494	PUBMED	off topic	no
fragmented qrs as a marker of myocardial fibrosis in hypertension: a systematic review	2019	10.1007/s11906-019-0982-3	PUBMED	off topic	no
fragmented qrs as a predictor of hypertensive crisis in patients with newly diagnosed essential hypertension: 4-year follow-up data	2023	10.1007/s00059-023-05194-2	PUBMED	off topic	no

fragmented qrs as a predictor of subclinical cardiovascular disease in patients with chronic kidney disease	2020	10.1111/imj.14743	PUBMED	duplicate	no
fragmented qrs complex frequency and location as predictor of cardiogenic shock and mortality following acute coronary syndrome	2020	10.1186/s43044-020-00076-y	PUBMED	off topic	no
fragmented qrs complex in patients with systemic lupus erythematosus at the time of diagnosis and its relationship with disease activity	2020	10.1371/journal.pone.0227022	PUBMED	off topic	no
fragmented qrs complex may predict long-term mortality after isolated surgical aortic valve replacement in patients with severe aortic stenosis	2022	10.1093/icvts/ivab214	PUBMED	off topic	no
fragmented qrs complexes are associated with subclinical left ventricular dysfunction in patients with behcet's disease: four-dimensional speckle tracking echocardiography	2021	10.1002/jcu.22899	PUBMED	off topic	no
fragmented qrs is associated with ventricular arrhythmias in heart failure patients: a systematic review and meta-analysis	2022	10.1111/anec.12910	PUBMED	off topic	no
fragmented qrs on 12-lead electrocardiogram is correlated with severe coronary artery disease and abnormal myocardial perfusion scintigraphy results in renal transplant candidates	2018	10.6002/ect.2017.0263	PUBMED	multichannel	no
fragmented qrs on electrocardiography as a predictor for diastolic cardiac dysfunction in type 2 diabetes	2022	10.1111/jdi.13759	PUBMED	off topic	no
fragmented qrs on electrocardiography as a predictor of myocardial scar in patients with hypertrophic cardiomyopathy	2020	10.1080/00015385.2018.1547355	PUBMED	off topic	no
fragmented qrs on far-field intracardiac electrograms as a predictor of arrhythmic events	2021	10.1002/joa3.12622	PUBMED	off topic	no
fragmented qrs on surface electrocardiogram as a predictor of perfusion defect in patients with suspected coronary artery disease undergoing myocardial perfusion imaging	2018	10.1016/j.ihj.2018.09.011	PUBMED	off topic	no
fragmented ventricular complexes and blood pressure variability assessed by ambulatory blood pressure monitoring in patients with metabolic syndrome	2024	10.7759/cureus.59950	PUBMED	off topic	no

frequent accelerated idioventricular rhythm in an otherwise healthy child: a case report and review of literature	2023	10.1186/s12872-023-03074-5	PUBMED	off topic	no
from pacemaker to wearable: techniques for ecg detection systems	2018	10.1007/s10916-017-0886-1	PUBMED	duplicate	no
frontal plane qrs-t angle as a marker of cardiac iron overload in patients with beta thalassemia major	2023	10.6515/acs.202305_39(3).20221114b	PUBMED	off topic	no
frontal qrs-t angle as a predictive marker for myocardial damage in acute carbon monoxide poisoning	2021	10.1177/09603271211043477	PUBMED	off topic	no
frontal qrs-t angle may predict reverse dipping pattern in masked hypertensives	2022	10.1080/10641963.2022.2043892	PUBMED	off topic	no
frontal qrs-t angle predicts syntax score in patients with non-st elevation myocardial infarction	2020	10.1016/j.jelectrocard.2020.06.008	PUBMED	duplicate	no
fully automatic detection of strict left bundle branch block	2018	10.1016/j.jelectrocard.2018.06.013	PUBMED	off topic	no
fully disposable wireless patch sensor for continuous remote patient monitoring	2018	10.1109/embc.2018.8512569	PUBMED	duplicate	no
general introduction, classification, and electrocardiographic diagnosis of cardiac arrhythmias	2017	10.1016/j.ccep.2017.05.009	PUBMED	off topic	no
generalising electrocardiogram detection and delineation: training convolutional neural networks with synthetic data augmentation	2024	10.3389/fcvm.2024.1341786	PUBMED	off topic	no
heart rate monitoring and therapeutic devices: a wavelet transform based approach for the modeling and classification of congestive heart failure	2018	10.1016/j.isatra.2018.05.003	PUBMED	duplicate	no
heart rate variability: measurement and emerging use in critical care medicine	2020	10.1177/1751143719853744	PUBMED	off topic	no
heartbeat detection in seismocardiograms with semantic segmentation	2022	10.1109/embc48229.2022.9871477	PUBMED	off topic	no
heartbeat detector from ecg and ppg signals based on wavelet transform and upper envelopes	2023	10.1007/s13246-023-01235-6	PUBMED	duplicate	no
hidden markov model-based heartbeat detector using electrocardiogram and arterial pressure signals	2021	10.1007/s13534-021-00192-x	PUBMED	duplicate	no

high incidence of subclinical atrial fibrillation in patients with syncope monitored with implantable cardiac monitor	2020	10.1016/ j.ijcard.2020.05.078	PUBMED	off topic	no
high precision digitization of paper-based ecg records: a step toward machine learning	2019	10.1109/ jtehm.2019.2949784	PUBMED	duplicate	no
high-frequency ecg for detection of myocardial ischemia associated with right coronary artery stenosis in ihd patients	2020	10.17691/ stm2020.12.1.11	PUBMED	off topic	no
high-resolution mapping of ventricular scar: evaluation of a novel integrated multielectrode mapping and ablation catheter	2017	10.1016/ j.jacep.2016.12.016	PUBMED	off topic	no
higher dispersion measures of conduction and repolarization in type 1 compared to non-type 1 brugada syndrome patients: an electrocardiographic study from a single center	2018	10.3389/ fcvm.2018.00132	PUBMED	off topic	no
his bundle pacing - a curative method: a case report	2020	10.1097/ md.0000000000021 633	PUBMED	off topic	no
how are ecg parameters related to cardiac magnetic resonance images? electrocardiographic predictors of left ventricular hypertrophy and myocardial fibrosis in hypertrophic cardiomyopathy	2020	10.1111/anec.12763	PUBMED	off topic	no
hrv-spark: computing heart rate variability measures using apache spark	2020	10.1109/ bibm49941.2020.93 13361	PUBMED	off topic	no
hsf1 and gm-csf expression, its association with cardiac health, and assessment of organ function during heat stress in crossbred jersey cattle	2021	10.1016/ j.rvsc.2021.07.018	PUBMED	off topic	no
hypertension predisposition and thermoregulation delays in adolescents with polycystic ovary syndrome: a pilot study	2022	10.3390/ children9030316	PUBMED	off topic	no
identification of transient noise to reduce false detections in screening for atrial fibrillation	2021	10.3389/ fphys.2021.672875	PUBMED	off topic	no
identifying mitral valve prolapse at risk for arrhythmias and fibrosis from electrocardiograms using deep learning	2023	10.1016/ j.jacadv.2023.10044 6	PUBMED	duplicate	no

impact of qrs misclassifications on heart-rate-variability parameters (results from the carla cohort study)	2024	10.1371/ journal.pone.030489 3	PUBMED	duplicate	no
impact of the distance from the chest wall to the heart on surface ecg voltage in athletes	2020	10.1136/ bmjsem-2019- 000696	PUBMED	off topic	no
implementation and validation of real-time algorithms for atrial fibrillation detection on a wearable ecg device	2020	10.1016/ j.compbiomed.2019. 103540	PUBMED	off topic	no
implementation of wavelet-transform-based algorithms in an fpga for heart rate and rt interval automatic measurements in real time: application in a long-term ambulatory electrocardiogram monitor	2023	10.3390/ mi14091748	PUBMED	hardware	no
importance of the heart vector origin point definition for an ecg analysis: the atherosclerosis risk in communities (aric) study	2019	10.1016/ j.compbiomed.2018. 11.013	PUBMED	duplicate	no
improved evaluation of left ventricular hypertrophy using the spatial qrs-t angle by electrocardiography	2022	10.1038/s41598- 022-16712-3	PUBMED	duplicate	no
improved scoring system for the electrocardiographic diagnosis of left ventricular hypertrophy	2019	10.4330/ wjc.v11.i3.94	PUBMED	off topic	no
improved t-wave detection in electrocardiogram signals based non-stationary wavelet transform and qrs complex cancellation with kurtosis analysis	2023	10.1088/1361- 6579/ad0b3e	PUBMED	off topic	no
improving r peak detection in ecg signal using dynamic mode selected energy and adaptive window sizing algorithm with decision tree algorithm	2021	10.3390/s21196682	PUBMED	duplicate	no
improving the qrs detection for one-channel ecg sensor	2019	10.3233/thc-181589	PUBMED	hardware	no
in search of an optimal subset of ecg features to augment the diagnosis of acute coronary syndrome at the emergency department	2021	10.1161/ jaha.120.017871	PUBMED	off topic	no
incidence of myopotential induction in subcutaneous implantable cardioverter-defibrillator patients: is the oversensing issue really solved?	2019	10.1016/ j.hrthm.2019.04.044	PUBMED	off topic	no
incremental diagnostic and prognostic value of the qrs-t angle, a 12-lead ecg marker quantifying heterogeneity of depolarization and repolarization, in patients with suspected non-st-elevation myocardial infarction	2019	10.1016/ j.ijcard.2018.09.040	PUBMED	off topic	no

incremental value of high-frequency qrs analysis for diagnosis and prognosis in suspected exercise-induced myocardial ischaemia	2020	10.1177/2048872619842988	PUBMED	off topic	no
independent component analysis algorithms for non-invasive fetal electrocardiography	2023	10.1371/journal.pone.0286858	PUBMED	fetal	no
independent detection of t-waves in single lead ecg signal using continuous wavelet transform	2023	10.1007/s13239-022-00643-1	PUBMED	duplicate	no
index of cardiac-electrophysiological balance and the effects of thrombolytic therapy on the electrocardiogram of patients with pulmonary embolism	2020	10.1590/1806-9282.66.12.1657	PUBMED	off topic	no
interventricular differences in sodium current and its potential role in brugada syndrome	2018	10.14814/phy2.13787	PUBMED	off topic	no
intraventricular delay and blocks	2018	10.1016/j.ccep.2018.02.003	PUBMED	off topic	no
investigation of icam-1 levels in hypertensive patients with fragmented qrs complexes	2020	10.1080/00015385.2018.1555200	PUBMED	off topic	no
iot-enabled cloud-based real-time remote ecg monitoring system	2021	10.1080/03091902.2021.1921870	PUBMED	duplicate	no
is ventricular sensing always right, when it is left?	2018	10.1002/clc.23033	PUBMED	off topic	no
j wave syndromes in patients with spinal and bulbar muscular atrophy	2022	10.1007/s00415-022-10992-5	PUBMED	duplicate	no
left atrial diameter and atrial ectopic burden in patients with embolic stroke of undetermined source: risk stratification of atrial fibrillation with insertable cardiac monitor analysis	2021	10.3988/jcn.2021.17.2.213	PUBMED	off topic	no
left bundle branch area pacing guided by continuous uninterrupted monitoring of unipolar pacing characteristics	2022	10.1111/jce.15302	PUBMED	off topic	no
left bundle branch potential predicts better electrical synchrony in bradycardia patients receiving left bundle branch pacing	2022	10.1186/s12872-022-02812-5	PUBMED	off topic	no
left ventricular outflow tract arrhythmias with divergent qrs morphology: mapping of different exits and ablation strategy	2018	10.1007/s10840-017-0307-x	PUBMED	off topic	no
left ventricular systolic dysfunction predicted by artificial intelligence using the electrocardiogram in chagas disease patients-the sami-trop cohort	2021	10.1371/journal.pntd.0009974	PUBMED	duplicate	no

left ventricular twist in hypertrophic cardiomyopathy : predictor of nonsustained ventricular tachycardia	2019	10.1007/s00059-017-4633-7	PUBMED	off topic	no
life-threatening ventricular arrhythmia prediction in patients with dilated cardiomyopathy using explainable electrocardiogram-based deep neural networks	2022	10.1093/europace/euac054	PUBMED	duplicate	no
lightweight heartbeat detection algorithm for consumer grade wearable ecg measurement devices and its implementation	2022	10.1109/embc48229.2022.9871514	PUBMED	inaccessible dataset	yes
limitations of electrocardiography for detecting left ventricular hypertrophy or concentric remodeling in athletes	2020	10.1016/j.amjmed.2019.06.028	PUBMED	off topic	no
linear phase sharp transition bpf to detect noninvasive maternal and fetal heart rate	2018	10.1155/2018/5485728	PUBMED	duplicate	no
local interval estimation improves accuracy and robustness of heart rate variability derivation from photoplethysmography	2018	10.1109/embc.2018.8512908	PUBMED	off topic	no
local left ventricular epicardial j waves and late potentials in brugada syndrome patients with inferolateral early repolarization pattern	2017	10.3389/fphys.2017.00014	PUBMED	off topic	no
longitudinal association of electrocardiogram abnormalities with major adverse cardiac events in people with type 2 diabetes: the hoorn diabetes care system cohort	2023	10.1093/eurjpc/zwac314	PUBMED	off topic	no
low-power long-term ambulatory electrocardiography monitor of three leads with beat-to-beat heart rate measurement in real time	2023	10.3390/s23198303	PUBMED	off topic	no
machine algorithm for heartbeat monitoring and arrhythmia detection based on ecg systems	2021	10.1155/2021/7677568	PUBMED	off topic	no
machine learning models of 6-lead ecgs for the interpretation of left ventricular hypertrophy (lvh)	2023	10.1016/j.jelectrocard.2022.12.001	PUBMED	duplicate	no
machine learning workflow for edge computed arrhythmia detection in exploration class missions	2024	10.1038/s41526-024-00409-0	PUBMED	off topic	no
magnetocardiography on an isolated animal heart with a room-temperature optically pumped magnetometer	2018	10.1038/s41598-018-34535-z	PUBMED	off topic	no
magnetocardiography using a magnetoresistive sensor array	2019	10.1536/ihj.18-002	PUBMED	off topic	no

mahaim fiber-mediated tachycardia	2018	10.1016/ j.repc.2017.01.008	PUBMED	off topic	no
major depressive disorder is associated with fragmented qrs	2017	10.1080/00015385.2 017.1291138	PUBMED	off topic	no
matched filtering for heart rate estimation on compressive sensing ecg measurements	2018	10.1109/ tbme.2017.2752422	PUBMED	does not introduce a new technique	yes
measurement of electrocardiograms in a bath through tap water utilizing capacitive coupling electrodes placed outside the bathtub wall	2017	10.1186/s12938-016-0304-9	PUBMED	off topic	no
mobile cardiac acoustic monitoring system to evaluate left ventricular systolic function in pacemaker patients	2022	10.3390/ jcm11133862	PUBMED	off topic	no
modeling the his-purkinje effect in non-invasive estimation of endocardial and epicardial ventricular activation	2022	10.1007/s10439-022-02905-4	PUBMED	off topic	no
modelling of the electrocardiographic signal during an angioplasty procedure in the right coronary artery	2020	10.1016/ j.jelectrocard.2020.0 8.003	PUBMED	off topic	no
modifications in electrocardiographic and vectordardiographic morphological parameters in elderly males as result of cardiovascular diseases and diabetes mellitus	2022	10.3390/ diagnostics1212291 1	PUBMED	off topic	no
modified automatic r-peak detection algorithm for patients with epilepsy using a portable electrocardiogram recorder	2017	10.1109/ embc.2017.8037753	PUBMED	off topic	no
morbid obesity influences the nocturnal electrocardiogram wave and interval durations among suspected sleep apnea patients	2024	10.1111/anec.13101	PUBMED	off topic	no
morphologically proved anca positive loeffler's pancarditis: medical and surgical treatment	2019	10.26442/00403660. 2019.04.000048	PUBMED	off topic	no
morphology extraction of fetal ecg using temporal cnn-based nonlinear adaptive noise cancelling	2022	10.1371/ journal.pone.027891 7	PUBMED	fetal	no
multi-dry-electrode plate sensor for non-invasive electrocardiogram and heart rate monitoring for the assessment of drug responses in freely behaving mice	2019	10.1016/ j.vascn.2019.02.009	PUBMED	off topic	no

multi-ethnic genome-wide association study of decomposed cardioelectric phenotypes illustrates strategies to identify and characterize evidence of shared genetic effects for complex traits	2020	10.1161/circgen.119.002680	PUBMED	off topic	no
multi-purpose ecg telemetry system	2017	10.1186/s12938-017-0371-6	PUBMED	off topic	no
multichannel ecg recording from waist using textile sensors	2020	10.1186/s12938-020-00788-x	PUBMED	duplicate	no
multichannel high noise level ecg denoising based on adversarial deep learning	2024	10.1038/s41598-023-50334-7	PUBMED	multichannel	no
multichannel qrs detection by combinatorial optimization	2024	10.1109/embc53108.2024.10782971	PUBMED	multichannel	no
multiple ecg fiducial points-based random binary sequence generation for securing wireless body area networks	2017	10.1109/jbhi.2016.2546300	PUBMED	off topic	no
myocardial fibrosis by late gadolinium enhancement cardiovascular magnetic resonance in myotonic muscular dystrophy type 1: highly prevalent but not associated with surface conduction abnormality	2019	10.1186/s12968-019-0535-6	PUBMED	off topic	no
myocardial scar on surface ecg: selvester score, but not fragmentation, predicts response to crt	2020	10.1155/2020/2036545	PUBMED	off topic	no
neonatal atrial flutter: a novel triad for immediate electrocardiographic diagnosis	2024		PUBMED	off topic	no
new features for the detection of fetal qrs complexes in non-invasive fetal electrocardiograms	2023	10.1109/embc40787.2023.10340399	PUBMED	duplicate	no
new implantable cardiac monitor with three-lead ecg and active noise detection	2017	10.1007/s00059-016-4492-7	PUBMED	multichannel	no
new possibilities for st analysis - a post-hoc analysis on the dutch stan rct	2022	10.1016/j.earlhumdev.2021.105537	PUBMED	duplicate	no
noise detection in electrocardiogram signals for intensive care unit patients	2019	10.1109/access.2019.2926199	PUBMED	duplicate	no
noise detection in electrocardiography signal for robust heart rate variability analysis: a deep learning approach	2018	10.1109/embc.2018.8513537	PUBMED	off topic	no

noise reduction and qrs detection in ecg signal using eemd with modified sigmoid thresholding	2023	10.1515/bmt-2022-0450	PUBMED	denoising	yes
non-contact monitoring of ecg in the home environment-selecting optimal electrode configuration	2022	10.3390/s22239475	PUBMED	off topic	no
non-invasive diagnosis of sleep apnoea using ecg and respiratory bands	2019	10.1109/embc.2019.8857414	PUBMED	off topic	no
non-invasive heart failure evaluation using machine learning algorithms	2024	10.3390/s24072248	PUBMED	off topic	no
non-perfusing cardiac rhythms in asphyxiated newborn piglets	2019	10.1371/journal.pone.0214506	PUBMED	off topic	no
non-standard electrode placement strategies for ecg signal acquisition	2022	10.3390/s22239351	PUBMED	off topic	no
nontraditional electrocardiogram and algorithms for inconspicuous in-home monitoring: comparative study	2018	10.2196/mhealth.9604	PUBMED	off topic	no
nortriptyline serum concentration as a predictor for cardiac risk in amitriptyline-treated patients	2020	10.1007/s00228-019-02766-2	PUBMED	off topic	no
novel cineecg derived from standard 12-lead ecg enables right ventricle outflow tract localization of electrical substrate in patients with brugada syndrome	2020	10.1161/circep.120.008524	PUBMED	multichannel	no
novel derma fusion technique for ecg heartbeat classification	2022	10.3390/life12060842	PUBMED	duplicate	no
novel ecg features and machine learning to optimize culprit lesion detection in patients with suspected acute coronary syndrome	2021	10.1016/j.jelectrocard.2021.07.012	PUBMED	duplicate	no
novel electrocardiographic dyssynchrony criteria that may improve patient selection for cardiac resynchronization therapy	2022	10.11909/j.issn.1671-5411.2022.01.006	PUBMED	off topic	no
novel t-wave detection technique with minimal processing and rr-interval based enhanced efficiency	2019	10.1007/s13239-019-00415-4	PUBMED	duplicate	no
occurrence of ventricular septal perforation in patients with permanent left bundle branch pacing followed up using echocardiographic and computed tomography images	2022	10.1111/anec.13002	PUBMED	off topic	no
on the beat detection performance in long-term ecg monitoring scenarios	2018	10.3390/s18051387	PUBMED	off topic	no

one-dimensional w-netr for non-invasive single channel fetal ecg extraction	2023	10.1109/ jbhi.2023.3266645	PUBMED	fetal	no
opening the black box: interpretability of machine learning algorithms in electrocardiography	2021	10.1098/ rsta.2020.0253	PUBMED	duplicate	no
optimal data fusion for the improvement of qrs complex detection in multi-channel ecg recordings	2019	10.1007/s11517-019-01990-3	PUBMED	multichannel	no
optimizing ecg to detect echocardiographic left ventricular hypertrophy with computer-based ecg data and machine learning	2021	10.1371/ journal.pone.0260661	PUBMED	duplicate	no
osborn wave and new-onset atrial fibrillation related to hypothermia after synthetic cannabis (bonsai) abuse	2019	10.5543/ tkda.2018.30513	PUBMED	off topic	no
overlapping and distinct features of cardiac pathology in inherited human and murine ether lipid deficiency	2023	10.3390/ ijms24031884	PUBMED	off topic	no
p and t wave detection and delineation of ecg signal using differential evolution (de) optimization strategy	2018	10.1007/s13246-018-0629-8	PUBMED	off topic	no
parallel use of a convolutional neural network and bagged tree ensemble for the classification of holter ecg	2018	10.1088/1361-6579/aad9ee	PUBMED	off topic	no
paroxysmal atrial fibrillation recognition based on multi-scale wavelet α-entropy	2017	10.1186/s12938-017-0406-z	PUBMED	off topic	no
pediatric ecg-based deep learning to predict left ventricular dysfunction and remodeling	2024	10.1161/ circulationaha.123.067750	PUBMED	duplicate	no
performance analysis of ten common qrs detectors on different ecg application cases	2018	10.1155/2018/9050812	PUBMED	review	no
performance evaluation of time-frequency distributions for ecg signal analysis	2017	10.1007/s10916-017-0871-8	PUBMED	duplicate	no
pharmacogenomics study of thiazide diuretics and qt interval in multi-ethnic populations: the cohorts for heart and aging research in genomic epidemiology	2018	10.1038/tpj.2017.10	PUBMED	off topic	no
phase space reconstruction based cvd classifier using localized features	2019	10.1038/s41598-019-51061-8	PUBMED	duplicate	no
physiological recording in the mri environment (prime): mri-compatible hemodynamic recording system	2018	10.1109/ jtehm.2018.2807813	PUBMED	off topic	no
platform for analysis and labeling of medical time series	2020	10.3390/s20247302	PUBMED	duplicate	no

pocketecg: a new noninvasive method for continuous and real-time ecg monitoring-initial results in children and adolescents	2017	10.1007/s00246-016-1534-0	PUBMED	off topic	no
postsystolic shortening is associated with altered right ventricular function in children after tetralogy of fallot surgical repair	2017	10.1371/journal.pone.0169178	PUBMED	off topic	no
power-mf: robust fetal qrs detection from non-invasive fetal electrocardiogram recordings	2024	10.1088/1361-6579/ad4952	PUBMED	fetal	no
pqrst wave detection on ecg signals	2021	10.1016/j.gaceta.2021.10.052	PUBMED	off topic	no
precise detection and localization of r-peaks from ecg signals	2023	10.3934/mbe.2023848	PUBMED	duplicate	no
precision and reproducibility of non-automatic measurement of the qrs complex in potential candidates for cardiac resynchronization therapy	2019	10.1016/j.jelectrocard.2019.08.011	PUBMED	duplicate	no
precision of automated qrs duration measurement in patients treated with cardiac resynchronization therapy	2018	10.1007/s10840-018-0334-2	PUBMED	off topic	no
precordial electrocardiographic recording and qt measurement from a novel wearable ring device	2023	10.1016/j.cvdhj.2023.11.021	PUBMED	duplicate	no
predicting long-term risk of sudden cardiac death with automatic computer-interpretations of electrocardiogram	2024	10.3389/fcvm.2024.1439069	PUBMED	off topic	no
predicting peri-operative troponin elevation by advanced electrocardiography	2021	10.1016/j.jelectrocard.2021.06.008	PUBMED	duplicate	no
prediction of both electrical and mechanical reverse remodeling on acute electrocardiogram changes after cardiac resynchronization therapy	2017	10.1253/circj.cj-16-1181	PUBMED	off topic	no
predictive value of frontal qrs-t angle after cardiac resynchronization therapy	2021	10.1016/j.jelectrocard.2021.06.015	PUBMED	duplicate	no
predictors of advanced conduction disturbances requiring a late (≥48 h) permanent pacemaker following transcatheter aortic valve replacement	2018	10.1016/j.jcin.2018.06.014	PUBMED	off topic	no
presence of fragmented qrs is associated with increased epicardial adipose tissue thickness in hypertensive patients	2019	10.1002/jcu.22683	PUBMED	off topic	no

presetting ecg electrodes for earlier heart rate detection in the delivery room	2018	10.1016/j.resuscitation.2018.03.038	PUBMED	duplicate	no
prevalence and factors associated with impaired left ventricular global longitudinal strain in patients with chagas disease: sami-trop cohort study	2022	10.1007/s10554-022-02640-w	PUBMED	off topic	no
prevalence and predictive value of electrocardiographic abnormalities in pulmonary hypertension: evidence from the pan-african pulmonary hypertension cohort (papuco) study	2017	10.5830/cvja-2017-020	PUBMED	duplicate	no
prevalence and predictors of false positive qtc prolongation by the automated measurement	2024	10.3389/fcvm.2024.1465264	PUBMED	off topic	no
prevalence and temporal distribution of extrasystoles in septic icu patients: the feasibility of predicting fluid responsiveness using extrasystoles	2018	10.1155/2018/5697092	PUBMED	off topic	no
prevalence of asymptomtic ventricular preexcitation among georgian athletes	2021		PUBMED	off topic	no
prognostic significance of fragmented qrs in patients with st-elevation myocardial infarction undergoing revascularization	2018	10.1016/j.ihj.2018.07.014	PUBMED	off topic	no
prognostic value of fragmented qrs complex in patients with acute myocardial infarction	2021	10.1007/s00059-020-04940-0	PUBMED	off topic	no
prognostic value of goldberger's electrocardiographic criteria for left ventricular dysfunction	2021	10.1016/j.jelectrocard.2020.11.011	PUBMED	duplicate	no
prolonged ventricular repolarization associated with mild cognitive impairment and white matter hyperintensities: a cross-sectional study	2024	10.1038/s41598-024-65364-y	PUBMED	off topic	no
prominent j wave in cats with hypertrophic cardiomyopathy	2021	10.1292/jvms.20-0415	PUBMED	off topic	no
prone and supine 12-lead ecg comparisons: implications for cardiac assessment during prone ventilation for covid-19	2021	10.1016/j.jacep.2021.04.011	PUBMED	multichannel	no
publisher correction: efficient ecg compression and qrs detection for e-health applications	2017	10.1038/s41598-017-17101-x	PUBMED	duplicate	no
pulsation artifact removal from intra-operatively recorded local field potentials using sparse signal processing and data-specific dictionary	2023	10.1109/embc40787.2023.10340160	PUBMED	duplicate	no

pulse arrival time and pulse interval as accurate markers to detect mechanical alternans	2019	10.1007/s10439-019-02221-4	PUBMED	duplicate	no
qrs complex detection and measurement algorithms for multichannel ecgs in cardiac resynchronization therapy patients	2018	10.1109/jtehm.2018.2844195	PUBMED	duplicate	no
qrs complex detection in ecg signals using locally adaptive weighted total variation denoising	2017	10.1016/j.compbiomed.2017.05.027	PUBMED	denoising	no
qrs complex detection using stationary wavelet transform and adaptive thresholding	2022	10.1088/2057-1976/ac8e70	PUBMED	duplicate	no
qrs detection and classification in holter ecg data in one inference step	2022	10.1038/s41598-022-16517-4	PUBMED	duplicate	no
qrs detection and measurement method of ecg paper based on convolutional neural networks	2018	10.1109/embc.2018.8513132	PUBMED	duplicate	no
qrs detection based on improved adaptive threshold	2018	10.1155/2018/5694595	PUBMED	does not introduce a new technique	yes
qrs detection in single-lead, telehealth electrocardiogram signals: benchmarking open-source algorithms	2024	10.1371/journal.pdig.0000538	PUBMED	does not introduce a new technique	no
qrs detection using adaptive filters: a comparative study	2017	10.1016/j.isatra.2016.09.023	PUBMED	INCLUDED	yes
qrs detector performance evaluation aware of temporal accuracy and presence of noise	2024	10.3390/s24051698	PUBMED	off topic	no
qrs dispersion detected in arvc patients and healthy gene carriers using 252-leads body surface mapping: an explorative study of a potential diagnostic tool for arrhythmogenic right ventricular cardiomyopathy	2021	10.1111/pace.14295	PUBMED	duplicate	no
qrs transitional zone rotations and the risk of atrial fibrillation: the suita study	2022	10.1007/s00380-022-02101-3	PUBMED	duplicate	no
qrstree: a prefix tree-based model to fetal qrs complexes detection	2019	10.1371/journal.pone.0223057	PUBMED	fetal	no
qt interval instability and qrs interval dispersion in healthy cats and cats with a hypertrophic cardiomyopathy phenotype	2023	10.1177/1098612x231151479	PUBMED	animal	no
quantification of abnormal qrs peaks predicts response to cardiac resynchronization therapy and tracks structural remodeling	2019	10.1371/journal.pone.0217875	PUBMED	off topic	no

quantitative approach to fragmented qrs in arrhythmogenic cardiomyopathy: from disease towards asymptomatic carriers of pathogenic variants	2020	10.3390/jcm9020545	PUBMED	off topic	no
quantitative fragmented qrs has a good diagnostic value on myocardial fibrosis in hypertrophic obstructive cardiomyopathy based on clinical-pathological study	2020	10.1186/s12872-020-01590-2	PUBMED	duplicate	no
r peak detection method using wavelet transform and modified shannon energy envelope	2017	10.1155/2017/4901017	PUBMED	INCLUDED	yes
r-deco: an open-source matlab based graphical user interface for the detection and correction of r-peaks	2019	10.7717/peerj-cs.226	PUBMED	off topic	no
r-wave singularity: a new morphological approach to the analysis of cardiac electrical dyssynchrony	2020	10.3389/fphys.2020.599838	PUBMED	off topic	no
ranking of the most reliable beat morphology and heart rate variability features for the detection of atrial fibrillation in short single-lead ecg	2018	10.1088/1361-6579/aad9f0	PUBMED	off topic	no
real time qrs complex detection using dfa and regular grammar	2017	10.1186/s12938-017-0322-2	PUBMED	missing technical details	yes
real-time magnetocardiography with passive miniaturized coil array in earth ambient field	2023	10.3390/s23125567	PUBMED	off topic	no
real-time quality assessment of long-term ecg signals recorded by wearables in free-living conditions	2020	10.1109/tbme.2020.2969719	PUBMED	off topic	no
recognition of premature ventricular contraction beat from 12lead ecg based on a novel detection function of qrs onset	2020	10.1109/embc44109.2020.9175775	PUBMED	off topic	no
recurrent nocturnal st-t deviation and nonsustained ventricular tachycardias recorded with a smartwatch: a case report	2022	10.1016/j.jccase.2022.04.006	PUBMED	duplicate	no
redundancy cancellation of compressed measurements by qrs complex alignment	2022	10.1371/journal.pone.0262219	PUBMED	off topic	no
reference intervals and method sensitivity for electrocardiology, hemodynamics, and body temperature parameters in healthy cynomolgus monkeys	2023	10.1016/j.vascn.2022.107247	PUBMED	duplicate	no
relationship between changes of electrocardiogram indexes in chronic heart failure with arrhythmia and serum piiinp and bnp	2020	10.3892/etm.2019.8269	PUBMED	off topic	no

relationship between fragmented qrs complex and aortic stiffness in chronic hemodialysis patients	2017	10.1159/000452418	PUBMED	off topic	no
relationship between late gadolinium enhancement and ventricular repolarization parameters in heart failure patients with reduced ejection fraction	2021	10.36660/ abc.20200149	PUBMED	off topic	no
relationship between resting 12-lead electrocardiogram and all-cause death in patients without structural heart disease: shinken database analysis	2021	10.1186/s12872-021-01864-3	PUBMED	multichannel	no
relationship of the t-wave tpeak-tend interval with conduction system disorders in arterial hypertension	2023	10.24875/ acm.21000396	PUBMED	off topic	no
relative versus absolute rises in t/qrs ratio by st analysis of fetal electrocardiograms in labour: a case-control pilot study	2019	10.1371/ journal.pone.0214357	PUBMED	fetal	no
remote ecg monitoring by ecg247 smart heart sensor	2022	10.1155/2022/6812889	PUBMED	duplicate	no
removal of ecg artifacts affects respiratory muscle fatigue detection-a simulation study	2021	10.3390/s21165663	PUBMED	duplicate	no
repolarization abnormalities unmasked with a 252-lead bsm system in patients with arvc and healthy gene carriers	2022	10.1111/pace.14456	PUBMED	off topic	no
representative qrs loop of the vcg record evaluation	2024	10.3389/ fphys.2023.1260074	PUBMED	off topic	no
resiniferatoxin reduces cardiac sympathetic nerve activation to exert a cardioprotective effect during myocardial infarction	2021		PUBMED	off topic	no
restoration of the electrocardiogram during mechanical cardiopulmonary resuscitation	2020	10.1088/1361-6579/ab9e53	PUBMED	off topic	no
resuscitated sudden cardiac death due to severe hypokalemia caused by teff grain herbal tea: a case report	2020	10.5543/ tkda.2020.57996	PUBMED	off topic	no
retrospective analysis of time limit of qrs complex in the electrocardiogram and its clinical significance in patients with acute myocardial infarction	2021		PUBMED	off topic	no
reverse electrical remodeling following pressure unloading in a rat model of hypertension-induced left ventricular myocardial hypertrophy	2017	10.1038/hr.2017.1	PUBMED	off topic	no

robust algorithm for the detection and classification of qrs complexes with different morphologies using the continuous spline wavelet transform with automatic scale detection	2024	10.1088/2057-1976/ad16c0	PUBMED	missing technical details	yes
robust arrhythmia classification based on qrs detection and a compact 1d-cnn for wearable ecg devices	2022	10.1109/jbhi.2022.3207456	PUBMED	duplicate	no
robust electrocardiogram delineation model for automatic morphological abnormality interpretation	2023	10.1038/s41598-023-40965-1	PUBMED	duplicate	no
robust heartbeat detection using multimodal recordings and ecg quality assessment with signal amplitudes dispersion	2018	10.1016/j.cmpb.2018.06.009	PUBMED	multichannel	no
robust qrs detection based on simulated degenerate optical parametric oscillator-assisted neural network	2024	10.1016/j.heliyon.2024.e28903	PUBMED	off topic	no
robust qrs detection for hrv estimation from compressively sensed ecg measurements for remote health-monitoring systems	2018	10.1088/1361-6579/aaa3c9	PUBMED	off topic	yes
robust, real-time generic detector based on a multi-feature probabilistic method	2019	10.1371/journal.pone.0223785	PUBMED	INCLUDED	yes
robustness of electrocardiogram signal quality indices	2022	10.1098/rsif.2022.0012	PUBMED	off topic	no
role of electrocardiographic and echocardiographic types of left bundle branch block in prediction of response to cardiac resynchronization therapy	2018	10.26442/00403660.2018.12.000012	PUBMED	off topic	no
rule-based rough-refined two-step-procedure for real-time premature beat detection in single-lead ecg	2020	10.1088/1361-6579/ab87b4	PUBMED	off topic	no
rundae model: running denoising autoencoder models for denoising ecg signals	2023	10.1016/j.compbiomed.2023.107553	PUBMED	denoising	no
safety and performance of the subcutaneous implantable cardioverter defibrillator detection algorithm insight(tm) in pacemaker patients	2023	10.3390/jcm13010129	PUBMED	off topic	no
saliency maps provide insights into artificial intelligence-based electrocardiography models for detecting hypertrophic cardiomyopathy	2023	10.1016/j.jelectrocard.2023.07.002	PUBMED	duplicate	no

scn5a mutation is associated with a higher shanghai score in patients with type 1 brugada ecg pattern	2023	10.2459/jcm.0000000000001560	PUBMED	off topic	no
sdnn24 estimation from semi-continuous hr measures	2021	10.3390/s21041463	PUBMED	off topic	no
searching for the best machine learning algorithm for the detection of left ventricular hypertrophy from the ecg: a review	2024	10.3390/bioengineering11050489	PUBMED	off topic	no
second-order level-crossing sampling analog to digital converter for electrocardiogram delineation and premature ventricular contraction detection	2023	10.1109/tbcas.2023.3296529	PUBMED	hardware	no
secure hardware ip of glrt cascade using color interval graph based embedded fingerprint for ecg detector	2024	10.1038/s41598-024-63533-7	PUBMED	hardware	no
self-attention mhdnet: a novel deep learning model for the detection of r-peaks in the electrocardiogram signals corrupted with magnetohydrodynamic effect	2023	10.3390/bioengineering10050542	PUBMED	duplicate	no
semantic segmentation of qrs complex in 12-lead ecg signals	2024	10.1109/embc53108.2024.10782056	PUBMED	multichannel	no
semi-automated detection of polysomnographic rem sleep without atonia (rswa) in rem sleep behavioral disorder	2019	10.1016/j.bspc.2019.02.023	PUBMED	off topic	no
seresuter: a deep learning approach for accurate ecg signal delineation and atrial fibrillation detection	2023	10.1088/1361-6579/ad02da	PUBMED	off topic	no
serial electrocardiograms at follow-up for early detection of transplanted heart rejection: a viewpoint	2024	10.1016/j.jelectrocard.2023.12.008	PUBMED	off topic	no
serial electrocardiography to detect newly emerging or aggravating cardiac pathology: a deep-learning approach	2019	10.1186/s12938-019-0630-9	PUBMED	duplicate	no
sevoflurane reduces the cardiac toxicity of bupivacaine compared with propofol in rabbits: an experimental study using early electrocardiographic detection and measurement of toxic plasma concentration	2024	10.5114/ait.2024.145167	PUBMED	off topic	no
shannon's energy based algorithm in ecg signal processing	2017	10.1155/2017/8081361	PUBMED	duplicate	no
signal processing framework for the detection of ventricular ectopic beat episodes	2023	10.4103/jmss.jmss_12_22	PUBMED	duplicate	no

signal quality analysis of single-arm electrocardiography	2023	10.3390/s23135818	PUBMED	off topic	no
signal quality assessment of a novel ecg electrode for motion artifact reduction	2021	10.3390/s21165548	PUBMED	off topic	no
signal quality in reconstructed 12-lead ambulatory ecgs recorded using 3-lead device	2019	10.1109/ embc.2019.8857251	PUBMED	multichannel	no
significant delayed conduction and characteristic ventricular tachycardias in patients with cardiac sarcoidosis and electrical storm	2024	10.1111/jce.16156	PUBMED	off topic	no
silent myocardial infarction: a case report	2023	10.7759/ cureus.43906	PUBMED	off topic	no
simultaneous electrocardiogram during routine electroencephalogram: arrhythmia rates through the eyes of the cardiologist	2021	10.1590/0004-282x20200105	PUBMED	off topic	no
single-arm diagnostic electrocardiography with printed graphene on wearable textiles	2023	10.1016/ j.sna.2022.114058	PUBMED	off topic	no
single-lead noninvasive fetal ecg extraction by means of combining clustering and principal components analysis	2020	10.1007/s11517-019-02087-7	PUBMED	duplicate	no
sleep-wake stage detection with single channel ecg and hybrid machine learning model in patients with obstructive sleep apnea	2021	10.1007/s13246-020-00953-5	PUBMED	duplicate	no
smartphone-based six-lead ecg: a new device for electrocardiographic recording in dogs	2024	10.1016/ j.tvjl.2023.106043	PUBMED	off topic	no
solithromycin, a novel macrolide, does not prolong cardiac repolarization: a randomized, three-way crossover study in healthy subjects	2017	10.1093/jac/dkw428	PUBMED	duplicate	no
spatiotemporal approximation of cardiac activation and recovery isochrones	2022	10.1016/ j.jelectrocard.2021.12.007	PUBMED	off topic	no
sqi quality evaluation mechanism of single-lead ecg signal based on simple heuristic fusion and fuzzy comprehensive evaluation	2018	10.3389/ fphys.2018.00727	PUBMED	off topic	no
st waveform analysis for monitoring hypoxic distress in fetal sheep after prolonged umbilical cord occlusion	2018	10.1371/ journal.pone.0195978	PUBMED	duplicate	no

stall-side screening potential of a smartphone electrocardiogram recorded over both sides of the thorax in horses	2020	10.1111/jvim.15795	PUBMED	off topic	no
structural, pro-inflammatory and calcium handling remodeling underlies spontaneous onset of paroxysmal atrial fibrillation in jdp2-overexpressing mice	2020	10.3390/ijms21239095	PUBMED	off topic	no
study of the few-shot learning for ecg classification based on the ptb-xl dataset	2022	10.3390/s22030904	PUBMED	off topic	no
substrate mapping for scar-related ventricular tachycardia in patients with resynchronization therapy-the importance of the pacing mode	2019	10.1007/s10840-019-00548-5	PUBMED	off topic	no
sudden cardiac death in patients with myocarditis: evaluation, risk stratification, and management	2020	10.1016/j.ahj.2019.08.007	PUBMED	off topic	no
supraventricular runs in 7-day holter monitoring are related to increased incidence of atrial fibrillation in a 3-year follow-up of cryptogenic stroke patients free from arrhythmia in a 24 h-holter	2021	10.3390/jcdd8070081	PUBMED	off topic	no
supraventricular tachycardias in neonates and infants: factors associated with fatal or near-fatal outcome	2021	10.1007/s00431-021-04159-z	PUBMED	off topic	no
survival and arrhythmic risk among ischemic and non-ischemic heart failure patients with prophylactic implantable cardioverter defibrillator only therapy: a propensity score-matched analysis	2019	10.1016/j.ijcard.2018.09.003	PUBMED	off topic	no
system for automatic heart rate calculation in epileptic seizures	2017	10.1007/s13246-017-0557-z	PUBMED	off topic	no
systematic analysis of single- and multi-reference adaptive filters for non-invasive fetal electrocardiography	2019	10.3934/mbe.2020016	PUBMED	fetal	no
temporal and spatial pacemap parameters for identification of cardiac surfaces with critical sites for ventricular tachycardia	2022	10.1111/jce.15611	PUBMED	off topic	no
temporal performance of laplacian eigenmaps and 3d conduction velocity in detecting ischemic stress	2018	10.1016/j.jelectrocard.2018.08.017	PUBMED	duplicate	no
the 43rd international society for computerized electrocardiology ecg initiative for the automated detection of strict left bundle branch block	2018	10.1016/j.jelectrocard.2018.08.001	PUBMED	off topic	no

the ability of the electrocardiogram in left bundle branch block to detect myocardial scar determined by cardiovascular magnetic resonance	2018	10.1016/j.jelectrocard.2018.05.019	PUBMED	duplicate	no
the accuracy of combined electrocardiogram criteria to diagnose right atrial enlargement in adults with uncorrected secundum atrial septal defect	2019	10.1177/1179546819869948	PUBMED	off topic	no
the application of ecg cancellation in diaphragmatic electromyographic by using stationary wavelet transform	2018	10.1007/s13534-018-0064-5	PUBMED	off topic	no
the association between ecg criteria and echo criteria for left ventricular hypertrophy in a general chinese population	2021	10.1111/anec.12880	PUBMED	duplicate	no
the association between heart rate variability and 90-day prognosis in patients with transient ischemic attack and minor stroke	2021	10.3389/fneur.2021.636474	PUBMED	off topic	no
the association of qt interval components with atrial fibrillation	2018	10.1111/anec.12467	PUBMED	off topic	no
the characterization of the transit through the anaerobic threshold based on relationships between rr and qrs cardiac intervals	2019	10.1371/journal.pone.0216938	PUBMED	off topic	no
the combination of high-frequency qrs and st-segment alterations during exercise stress tests enhanced the diagnostic efficacy for coronary artery disease	2024	10.1002/clc.24254	PUBMED	off topic	no
the ecg in sarcoidosis - a marker of cardiac involvement? current evidence and clinical implications	2021	10.1016/j.jjcc.2020.07.006	PUBMED	duplicate	no
the effect of electroconvulsive therapy on frontal qrs-t angle in psychiatric patients	2024	10.29399/npa.28443	PUBMED	off topic	no
the effect of exposure to mobile phones on electrical cardiac measurements: a multivariate analysis and a variable selection algorithm to detect the relationship with mean changes	2024	10.1155/2024/7093771	PUBMED	off topic	no
the effect of low-flow and normal-flow desflurane anesthesia on the frontal qrs-t angle in patients undergoing rhinoplasty operation: a randomized prospective study	2022	10.7759/cureus.28920	PUBMED	off topic	no
the effect of the severity covid-19 infection on electrocardiography	2021	10.1016/j.ajem.2020.10.005	PUBMED	off topic	no

the effectiveness of a deep learning model to detect left ventricular systolic dysfunction from electrocardiograms	2021	10.1536/ihj.21-407	PUBMED	duplicate	no
the feasibility of arrhythmias detection from a capacitive ecg measurement using convolutional neural network	2019	10.1109/embc.2019.8856867	PUBMED	off topic	no
the groningen electrocardiographic criteria for left ventricular hypertrophy: a sex-specific analysis	2021	10.1038/s41598-021-83137-9	PUBMED	duplicate	no
the hf-cgm study: an analysis of cardiogoniometric axes in patients with cardiac resynchronization therapy	2018	10.1109/tbme.2017.2769060	PUBMED	off topic	no
the hidden waves in the ecg uncovered revealing a sound automated interpretation method	2021	10.1038/s41598-021-82520-w	PUBMED	off topic	no
the impact of torso signal processing on noninvasive electrocardiographic imaging reconstructions	2021	10.1109/tbme.2020.3003465	PUBMED	off topic	no
the in-ear region as a novel anatomical site for ecg signal detection: validation study on healthy volunteers	2021	10.1007/s10840-020-00709-x	PUBMED	duplicate	no
the precordial r' wave: a novel discriminator between cardiac sarcoidosis and arrhythmogenic right ventricular cardiomyopathy in patients presenting with ventricular tachycardia	2021	10.1016/j.hrthm.2021.04.032	PUBMED	off topic	no
the r″ wave in v1 and the negative terminal qrs vector in avf combine to a novel 12-lead ecg algorithm to identify slow conducting anatomical isthmus 3 in patients with tetralogy of fallot	2023	10.1093/europace/euad139	PUBMED	off topic	no
the relation of blood lead and qrs-t angle in american adults	2019	10.1080/19338244.2018.1488674	PUBMED	duplicate	no
the relationship between fragmented qrs and functional significance of coronary lesions	2017	10.1016/j.jelectrocard.2017.01.005	PUBMED	off topic	no
the relationship between fragmented qrs and myocardial injury in patients with acute carbon monoxide poisoning	2024	10.3390/medicina60060891	PUBMED	off topic	no
the relationship between lymphocyte dna damage, coronary artery disease, and blood trace elements	2024	10.1093/mutage/gead030	PUBMED	off topic	no
the relationship between qrs-t angle and left ventricular global longitudinal strain in prehypertensive patients	2024	10.1016/j.jelectrocard.2024.04.010	PUBMED	off topic	no

the relationship between severe acute respiratory syndrome coronavirus 2 (sars - cov - 2) pandemic and fragmented qrs	2020	10.1016/ j.jelectrocard.2020.07.009	PUBMED	off topic	no
the role of baseline and post-treatment frontal qrs-t angle for detecting arterial blood pressure control	2021	10.1080/10641963.2021.1890763	PUBMED	off topic	no
the role of echocardiography and 99mtc-hdp scintigraphy in non-invasive diagnosis of cardiac amyloidosis: a case series and literature review	2019	10.1097/ md.0000000000017256	PUBMED	off topic	no
the role of heart rate variability and fragmented qrs for determination of subclinical cardiac involvement in beta-thalassemia major	2020	10.1159/000505556	PUBMED	off topic	no
the selvester qrs score as an estimative of myocardial injury in acute chagasic patients from the brazilian amazon	2021	10.1186/s12879-021-06083-x	PUBMED	off topic	no
the specification of zebrafish (danio rerio) heart electrocardiogram index characteristic responses to different types of pollutants	2021	10.1016/ j.chemosphere.2020.129199	PUBMED	animal	no
the standardized 12-lead fetal electrocardiogram of the healthy fetus in mid-pregnancy: a cross-sectional study	2020	10.1371/ journal.pone.0232606	PUBMED	duplicate	no
the use of machine learning algorithms in the evaluation of the effectiveness of resynchronization therapy	2022	10.3390/ jcdd9010017	PUBMED	off topic	no
the utility of electrocardiography and echocardiography in copper deficiency-induced cardiac damage in goats	2021	10.1007/s11356-020-11014-5	PUBMED	duplicate	no
the value and applicability of the electrocardiography in revealing the cardiac involvement of covid-19 patients	2021	10.5455/ aim.2021.29.253-259	PUBMED	duplicate	no
the δwaveecg: the differences to the normal 12-lead ecg amplitudes	2023	10.1016/ j.jelectrocard.2022.10.014	PUBMED	duplicate	no
three-dimensional vectorcardiographic characteristics of breast cancer patients treated with chemotherapy	2021	10.1016/ j.jelectrocard.2021.04.018	PUBMED	off topic	no
time course of electrical remodeling of native conduction after cardiac resynchronization therapy and its impact on clinical outcome	2017	10.1016/ j.cardfail.2016.10.014	PUBMED	off topic	no

toward ecg-based analysis of hypertrophic cardiomyopathy: a novel ecg segmentation method for handling abnormalities	2022	10.1093/jamia/ocac122	PUBMED	off topic	no
towards accurate search for neonatal heartbeat: weighted algorithm for reliable ecg analysis of premature infants	2024	10.3233/shti230960	PUBMED	off topic	no
towards end-to-end ecg classification with raw signal extraction and deep neural networks	2019	10.1109/jbhi.2018.2871510	PUBMED	duplicate	no
transcription factor meis1 act as a new regulator of ischemic arrhythmias in mice	2022	10.1016/j.jare.2021.11.004	PUBMED	duplicate	no
transfer learning in ecg classification from human to horse using a novel parallel neural network architecture	2020	10.1038/s41598-019-57025-2	PUBMED	animal	no
unveiling cardiac insights: exploring early left ventricular dysfunction in polycythemia vera through 4d-ste and fragmented qrs analysis	2023	10.1111/echo.15715	PUBMED	off topic	no
update on the ecg component of the cipa initiative	2018	10.1016/j.jelectrocard.2018.08.003	PUBMED	duplicate	no
use of a transformed ecg signal to detect respiratory effort during apnea	2019	10.5664/jcsm.7880	PUBMED	duplicate	no
use of electrocardiographic screening to clear athletes for return to sports following covid-19 infection	2021	10.1016/j.mayocpiqo.2021.01.007	PUBMED	off topic	no
use of the wearable cardioverter-defibrillator (wcd) and wcd-based remote rhythm monitoring in a real-life patient cohort	2018	10.1007/s00380-018-1181-x	PUBMED	off topic	no
usefulness of routine transtelephonic monitoring for supraventricular tachycardia in infants	2018	10.1016/j.jpeds.2017.10.014	PUBMED	duplicate	no
user perceptions and experiences of a handheld 12-lead electrocardiographic device in a clinical setting: usability evaluation	2021	10.2196/21186	PUBMED	off topic	no
using the redundant convolutional encoder-decoder to denoise qrs complexes in ecg signals recorded with an armband wearable device	2020	10.3390/s20164611	PUBMED	denoising	no
utility of a novel wearable electrode embedded in an undershirt for electrocardiogram monitoring and detection of arrhythmias	2022	10.1371/journal.pone.0273541	PUBMED	hardware	no

utility of surveillance ambulatory rhythm monitoring in the pediatric fontan population	2021	10.1007/s00246-021-02630-0	PUBMED	off topic	no
validation of a simple electrocardiographic algorithm for detection of ventricular tachycardia	2021	10.36660/abc.20190501	PUBMED	off topic	no
validation of an automatic diagnosis of strict left bundle branch block criteria using 12-lead electrocardiograms	2017	10.1111/anec.12398	PUBMED	duplicate	no
value of a novel 16-lead high-definition ecg machine to detect conduction abnormalities in structural heart disease	2018	10.1111/pace.13338	PUBMED	duplicate	no
vectorcardiographic qrs area as a predictor of response to cardiac resynchronization therapy	2022	10.11909/j.issn.1671-5411.2022.01.003	PUBMED	duplicate	no
ventricular dyssynchrony assessment using ultra-high frequency ecg technique	2017	10.1007/s10840-017-0268-0	PUBMED	duplicate	no
ventricular late potentials and myocardial fibrosis in hypertrophic cardiomyopathy	2020	10.1016/j.jelectrocard.2019.10.003	PUBMED	off topic	no
verb: vfcdm-based electrocardiogram reconstruction and beat detection algorithm	2019	10.1109/access.2019.2894092	PUBMED	duplicate	no
visual saliency detection approach for long-term ecg analysis	2022	10.1016/j.cmpb.2021.106518	PUBMED	duplicate	no
watch out for st-elevation myocardial infarction: a case report of st-elevation in single-lead electrocardiogram tracing of a smartwatch	2020	10.1093/ehjcr/ytaa353	PUBMED	duplicate	no
wave intensity of aortic root pressure as diagnostic marker of left ventricular systolic dysfunction	2017	10.1371/journal.pone.0179938	PUBMED	off topic	no
wavelet denoising as a post-processing enhancement method for non-invasive foetal electrocardiography	2020	10.1016/j.cmpb.2020.105558	PUBMED	fetal	no
wearable system for biosignal acquisition and monitoring based on reconfigurable technologies	2019	10.3390/s19071590	PUBMED	hardware	no
why -avf can be used in stan as a proxy for scalp electrode-derived signal; reply to comments by kjellmer et al	2019	10.1371/journal.pone.0221220	PUBMED	off topic	no
zika virus infection downregulates connexin 43, disrupts the cardiomyocyte gap junctions and induces heart diseases in a129 mice	2022	10.1128/jvi.01373-22	PUBMED	off topic	no

2nd international conference on mechanical, electronics, computer, and industrial technology	2019		SCOPUS	off topic	no
3.6 mw active-electrode ecg/eti sensor system using wideband low-noise instrumentation amplifier and high impedance balanced current driver	2023	10.3390/s23052536	SCOPUS	hardware	no
a 2.2 nw analog electrocardiogram processor based on stochastic resonance achieving a 99.94% qrs complex detection sensitivity	2023	10.1109/ tbcas.2023.3235786	SCOPUS	hardware	no
a 3d-cnt micro-electrode array for zebrafish ecg study including directionality measurement and drug test	2020	10.1016/ j.bbe.2020.02.008	SCOPUS	animal	no
a 746 nw ecg processor asic based on ternary neural network	2022	10.1109/ tbcas.2022.3196059	SCOPUS	hardware	no
a brief review of computation techniques for ecg signal analysis	2022	10.1007/978-981-16-7597-3_18	SCOPUS	review	no
a brief review of computation techniques for ecg signal analysis	2022	10.1007/978-981-16-7597-3_18	SCOPUS	duplicate	no
a channel-dependent algorithm for heart beats detection in ecg recordings	2017	10.23919/ cisti.2017.7975975	SCOPUS	multichannel	yes
a comparison of three ecg-derived respiration methods for sleep apnoea detection	2019	10.1088/2057-1976/aafc80	SCOPUS	off topic	no
a comprehensive qrs detection method based on exclusive mother wavelet and artificial neural network	2022	10.4015/ s1016237222500144	SCOPUS	duplicate	no
a comprehensive qrs detection method based on exclusive mother wavelet and artificial neural network	2022	10.4015/ s1016237222500144	SCOPUS	not accessible	no
a crucial wave detection and delineation method for twelve-lead ecg signals	2020	10.1109/ access.2020.2965334	SCOPUS	off topic	no
a decision support system and rule-based algorithm to augment the human interpretation of the 12-lead electrocardiogram	2017	10.1016/ j.jelectrocard.2017.08.007	SCOPUS	multichannel	no
a deep learning algorithm for detecting acute pericarditis by electrocardiogram	2022	10.3390/ jpm12071150	SCOPUS	off topic	no
a deep learning algorithm to detect anaemia with ecgs: a retrospective, multicentre study	2020	10.1016/s2589-7500(20)30108-4	SCOPUS	off topic	no

a deep learning approach for atrial fibrillation classification using multi-feature time series data from ecg and ppg	2023	10.3390/diagnostics13142442	SCOPUS	off topic	no
a deep learning architecture using 3d vectorcardiogram to detect r-peaks in ecg with enhanced precision	2023	10.3390/s23042288	SCOPUS	multichannel	yes
a deep learning framework for noninvasive fetal ecg signal extraction	2024	10.3389/fphys.2024.1329313	SCOPUS	fetal	no
a deep neural network approach to qrs detection using autoencoders	2021	10.1016/j.eswa.2021.115528	SCOPUS	INCLUDED	yes
a detection system of exercise electrocardiogram [um sistema de detecção de eletrocardiograma de exercício] [un sistema de detección de electrocardiograma de ejercicio]	2021	10.1590/1517-8692202127042021_0122	SCOPUS	off topic	no
a different view on artificial intelligence applications for cardiac arrhythmia detection and classification	2022	10.1007/978-3-030-82529-4_41	SCOPUS	off topic	no
a feasible qrs detection algorithm for arrhythmia diagnosis	2017	10.1109/icaees.2016.7888004	SCOPUS	INCLUDED	yes
a four-lead real time arrhythmia analysis algorithm	2017	10.22489/cinc.2017.214-182	SCOPUS	multichannel	no
a greedy graph search algorithm based on changepoint analysis for automatic qrs complex detection	2021	10.1016/j.compbiomed.2021.104208	SCOPUS	off topic	no
a low-complexity r-peak detection based on exponential weight mean-variance for wearable ecg devices	2023	10.1109/atc58710.2023.10318901	SCOPUS	INCLUDED	yes
a low-power dry electrode-based ecg signal acquisition with de-noising and feature extraction	2022	10.1007/s11265-021-01681-z	SCOPUS	off topic	no
a machine-learning approach for detection and quantification of qrs fragmentation	2019	10.1109/jbhi.2018.2878492	SCOPUS	off topic	no
a mixed approach for fetal qrs complex detection	2019	10.1007/978-981-13-2288-4_38	SCOPUS	fetal	no
a modeling and machine learning approach to ecg feature engineering for the detection of ischemia using pseudo-ecg	2019	10.1371/journal.pone.0220294	SCOPUS	off topic	no
a modified algorithm for qrs complex detection for fpga implementation	2018	10.1007/s00034-017-0711-6	SCOPUS	hardware	no

a new approach to cardiovascular screening in newborn	2020	10.1007/978-3-030-30648-9_62	SCOPUS	off topic	no
a new deep learning algorithm of 12-lead electrocardiogram for identifying atrial fibrillation during sinus rhythm	2021	10.1038/s41598-021-92172-5	SCOPUS	multichannel	no
a new method of detecting the characteristic waves and their onset and end in electrocardiogram signals	2022	10.1016/j.bspc.2022.103607	SCOPUS	INCLUDED	yes
a new methodology for classifying qrs morphology in ecg signals	2020	10.1109/ijcnn48605.2020.9206707	SCOPUS	off topic	no
a novel and lightweight p, qrs, and t peaks detector using adaptive thresholding and template waveform	2021	10.1016/j.compbiomed.2021.104307	SCOPUS	INCLUDED	yes
a novel approach for denoising electrocardiogram signals to detect cardiovascular diseases using an efficient hybrid scheme	2024	10.3389/fcvm.2024.1277123	SCOPUS	denoising	no
a novel approach to the extraction of fetal electrocardiogram based on empirical mode decomposition and correlation analysis	2017	10.1007/s13246-017-0560-4	SCOPUS	fetal	no
a novel deep arrhythmia-diagnosis network for atrial fibrillation classification using electrocardiogram signals	2019	10.1109/access.2019.2918792	SCOPUS	off topic	no
a novel ecg enhancement and qrs detection scheme based on the 1-d high-order non-convex total variation denoising	2023	10.1007/s00034-023-02357-8	SCOPUS	denoising	yes
a novel hybrid deep learning method with cuckoo search algorithm for classification of arrhythmia disease using ecg signals	2021	10.1007/s00521-021-06005-7	SCOPUS	off topic	no
a novel machine learning approach to classify and detect atrial fibrillation using optimized implantable electrocardiogram sensor	2021	10.1109/access.2021.3123367	SCOPUS	off topic	no
a novel method based on adaptive periodic segment matrix and singular value decomposition for removing emg artifact in ecg signal	2020	10.1016/j.bspc.2020.102060	SCOPUS	off topic	no
a novel method for fetal ecg extraction using ica based wavelet transform	2023	10.14445/22315381/ijett-v71i8p211	SCOPUS	fetal	no

a novel method for the detection of qrs complex using vectorcardiographic octants	2021	10.23919/ cinc53138.2021.966 2893	SCOPUS	off topic	no
a novel modular fetal ecg stan and hrv analysis: towards robust hypoxia detection	2019	10.3233/thc-181375	SCOPUS	fetal	no
a novelwearable device for continuous ambulatory ecg recording: proof of concept and assessment of signal quality	2019	10.3233/thc-181375	SCOPUS	off topic	no
a programmed cardiac arrhythmia analysis system by adaptive cardiac outlining	2023	10.1007/s42979-023-01891-1	SCOPUS	off topic	no
a real time qrs detection algorithm based on et and pd controlled threshold strategy	2020	10.3390/s20144003	SCOPUS	INCLUDED	yes
a real-time embedded system to detect qrs-complex and arrhythmia classification using lstm through hybridized features	2023	10.1016/ j.eswa.2022.119221	SCOPUS	hardware	yes
a real-time qrs complex detector based on discrete wavelet transform and adaptive threshold as standalone application on arm microcontrollers	2018	10.1109/ icbea.2018.8471741	SCOPUS	INCLUDED	yes
a real-time qrs detection method based on phase portraits and box-scoring calculation	2018	10.1109/ jsen.2018.2812792	SCOPUS	hardware	no
a real-time qrs detection system with pr/rt interval and st segment measurements for wearable ecg sensors using parallel delta modulators	2018	10.1109/ tbcas.2018.2823275	SCOPUS	off topic	no
a review of methods for myocardial infarction detection using of electrocardiographic features	2019	10.1063/1.5141711	SCOPUS	off topic	no
a review on computational methods for denoising and detecting ecg signals to detect cardiovascular diseases	2021	10.1007/s11831-021-09642-2	SCOPUS	review	no
a robust qrs complex detection method based on shannon energy envelope and hilbert transform	2022	10.1142/ s021951942240013 9	SCOPUS	INCLUDED	yes
a screening method for predicting left ventricular dysfunction based on spectral analysis of a single-channel electrocardiogram using machine learning algorithms	2023	10.1016/ j.bspc.2023.105219	SCOPUS	off topic	no
a secure fuzzy extractor based biometric key authentication scheme for body sensor network in internet of medical things	2020	10.1016/ j.comcom.2020.01.0 77	SCOPUS	off topic	no

a single-lead ecg based cardiotoxicity detection in patients on polychemotherapy	2024	10.1016/j.ijcha.2024.101336	SCOPUS	off topic	no
a study on the optimization of ecg qrs detection using the hidden markov model	2019	10.5370/kiee.2019.68.11.1425	SCOPUS	language not supported	no
a system for disease identification using ecg and other variables	2020	10.1007/978-981-15-3172-9_47	SCOPUS	off topic	no
a system to study the evolution of cardiac arrhythmias at home	2017	10.1007/978-981-10-4086-3_1	SCOPUS	off topic	no
a watch-type electrocardiography is a reliable tool for detecting paroxysmal cardiac arrhythmias	2022	10.3390/jcm11123333	SCOPUS	off topic	no
abnormal beat detection from unreconstructed compressed signals based on linear approximation in ecg signals suitable for embedded iot devices	2022	10.1007/s12652-021-03578-y	SCOPUS	off topic	no
abnormal blood lipid and electrocardiogram characteristics in common mental disorders	2023	10.1186/s12888-023-04965-9	SCOPUS	off topic	no
accidentally 1st author developed early stage of cancers within several hours after drinking hpv-16 & hhv-8 infected eggnog & pie with bacterial infection, which created severe abdominal pain & diarrhea. but, succeeded in completely eliminating cancer activities of potentially infectious cancer by combination of 3 methods of treatment which were discovered recently for hopelessly advanced cancer patients	2018	10.3727/036012919x15475948072791	SCOPUS	off topic	no
accurate detection of atrial fibrillation from 12-lead ecg using deep neural network	2020	10.1016/j.compbiomed.2019.103378	SCOPUS	multichannel	no
acute high level noise exposure can cause physiological dysfunction in macaque monkeys: insight on the medical protection for special working environmental personnel	2021	10.3390/healthcare9070840	SCOPUS	off topic	no
acute osimertinib exposure induces electrocardiac changes by synchronously inhibiting the currents of cardiac ion channels	2023	10.3389/fphar.2023.1177003	SCOPUS	off topic	no
adaptive neuro-fuzzy inference healthcare system for cardiac arrhythmia detection using heart rate variability features	2021	10.1007/978-981-15-8221-9_201	SCOPUS	off topic	no

adaptive noise reduction of multichannel signals obtained by capacitive ecg sensors	2018	10.1109/ icfsp.2018.8552062	SCOPUS	denoising	no
adaptive r-peak detection on wearable ecg sensors for high-intensity exercise	2023	10.1109/ tbme.2022.3205304	SCOPUS	inaccessible dataset	yes
adaptive r-peak detector in extreme noise using emd selective analyzer	2022	10.1109/ memea54994.2022. 9856531	SCOPUS	inaccessible dataset	yes
adaptive threshold, wavelet and hilbert transform for qrs detection in electrocardiogram signals	2018	10.1007/978-3-319- 69835-9_73	SCOPUS	not accessible	no
advanced time-frequency methods for ecg waves recognition	2023	10.3390/ diagnostics1302030 8	SCOPUS	off topic	no
afcnnet: automated detection of af using chirplet transform and deep convolutional bidirectional long short term memory network with ecg signals	2021	10.1016/ j.compbiomed.2021. 104783	SCOPUS	off topic	no
aiding the detection of qrs complex in ecg signals by detecting s peaks independently	2018	10.1007/s13239- 018-0355-0	SCOPUS	duplicate	no
amplitude and frequency based evaluations for algorithm development of premature ventricular contraction detection system	2022	10.1109/ icoris56080.2022.10 031562	SCOPUS	off topic	no
an accurate qrs complex and p wave detection in ecg signals using complete ensemble empirical mode decomposition with adaptive noise approach	2019	10.1109/ access.2019.293994 3	SCOPUS	INCLUDED	yes
an analysis of the effects of noisy electrocardiogram signal on heartbeat detection performance	2020	10.3390/ bioengineering70200 53	SCOPUS	off topic	no
an application of zero cross qrs detection algorithm of ecg signals with various subject conditions	2018	10.1109/ incae.2018.8579419	SCOPUS	inaccessible dataset	no
an approach of cardiac disease prediction by analyzing ecg signal	2017	10.1109/ ceeict.2016.7873093	SCOPUS	off topic	no
an area and power efficient vlsi architecture to detect obstructive sleep apnea for wearable devices	2022	10.1109/ radioelektronika5453 7.2022.9764917	SCOPUS	off topic	no
an area and power efficient vlsi architecture to detect obstructive sleep apnea for wearable devices	2022	10.1109/ radioelektronika5453 7.2022.9764917	SCOPUS	off topic	no

an arrhythmia classification approach via deep learning using single-lead ecg without qrs wave detection	2024	10.1016/j.heliyon.2024.e27200	SCOPUS	off topic	no
an artificial intelligence mediated integrated wearable device for diagnosis of cardio through remote monitoring	2021	10.1016/b978-0-12-824410-4.00008-8	SCOPUS	off topic	no
an automatic detection of arrhythmia disease diagnosis system based on artificial neural network and support vector machine	2020	10.1109/compe49325.2020.9200140	SCOPUS	off topic	no
an ecg arrhythmia image classification system based on convolutional neural network	2020	10.1088/1742-6596/1544/1/012109	SCOPUS	off topic	no
an ecg delineation and arrhythmia classification system using slope variation measurement by ternary second-order delta modulators for wearable ecg sensors	2021	10.1109/tbcas.2021.3113665	SCOPUS	off topic	no
an efficient and robust deep learning method with 1-d octave convolution to extract fetal electrocardiogram	2020	10.3390/s20133757	SCOPUS	fetal	no
an efficient and robust digital fractional order differentiator based ecg pre-processor design for qrs detection	2019	10.1109/tbcas.2019.2916676	SCOPUS	does not introduce a new technique	yes
an efficient architecture for qrs detection in fpga using integer haar wavelet transform	2020	10.1007/s00034-019-01328-2	SCOPUS	hardware	no
an efficient wavelet-based automated r-peaks detection method using hilbert transform	2017	10.1016/j.bbe.2017.02.002	SCOPUS	INCLUDED	yes
an efficient wavelet-based feature extraction scheme for electrocardiogram signals	2017	10.1109/wits.2017.7934670	SCOPUS	off topic	no
an energy-efficient analog circuit for detecting qrs complexes from ecg signal	2023	10.1016/j.vlsi.2022.11.001	SCOPUS	hardware	no
an enhanced t-wave delineation method using phasor transform in the electrocardiogram	2021	10.1088/2057-1976/ac0502	SCOPUS	off topic	no
an ensemble of machine learning models for multilabel classification of cardiovascular diseases by ecgs	2023	10.22489/cinc.2023.258	SCOPUS	hardware	no
an image processing approach for compression of ecg signals based on 2d rle and spiht	2019	10.1007/978-981-13-5802-9_86	SCOPUS	off topic	no
an improved iterative pace-mapping algorithm to detect the origin of premature ventricular contractions	2020	10.22489/cinc.2020.062	SCOPUS	off topic	no
an improved method to detect arrhythmia using ensemble learning-based model in multi lead electrocardiogram (ecg)	2024	10.1371/journal.pone.0297551	SCOPUS	off topic	no

an innovative machine learning approach for classifying ecg signals in healthcare devices	2022	10.1155/2022/7194419	SCOPUS	off topic	no
an iomt enabled deep learning framework for automatic detection of fetal qrs: a solution to remote prenatal care	2022	10.1016/j.jksuci.2022.07.002	SCOPUS	fetal	no
an iot framework for detecting cardiac arrhythmias in real-time using deep learning resnet model	2023	10.1016/j.measen.2023.100866	SCOPUS	off topic	no
an objective tool for quantifying atrial fibrillation substrate in rats	2023	10.1152/ajpheart.00728.2022	SCOPUS	animal	no
an octave convolution neural network-based qrs detector	2020	10.1109/icsmd50554.2020.9261658	SCOPUS	INCLUDED	yes
analysis of cnn and feed-forward ann model for the evaluation of ecg signal	2023	10.2174/1574362417666220328144453	SCOPUS	not accessible	no
analysis of different heart rate monitoring and pre-processing techniques for ecg	2019	10.1109/icnte44896.2019.8946042	SCOPUS	off topic	no
analysis of ecg arrhythmia for heart disease detection using svm and cuckoo search optimized neural network	2018	10.14419/ijet.v7i2.17.11553	SCOPUS	off topic	no
analysis of qrs detection algorithms barely sensitive to the qrs shape	2017	10.1109/eiconrus.2017.7910663	SCOPUS	inaccessible dataset	no
analysis of sampling frequency and resolution in ecg signals	2018	10.1109/telfor.2017.8249438	SCOPUS	off topic	no
analyzing long-term electrocardiography recordings to detect arrhythmias in mice	2021	10.3791/62386	SCOPUS	animal	no
apnoea detection using ecg signal based on machine learning classifiers and its performances	2023	10.1080/03091902.2024.2336500	SCOPUS	off topic	no
application of savitzky-golay digital differentiator for qrs complex detection in an electrocardiographic monitoring system	2017	10.1109/memea.2017.7985881	SCOPUS	inaccessible dataset	no
approxbiowear: approximating additions for efficient biomedical wearable computing at the edge	2021	10.1109/embc46164.2021.9630165	SCOPUS	off topic	no
arrhythmia analysis in the long-term electrocardiogram monitoring system	2023	10.22266/ijies2023.1031.25	SCOPUS	off topic	no

arrhythmia classification using mahalanobis distance based improved fuzzy c-means clustering for mobile health monitoring systems	2017	10.1016/ j.neucom.2016.08.04 2	SCOPUS	off topic	no
arrhythmia detection and classification by using modified recurrent neural network	2022	10.32604/ iasc.2022.023924	SCOPUS	duplicate	no
arrhythmia detection and classification by using modified recurrent neural network	2022	10.32604/ iasc.2022.023924	SCOPUS	off topic	no
arrhythmia detection by using chaos theory with machine learning algorithms	2024	10.32604/ cmc.2023.039936	SCOPUS	off topic	no
arrhythmia detection using tqwt, ceemd and deep cnn-lstm neural networks with ecg signals	2023	10.1007/s11042-022-14227-7	SCOPUS	off topic	no
arrhythmia recognition and classification using combined parametric and visual pattern features of ecg morphology	2020	10.1109/ access.2020.297925 6	SCOPUS	off topic	no
arrhythmias prediction using an hybrid model based on convolutional neural network and nonlinear regression	2020	10.1142/ s146902682050024 8	SCOPUS	off topic	no
artificial intelligence assessment for early detection and prediction of renal impairment using electrocardiography	2022	10.1007/s11255-022-03165-w	SCOPUS	off topic	no
artificial intelligence-assisted electrocardiography for early diagnosis of thyrotoxic periodic paralysis	2021	10.1210/jendso/ bvab120	SCOPUS	off topic	no
artificial intelligence-based electrocardiographic biomarker for outcome prediction in patients with acute heart failure: prospective cohort study	2024	10.2196/52139	SCOPUS	off topic	no
artificial intelligence-based identification of left ventricular systolic dysfunction from 12-lead electrocardiograms: external validation and advanced application of an existing model	2024	10.1093/ehjdh/ ztad081	SCOPUS	off topic	no
artificial intelligence-based opportunistic screening for the detection of arterial hypertension through ecg signals	2022	10.1097/ hjh.0000000000003 286	SCOPUS	off topic	no
artificial intelligence-enhanced electrocardiogram analysis for identifying cardiac autonomic neuropathy in patients with diabetes	2024	10.1111/dom.15578	SCOPUS	off topic	no
artificial intelligence-enhanced smartwatch ecg for heart failure-reduced ejection fraction detection by generating 12-lead ecg	2022	10.3390/ diagnostics1203065 4	SCOPUS	multichannel	no

artificial neural networks for ecg interpretation in acute coronary syndrome: a scoping review	2024	10.1016/ j.ajem.2024.06.026	SCOPUS	off topic	no
assertion-based verification technique for ecg bio-sensor algorithms	2017	10.1109/ mwscas.2016.78700 77	SCOPUS	off topic	no
assessment of delayed ventricular activation after myocardial infarction	2019	10.1016/ j.bspc.2018.10.009	SCOPUS	off topic	no
association of silent myocardial infarction and sudden cardiac death	2019	10.1001/ jamacardio.2019.221 0	SCOPUS	off topic	no
atrial fibrillation detection during sepsis: study on mimic iii icu data	2020	10.1109/ jbhi.2020.2995139	SCOPUS	off topic	no
atrial fibrillation detection using a feedforward neural network	2022	10.1007/s40846-022-00681-z	SCOPUS	missing technical details	yes
atrial fibrillation detection with an analog smartwatch: prospective clinical study and algorithm validation	2022	10.2196/37280	SCOPUS	off topic	no
automated algorithm for qrs detection in cardiac arrest patients with pea	2022	10.22489/ cinc.2022.270	SCOPUS	inaccessible dataset	yes
automated detection of arrhythmias using different intervals of tachycardia ecg segments with convolutional neural network	2017	10.1016/ j.ins.2017.04.012	SCOPUS	off topic	no
automated detection of cardinal points of ecg signal for feature extraction using a single median filter	2022	10.1007/s40031-022-00754-2	SCOPUS	INCLUDED	yes
automated detection of left bundle branch block from ecg signal utilizing the maximal overlap discrete wavelet transform with anfis	2022	10.3390/ computers11060093	SCOPUS	does not introduce a new technique	yes
automated extraction of fetal ecg signal features using twinned filter and integrated methodologies	2024	10.1007/s00034-023-02494-0	SCOPUS	fetal	no
automated interpretable detection of myocardial infarction fusing energy entropy and morphological features	2019	10.1016/ j.cmpb.2019.03.012	SCOPUS	off topic	no
automated qrs complex detection using mfo-based dfod	2018	10.1049/iet-spr.2018.5230	SCOPUS	INCLUDED	yes
automatic detection of arrhythmia from imbalanced ecg database using cnn model with smote	2019	10.1007/s13246-019-00815-9	SCOPUS	off topic	no
automatic ecg signals recognition based on time domain features extraction using fiducial mean square algorithm	2019	10.1007/978-981-13-1132-1_7	SCOPUS	off topic	no

automatic electrocardiogram detection and classification using bidirectional long short-term memory network improved by bayesian optimization	2022	10.1016/ j.bspc.2021.103424	SCOPUS	off topic	no
automatic electrocardiogram sensing classifier based on improved backpropagation neural network	2020	10.18494/ sam.2020.2804	SCOPUS	off topic	no
automatic identification of premature ventricular contraction using ecgs	2019	10.1007/978-3-030-32962-4_14	SCOPUS	off topic	no
automatic qrs onset detection of ecg signal using secant line slope formula	2019	10.1109/ cspa.2019.8695982	SCOPUS	inaccessible dataset	no
automatic qrs-complex peak detector based on moving average and thresholding	2019	10.1088/1742-6596/1153/1/012039	SCOPUS	inaccessible dataset	no
automation algorithm to detect and quantify electrocardiogram waves and intervals	2019	10.1016/ j.procs.2019.04.131	SCOPUS	off topic	no
autonomic activity and cardiovascular system risk assessment in pediatric patients with hemolytic uremic syndrome	2024	10.1007/s00431-024-05420-x	SCOPUS	off topic	no
autonomous detection of myocarditis based on the fusion of improved quantum genetic algorithm and adaptive differential evolution optimization back propagation neural network	2023	10.1007/s13755-023-00237-8	SCOPUS	off topic	no
bayesian real-time qrs complex detector for healthcare system	2019	10.1109/ jiot.2019.2903530	SCOPUS	missing accuracy	no
bioluminescent sport horse saliva test: prospects for use [биолюминесцентное тестирование слюны спортивных лошадей: перспективы использования]	2021	10.15389/ agrobiology.2021.6.1199eng	SCOPUS	off topic	no
body surface potential mapping detects early disease onset in plakophilin-2-pathogenic variant carriers	2023	10.1093/europace/ euad197	SCOPUS	off topic	no
building an automatic arrhythmia detection software based on matlab	2018	10.1007/978-981-10-4361-1_102	SCOPUS	off topic	no
cardiac arrhythmia detection through ecg signals	2018	10.1109/ i2ct42659.2018.9057836	SCOPUS	off topic	no
cardiac arrhythmia detection using dual-tree wavelet transform and convolutional neural network	2022	10.1007/s00500-021-06653-w	SCOPUS	off topic	no
cardiac arrhythmia, chf, and nsr classification with nca-based feature fusion and svm classifier	2023	10.4018/ijsi.315659	SCOPUS	off topic	no

cardiac mri may not detect myocardial ischemia and fibrosis in dogs with cardiomegaly secondary to myxomatous mitral valve disease	2022	10.2460/ ajvr.22.05.0076	SCOPUS	off topic	no
cardiacwave: a mmwave-based scheme of non-contact and high-definition heart activity computing	2021	10.1145/3478127	SCOPUS	off topic	no
cardiovascular disease prediction among the malaysian cohort participants using electrocardiogram	2022	10.32604/ cmc.2022.022123	SCOPUS	inaccessible dataset	yes
category: cardiovascular comparative assessment of electrocardiographic parameters of some birds in ilorin	2019		SCOPUS	off topic	no
characterizing the transient electrocardiographic signature of ischemic stress using laplacian eigenmaps for dimensionality reduction	2020	10.1016/ j.compbiomed.2020. 104059	SCOPUS	off topic	no
cineecg for visualization of changes in ventricular electrical activity during ischemia	2024	10.1016/ j.jelectrocard.2024.0 1.007	SCOPUS	off topic	no
cineecg: a novel method to image the average activation sequence in the heart from the 12-lead ecg	2022	10.1016/ j.compbiomed.2021. 105128	SCOPUS	multichannel	no
circulation assessment by automated external defibrillators during cardiopulmonary resuscitation	2018	10.1016/ j.resuscitation.2018. 04.036	SCOPUS	off topic	no
classification of electrocardiogram signals with waveform morphological analysis and support vector machines	2022	10.1007/s11517- 021-02461-4	SCOPUS	off topic	no
classification of heart rhythm disorders using instructive features and artificial neural networks	2018	10.1504/ ijmei.2018.095085	SCOPUS	off topic	no
classification of ischemic and non-ischemic cardiac events in holter recordings based on the continuous wavelet transform	2020	10.1007/s11517- 020-02134-8	SCOPUS	off topic	no
classification of normal and abnormal ecg signals based on their pqrst intervals	2017	10.1109/ icmsc.2017.7959507	SCOPUS	off topic	no
classification of premature ventricular contraction using error back-propagation	2018	10.3837/ tiis.2018.02.028	SCOPUS	off topic	no
classification of qrs complexes to detect premature ventricular contraction using machine learning techniques	2022	10.1371/ journal.pone.026855 5	SCOPUS	off topic	no

clinical applicability of an artificial intelligence prediction algorithm for early prediction of non-persistent atrial fibrillation	2023	10.3389/ fcvm.2023.1168054	SCOPUS	off topic	no
cnn-based two step r peak detection method: combining segmentation and regression	2022	10.1109/ embc48229.2022.98 71227	SCOPUS	missing technical details	yes
combined cardiac and respiratory monitoring from a single signal: a case study employing the fantasia database	2023	10.3390/s23177401	SCOPUS	off topic	no
common spatial pattern with deep learning for fetal heart rate monitoring	2022	10.1109/ sips55645.2022.991 9257	SCOPUS	fetal	no
comparative assessment of electrocardiographic parameters of some birds in ilorin-an essential diagnostic tool [evaluación comparativa de los parámetros electrocardiográficos de algunas aves en ilorin - una esencial herramienta de diagnóstico]	2019		SCOPUS	animal	no
comparative study on heart rate variability analysis for atrial fibrillation detection in short single-lead ecg recordings	2018	10.1109/ embc.2018.8512345	SCOPUS	off topic	no
comparing different methods of hand-crafted hrv, edr and cpc features for sleep apnoea detection	2019	10.1109/ embc.2019.8856779	SCOPUS	off topic	no
comparison of 10 qrs detection methods for heart beat detection on portable ecg systems	2020	10.1109/ cenim51130.2020.92 97903	SCOPUS	does not introduce a new technique	no
comparison of acceleration python library on design and implementation of qrs detection module from ecg heart signal	2019	10.1088/1757- 899x/673/1/012055	SCOPUS	off topic	no
complete right bundle branch block and qrs-t discordance can be the initial clue to detect s-icd ineligibility	2017	10.1016/ j.jjcc.2016.11.014	SCOPUS	off topic	no
complex-pan-tompkins-wavelets: cross-channel ecg beat detection and delineation	2021	10.1016/ j.bspc.2021.102450	SCOPUS	multichannel	no
computer aided atrial fibrillation detection from the statistical attributes of ecg signal	2021	10.1109/ icecit54077.2021.96 41198	SCOPUS	off topic	no

computer-aided diagnostic system for feature-based classification using heart rate variability	2020	10.4015/s101623722050009x	SCOPUS	off topic	no
continuous non-invasive estimates of cerebral blood flow using electrocardiography signals: a feasibility study	2023	10.1007/s13534-023-00265-z	SCOPUS	off topic	no
correlation between the electrocardiogram amplitude detected by an implantable cardiac monitor and the implantation depth	2024	10.1111/anec.13102	SCOPUS	off topic	no
current clinical practice of cardiac resynchronization therapy in turkey: reflections from cardiac resynchronization therapy survey-ii	2020	10.14744/anatoljcardiol.2020.02680	SCOPUS	off topic	no
dapagliflozin: a sodium–glucose cotransporter 2 inhibitor, attenuates angiotensin ii-induced atrial fibrillation by regulating atrial electrical and structural remodeling	2024	10.1016/j.ejphar.2024.176712	SCOPUS	off topic	no
data extraction from an electrocardiogram signal and qrs peak detection employing wavelet-based approach	2022	10.1109/incet54531.2022.9824913	SCOPUS	missing technical details	yes
dcda-net: dual-convolutional dual-attention network for obstructive sleep apnea diagnosis from single-lead electrocardiograms	2023	10.1016/j.engappai.2023.106451	SCOPUS	off topic	no
decision tree-based model for signal quality scanning in wearable ecg	2022	10.22489/cinc.2022.121	SCOPUS	off topic	no
deep learning based depression analysis using eeg and ecg signals	2023	10.14445/23488379/ijeee-v10i7p105	SCOPUS	off topic	no
deep learning model to detect significant aortic regurgitation using electrocardiography	2022	10.1016/j.jjcc.2021.08.029	SCOPUS	off topic	no
deep learning models for predicting left heart abnormalities from single-lead electrocardiogram for the development of wearable devices	2024	10.1253/circj.cj-23-0216	SCOPUS	off topic	no
deep learning-mediated prediction of concealed accessory pathway based on sinus rhythmic electrocardiograms	2023	10.1111/anec.13072	SCOPUS	off topic	no
deeparr: an investigative tool for arrhythmia detection using a contextual deep neural network from electrocardiograms (ecg) signals	2023	10.1016/j.bspc.2023.104954	SCOPUS	off topic	no

denoising and beat detection of ecg signal by using fpga	2017	10.1142/ s012915641740016x	SCOPUS	denoising	no
dens-ecg: a deep learning approach for ecg signal delineation	2021	10.1016/ j.eswa.2020.113911	SCOPUS	off topic	no
depression analysis based on eeg and ecg signals	2023	10.1109/ incet57972.2023.10170067	SCOPUS	off topic	no
derivative-based peak detection algorithm for ppa waveforms	2018	10.1109/ iccic.2018.8782379	SCOPUS	off topic	no
design and evaluation of a wearable single-lead ecg for continuous monitoring	2023	10.1109/ metroxraine58569.2023.10405813	SCOPUS	off topic	no
design and implementation of a novel real time p-qrs-t waves detection algorithm	2020	10.1109/ itnec48623.2020.9084924	SCOPUS	denoising	no
design of a biorthogonal wavelet transform based r-peak detection and data compression scheme for implantable cardiac pacemaker systems	2018	10.1007/s10916-018-0953-2	SCOPUS	hardware	yes
design of a web laboratory interface for ecg signal analysis using matlab builder ne	2022	10.1515/comp-2022-0244	SCOPUS	off topic	no
design of an energy detector for heartbeat localization in ecg signals	2017	10.1109/ icecs.2016.7841135	SCOPUS	off topic	no
designing a low-cost real-time group heart rate monitoring system	2018	10.1016/ j.micpro.2018.08.010	SCOPUS	off topic	no
detect qrs complex in ecg	2018	10.1109/ iciea.2017.8283170	SCOPUS	missing dataset information	no
detecting 'strict' left bundle branch block from 12-lead electrocardiogram using support vector machine classification and derivative analysis	2018	10.22489/ cinc.2018.030	SCOPUS	multichannel	no
detecting drug-induced changes in ecg parameters using jacketed telemetry: effect of different data reduction techniques	2017	10.1016/ j.vascn.2016.12.004	SCOPUS	off topic	no
detecting ecg abnormalities using an ensemble framework enhanced by bayesian belief network	2022	10.1016/ j.bspc.2021.103320	SCOPUS	off topic	no

detecting noisy ecg qrs complexes using waveletcnn autoencoder and convlstm	2020	10.1109/access.2020.3012904	SCOPUS	denoising	no
detecting obstructive sleep apnea by extracting multimodal hrv features using ensemble subspace discriminant classifier	2023	10.1504/ijmei.2023.134539	SCOPUS	off topic	no
detection and classification of arrhythmia using an explainable deep learning model	2021	10.1016/j.jelectrocard.2021.06.006	SCOPUS	off topic	no
detection and classification of myocardial infarction with support vector machine classifier using grasshopper optimization algorithm	2021	10.4103/jmss.jmss_24_20	SCOPUS	off topic	no
detection and delineation of the enigmatic u-wave in an electrocardiogram	2021	10.1007/s41870-019-00287-w	SCOPUS	off topic	no
detection of abnormal left ventricular geometry in patients without cardiovascular disease through machine learning: an ecg-based approach	2021	10.1111/jch.14200	SCOPUS	off topic	no
detection of apnea bradycardia from ecg signals of preterm infants using layered hidden markov model	2021	10.1007/s10439-021-02732-z	SCOPUS	off topic	no
detection of cardiovascular autonomic neuropathy using machine learning algorithms	2023		SCOPUS	off topic	no
detection of ecg wave components for the prediction of acute coronary syndrome - brief survey	2024		SCOPUS	off topic	no
detection of electrocardiogram qrs complex based on modified adaptive threshold	2019	10.11591/ijece.v9i5.pp3512-3521	SCOPUS	INCLUDED	yes
detection of fetal ecg r wave from single-lead abdominal ecg using a combination of rr time-series smoothing and template-matching approach	2019	10.1109/access.2019.2917826	SCOPUS	fetal	no
detection of fetal heart rate using anfis displayed on a smartphone	2017	10.1109/tencon.2016.7848269	SCOPUS	fetal	no
detection of heart diseases using cnn-lstm	2023	10.1007/978-3-031-27409-1_45	SCOPUS	off topic	no
detection of myocardial infarction in 12 lead ecg using support vector machine	2018	10.1016/j.asoc.2017.12.001	SCOPUS	multichannel	no

detection of uninterpretable ecg signal segments	2020	10.23919/ mipro48935.2020.92 45427	SCOPUS	off topic	no
development and validation of a deep-learning model to detect crp level from the electrocardiogram	2022	10.3389/ fphys.2022.864747	SCOPUS	off topic	no
development and validation of an algorithm for the digitization of ecg paper images	2022	10.3390/s22197138	SCOPUS	off topic	no
development of a baby incubator with a vital sign monitoring tool based on pan-tompkins method for heart rate calculation	2024	10.1007/978-981-97-1463-6_42	SCOPUS	off topic	no
development of a driver drowsiness monitoring system using electrocardiogram	2018		SCOPUS	off topic	no
development of arrhythmia classification system for personal cardiac monitor in thailand	2019	10.1109/ eltech.2019.8839615	SCOPUS	off topic	no
digital biomarkers and algorithms for detection of atrial fibrillation using surface electrocardiograms: a systematic review: digital biomarkers for af in surface ecgs	2021	10.1016/ j.compbiomed.2021. 104404	SCOPUS	off topic	no
discrete electro cardiogram t amplitude detection based on cycle duration	2023	10.15587/1729-4061.2023.282759	SCOPUS	off topic	no
discriminating cocaine use from other sympathomimetics using wearable electrocardiographic (ecg) sensors	2023	10.1016/ j.drugalcdep.2023.11 0898	SCOPUS	off topic	no
disproportionality analysis of quinolone safety in children using data from the fda adverse event reporting system (faers)	2023	10.3389/ fped.2022.1069504	SCOPUS	off topic	no
doppler cardiogram: a remote detection of human heart activities	2020	10.1109/ tmtt.2019.2948844	SCOPUS	off topic	no
driver drowsiness detection using different classification algorithms	2020	10.1088/1742-6596/1502/1/012037	SCOPUS	off topic	no
dynamic changes in the signal-averaged electrocardiogram are associated with the long-term outcomes after ablation of ischemic ventricular tachycardia	2021	10.1007/s10840-020-00708-y	SCOPUS	off topic	no
early detection of myocardial ischemia in resting ecg: analysis by hht	2023	10.1186/s12938-023-01089-9	SCOPUS	off topic	no

ecg beat classification using empirical mode decomposition and mixture of features	2017	10.1080/03091902.2017.1394386	SCOPUS	INCLUDED	yes
ecg biometric recognition	2018	10.1007/978-981-13-0023-3_7	SCOPUS	off topic	no
ecg changes during adult life in fabry disease: results from a large longitudinal cohort study	2023	10.3390/diagnostics13030354	SCOPUS	off topic	no
ecg compressed sensing method with high compression ratio and dynamic model reconstruction	2021	10.1016/j.measurement.2021.109803	SCOPUS	off topic	no
ecg criteria for the detection of high-risk cardiovascular conditions in master athletes	2020	10.1177/2047487319901060	SCOPUS	off topic	no
ecg derived ventricular gradient exceeds echocardiography in the early detection of pulmonary hypertension in scleroderma patients	2018	10.1016/j.ijcard.2018.07.122	SCOPUS	off topic	no
ecg dry-electrode 3d printing and signal quality considerations	2021	10.1109/embc46164.2021.9630599	SCOPUS	off topic	no
ecg heartbeat signal classification and detection of cardiac abnormalities using deep learning	2023	10.1109/ccpis59145.2023.10291586	SCOPUS	off topic	no
ecg in the clinical and prognostic evaluation of patients with pulmonary arterial hypertension: an underestimated value	2022	10.1177/17534666221087846	SCOPUS	duplicate	no
ecg in the clinical and prognostic evaluation of patients with pulmonary arterial hypertension: an underestimated value	2022	10.1177/17534666221087846	SCOPUS	off topic	no
ecg indices poorly predict left ventricular hypertrophy and are applicable only in individuals with low cardiovascular risk	2020	10.3390/jcm9051364	SCOPUS	off topic	no
ecg marker evaluation for the machine-learning-based classification of acute and chronic phases of trypanosoma cruzi infection in a murine model	2023	10.3390/tropicalmed8030157	SCOPUS	off topic	no
ecg morphological features based sudden cardiac arrest (sca) prediction using nonlinear classifiers	2020	10.1007/978-981-15-5558-9_68	SCOPUS	off topic	no
ecg pqrst complex detector and heart rate variability analysis using temporal characteristics of fiducial points	2021	10.1016/j.bspc.2020.102291	SCOPUS	INCLUDED	yes

ecg processing algorithm in the qrs complex	2021	10.1109/icaacca51523.2021.9465280	SCOPUS	hardware	no
ecg r-r interval automatic detection using iot	2022	10.1109/nkcon56289.2022.10126731	SCOPUS	off topic	no
ecg signal based power aware system for obstructive sleep apnea detection	2017	10.1109/icrteect.2017.43	SCOPUS	off topic	no
ecg signal classification and parameter estimation using multiwavelet transform	2017		SCOPUS	off topic	no
ecg signal classification for the detection of cardiac arrhythmias using a convolutional recurrent neural network	2018	10.1088/1361-6579/aad9ed	SCOPUS	off topic	no
ecg signal denoising and features extraction using unbiased fir smoothing	2019	10.1155/2019/2608547	SCOPUS	denoising	no
ecg signal parameters extraction using intelligent adaptive algorithm	2018	10.1109/sta.2017.8314972	SCOPUS	off topic	no
ecg signal processing and human state detection based on wearable electrodes	2021	10.1088/1742-6596/1952/3/032055	SCOPUS	off topic	no
ecg signals denoising using optimized threshold function using discrete wavelet transform	2023	10.1063/5.0113586	SCOPUS	denoising	no
ecg signals segmentation using deep spatiotemporal feature fusion u-net for qrs complexes and r-peak detection	2023	10.1109/tim.2023.3241997	SCOPUS	multichannel	yes
ecg wave segmentation algorithm for complete p-qrs-t detection	2023	10.1109/memea57477.2023.10171894	SCOPUS	inaccessible dataset	yes
ecg_segnet: an ecg delineation model based on the encoder-decoder structure	2022	10.1016/j.compbiomed.2022.105445	SCOPUS	INCLUDED	yes
ecg-based detection and prediction models of sudden cardiac death: current performances and new perspectives on signal processing techniques	2019	10.3991/ijoe.v15i15.11688	SCOPUS	off topic	no
ecg-based estimation of respiration-induced autonomic modulation of av nodal conduction during atrial fibrillation	2024	10.3389/fphys.2024.1281343	SCOPUS	off topic	no
ecg-based multiclass arrhythmia classification using beat-level fusion network	2023	10.1155/2023/1755121	SCOPUS	off topic	no

ecg-based score estimates the probability to detect fabry disease cardiac involvement	2021	10.1016/j.ijcard.2021.07.022	SCOPUS	off topic	no
ecg-based user authentication and identification method on vanets	2018	10.1145/3277103.3277138	SCOPUS	off topic	no
ecg-make: an ecg signal delineation approach based on medical attribute knowledge extraction	2023	10.1016/j.ins.2023.118978	SCOPUS	off topic	no
ecgdeli - an open source ecg delineation toolbox for matlab	2021	10.1016/j.softx.2020.100639	SCOPUS	off topic	no
effect of bone marrow-derived mesenchymal stem cells on ischaemic-reperfused hearts in adult rats with established chronic kidney disease	2019	10.15283/ijsc18114	SCOPUS	off topic	no
effects of xin-ji-er-kang on heart failure induced by myocardial infarction: role of inflammation, oxidative stress and endothelial dysfunction	2018	10.1016/j.phymed.2018.03.036	SCOPUS	off topic	no
efficient clustering-based electrocardiographic biometric identification	2023	10.1016/j.eswa.2023.119609	SCOPUS	off topic	no
electrical and morphological changes of cardiac pressure over-load left ventricular hypertrophy in rabbits	2017	10.16781/j.0258-879x.2017.01.0066	SCOPUS	off topic	no
electrocardiogram signal analysis for r-peak detection and denoising with hybrid linearization and principal component analysis	2017	10.3906/elk-1604-84	SCOPUS	missing technical details	yes
electrocardiogram signal classification in the diagnosis of heart disease based on rbf neural network	2022	10.1155/2022/9251225	SCOPUS	off topic	no
electrocardiographic abnormalities in patients with cardiomyopathies	2024	10.1007/s10741-023-10358-7	SCOPUS	off topic	no
electrocardiographic alternans: a new approach	2020	10.1007/978-3-030-31635-8_19	SCOPUS	off topic	no
electrocardiographic and autonomic nervous system changes after changes in the posture of children and adolescents with duchenne muscular dystrophy	2024	10.36660/abc.20230483	SCOPUS	off topic	no
electrocardiographic changes during initiation of lithium augmentation of antidepressant pharmacotherapy	2022	10.1097/jcp.0000000000001496	SCOPUS	off topic	no
electrocardiographic characteristics of bladder cancer patients receiving preoperative chemotherapy combined with immunotherapy	2024	10.1111/anec.13107	SCOPUS	off topic	no

electrocardiographic characteristics of breast cancer patients treated with chemotherapy	2020	10.1155/2020/6678503	SCOPUS	off topic	no
electrocardiographic characteristics of diffuse large b-cell lymphoma patients treated with anthracycline-based chemotherapy	2020	10.1016/ j.jelectrocard.2020.04.024	SCOPUS	off topic	no
electrocardiographic characteristics of trained and untrained standardbred racehorses	2022	10.1111/jvim.16427	SCOPUS	off topic	no
electrocardiographic modifications and cardiac involvement in covid-19 patients: results from an italian cohort	2021	10.2459/ jcm.0000000000001166	SCOPUS	off topic	no
electrocardiographic predictors of myocardial fibrosis and apical hypertrophic cardiomyopathy	2019	10.1111/anec.12612	SCOPUS	off topic	no
electrocardiographic strain pattern is associated with left ventricular concentric remodeling, scar, and mortality over 10 years: the multi-ethnic study of atherosclerosis	2017	10.1161/ jaha.117.006624	SCOPUS	off topic	no
electrocardiographic versus echocardiographic left ventricular hypertrophy in severe aortic stenosis	2021	10.3390/ jcm10112362	SCOPUS	off topic	no
electrocardiography in bitches with mammary tumors [eletrocardiografia computadorizada em cadelas com tumores mamários]	2021	10.22456/1679-9216.114172	SCOPUS	off topic	no
electrocardiography is unreliable to detect potential lethal hyperkalemia in patients with non-dialysis chronic kidney disease	2022	10.1007/s00246-022-02826-y	SCOPUS	off topic	no
electrocardiography is unreliable to detect potential lethal hyperkalemia in patients with non-dialysis chronic kidney disease	2022	10.1007/s00246-022-02826-y	SCOPUS	duplicate	no
electrogram analysis to detect cathodal and anodal capture in left ventricular cardiac resynchronization pacing leads	2022	10.1111/pace.14403	SCOPUS	off topic	no
electrographic lead i and v5 monitoring could have detected a missed left-side pneumothorax intraoperatively	2023	10.1111/anec.13017	SCOPUS	off topic	no
electronic health record with computerized decision support tools for the purposes of a pediatric cardiovascular heart disease screening program in crete	2018	10.1016/ j.cmpb.2018.03.009	SCOPUS	off topic	no

eletrocardiografia basal e após exercício em cães braquicefálicos	2023	10.22456/1679-9216.128359	SCOPUS	language not supported	no
emerging ecg methods for acute coronary syndrome detection: recommendations & future opportunities	2022	10.1016/j.jelectrocard.2022.08.003	SCOPUS	off topic	no
energy efficient vital signal analyzer	2020		SCOPUS	off topic	no
enhanced and optimal algorithm for qrs detection	2017	10.1016/j.irbm.2016.11.004	SCOPUS	INCLUDED	yes
enhancement of feature extraction in image quality	2019	10.1109/i-smac47947.2019.9032696	SCOPUS	off topic	no
entropy information of cardiorespiratory dynamics in neonates during sleep	2017	10.3390/e19050225	SCOPUS	off topic	no
envisaging ventricular arrhythmia from an ecg by using machine learning algorithm	2019	10.1109/icaccs.2019.8728525	SCOPUS	off topic	no
esophageal-ecg in the emergency department; [abc om esofagus-ekg]	2024		SCOPUS	off topic	no
estimation of pq distance dispersion for atrial fibrillation detection	2021	10.1016/j.cmpb.2021.106167	SCOPUS	off topic	no
evaluation of sensitivity and specificity of ecg left ventricular hypertrophy criteria in obese and hypertensive patients	2023	10.5603/ah.96273	SCOPUS	off topic	no
evaluation of the tp-e interval and tp-e/qtc ratio in patients with benign paroxysmal positional vertigo in the emergency department compared with the normal population	2020	10.1016/j.jelectrocard.2019.11.002	SCOPUS	off topic	no
existing methods to evaluate pacemaker device performance	2023	10.1007/978-981-19-5303-3_2	SCOPUS	off topic	no
extracting fetal ecg signals through a hybrid technique utilizing two wavelet-based denoising algorithms	2023	10.1109/access.2023.3308409	SCOPUS	fetal	no
extraction of p and t waves from electrocardiogram signals with modified hamilton algorithm	2019	10.1109/icsecc.2019.8907016	SCOPUS	off topic	no
false negative ecg device results may increase the risk of adverse events in clinical oncology trials	2022	10.1007/s43441-022-00405-0	SCOPUS	off topic	no

fast detection of p, q, s and t waves from normal ecg signals using local context windows	2019	10.1109/ rcar.2018.8621824	SCOPUS	off topic	no
fast qrs detection and ecg compression based on signal structural analysis	2019	10.1109/ jbhi.2018.2792404	SCOPUS	INCLUDED	yes
feasibility and diagnostic value of recording smartwatch electrocardiograms in neonates and children	2023	10.1016/ j.jpeds.2022.09.010	SCOPUS	off topic	no
feasibility of a novel ecg electrode placement method in newborn infants	2022	10.1159/000521530	SCOPUS	off topic	no
fetal cardiac time intervals in healthy pregnancies - an observational study by fetal ecg (monica healthcare system)	2018	10.1515/jpm-2017-0003	SCOPUS	fetal	no
fetal ecg extraction and qrs detection using advanced adaptive filtering-based signal decomposition and peak threshold technique from abdominal ecg signals	2023	10.1007/s00034-023-02386-3	SCOPUS	fetal	no
fetal electrocardiogram signal extraction based on fast independent component analysis and singular value decomposition	2022	10.3390/s22103705	SCOPUS	fetal	no
fetal heart rate extraction using nlms	2021	10.46300/91011.2021.15.8	SCOPUS	fetal	no
fetal qrs detection based on convolutional neural networks in noninvasive fetal electrocardiogram	2018	10.1109/ icfsp.2018.8552074	SCOPUS	fetal	no
foetal heart rate estimation by empirical mode decomposition and music spectrum	2018	10.1016/ j.bspc.2018.01.024	SCOPUS	fetal	no
fpga implementation of iot-based health monitoring system	2021	10.1109/ tssa52866.2021.9768261	SCOPUS	hardware	no
fragmented qrs as a predictor of subclinical cardiovascular disease in patients with chronic kidney disease	2020	10.1111/imj.14743	SCOPUS	off topic	no
fragmented qrs complex as a highly informative marker of ventricular depolarization disorders	2019		SCOPUS	off topic	no
fragmented qrs, as a surrogate marker for myocardial fibrosis, is associated with high blood lead levels	2020		SCOPUS	off topic	no
from pacemaker to wearable: techniques for ecg detection systems	2018	10.1007/s10916-017-0886-1	SCOPUS	does not introduce a new technique	no
frontal qrs-t angle predicts syntax score in patients with non-st elevation myocardial infarction	2020	10.1016/ j.jelectrocard.2020.06.008	SCOPUS	off topic	no

fusion algorithm for accurate delineation of qrs complex in ecg signal	2019	10.1007/s00034-018-0939-9	SCOPUS	not accessible	no
get rid of the beat in mobile eeg applications: a framework towards automated cardiogenic artifact detection and removal in single-channel eeg	2022	10.1016/j.bspc.2021.103220	SCOPUS	off topic	no
global longitudinal strain, myocardial storage and hypertrophy in fabry disease	2019	10.1136/heartjnl-2018-313699	SCOPUS	off topic	no
hamming filter design for ecg signal detection and processing using co-simulation	2017	10.1109/ccaa.2017.8229992	SCOPUS	denoising	no
health monitoring of human multiple physiological parameters based on wireless remote medical system	2020	10.1109/access.2020.2987058	SCOPUS	off topic	no
heart disease classification based on combination of pca /anfis model	2024	10.1007/s42600-024-00367-2	SCOPUS	off topic	no
heart rate monitoring and therapeutic devices: a wavelet transform based approach for the modeling and classification of congestive heart failure	2018	10.1016/j.isatra.2018.05.003	SCOPUS	off topic	no
heart rate variability and electrocardiographic parameters predictive of arrhythmias in dogs with stage iv chronic kidney disease undergoing intermittent haemodialysis	2020	10.3390/ani10101829	SCOPUS	animal	no
heartbeat detector from ecg and ppg signals based on wavelet transform and upper envelopes	2023	10.1007/s13246-023-01235-6	SCOPUS	INCLUDED	yes
heartquake: accurate low-cost non-invasive ecg monitoring using bed-mounted geophones	2020	10.1145/3411843	SCOPUS	off topic	no
hidden markov model-based heartbeat detector using electrocardiogram and arterial pressure signals	2021	10.1007/s13534-021-00192-x	SCOPUS	multichannel	no
high ecg diagnosis rate using novel machine learning techniques with distributed arithmetic (da) based gated recurrent units	2023	10.1016/j.micpro.2023.104796	SCOPUS	off topic	no
high quality ecg dataset based on mit-bih recordings for improved heartbeats classification	2023	10.1109/coins57856.2023.10189299	SCOPUS	not accessible	no
high-precision rhythm-based detection of atrial fibrillation and frequent premature contractions using extreme gradient boosting	2023	10.1109/bmeicon60347.2023.10322038	SCOPUS	off topic	yes

home management of the device detected atrial fibrillation during covid-19 pandemic: a case report	2021	10.3889/oamjms.2021.6687	SCOPUS	off topic	no
how to evaluate resting ecg and imaging in children practising sport: a critical review and proposal of an algorithm for ecg interpretation	2023	10.1093/eurjpc/zwac218	SCOPUS	off topic	no
human heart arrhythmia identification using ecg signals: an approach towards biomedical signal processing	2021	10.1007/978-981-15-4112-4_6	SCOPUS	off topic	no
hybrid feature extraction and stacking based ensemble classifier model for cardiomyopathy classification	2020		SCOPUS	off topic	no
identifying mitral valve prolapse at risk for arrhythmias and fibrosis from electrocardiograms using deep learning	2023	10.1016/j.jacadv.2023.100446	SCOPUS	off topic	no
impact of qrs misclassifications on heart-rate-variability parameters (results from the carla cohort study)	2024	10.1371/journal.pone.0304893	SCOPUS	off topic	no
implementation of the pulse rhythmic rate for the efficient diagnosing of the heartbeat	2019	10.1049/htl.2018.5043	SCOPUS	off topic	no
importance of the heart vector origin point definition for an ecg analysis: the atherosclerosis risk in communities (aric) study	2019	10.1016/j.compbiomed.2018.11.013	SCOPUS	off topic	no
improved two-layer bilstm electrocardiosignal segmentation method; [改进的两层 bilstm 的心电信号分割方法]	2023	10.12068/j.issn.1005-3026.2023.12.005	SCOPUS	language not supported	no
improving r peak detection in ecg signal using dynamic mode selected energy and adaptive window sizing algorithm with decision tree algorithm	2021	10.3390/s21196682	SCOPUS	inaccessible dataset	yes
independent detection of t-waves in single lead ecg signal using continuous wavelet transform	2023	10.1007/s13239-022-00643-1	SCOPUS	off topic	no
intelligent fatigue detection based on hierarchical multi-scale ecg representations and hrv measures	2024	10.1016/j.bspc.2024.106127	SCOPUS	off topic	no
inter-beat interval estimation from extremely noisy single lead electrocardiograms	2020	10.1016/j.ifacol.2020.12.744	SCOPUS	off topic	no
inter-patient cnn-lstm for qrs complex detection in noisy ecg signals	2019	10.1109/access.2019.2955738	SCOPUS	multichannel	no
interpretability analysis of machine learning algorithms in the detection of st-elevation myocardial infarction	2020	10.22489/cinc.2020.403	SCOPUS	off topic	no

interpretation of arrhythmogenic effects of covid-19 disease through ecg	2021	10.1080/13685538.2020.1769058	SCOPUS	off topic	no
interpretation of resnet50 model for mi related cardiac events using explainable grad-cam approach	2022	10.1515/cdbme-2022-1184	SCOPUS	off topic	no
investigating and importance of fetal monitoring methods and presenting a new method according to convolutional deep learning based on image processing to separate fetal heart signal from mother	2024	10.1007/978-3-031-48121-5_66	SCOPUS	fetal	no
investigation of telecardiology system to detect cardiac abnormalities	2019	10.1016/b978-0-12-816948-3.00004-0	SCOPUS	off topic	no
iot-enabled cloud-based real-time remote ecg monitoring system	2021	10.1080/03091902.2021.1921870	SCOPUS	off topic	no
iot-enabled heart monitoring device with signal de-noising and segmentation using discrete wavelet transform	2018	10.1109/icarcv.2018.8581315	SCOPUS	off topic	no
is screening for abnormal ecg patterns justified in long-term follow-up of childhood cancer survivors treated with anthracyclines?	2017	10.1002/pbc.26243	SCOPUS	off topic	no
italian return to play protocol after covid-19 in young competitive and professional athletes: diagnostic yield and cost-benefit analysis	2023	10.23736/s2724-5683.22.06191-9	SCOPUS	off topic	no
j wave syndromes in patients with spinal and bulbar muscular atrophy	2022	10.1007/s00415-022-10992-5	SCOPUS	off topic	no
left ventricular systolic dysfunction predicted by artificial intelligence using the electrocardiogram in chagas disease patients–the sami-trop cohort	2021	10.1371/journal.pntd.0009974	SCOPUS	off topic	no
life-threatening ventricular arrhythmia prediction in patients with dilated cardiomyopathy using explainable electrocardiogram-based deep neural networks	2022	10.1093/europace/euac054	SCOPUS	off topic	no
linear and nonlinear features for myocardial infarction detection using support vector machine on 12-lead ecg recordings	2021	10.1007/978-3-030-64610-3_85	SCOPUS	multichannel	no
linear phase sharp transition bpf to detect noninvasive maternal and fetal heart rate	2018	10.1155/2018/5485728	SCOPUS	fetal	no
machine learning approach to detect cardiac arrhythmias in ecg signals: a survey	2020	10.1016/j.irbm.2019.12.001	SCOPUS	off topic	no

machine learning approach to detect ecg abnormalities using cost-sensitive decision tree classifier	2023	10.4015/ s1016237223500217	SCOPUS	off topic	no
machine learning based decision support system for atrial fibrillation detection using electrocardiogram	2020	10.1109/ discover50404.2020.9278124	SCOPUS	off topic	no
machine learning for detecting atrial fibrillation from ecgs: systematic review and meta-analysis	2024	10.31083/ j.rcm2501008	SCOPUS	off topic	no
machine learning for real-time heart disease prediction	2021	10.1109/ jbhi.2021.3066347	SCOPUS	off topic	no
machine learning models of 6-lead ecgs for the interpretation of left ventricular hypertrophy (lvh)	2023	10.1016/ j.jelectrocard.2022.12.001	SCOPUS	multichannel	no
machine-learning algorithm to non-invasively detect diabetes and pre-diabetes from electrocardiogram	2022	10.1136/ bmjinnov-2021-000759	SCOPUS	off topic	no
magnetocardiography for the detection of myocardial ischemia	2023	10.3389/ fcvm.2023.1242215	SCOPUS	off topic	no
manifold analysis of the p-wave changes induced by pulmonary vein isolation during cryoballoon procedure	2023	10.1016/ j.compbiomed.2023.106655	SCOPUS	off topic	no
matlab based gui for ecg arrhythmia detection using pan-tompkin algorithm	2018	10.1109/ pdgc.2018.8745865	SCOPUS	does not introduce a new technique	no
measuring fine-grained heart-rate using a flexible wearable sensor in the presence of noise	2018	10.1109/ bsn.2018.8329683	SCOPUS	off topic	no
methodology for detection of paroxysmal atrial fibrillation based on p-wave, hrv and qr electrical alternans features	2020	10.11591/ ijece.v10i4.pp4023-4034	SCOPUS	off topic	no
methodology for the nocturnal cardiac arrhythmia ancillary study of the advent-hf trial in patients with heart failure with reduced ejection fraction and sleep-disordered breathing	2022	10.1016/ j.ijcha.2022.101057	SCOPUS	off topic	no
mobile health technologies in the diagnosis and management of atrial fibrillation	2022	10.1097/ hco.0000000000000930	SCOPUS	off topic	no
mobile/android application for qrs detection using zero cross method	2018	10.1088/1742-6596/978/1/012048	SCOPUS	missing accuracy	no

modeling of a system for fecg extraction from abdecg	2018	10.1007/978-3-319-76348-4_55	SCOPUS	off topic	no
moving dipole determination from 12-lead ecgs can improve detection of acute myocardial ischemia	2020	10.22489/cinc.2020.115	SCOPUS	multichannel	no
multi physiological signs model to enhance accuracy of ecg peaks detection	2017	10.1007/978-981-10-4220-1_12	SCOPUS	multichannel	no
multi-channel non-invasive fetal electrocardiography detection using wavelet decomposition	2017	10.1117/12.2286749	SCOPUS	fetal	no
multi-class arrhythmia classification and r-peak detection method of ecg signal based on one dimensional u net with skip-connection and data augmentation	2023	10.3233/faia231104	SCOPUS	off topic	yes
multi-dynamics analysis of qrs complex for atrial fibrillation diagnosis	2018	10.1109/codit.2018.8394935	SCOPUS	off topic	no
multichannel ecg recording from waist using textile sensors	2020	10.1186/s12938-020-00788-x	SCOPUS	multichannel	no
multilead qt interval analysis algorithm based on continuous wavelet transform	2017	10.1109/iceee2.2017.7935842	SCOPUS	multichannel	no
multiresolution wavelet transform based feature extraction and ecg classification to detect cardiac abnormalities	2017	10.1016/j.measurement.2017.05.022	SCOPUS	off topic	no
myocardial infarction detection using morphological features of ecg signal	2023	10.52711/0974-360x.2023.00482	SCOPUS	off topic	no
myocardial injury in patients with sickle cell anaemia and myocardial ischaemia in calabar, nigeria	2020	10.1080/20469047.2020.1789398	SCOPUS	off topic	no
neural networks for biomedical signals classification based on empirical mode decomposition and principal component analysis	2018	10.1007/978-3-319-72965-7_25	SCOPUS	off topic	no
new approach to eliminate noise attendants ecg signal corrupted	2021		SCOPUS	denoising	no
new features for the detection of fetal qrs complexes in non-invasive fetal electrocardiograms	2023	10.1109/embc40787.2023.10340399	SCOPUS	fetal	no
new method exploiting a hybrid techniques for fetal cardiac signal extraction	2019	10.4015/s1016237219500273	SCOPUS	fetal	no

new possibilities for st analysis – a post-hoc analysis on the dutch stan rct	2022	10.1016/ j.earlhumdev.2021.105537	SCOPUS	off topic	no
noise detection in electrocardiogram signals for intensive care unit patients	2019	10.1109/ access.2019.2926199	SCOPUS	off topic	no
non-invasive chronic asymptomatic chagas disease screening through high-resolution electrocardiography (ecg)	2017	10.12659/ mst.905455	SCOPUS	off topic	no
novel derma fusion technique for ecg heartbeat classification	2022	10.3390/ life12060842	SCOPUS	off topic	no
novel ecg features and machine learning to optimize culprit lesion detection in patients with suspected acute coronary syndrome	2021	10.1016/ j.jelectrocard.2021.07.012	SCOPUS	off topic	no
novel t-wave detection technique with minimal processing and rr-interval based enhanced efficiency	2019	10.1007/s13239-019-00415-4	SCOPUS	off topic	no
obstructive sleep apnea detection from ecg signal using neuro-fuzzy classifier	2018	10.1109/ icicict1.2017.8342686	SCOPUS	off topic	no
opening the black box: interpretability of machine learning algorithms in electrocardiography	2021	10.1098/ rsta.2020.0253	SCOPUS	off topic	no
optimized cardiovascular disease detection and features extraction algorithms from ecg data	2020	10.14569/ ijacsa.2020.0110826	SCOPUS	off topic	no
optimized systolic array filters with noise classification for extracting fecg	2023	10.59018/1123287	SCOPUS	off topic	no
optimizing ecg to detect echocardiographic left ventricular hypertrophy with computerbased ecg data and machine learning	2021	10.1371/ journal.pone.0260661	SCOPUS	off topic	no
overview of algorithms for electrocardiograms analysis	2017	10.23919/ fruct.2013.8124224	SCOPUS	off topic	no
parameter extraction of ecg using labview	2018	10.1109/ ssd.2018.8570391	SCOPUS	off topic	no
partition of medical signals with a two band adaptive filter bank [medikal isaretlerin iki bant uyarlamali süzgeç çatisi ile bölünmesi]	2018	10.1109/ siu.2018.8404160	SCOPUS	off topic	no
patient specific higher order tensor based approach for the detection and localization of myocardial infarction using 12-lead ecg	2023	10.1016/ j.bspc.2023.104701	SCOPUS	multichannel	no

pattern recognition of ecg signal to detect heart disorders	2023	10.1109/ vitecon58111.2023.10157257	SCOPUS	inaccessible dataset	yes
pediatric ecg-based deep learning to predict left ventricular dysfunction and remodeling	2024	10.1161/ circulationaha.123.067750	SCOPUS	off topic	no
performance evaluation of time-frequency distributions for ecg signal analysis	2018	10.1007/s10916-017-0871-8	SCOPUS	off topic	no
phase space reconstruction based cvd classifier using localized features	2019	10.1038/s41598-019-51061-8	SCOPUS	off topic	no
platform for analysis and labeling of medical time series	2020	10.3390/s20247302	SCOPUS	off topic	no
portable real time ecg monitor and disease diagnostics	2019	10.1109/ becithcon48839.2019.9063172	SCOPUS	off topic	no
portable system for real time detection of p, qrs and t waves from ecg signals	2019	10.1109/ i2ct45611.2019.9033666	SCOPUS	inaccessible dataset	no
precise detection and localization of r-peaks from ecg signals	2023	10.3934/ mbe.2023848	SCOPUS	INCLUDED	yes
precision and reproducibility of non-automatic measurement of the qrs complex in potential candidates for cardiac resynchronization therapy	2019	10.1016/ j.jelectrocard.2019.08.011	SCOPUS	off topic	no
precordial bipolar leads: a new method to study anterior acute myocardial infarction	2020	10.1016/ j.jelectrocard.2019.12.017	SCOPUS	off topic	no
precordial electrocardiographic recording and qt measurement from a novel wearable ring device	2024	10.1016/ j.cvdhj.2023.11.021	SCOPUS	off topic	no
predicting peri-operative troponin elevation by advanced electrocardiography	2021	10.1016/ j.jelectrocard.2021.06.008	SCOPUS	off topic	no
prediction of ecg cardiac abnormality signal using supervised prediction model	2023	10.1109/ icspc59664.2023.10420227	SCOPUS	off topic	no
prediction of ecg fiducial parameters from ppg signals for the analysis of cardiovascular diseases: a novel gaussian process regression-based approach	2024	10.1016/ j.bspc.2023.105838	SCOPUS	off topic	no

predictive value of frontal qrs-t angle after cardiac resynchronization therapy	2021	10.1016/ j.jelectrocard.2021.06.015	SCOPUS	off topic	no
premature ventricular contraction (pvc) detection system based on tunable q-factor wavelet transform	2022	10.31661/ jbpe.v0i0.1235	SCOPUS	off topic	no
premature ventricular contraction analysis for real-time patient monitoring	2019	10.1016/ j.bspc.2018.08.040	SCOPUS	off topic	no
presetting ecg electrodes for earlier heart rate detection in the delivery room	2018	10.1016/ j.resuscitation.2018.03.038	SCOPUS	off topic	no
prevalence and predictive value of electrocardiographic abnormalities in pulmonary hypertension: evidence from the pan-african pulmonary hypertension cohort (papuco) study	2017	10.5830/cvja-2017-020	SCOPUS	off topic	no
prevalence of left ventricular systolic dysfunction in myotonic dystrophy type 1: a systematic review	2020	10.1016/ j.cardfail.2019.07.548	SCOPUS	off topic	no
prognostic implications of supraventricular arrhythmias	2021	10.1016/ j.amjcard.2021.04.020	SCOPUS	off topic	no
prognostic value of ecg monitor findings in covid-19	2023	10.1136/ openhrt-2023-002404	SCOPUS	off topic	no
*	2021	10.1016/ j.jelectrocard.2020.11.011	SCOPUS	off topic	no
progression towards heart failure after myocardial infarction is accompanied by a change in the spatial qrs-t angle	2017	10.22489/ cinc.2017.292-342	SCOPUS	off topic	no
pulsation artifact removal from intra-operatively recorded local field potentials using sparse signal processing and data-specific dictionary	2023	10.1109/ embc40787.2023.10340160	SCOPUS	off topic	no
pulse arrival time and pulse interval as accurate markers to detect mechanical alternans	2019	10.1007/s10439-019-02221-4	SCOPUS	off topic	no
pulse transit time measurement using bioimpedance	2019	10.1007/978-981-13-2119-1_82	SCOPUS	off topic	no

qrs complex detection and measurement algorithms for multichannel ecgs in cardiac resynchronization therapy patients	2018	10.1109/ jtehm.2018.2844195	SCOPUS	multichannel	no
qrs complex detection in tele-health ecg recordings using modified unsw algorithm	2018	10.1109/ wispnet.2017.82999 51	SCOPUS	missing accuracy	no
qrs complex detection using novel deep learning neural networks	2020	10.1109/ access.2020.299747 3	SCOPUS	INCLUDED	yes
qrs detection and classification in holter ecg data in one inference step	2022	10.1038/s41598-022-16517-4	SCOPUS	inaccessible dataset	yes
qrs detection and measurement method of ecg paper based on convolutional neural networks	2018	10.1109/ embc.2018.8513132	SCOPUS	off topic	no
qrs detection in ecg signal with convolutional network	2019	10.1007/978-3-030-13469-3_93	SCOPUS	off topic	no
qrs detection method based on fully convolutional networks for capacitive electrocardiogram	2019	10.1016/ j.eswa.2019.05.033	SCOPUS	off topic	no
qrs detection of ecg signal using hybrid derivative and mamemi filter by effectively eliminating the baseline wander	2019	10.1007/s10470-018-1249-7	SCOPUS	denoising	no
qrs detection using novel digital differentiators	2017	10.1109/ icictm.2016.7890773	SCOPUS	does not introduce a new technique	no
qrs detector for maternal abdominal ecg	2017	10.1109/ iconsip.2016.785746 3	SCOPUS	fetal	no
qrs dispersion detected in arvc patients and healthy gene carriers using 252-leads body surface mapping: an explorative study of a potential diagnostic tool for arrhythmogenic right ventricular cardiomyopathy	2021	10.1111/pace.14295	SCOPUS	multichannel	no
qrs fragmentation index as a new discriminator for early diagnosis of heart diseases	2017	10.22489/ cinc.2017.267-258	SCOPUS	off topic	no
qrs peak detection for heart rate monitoring on android smartphone	2017	10.1088/1742-6596/909/1/012006	SCOPUS	hardware	no
qrs slopes for potassium and calcium monitoring in end-stage renal disease patients	2021	10.23919/ cinc53138.2021.966 2720	SCOPUS	off topic	no
qrs transitional zone rotations and the risk of atrial fibrillation: the suita study	2022	10.1007/s00380-022-02101-3	SCOPUS	off topic	no

qt interval measurement using derivative and thresholding process	2018	10.1063/1.5054437	SCOPUS	off topic	no
quantitative fragmented qrs has a good diagnostic value on myocardial fibrosis in hypertrophic obstructive cardiomyopathy based on clinical-pathological study	2020	10.1186/s12872-020-01590-2	SCOPUS	off topic	no
qvat: qrs complex detection based on variance analysis and adaptive threshold for electrocardiogram signal	2020	10.1109/isitia49792.2020.9163784	SCOPUS	INCLUDED	yes
r - peak detection using altered pan-tompkins algorithm	2020	10.1109/iccsp48568.2020.9182298	SCOPUS	does not introduce a new technique	no
r - peak detection using bayesian regularization neural network	2018	10.1109/icomicon.2017.8279025	SCOPUS	multichannel	yes
r-peak detection for ecg biomedical monitoring	2021	10.1109/icpics52425.2021.9524155	SCOPUS	missing accuracy	yes
r-peak detection from ecg signals using fractal based mathematical morphological operators	2021	10.1109/tencon54134.2021.9707247	SCOPUS	missing technical details	no
r-peak detection in electrocardiogram signals using continuous wavelet transform	2017		SCOPUS	not accessible	no
r-peak detection in holter ecg signals using non-negative matrix factorization	2018	10.22489/cinc.2018.123	SCOPUS	INCLUDED	yes
r-peak detection using efficient technique for tachycardia detection	2018	10.1109/mami.2017.8307877	SCOPUS	off topic	no
real time detection of r–peak in qrs complex of ecg using microcontroller	2018	10.11591/ijeecs.v11.i1.pp372-376	SCOPUS	hardware	no
real time ecg acquisition and fpga based qrs detection	2018	10.1109/iccubea.2018.8697710	SCOPUS	hardware	no
real-time ecg feature detection algorithm using moving statistic adaptive thresholding method for signal extremum sampling in microcontroller systems: a simulink approach	2022	10.1145/3535694.3535712	SCOPUS	hardware	no
real-time heart arrhythmia detection using apache spark structured streaming	2021	10.1155/2021/6624829	SCOPUS	off topic	no

recurrent nocturnal st-t deviation and nonsustained ventricular tachycardias recorded with a smartwatch: a case report	2022	10.1016/ j.jccase.2022.04.006	SCOPUS	off topic	no
reference intervals and method sensitivity for electrocardiology, hemodynamics, and body temperature parameters in healthy cynomolgus monkeys	2023	10.1016/ j.vascn.2022.107247	SCOPUS	off topic	no
remote ecg monitoring by ecg247 smart heart sensor	2022	10.1155/2022/6812889	SCOPUS	duplicate	no
remote ecg monitoring by ecg247 smart heart sensor	2022	10.1155/2022/6812889	SCOPUS	off topic	no
removal of ecg artifacts affects respiratory muscle fatigue detection (a simulation study)	2021	10.3390/s21165663	SCOPUS	denoising	no
research on qrs wave detection algorithm of ecg system	2023	10.1109/ itnec56291.2023.10082483	SCOPUS	missing accuracy	yes
research on the detection algorithm of electrocardiogram characteristic wave based on energy segmentation and stationary wavelet transform	2021	10.7507/1001-5515.202002038	SCOPUS	off topic	no
retraction: depression detection using comparative analysis of qrs detection algorithms and hrv of ecg signal implemented on matlab and verilog	2021	10.1088/1742-6596/1916/1/012018	SCOPUS	off topic	no
review on higher-order neural units to monitor cardiac arrhythmia patterns	2017	10.3233/978-1-61499-773-3-219	SCOPUS	review	no
revisiting derivative based methods on qrs detections from an ecg signal	2021	10.1109/ icaeca52838.2021.9675580	SCOPUS	off topic	no
robust electrocardiogram delineation model for automatic morphological abnormality interpretation	2023	10.1038/s41598-023-40965-1	SCOPUS	multichannel	yes
robust qrs detection using high-resolution wavelet packet decomposition and time-attention convolutional neural network	2020	10.1109/ access.2020.2967775	SCOPUS	off topic	no
rvm for recognition of qrs complexes in electrocardiogram	2018	10.1007/978-3-319-69096-4_17	SCOPUS	not accessible	no
saliency maps provide insights into artificial intelligence-based electrocardiography models for detecting hypertrophic cardiomyopathy	2023	10.1016/ j.jelectrocard.2023.07.002	SCOPUS	off topic	no

self-attention lstm-fcn model for arrhythmia classification and uncertainty assessment	2023	10.1016/ j.artmed.2023.10257 0	SCOPUS	off topic	no
self-attention mhdnet: a novel deep learning model for the detection of r-peaks in the electrocardiogram signals corrupted with magnetohydrodynamic effect	2023	10.3390/ bioengineering10050 542	SCOPUS	off topic	no
semi-supervised learning for ecg classification	2021	10.23919/ cinc53138.2021.966 2693	SCOPUS	multichannel	no
serial ecg analysis: absolute rather than signed changes in the spatial qrs-t angle should be used to detect emerging cardiac pathology	2018	10.22489/ cinc.2018.099	SCOPUS	off topic	no
serial electrocardiography to detect newly emerging or aggravating cardiac pathology: a deep-learning approach	2019	10.1186/s12938- 019-0630-9	SCOPUS	off topic	no
shannon's energy based algorithm in ecg signal processing	2017	10.1155/2017/80813 61	SCOPUS	multichannel	no
signal filtering and peak analysis of ballistocardiography for heartbeat detection	2024	10.1007/978-3-031- 59216-4_33	SCOPUS	off topic	no
signal processing framework for the detection of ventricular ectopic beat episodes	2023	10.4103/ jmss.jmss_12_22	SCOPUS	off topic	no
simclr-unet: an ecg feature wave segmentation algorithm based on a self-supervised learning strategy	2022	10.1145/3584376.35 84615	SCOPUS	off topic	no
simplified process of obstructive sleep apnea detection using ecg signal based analysis with data flow programming	2018	10.1007/978-3-319- 63645-0_18	SCOPUS	off topic	no
single-lead noninvasive fetal ecg extraction by means of combining clustering and principal components analysis	2020	10.1007/s11517- 019-02087-7	SCOPUS	fetal	no
sleep order detection model using support vector machines and features extracted from brain ecg signals	2018	10.1109/ icici.2017.8365290	SCOPUS	off topic	no
sleep–wake stage detection with single channel ecg and hybrid machine learning model in patients with obstructive sleep apnea	2021	10.1007/s13246- 020-00953-5	SCOPUS	off topic	no
smart ecg holter monitoring system using smartphone	2019	10.1109/ iotais.2018.8600891	SCOPUS	off topic	no

smartphone-based ecg signals encryption for transmission and analyzing via iomts	2021	10.1080/09720529.2021.1958996	SCOPUS	off topic	no
smartwatch: looking beyond what you see	2023	10.1016/j.jelectrocard.2023.07.010	SCOPUS	off topic	no
solithromycin, a novel macrolide, does not prolong cardiac repolarization: a randomized, three-way crossover study in healthy subjects	2017	10.1093/jac/dkw428	SCOPUS	off topic	no
st waveform analysis for monitoring hypoxic distress in fetal sheep after prolonged umbilical cord occlusion	2018	10.1371/journal.pone.0195978	SCOPUS	fetal	no
stages-based ecg signal analysis from traditional signal processing to machine learning approaches: a survey	2020	10.1109/access.2020.3026968	SCOPUS	off topic	no
statistical and entropy-based features can efficiently detect the short-term effect of caffeinated coffee on the cardiac physiology	2020	10.1016/j.mehy.2020.110323	SCOPUS	off topic	no
study on abnormal detection of ecg signal base on dcnn	2019	10.1088/1742-6596/1345/4/042006	SCOPUS	off topic	no
successful catheter ablation of the “r on t” ventricular fibrillation	2021	10.3390/ijerph18189587	SCOPUS	off topic	no
temporal performance of laplacian eigenmaps and 3d conduction velocity in detecting ischemic stress	2018	10.1016/j.jelectrocard.2018.08.017	SCOPUS	off topic	no
tensor-domain machine learning based cardiac diseases detection using 12-lead ecg	2023	10.1109/ncc56989.2023.10068027	SCOPUS	multichannel	no
the ability of the electrocardiogram in left bundle branch block to detect myocardial scar determined by cardiovascular magnetic resonance	2018	10.1016/j.jelectrocard.2018.05.019	SCOPUS	off topic	no
the association between ecg criteria and echo criteria for left ventricular hypertrophy in a general chinese population	2021	10.1111/anec.12880	SCOPUS	off topic	no
the ecg in sarcoidosis – a marker of cardiac involvement? current evidence and clinical implications	2021	10.1016/j.jjcc.2020.07.006	SCOPUS	off topic	no
the effectiveness of a deep learning model to detect left ventricular systolic dysfunction from electrocardiograms	2021	10.1536/ihj.21-407	SCOPUS	off topic	no

the electro-anatomical pathway for normal and abnormal ecgs in covid patients	2020	10.22489/ cinc.2020.096	SCOPUS	off topic	no
the feature extraction of ecg signal in myocardial infarction patients	2018	10.1007/978-3-319-70016-8_14	SCOPUS	off topic	no
the groningen electrocardiographic criteria for left ventricular hypertrophy: a sex-specific analysis	2021	10.1038/s41598-021-83137-9	SCOPUS	off topic	no
the in-ear region as a novel anatomical site for ecg signal detection: validation study on healthy volunteers	2021	10.1007/s10840-020-00709-x	SCOPUS	off topic	no
the limb leads ecg signal analysis in inferior myocardial infarction patients by rule base	2018	10.1109/isai-nlp.2018.8692998	SCOPUS	off topic	no
the relation of blood lead and qrs-t angle in american adults	2019	10.1080/19338244.2018.1488674	SCOPUS	off topic	no
the standardized 12-lead fetal electrocardiogram of the healthy fetus in mid-pregnancy: a cross-sectional study	2020	10.1371/ journal.pone.0232606	SCOPUS	fetal	no
the use of heart rate variability analysis to detect arrhythmias in horses undergoing a standard treadmill exercise test	2019	10.1111/jvim.15358	SCOPUS	off topic	no
the utility of a lewis lead for distinguishing atrioventricular reentrant tachycardia from typical atrioventricular nodal reentrant tachycardia	2022	10.2169/ internalmedicine.8470-21	SCOPUS	off topic	no
the utility of electrocardiography and echocardiography in copper deficiency-induced cardiac damage in goats	2021	10.1007/s11356-020-11014-5	SCOPUS	off topic	no
the value and applicability of the electrocardiography in revealing the cardiac involvement of covid-19 patients	2021	10.5455/ aim.2021.29.253-259	SCOPUS	off topic	no
the δwaveecg: the differences to the normal 12-lead ecg amplitudes	2023	10.1016/ j.jelectrocard.2022.10.014	SCOPUS	multichannel	no
towards end-to-end ecg classification with raw signal extraction and deep neural networks	2019	10.1109/ jbhi.2018.2871510	SCOPUS	off topic	no
transcription factor meis1 act as a new regulator of ischemic arrhythmias in mice	2021	10.1016/ j.jare.2021.11.004	SCOPUS	off topic	no
true complete left bundle branch block reveals dyssynchrony evaluated by semiconductor single-photon emission computed tomography	2019	10.1002/joa3.12148	SCOPUS	off topic	no
twenty-four hour continuous transvenous temporary right ventricular pacing in healthy horses	2024	10.1111/jvim.17027	SCOPUS	off topic	no

ultra-low power qrs detection using adaptive thresholding based on forward search interval technique	2017	10.1109/ edssc.2017.8126486	SCOPUS	not accessible	no
uncertainty quantification in densenet model using myocardial infarction ecg signals	2023	10.1016/ j.cmpb.2022.107308	SCOPUS	off topic	no
uncovering electromechanical uncoupling in subclinical pathogenic mutation carriers and arrhythmogenic cardiomyopathy patients	2021	10.23919/ cinc53138.2021.9662949	SCOPUS	off topic	no
update on the ecg component of the cipa initiative	2018	10.1016/ j.jelectrocard.2018.08.003	SCOPUS	off topic	no
use of a transformed ecg signal to detect respiratory effort during apnea	2019	10.5664/jcsm.7880	SCOPUS	off topic	no
usefulness of routine transtelephonic monitoring for supraventricular tachycardia in infants	2018	10.1016/ j.jpeds.2017.10.014	SCOPUS	off topic	no
validation of an automatic diagnosis of strict left bundle branch block criteria using 12-lead electrocardiograms	2017	10.1111/anec.12398	SCOPUS	multichannel	no
value of 12-lead electrocardiogram to predict myocardial scar on fdg pet in heart failure patients	2021	10.1007/s12350-019-01841-6	SCOPUS	multichannel	no
value of a novel 16-lead high-definition ecg machine to detect conduction abnormalities in structural heart disease	2018	10.1111/pace.13338	SCOPUS	multichannel	no
values of electrocardiogram qrs duration and serum apob/apoa- ⅰ in severity assessment of coronary artery lesions in patients with coronary heart disease	2020	10.14188/j.1671-8852.2019.0052	SCOPUS	off topic	no
variational mode decomposition-based simultaneous r peak detection and noise suppression for automatic ecg analysis	2023	10.1109/ jsen.2023.3257332	SCOPUS	INCLUDED	yes
vectorcardiographic qrs area as a predictor of response to cardiac resynchronization therapy	2022	10.11909/ j.issn.1671-5411.2022.01.003	SCOPUS	off topic	no
ventricular dyssynchrony assessment using ultra-high frequency ecg technique	2017	10.1007/s10840-017-0268-0	SCOPUS	off topic	no
verb: vfcdm-based electrocardiogram reconstruction and beat detection algorithm	2019	10.1109/ access.2019.2894092	SCOPUS	INCLUDED	yes

verification and comparison of mit-bih arrhythmia database based on number of beats	2021	10.11591/ ijece.v11i6.pp4950-4961	SCOPUS	off topic	no
visual saliency detection approach for long-term ecg analysis	2022	10.1016/ j.cmpb.2021.106518	SCOPUS	off topic	no
vlsi architecture for energy-efficient and accurate pre-processing pan-tompkins design	2023	10.1109/ tcsii.2023.3241124	SCOPUS	hardware	no
watch out for st-elevation myocardial infarction: a case report of st-elevation in single-lead electrocardiogram tracing of a smartwatch	2020	10.1093/ehjcr/ ytaa353	SCOPUS	off topic	no
wavelet and kernel dimensional reduction on arrhythmia classification of ecg signals	2020	10.4108/eai.13-7-2018.163095	SCOPUS	off topic	no
wavelet transform based detection of the first-degree atrioventricular block	2021	10.23919/ cinc53138.2021.9662877	SCOPUS	off topic	no
wearable ecg for real time complex p-qrs-t detection and classification of various arrhythmias	2019	10.1109/ comsnets.2019.8711218	SCOPUS	does not introduce a new technique	yes

DOCUMENT TITLE	YEAR	DOI	DATA BASE	INCLUSION/EXCLUSION	ASSESSED FULL TEXT
17.8 a 2.6µw monolithic cmos photoplethysmographic sensor operating with 2µw led power	2019	10.1109/ isscc.2019.8662404	IEEE	duplicate	no
28.2 a 400-to-1000nm 24µ w monolithic ppg sensor with 0.3a/w spectral responsivity for miniature wearables	2021	10.1109/ isscc42613.2021.93 66035	IEEE	duplicate	no
28.3 a 28µw 134db dr 2nd-order noise-shaping slope light-to-digital converter for chest ppg monitoring	2021	10.1109/ isscc42613.2021.93 65757	IEEE	duplicate	no
a 0.5v ppg-based heart rate and variability detection system	2018	10.1109/ biocas.2018.858475 6	IEEE	duplicate	no
a 1.8v 16µa 136.5db dr ppg/nirs recording ic using noise shaping triple slope light to digital converter	2023	10.1109/ cicc57935.2023.101 21288	IEEE	duplicate	no
a 171µw ppg-based vitals monitoring soc for asthmatic patients	2022	10.1109/ biocas54905.2022.9 948619	IEEE	duplicate	no
a 172 μw compressively sampled photoplethysmographic (ppg) readout asic with heart rate estimation directly from compressively sampled data	2017	10.1109/ tbcas.2017.2661701	IEEE	duplicate	no
a 1mw vitals monitoring system for asthmatic patients based on photoplethysmography	2019	10.1109/ biocas.2019.891872 4	IEEE	duplicate	no
a 2.3–5.7 µw tri-modal self-adaptive photoplethysmography sensor interface ic for heart rate, spo2, and pulse transit time co-monitoring	2024	10.1109/ tbcas.2024.3360140	IEEE	duplicate	no
a 206 µw vital signs monitoring system on chip for measuring five vitals	2024	10.1109/ tvlsi.2024.3415469	IEEE	duplicate	no
a 385µw photoplethysmography-based vitals monitoring soc with 110db current-to-digital converter	2022	10.1109/ iscas48785.2022.99 37795	IEEE	duplicate	no
a 3d-printed wearable ring sensor for long-term accurate monitoring of human cardiovascular condition	2022	10.1109/ sensors52175.2022. 9967182	IEEE	off topic	no
a 43.4µw photoplethysmogram-based heart-rate sensor using heart-beat-locked loop	2018	10.1109/ isscc.2018.8310390	IEEE	duplicate	no

a camera-based pulse transit time estimation approach towards non-intrusive blood pressure monitoring	2019	10.1109/ ichi.2019.8904498	IEEE	duplicate	no
a comparative evaluation of a wearable mems tactile sensors array and a photoplethysmography sensor for atrial fibrillation detection under sitting condition	2021	10.1109/ transducers50396.2021.9495407	IEEE	duplicate	no
a comparison of wearable tonometry, photoplethysmography, and electrocardiography for cuffless measurement of blood pressure in an ambulatory setting	2022	10.1109/ jbhi.2022.3153259	IEEE	duplicate	no
a comprehensive review of remote photoplethysmography techniques for accurate and robust heart rate monitoring	2024	10.1109/ nigercon62786.2024.10927392	IEEE	review	no
a dual-channel ppg readout system with motion-tolerant adaptability for oled-opd sensors	2022	10.1109/ tbcas.2021.3138996	IEEE	duplicate	no
a dynamic bayesian multichannel fusion scheme for heart rate monitoring with ballistocardiograph signals in free-living environments	2024	10.1109/ jsas.2024.3485544	IEEE	multichannel	no
a fast fpga hardware accelerator for remote heart rate detection based on rgb vision	2024	10.1109/ tbcas.2024.3354505	IEEE	duplicate	no
a flexible and miniaturized chest patch for real-time ppg/ecg/bio-z monitoring	2022	10.1109/ embc48229.2022.9872005	IEEE	duplicate	no
a frame work to estimate heart rate and arterial oxygen saturation (spo2)	2017	10.1109/ iccsp.2017.8286669	IEEE	duplicate	no
a high accuracy & ultra-low power ppg-derived hr estimation ai processor for wearable devices	2023	10.1109/ icet58434.2023.10212060	IEEE	duplicate	no
a low-power heart rate sensor with adaptive heartbeat locked loop	2021	10.1109/ iscas51556.2021.9401726	IEEE	duplicate	no
a low-power photoplethysmogram-based heart rate sensor using heartbeat locked loop	2018	10.1109/ tbcas.2018.2876671	IEEE	duplicate	no
a low-power ppg processor for real-time biometric identification and heart rate estimation	2023	10.1109/ tcsii.2023.3291891	IEEE	duplicate	no
a low-power predictive sampling ppg sensor	2024	10.1109/ iscas58744.2024.10557982	IEEE	duplicate	no

a low-power sleep apnea monitoring ic with a duty-recovered body channel communication receiver	2022	10.1109/a-sscc56115.2022.9980624	IEEE	off topic	no
a lstm-based realtime signal quality assessment for photoplethysmogram and remote photoplethysmogram	2021	10.1109/cvprw53098.2021.00424	IEEE	duplicate	no
a machine learning approach for heart rate estimation from ppg signal using random forest regression algorithm	2019	10.1109/ecace.2019.8679356	IEEE	duplicate	no
a machine learning based human activity recognition during physical exercise using wavelet packet transform of ppg and inertial sensors data	2019	10.1109/eict48899.2019.9068768	IEEE	off topic	no
a machine learning method to improve non-contact heart rate monitoring using an rgb camera	2018	10.1109/access.2018.2872756	IEEE	duplicate	no
a machine learning-empowered system for long-term motion-tolerant wearable monitoring of blood pressure and heart rate with ear-ecg/ppg	2017	10.1109/access.2017.2707472	IEEE	duplicate	no
a motion and illumination resistant non-contact method using undercomplete independent component analysis and levenberg-marquardt algorithm	2022	10.1109/jbhi.2022.3144677	IEEE	off topic	no
a multi-wavelength photoplethysmography-based patch physiological monitoring system: design and functional validation	2024	10.1109/wrrc62201.2024.10696252	IEEE	inaccessible dataset	no
a new intelligent approach for automatic stress level assessment based on multiple physiological parameters monitoring	2024	10.1109/tim.2023.3342218	IEEE	duplicate	no
a non-contact heart rate estimation framework based on photoplethysmography amplitude variation elimination and data fusion	2021	10.1109/icbme54433.2021.9750345	IEEE	duplicate	no
a novel adaptive spectrum noise cancellation approach for enhancing heartbeat rate monitoring in a wearable device	2018	10.1109/access.2018.2805223	IEEE	duplicate	no
a novel chest-based ppg measurement system	2024	10.1109/jtehm.2024.3471468	IEEE	hardware	no
a novel motion artifact removal method via joint basis pursuit linear program to accurately monitor heart rate	2019	10.1109/jsen.2019.2927994	IEEE	duplicate	no

a novel strategy for signal reconstruction of noisy segments in photoplethysmographic recordings	2022	10.1109/ ehb55594.2022.999 1390	IEEE	duplicate	no
a novel technique to compress photoplethysmogram signal: improvised with particle swarm optimization and rivest-shamir-adleman algorithm	2022	10.1109/ calcon56258.2022.1 0060588	IEEE	duplicate	no
a novel video-based real-time non-contact heart rate measurement algorithm: based on third-order adaptive fastica	2024	10.1109/icnc- fskd64080.2024.107 02318	IEEE	off topic	no
a practical approach to health status monitoring based on heart rate and respiratory rate assessment	2022	10.1109/ memea54994.2022. 9856576	IEEE	duplicate	no
a preliminary investigation of the effect of contact pressure on the accuracy of heart rate monitoring by wearable ppg wrist band	2019	10.1109/ metroi4.2019.87928 34	IEEE	duplicate	no
a random tree based algorithm for blood pressure estimation	2020	10.1109/ imbioc47321.2020.9 385038	IEEE	duplicate	no
a real-time non-contact heart rate measurement based on imaging photoplethysmography (ippg)-power spectral density (psd)	2021	10.1109/ isiea51897.2021.950 9987	IEEE	off topic	no
a real-time software to the acquisition of heart rate and photoplethysmography signal using two region of interest simultaneously via webcam	2017	10.1109/ urtc.2017.8284207	IEEE	duplicate	no
a review of ppg/nirs acquisition asic and system	2021	10.1109/ asicon52560.2021.9 620430	IEEE	duplicate	no
a robust fusion method for motion artifacts reduction in photoplethysmography signal	2020	10.1109/ tim.2020.3006636	IEEE	duplicate	no
a robust motion artifact detection algorithm for accurate detection of heart rates from photoplethysmographic signals using time–frequency spectral features	2017	10.1109/ jbhi.2016.2612059	IEEE	duplicate	no
a smart mandibular advancement device for intraoral cardiorespiratory monitoring	2020	10.1109/ embc44109.2020.91 76520	IEEE	duplicate	no

a sock-embedded wireless sensing system employing paper-substrate-based mems tactile sensors array and low-power asic for accurate and comfortable afib monitoring	2023	10.1109/jsen.2023.3329100	IEEE	duplicate	no
a sparse sampling sensor front-end ic for low power continuous spo$_{2}$ & hr monitoring	2022	10.1109/tbcas.2022.3223971	IEEE	duplicate	no
a subcutaneously injectable implant for multimodal physiological monitoring in animals	2024	10.1109/jsen.2024.3366195	IEEE	duplicate	no
a survey on fatigue and drowsiness detection techniques in driving	2021	10.1109/iccis51004.2021.9397224	IEEE	duplicate	no
a systematic analysis: remote monitoring of heart rate, blood pressure, respiratory rate, and oxygen saturation based on images and without physical touch	2022	10.1109/icac3n56670.2022.10074569	IEEE	off topic	no
a trellis-based peak frequency tracking technique for photoplethysmographic heart rate sensing during exercise	2020	10.1109/ismict48699.2020.9152718	IEEE	duplicate	no
a two-stream deep-learning network for heart rate estimation from facial image sequence	2024	10.1109/jsen.2024.3483629	IEEE	off topic	no
a vital-signs monitoring wristband with real-time in-sensor data analysis using very low-hardware resources	2024	10.1109/jsen.2024.3388950	IEEE	hardware	no
a wearable nose clip sensor system for monitoring vital body signs of apnea patients	2024	10.1109/jsen.2024.3457530	IEEE	off topic	no
a wearable wireless medical sensor network system towards internet-of-patients	2018	10.1109/icsens.2018.8589642	IEEE	duplicate	no
a wireless low-power single-unit wearable system for continuous early warning score calculation	2023	10.1109/jsen.2023.3267146	IEEE	duplicate	no
a wireless wearable sensor patch for the real-time estimation of continuous beat-to-beat blood pressure	2019	10.1109/embc.2019.8857446	IEEE	duplicate	no
accurate detection of heart rate and blood oxygen saturation in reflective photoplethysmography	2020	10.1109/isspit51521.2020.9408845	IEEE	duplicate	no
accurate heart rate monitoring during physical exercises using ppg	2017	10.1109/tbme.2017.2676243	IEEE	duplicate	no
accurate heart rate monitoring method during physical exercise from photoplethysmography signal	2019	10.1109/jsen.2018.2886001	IEEE	duplicate	no

accurate heart-rate estimation from face videos using quality-based fusion	2017	10.1109/icip.2017.8297060	IEEE	duplicate	no
acquiring photoplethysmography (ppg) signal without led	2023	10.1109/i2mtc53148.2023.10175960	IEEE	duplicate	no
acquisition and processing of ecg and ppg signals using face-worn sensors for extracting the cardio-respiratory parameters	2024	10.23919/splitech61897.2024.10612365	IEEE	multichannel	no
adaptive algorithm for motion artifacts removal in wearable biomedical sensors during physical exercise	2023	10.1109/jsen.2023.3256959	IEEE	duplicate	no
afodss: heart rate estimation method from photoplethysmographic signals with motion artifacts using fourier-sparse dual optimization	2019	10.1109/jsen.2019.2928643	IEEE	duplicate	no
ai-based models to predict the heart rate using ppg and accelerometer signals during physical exercise	2023	10.1109/bibm58861.2023.10385871	IEEE	inaccessible dataset	no
ai-driven rppg heart rate detection for in-vehicle monitoring	2024	10.1109/sensors60989.2024.10785230	IEEE	off topic	no
ambient light-driven wireless wearable finger patch for monitoring vital signs from ppg signal	2024	10.1109/jsen.2023.3335309	IEEE	duplicate	no
an adaptive filter based motion artifact cancellation technique using multi-wavelength ppg for accurate hr estimation	2023	10.1109/tbcas.2023.3315297	IEEE	duplicate	no
an automated device for recording peripheral arterial waveform	2019	10.22489/cinc.2019.260	IEEE	off topic	no
an effective photoplethysmography heart rate estimation framework integrating two-level denoising method and heart rate tracking algorithm guided by finite state machine	2022	10.1109/jbhi.2022.3165071	IEEE	duplicate	no
an efficient approach to estimating heart rate from facial videos with accurate region of interest	2024	10.1109/inocon60754.2024.10511840	IEEE	off topic	no
an extended system for external sensors data acquisition and validation during conducting polysomnography	2023	10.1109/ssp53291.2023.10208050	IEEE	duplicate	no
an integrated wearable wireless vital signs biosensor for continuous inpatient monitoring	2020	10.1109/jsen.2019.2942099	IEEE	off topic	no

an ultralow-power ppg sensor with adaptive predictive sampling	2024	10.1109/ jsen.2024.3378115	IEEE	duplicate	no
android based heart rate monitoring and automatic notification system	2017	10.1109/r10-htc.2017.8288993	IEEE	off topic	no
application of chrominance based rppg in estimation of heart rate from video signal	2021	10.1109/ iccit54785.2021.968 9811	IEEE	off topic	no
applying a deep learning network in continuous physiological parameter estimation based on photoplethysmography sensor signals	2022	10.1109/ jsen.2021.3126744	IEEE	duplicate	no
assessment of samsung galaxy watch4 ppg-based heart rate during light-to-vigorous physical activities	2024	10.1109/ lsens.2024.3408089	IEEE	duplicate	no
autonomous heart rate tracking methodology using kalman filter and the em algorithm	2019	10.23919/ fusion43075.2019.90 11407	IEEE	duplicate	no
binary cornet: accelerator for hr estimation from wrist-ppg	2020	10.1109/ tbcas.2020.3001675	IEEE	duplicate	no
blood pressure estimation based on pulse arrival time and heart rate : a correlation analysis for critically ill patients	2019	10.1109/ embc.2019.8856583	IEEE	off topic	no
blood pressure estimation using photoplethysmogram signal and its morphological features	2020	10.1109/ jsen.2019.2961411	IEEE	duplicate	no
blood pressure monitoring and hypertension prediction using dynamic opposite learning based prairie dog optimization algorithm	2024	10.1109/ icmnwc63764.2024. 10872294	IEEE	animal	no
boosting algorithms based cuff-less blood pressure estimation from clinically relevant ecg and ppg morphological features	2023	10.1109/ embc40787.2023.10 340405	IEEE	duplicate	no
camera-based cardiorespiratory monitoring of preterm infants in nicu	2024	10.1109/ tim.2024.3395314	IEEE	off topic	no
camera-based cardiovascular screening based on heart rate and its variability in pre- and post-exercise conditions	2023	10.1109/ embc40787.2023.10 340871	IEEE	duplicate	no
camera-based remote photoplethysmography for physiological monitoring in neonatal intensive care	2021	10.1109/ icsipa52582.2021.95 76779	IEEE	off topic	no
can accelerometry data improve estimates of heart rate variability from wrist pulse ppg sensors?	2017	10.1109/ embc.2017.8037141	IEEE	off topic	no

cardiac pulse monitoring through multi-scale spectrum	2024	10.1109/ amathe61652.2024. 10582227	IEEE	duplicate	no
cbppggan: a generic enhancement framework for unpaired pulse waveforms in camera-based photoplethysmography	2024	10.1109/ jbhi.2023.3314282	IEEE	off topic	no
characterization study of neck photoplethysmography	2018	10.1109/ embc.2018.8513247	IEEE	duplicate	no
chest wearable apparatus for cuffless continuous blood pressure measurements based on ppg and pcg signals	2020	10.1109/ access.2020.298130 0	IEEE	duplicate	no
chest-based real-time pulse and respiration monitoring based on bio-impedance	2020	10.1109/ embc44109.2020.91 76348	IEEE	duplicate	no
classifying nocturnal blood pressure patterns using photoplethysmogram features	2022	10.1109/ embc48229.2022.98 71099	IEEE	duplicate	no
cmrppgformer: 3-d spatio-temporal convolutional modulation transformer network for remote heart rate estimation	2024	10.1109/ jsen.2024.3407816	IEEE	off topic	no
comparative assessment of smartwatch photoplethysmography accuracy	2024	10.1109/ lsens.2023.3342292	IEEE	duplicate	no
comparing consumer and research-grade wristbands for inter-beat intervals monitoring	2024	10.1109/ metroxraine62247.2 024.10797108	IEEE	off topic	no
comparing remote heart rate accuracy using different wavelengths	2021	10.1109/ scored53546.2021.9 652720	IEEE	duplicate	no
comparison and evaluation of ippg methods for hr estimation under different face regions	2022	10.1109/ ssd54932.2022.9955 726	IEEE	off topic	no
comparison between heart rate variability and pulse rate variability for bradycardia and tachycardia subjects	2018	10.1109/ iccais.2018.8570697	IEEE	duplicate	no
comparison of region of interest segmentation methods for video-based heart rate measurements	2018	10.1109/ bibe.2018.00034	IEEE	duplicate	no
comparison of wristband type devices to measure heart rate variability for mental stress assessment	2021	10.1109/ ictc52510.2021.9620 772	IEEE	duplicate	no

comparison study of noncontact vital signs detection using a doppler stepped-frequency continuous-wave radar and camera-based imaging photoplethysmography	2017	10.1109/ tmtt.2017.2658567	IEEE	off topic	no
competitive state anxiety inventory assessment using remote photoplethysmography and deep learning	2023	10.1109/ sse60056.2023.00044	IEEE	off topic	no
complementary photoplethysmogram synthesis from electrocardiogram using generative adversarial network	2021	10.1109/ access.2021.3078534	IEEE	duplicate	no
complete cardiorespiratory monitoring via wearable ultra low power ultrasound	2023	10.1109/ ius51837.2023.10307398	IEEE	duplicate	no
compressed estimation of heart and respiratory rates from a photoplethysmogram	2017	10.1109/ biocas.2017.8325158	IEEE	duplicate	no
contact-free monitoring of physiological parameters in people with profound intellectual and multiple disabilities	2019	10.1109/ iccvw.2019.00206	IEEE	duplicate	no
contactless hr measurement from facial videos using alternative color spaces with ceemdan	2023	10.1109/ icsc60394.2023.10441625	IEEE	off topic	no
contactless patient care using hospital iot: cctv-camera-based physiological monitoring in icu	2024	10.1109/ jiot.2023.3308477	IEEE	off topic	no
contributions to the acquisition of heart rate and photoplethysmography signal using a real-time software	2018	10.1109/ ichi.2018.00046	IEEE	off topic	no
cornet: deep learning framework for ppg-based heart rate estimation and biometric identification in ambulant environment	2019	10.1109/ tbcas.2019.2892297	IEEE	duplicate	no
corrections to “advancing healthcare monitoring: integrating machine learning with innovative wearable and wireless systems for comprehensive patient care”	2024	10.1109/ jsen.2024.3476941	IEEE	off topic	no
correlation analysis of heart rate variability between ppg and ecg for wearable devices in different postures	2017	10.1109/ smc.2017.8123077	IEEE	duplicate	no
coverage of ppg-based wearable devices in office tasks	2023	10.22489/ cinc.2023.206	IEEE	duplicate	no

data-augmentation for deep learning based remote photoplethysmography methods	2021	10.1109/ ehb52898.2021.965 7650	IEEE	duplicate	no
deep adaptative spectral zoom for improved remote heart rate estimation	2024	10.1109/ fg59268.2024.10581 863	IEEE	off topic	no
deep learning associated with linear regression for heart beat rate (hbr) and respiration rate(rr) measurement	2024	10.1109/ iciteics61368.2024.1 0624896	IEEE	multichannel	no
deep learning based pulse rate prediction in noisy ppg signals with practical application to wearables	2022	10.1109/ siu55565.2022.9864 877	IEEE	duplicate	no
deep learning for heart rate estimation from reflectance photoplethysmography with acceleration power spectrum and acceleration intensity	2020	10.1109/ access.2020.298195 6	IEEE	duplicate	no
deep learning fused wearable pressure and ppg data for accurate heart rate monitoring	2021	10.1109/ jsen.2021.3123243	IEEE	duplicate	no
deep learning-based image enhancement for robust remote photoplethysmography in various illumination scenarios	2023	10.1109/ cvprw59228.2023.00 647	IEEE	duplicate	no
deep neural network to remove motion artifacts from heart rate sensor embedded on handle cane	2024	10.1109/ sensors60989.2024. 10784567	IEEE	denoising	no
deep super-resolution network for rppg information recovery and noncontact heart rate estimation	2021	10.1109/ tim.2021.3109398	IEEE	off topic	no
deepheart: accurate heart rate estimation from ppg signals based on deep learning	2019	10.1109/ mass.2019.00051	IEEE	duplicate	no
deeppulse: an uncertainty-aware deep neural network for heart rate estimations from wrist-worn photoplethysmography	2022	10.1109/ embc48229.2022.98 71813	IEEE	duplicate	no
delnet based systolic peak delineation in remote ppg signal from facial video frames	2023	10.1109/ icccnt56998.2023.10 306618	IEEE	duplicate	no
deriving heart rate and respiratory rate from pulse oximetry using neural networks	2021	10.1109/ hnicem54116.2021.9 731861	IEEE	duplicate	no
design a non-invasive pulse oximeter device based on pic microcontroller	2019	10.1109/ icpet.2019.00027	IEEE	hardware	no

design and development of a wristband for continuous vital signs monitoring of covid-19 patients	2021	10.1109/embc46164.2021.9630299	IEEE	duplicate	no
design and development of variscan: a continuous heart rate variability monitor	2024	10.1109/ises63344.2024.00044	IEEE	hardware	no
design and implementation of 1024 point pipelined radix 4 fft processor on fpga for biomedical signal processing applications	2020	10.1109/ises50453.2020.00012	IEEE	duplicate	no
design and implementation of auscultation blood pressure measurement using vascular transit time and physiological parameters	2017	10.1109/smc.2017.8123084	IEEE	duplicate	no
design of a low-cost wearable heart and respiratory rate measurement device using an arduino and bluetooth module	2021	10.1109/icccnt51525.2021.9579536	IEEE	duplicate	no
design of false heart rate feedback system for improving game experience	2018	10.1109/icce.2018.8326254	IEEE	off topic	no
detection of beat-to-beat intervals from wrist photoplethysmography in patients with sinus rhythm and atrial fibrillation after surgery	2018	10.1109/bhi.2018.8333387	IEEE	duplicate	no
detection of vital parameters using cloud computing technology	2020	10.1109/iccsp48568.2020.9182164	IEEE	off topic	no
determination of absolute heart beat from photoplethysmographic signals in the presence of motion artifacts	2018	10.1109/icaecc.2018.8479479	IEEE	duplicate	no
development and validation of heart rate -incorporated finger photoplethysmography fitness index algorithm for cardiovascular disease risk assessment	2018	10.1109/iecbes.2018.8626725	IEEE	off topic	no
development of a custom wrist wearable for use in nursing homes	2023	10.1145/3580252.3589434	IEEE	duplicate	no
development of a miniaturized, low-power, head-mounted pcb for continuous ppg monitoring and real-time hr estimation	2024	10.1109/sas60918.2024.10636362	IEEE	hardware	no
development of a wearable in-ear ppg system for continuous monitoring	2020	10.1109/jsen.2020.3008479	IEEE	duplicate	no

development of smart mask system integrated with alert detection and vital-sign measurement	2024	10.1109/ smc54092.2024.10831057	IEEE	off topic	no
direct estimation vs. indirect metrics: machine learning techniques for cardiac output estimation	2024	10.1109/ sensors60989.2024.10784599	IEEE	off topic	no
dlprppg: development and design of deep learning platform for remote photoplethysmography	2022	10.1109/ iscas48785.2022.9937698	IEEE	off topic	no
edge-based computation of super-resolution superlet spectrograms for real-time estimation of heart rate using an iomt-based reference-signal-less ppg sensor	2024	10.1109/ jiot.2023.3322947	IEEE	duplicate	no
effect of ambient lighting and skin tone on estimation of heart rate and pulse transit time from video plethysmography	2020	10.1109/ embc44109.2020.9176731	IEEE	duplicate	no
effect of position and fastening belt pressure on the accuracy of ppg-based heart rate sensor	2018	10.1109/ embc.2018.8513333	IEEE	duplicate	no
effect of skin tone and activity on the performance of wrist-worn optical beat-to-beat heart rate monitoring	2020	10.1109/ sensors47125.2020.9278523	IEEE	off topic	no
elicitation of anxiety without time pressure and its detection using physiological signals and artificial intelligence: a proof of concept	2024	10.1109/ access.2024.3362668	IEEE	duplicate	no
embedded low power heart rate estimation processor for flexible applications	2022	10.1109/ ifetc53656.2022.9948467	IEEE	duplicate	no
embedded multi-sensor smartwatch for computationally intensive biosignal processing	2024	10.1109/ biocas61083.2024.10798302	IEEE	hardware	no
emotion based media playback system using ppg signal	2021	10.1109/ wispnet51692.2021.9419475	IEEE	duplicate	no
end-to-end ppg processing pipeline for wearables: from quality assessment and motion artifacts removal to hr/hrv feature extraction	2023	10.1109/ bibm58861.2023.10385998	IEEE	duplicate	no
energy-efficient photoplethysmogram compression to estimate heart and respiratory rates simultaneously	2019	10.1109/ access.2019.2919745	IEEE	duplicate	no

energy-efficient wearable-to-mobile offload of ml inference for ppg-based heart-rate estimation	2023	10.23919/ date56975.2023.10137129	IEEE	duplicate	no
enhance heart rate measurement from remote ppg with head motion awareness from image	2024	10.1109/ embc53108.2024.10782369	IEEE	off topic	no
enhanced contactless heart rate monitoring using camera with motion artifact removal during physical activities	2023	10.1109/ embc40787.2023.10340279	IEEE	duplicate	no
enhanced deepphys: leveraging deep learning for heart rate detection from facial videos	2024	10.1109/ niles63360.2024.10753140	IEEE	off topic	no
enhancement of remote ppg and heart rate estimation with optimal signal quality index	2022	10.1109/ bsn56160.2022.9928503	IEEE	duplicate	no
enhancement of stress classification using web camera-based imaging photoplethysmography with a frame alignment method	2024	10.1109/ access.2024.3452934	IEEE	off topic	no
enhancing beat-to-beat analysis of heart signals with respiration harmonics reduction through demodulation and template matching	2024	10.1109/ tmtt.2023.3324444	IEEE	off topic	no
ensemble empirical mode decomposition with principal component analysis: a novel approach for extracting respiratory rate and heart rate from photoplethysmographic signal	2018	10.1109/ jbhi.2017.2679108	IEEE	duplicate	no
estimating heart rate during steady-state activities and transitions based on signal of ppg and acc	2020	10.1109/ icma49215.2020.9233764	IEEE	duplicate	no
estimating heart rate variability from wrist-worn photoplethysmography devices in daily activities: a preliminary convolutional denoising autoencoder approach	2024	10.1109/ metroxraine62247.2024.10796284	IEEE	denoising	no
estimating vital signs through non-contact video-based approaches: a survey	2017	10.1109/ rise.2017.8378141	IEEE	off topic	no
estimation and validation of arterial blood pressure using photoplethysmogram morphology features in conjunction with pulse arrival time in large open databases	2021	10.1109/ jbhi.2020.3009658	IEEE	duplicate	no

estimation of beat-by-beat blood pressure and heart rate from ecg and ppg using a fine-tuned deep cnn model	2022	10.1109/ access.2022.3195857	IEEE	duplicate	no
estimation of beat-to-beat interval from wearable photoplethysmography sensor on different measurement sites during daily activities	2018	10.1109/ icsens.2018.8589611	IEEE	duplicate	no
estimation of breathing rate and heart rate from photoplethysmogram	2017	10.1109/ iceei.2017.8312414	IEEE	duplicate	no
estimation of hrv and spo2 from wrist-worn commercial sensors for clinical settings	2018	10.1109/ bsn.2018.8329679	IEEE	duplicate	no
exploiting tunable q-factor wavelet transform domain sparsity to denoise wrist ppg signals	2023	10.1109/ tim.2023.3287248	IEEE	off topic	no
extraction of heart rate from photoplethysmography signals on multiple sites for wearable heart monitoring systems	2024	10.1109/ tencon61640.2024.10902829	IEEE	off topic	no
extraction of heart rate from ppg signal: a machine learning approach using decision tree regression algorithm	2019	10.1109/ eict48899.2019.9068845	IEEE	duplicate	no
facial remote photoplethysmography for continuous heart rate monitoring during prolonged cold liquid bolus administration	2024	10.1109/ embc53108.2024.10781709	IEEE	off topic	no
facial video-based remote photoplethysmography signal estimation with vision transformer	2023	10.1109/icce-asia59966.2023.10326412	IEEE	duplicate	no
facial-video-based physiological signal measurement: recent advances and affective applications	2021	10.1109/ msp.2021.3106285	IEEE	duplicate	no
feasibility of a wireless vital signal monitoring system in the nicu	2023	10.1109/ bsn58485.2023.10331194	IEEE	duplicate	no
feasibility study of deep neural network for heart rate estimation from wearable photoplethysmography and acceleration signals	2019	10.1109/ embc.2019.8857618	IEEE	duplicate	no
feature-level cross-attentional ppg and motion signal fusion for heart rate estimation	2023	10.1109/ compsac57700.2023.00267	IEEE	duplicate	no
fingertip plethysmography using a sensitive wafer-level-packaged capacitive mems strain sensor	2024	10.1109/ mems58180.2024.10439370	IEEE	duplicate	no

finite state machine framework for instantaneous heart rate validation using wearable photoplethysmography during intensive exercise	2019	10.1109/ jbhi.2018.2871177	IEEE	duplicate	no
freq2time: weakly supervised learning of camera-based rppg from heart rate	2024	10.1109/ icassp48485.2024.1 0446054	IEEE	duplicate	no
from sprint to recovery: lstm-powered heart rate recovery forecasting in hiit sessions	2024	10.1109/ embc53108.2024.10 781668	IEEE	multichannel	no
functional model of device of bracelet type based on photoplethysmography	2024	10.1109/ epei63510.2024.107 58131	IEEE	hardware	no
guest editorial camera-based health monitoring in real-world scenarios	2024	10.1109/ jbhi.2023.3348248	IEEE	off topic	no
heart pulse demodulation from emfit mattress sensor using spectral and source separation techniques	2022	10.22489/ cinc.2022.304	IEEE	duplicate	no
heart rate analysis through smartphone camera	2021	10.1109/ metroind4.0iot51437 .2021.9488509	IEEE	duplicate	no
heart rate and breathing rate calculated from cheeks and lips using green and derived colors from video	2020	10.1109/ ises50453.2020.000 16	IEEE	duplicate	no
heart rate and heart rate variability as classification features for mental fatigue using short-term ppg signals via smartphones instead of ecg recordings	2021	10.1109/ iccsn52437.2021.94 63614	IEEE	duplicate	no
heart rate estimation algorithm from wrist-based photoplethysmogram using subspace learning method	2019	10.1109/ percomw.2019.8730 797	IEEE	duplicate	no
heart rate estimation during exercise from photoplethysmographic signals using convolutional neural network	2019	10.1109/ biocas.2019.891918 5	IEEE	duplicate	no
heart rate estimation from facial video sequences using fast independent component analysis	2022	10.1109/ ncc55593.2022.9806 810	IEEE	duplicate	no
heart rate estimation from facial videos using 2d convolution neural network	2024	10.1109/ imsa61967.2024.106 52792	IEEE	off topic	no

heart rate estimation from neck photoplethysmography using fft-based scoring and a shallow neural network	2024	10.1109/embc53108.2024.10781693	IEEE	off topic	yes
heart rate estimation from photoplethysmography signals using fast fourier transform	2024	10.1109/icspis63676.2024.10812645	IEEE	hardware	no
heart rate estimation from remote photoplethysmography based on light-weight u-net and attention modules	2023	10.1109/access.2023.3281898	IEEE	duplicate	no
heart rate estimation from wrist-type photoplethysmographic signals corrupted by intense motion artifacts using nlms adaptive filter and spectral peak tracking	2019	10.1109/icaiit.2019.8834647	IEEE	duplicate	no
heart rate estimation from wrist-worn photoplethysmography: a review	2019	10.1109/jsen.2019.2914166	IEEE	duplicate	no
heart rate estimation of ppg signals with simultaneous accelerometry using adaptive neural network filtering	2020	10.1109/tce.2019.2961263	IEEE	duplicate	no
heart rate estimation using on-nail wearable photoplethysmography	2024	10.1109/embc53108.2024.10782437	IEEE	hardware	yes
heart rate extraction from novel neck photoplethysmography signals	2019	10.1109/embc.2019.8857415	IEEE	duplicate	no
heart rate extraction from photoplethysmography signal: a multi model machine learning approach	2020	10.1109/iicaiet49801.2020.9257869	IEEE	duplicate	no
heart rate imputation using accelerometers for wearable devices	2024	10.1109/embc53108.2024.10781795	IEEE	denoising	yes
heart rate measurement on pc and phone using facial videos	2023	10.1109/kst57286.2023.10086729	IEEE	duplicate	no
heart rate monitoring during physical exercise from photoplethysmography using neural network	2019	10.1109/lsens.2018.2878207	IEEE	duplicate	no
heart rate monitoring using ppg with smartphone camera	2021	10.1109/bibm52615.2021.9669735	IEEE	duplicate	no

heart rate monitoring using sparse spectral curve tracing	2020	10.1109/embc44109.2020.9175349	IEEE	duplicate	no
heart rate variability analysis from electrocardiogram (ecg) and photoplethysmogram (ppg) signals by using soft computing technique	2023	10.1109/ihcsp56702.2023.10127200	IEEE	duplicate	no
heart rate variability extraction from videos signals: ica vs. evm comparison	2017	10.1109/access.2017.2678521	IEEE	duplicate	no
heart: motion-resilient heart rate monitoring with in-ear microphones	2023	10.1109/percom56429.2023.10099317	IEEE	duplicate	no
heartbeat rate estimation using convolutional neural network	2024	10.1109/icict60155.2024.10544653	IEEE	duplicate	no
high fidelity mzi-bcg sensor with homodyne demodulation for unobtrusive hr and bp monitoring	2022	10.1109/jsen.2022.3158070	IEEE	off topic	no
high-responsivity van der waals schottky photodiodes based on maskless etched wafer-scale silicon nanoholes	2024	10.1109/led.2024.3376001	IEEE	duplicate	no
hreadai: heart rate estimation from face mask videos by consolidating eulerian and lagrangian approaches	2024	10.1109/tim.2023.3334359	IEEE	duplicate	no
hrunet: assessing uncertainty in heart rates measured from facial videos	2024	10.1109/jbhi.2024.3363006	IEEE	off topic	no
impact of bilateral thigh occlusion on the remote photoplethysmography signal recorded from the palm	2024	10.1109/nap62956.2024.10739727	IEEE	off topic	no
impact of finger contact force on aortic waveform parameters derived from finger photoplethysmography using a transfer function	2024	10.1109/sensors60989.2024.10785128	IEEE	off topic	no
implementation of adaptive threshold for peak detection of photoplethysmography applied on microcontroller	2019	10.1109/iceeie47180.2019.8981423	IEEE	duplicate	no
improved heart rate estimation from photoplethysmography during physical exercise using combination of nlms and rls adaptive filters	2018	10.1109/tencon.2018.8650495	IEEE	duplicate	no
improved heart rate tracking using multiple wrist-type photoplethysmography during physical activities	2018	10.1109/embc.2018.8512736	IEEE	duplicate	no

improving heart rate and heart rate variability estimation from video through a hr-rr-tuned filter	2023	10.1109/ icassp49357.2023.10096576	IEEE	duplicate	no
improving heart rate estimation on consumer grade wrist-worn device using post-calibration approach	2020	10.1109/ jsen.2020.2979191	IEEE	duplicate	no
improving ppg-based heart-rate monitoring with synthetically generated data	2022	10.1109/ biocas54905.2022.9948584	IEEE	duplicate	no
in your ear: a multimodal hearables device for the assessment of the state of body and mind	2023	10.1109/ mpuls.2024.3357008	IEEE	duplicate	no
in-ear ppg for vital signs	2022	10.1109/ mprv.2021.3121171	IEEE	duplicate	no
influence of roi selection for remote photoplethysmography with singular spectrum analysis	2021	10.1109/ aiid51893.2021.9456548	IEEE	off topic	no
integrated smart patch for heart rate and respiratory rate monitoring	2023	10.23919/ measurement59122.2023.10164383	IEEE	duplicate	no
investigation on pulse wave forward peak detection and its applications in cardiovascular health	2022	10.1109/ tbme.2021.3103552	IEEE	duplicate	no
kick ring II: a multi-sensor ring capturing respiration, electrocardiogram, oxygen saturation, and skin temperature1	2020	10.1109/ embc44109.2020.9176654	IEEE	duplicate	no
learning based quality indicator aiding heart rate estimation in wrist-worn ppg	2021	10.1109/ embc46164.2021.9630910	IEEE	duplicate	no
learning to estimate heart rate from accelerometer and user's demographics during physical exercises	2024	10.1109/ jbhi.2023.3251742	IEEE	inaccessible dataset	no
lightweight neural network based model for real-time precise hr monitoring during high intensity workout using consumer smartwatches	2022	10.1109/ embc48229.2022.9871612	IEEE	inaccessible dataset	no
live demonstration: a ring-type blood pressure monitoring system based on photoplesthygraphy	2017	10.1109/ biocas.2017.8325091	IEEE	duplicate	no
live demonstration: a smart ring for continuous health data monitoring based on photoplethysmography	2023	10.1109/ aicas57966.2023.10168644	IEEE	duplicate	no

live demonstration: a wearable wireless medical sensor network system towards internet-of-patients	2018	10.1109/icsens.2018.8589897	IEEE	duplicate	no
low complexity heart rate measurement from wearable wrist-type photoplethysmographic sensors robust to motion artifacts	2018	10.1109/icassp.2018.8461520	IEEE	duplicate	no
low intricate digital twin method to predict cardiac arrhythmia	2023	10.1109/iceccme57830.2023.10252270	IEEE	duplicate	no
low-cost microcontrolled based wireless heart rate and oxygen saturation monitor	2018	10.1109/conielecomp.2018.8327195	IEEE	duplicate	no
low-power continuous heart and respiration rates monitoring on wearable devices	2019	10.1109/icassp.2019.8683825	IEEE	duplicate	no
low-power high-sensitivity photoplethysmography sensor for wearable health monitoring system	2021	10.1109/jsen.2021.3062189	IEEE	duplicate	no
low-power organic led fabricated by a novel solution-based process for photoplethysmography sensing	2023	10.1109/jflex.2023.3259384	IEEE	hardware	no
lsdascformer: a transformer-like network with long–short-distance attention and scconv for heart rate measurement	2024	10.1109/jsen.2024.3429163	IEEE	off topic	no
lstm-only model for low-complexity hr estimation from wrist ppg	2021	10.1109/embc46164.2021.9630942	IEEE	duplicate	no
matlab gui design for heart rate monitoring using smart phone camera	2017	10.1109/tiptekno.2017.8238030	IEEE	duplicate	no
measurement of wrist pulse using fiber optic mzi for analysis of heart rate and respiration rate	2022	10.1109/tencon55691.2022.9977703	IEEE	off topic	no
measuring heart rate and heart rate variability with smartphone camera	2021	10.1109/mdm52706.2021.00049	IEEE	duplicate	no
measuring heart rate during physical exercise by subspace decomposition and kalman smoothing	2018	10.1109/tim.2017.2770818	IEEE	duplicate	no

modified squirrel algorithm for fault tolerance using photoplethysmography in iot applications	2021	10.1109/ iceca52323.2021.9676083	IEEE	duplicate	no
modulation model of the photoplethysmography signal for vital sign extraction	2021	10.1109/ jbhi.2020.3013811	IEEE	duplicate	no
monitoring clinical parameters through machine learning and image processing	2024	10.1109/ icsd60021.2024.10751023	IEEE	off topic	no
motion artifact canceling ppg heart rate sensor based on an adaptive filter algorithm with variable tap length	2020	10.1109/ embc44109.2020.9176715	IEEE	duplicate	no
motion artifact cancellation in wearable photoplethysmography using gyroscope	2019	10.1109/ jsen.2018.2879970	IEEE	duplicate	no
motion artifact mitigation for wearable pulse oximetry	2018	10.1109/ bsn.2018.8329662	IEEE	duplicate	no
motion artifact reduction from ppg signals during intense exercise using filtered x-lms	2017	10.1109/ iscas.2017.8050418	IEEE	duplicate	no
motion artifact removal and feature extraction from ppg signals using efficient signal processing algorithms	2018	10.1109/ icacci.2018.8554599	IEEE	duplicate	no
motion artifact removal for ppg signals based on accurate fundamental frequency estimation and notch filtering	2018	10.1109/ embc.2018.8512878	IEEE	duplicate	no
motion-robust multimodal heart rate estimation using bcg fused remote-ppg with deep facial roi tracker and pose constrained kalman filter	2021	10.1109/ tim.2021.3060572	IEEE	duplicate	no
movement, sweating, and contact pressure as sources of heart rate inaccuracy in wearable devices	2022	10.22489/ cinc.2022.232	IEEE	duplicate	no
movement, sweating, and contact pressure as sources of heart rate inaccuracy in wearable devices	2022	10.22489/ cinc.2022.237	IEEE	duplicate	no
multi-mode particle filtering methods for heart rate estimation from wearable photoplethysmography	2019	10.1109/ tbme.2019.2895685	IEEE	duplicate	no
multi-physiological parameters integrated medical system for home healthcare application	2021	10.1109/ asicon52560.2021.9620524	IEEE	duplicate	no
multibiometric system based on face video and heart rate estimation	2021	10.1109/ iwbf50991.2021.9465081	IEEE	duplicate	no

mvpd: a multimodal video physiology database for rppg	2023	10.1109/ seai59139.2023.102 17565	IEEE	duplicate	no
nas-ppg: ppg-based heart rate estimation using neural architecture search	2021	10.1109/ jsen.2021.3073047	IEEE	duplicate	no
networked wearable sensors for monitoring health and activities of an equine herd: an iot approach to improve horse welfare	2024	10.1109/ jsen.2024.3436665	IEEE	animal	no
non-contact fingertip microwave plethysmography based on near-field sensing with super-regenerative oscillator	2021	10.1109/ ims19712.2021.9574 888	IEEE	duplicate	no
non-contact heart rate measurement from facial video data using a 2d-vmd scheme	2022	10.1109/ jsen.2022.3169963	IEEE	duplicate	no
non-contact heart rate monitoring in neonatal intensive care unit using rgb camera	2020	10.1109/ embc44109.2020.91 75651	IEEE	duplicate	no
non-contact hr extraction from different color spaces using rgb camera	2022	10.1109/ ncc55593.2022.9806 722	IEEE	off topic	no
non-contact hr monitoring via smartphone and webcam during different respiratory maneuvers and body movements	2021	10.1109/ jbhi.2020.2998399	IEEE	off topic	no
non-contact methods for heart rate measurement: a review	2021	10.1109/ iccs54944.2021.000 64	IEEE	off topic	no
non-invasive blood pressure and heart rate sensing using photoplethysmogram sensor	2021	10.1109/ cspa52141.2021.937 7302	IEEE	duplicate	no
non-invasive blood pressure monitoring using a single-channel ppg sensor with adaptive kalman algorithm and 4-led arrayed structure	2024	10.1109/ tim.2024.3480192	IEEE	off topic	no
noncontact blood pressure estimation using bp-related cardiovascular knowledge: an uncalibrated method based on consumer-level camera	2023	10.1109/ tim.2023.3279448	IEEE	off topic	no
noncontact cardiac parameters estimation using radar acoustics for healthcare iot	2024	10.1109/ jiot.2023.3317670	IEEE	off topic	no
noncontact heart rate measurement system on single board computer	2018	10.23919/ icep.2018.8374314	IEEE	off topic	no

noncontact multiphysiological signals estimation via visible and infrared facial features fusion	2022	10.1109/ tim.2022.3209750	IEEE	off topic	no
noninvasive heart rate and blood glucose level estimation using photoplethysmography	2021	10.1109/ icict4sd50815.2021. 9396849	IEEE	duplicate	no
on the development of a wearable multi-spectral photoplethysmographic device for heart rate detection	2023	10.1109/ aisp57993.2023.101 34818	IEEE	inaccessible dataset	no
on the use of wavelet transform based adaptive filtering for de-noising of pulse oximeter signals	2021	10.1109/ i2mtc50364.2021.94 59833	IEEE	duplicate	no
on-device signal quality guided and embedded physiologic information for high fidelity continuous ppg compression	2024	10.1109/ tim.2024.3351256	IEEE	duplicate	no
p2e-lgan: ppg to ecg reconstruction methodology using lstm based generative adversarial network	2024	10.1109/ iscas58744.2024.10 558493	IEEE	duplicate	no
parameter optimization of motion artifact canceling ppg-based heart rate sensor by means of cross validation	2017	10.1109/ ismict.2017.7891771	IEEE	duplicate	no
parhelia: particle filter-based heart rate estimation from photoplethysmographic signals during physical exercise	2018	10.1109/ tbme.2017.2697911	IEEE	duplicate	no
patient-provider interaction system for efficient home-based cardiac rehabilitation exercise	2019	10.1109/ access.2019.289271 7	IEEE	off topic	no
peak frequency tracking versus outlier rejection for photoplethysmographic heart rate sensing during exercise	2020	10.1109/ ismict48699.2020.91 52726	IEEE	duplicate	no
performance analysis of adaptive filter and machine learning algorithms for heart rate estimation using ppg signal	2021	10.1109/ ict4da53266.2021.96 72242	IEEE	duplicate	no
personalized stress monitoring using wearable sensors in everyday settings	2021	10.1109/ embc46164.2021.96 30224	IEEE	duplicate	no
photoplethysmographic waveform analysis for autonomic reactivity assessment in depression	2021	10.1109/ tbme.2020.3025908	IEEE	duplicate	no

photoplethysmography-based heart action monitoring using a growing multilayer network	2023	10.1109/ jsen.2022.3228517	IEEE	duplicate	no
physiolstm: a novel 1d architecture for non-contact hr monitoring	2024	10.1109/ memea60663.2024. 10596855	IEEE	off topic	no
physiopatch: a multimodal and adaptable wearable patch for cardiovascular and cardiopulmonary assessment	2024	10.1109/ jsen.2024.3403846	IEEE	duplicate	no
portable and real-time iot-based healthcare monitoring system for daily medical applications	2023	10.1109/ tcss.2022.3207562	IEEE	duplicate	no
power-aware heart rate monitoring using particle filters	2021	10.1109/ islped52811.2021.95 02471	IEEE	duplicate	no
pp-net: a deep learning framework for ppg-based blood pressure and heart rate estimation	2020	10.1109/ jsen.2020.2990864	IEEE	duplicate	no
ppg derived heart rate estimation during intensive physical exercise	2019	10.1109/ access.2019.291314 8	IEEE	duplicate	no
ppg-based heart rate estimation using wiener filter, phase vocoder and viterbi decoding	2017	10.1109/ icassp.2017.795230 9	IEEE	duplicate	no
ppg-based heart rate estimation with efficient sensor sampling and learning models	2022	10.1109/hpcc-dss-smartcity-dependsys57074.20 22.00294	IEEE	duplicate	no
ppg-gan: an adversarial network to de-noise ppg signals during physical activity	2022	10.1109/ healthcom54947.202 2.9982757	IEEE	duplicate	no
ppgnet: deep network for device independent heart rate estimation from photoplethysmogram	2019	10.1109/ embc.2019.8856989	IEEE	duplicate	no
prediction of noise-type and its elimination from photoplethysmograph signal	2024	10.1109/ ciec59440.2024.104 68270	IEEE	duplicate	no
privacy protected contactless cardio-respiratory monitoring using defocused cameras during sleep	2023	10.1109/ healthcom56612.202 3.10472346	IEEE	duplicate	no
privacy-protected contactless sleep parameters measurement using a defocused camera	2024	10.1109/ jbhi.2024.3396397	IEEE	off topic	no

pulsegan: learning to generate realistic pulse waveforms in remote photoplethysmography	2021	10.1109/ jbhi.2021.3051176	IEEE	off topic	no
q-ppg: energy-efficient ppg-based heart rate monitoring on wearable devices	2021	10.1109/ tbcas.2021.3122017	IEEE	duplicate	no
rapid vital sign extraction for real-time opto-physiological monitoring at varying physical activity intensity levels	2023	10.1109/ jbhi.2023.3268240	IEEE	duplicate	no
real-time heart rate estimation algorithm based on adaptive spectrum correction and peak localization	2024	10.1109/ jsen.2024.3444038	IEEE	inaccessible dataset	no
real-time heartbeat sensing with face video using a webcam and opencv	2023	10.1109/ cises58720.2023.10 183446	IEEE	duplicate	no
real-time hr estimation from wrist ppg using binary lstms	2019	10.1109/ biocas.2019.891872 6	IEEE	duplicate	no
real-time ppg-to-ecg reconstruction model with on-device recalibration facility	2024	10.1109/ tim.2024.3450120	IEEE	off topic	no
real-time pulse oximetry extraction using a lightweight algorithm and a task pipeline scheme	2021	10.1109/ mocast52088.2021.9 493400	IEEE	duplicate	no
real-time robust heart rate estimation from wrist-type ppg signals using multiple reference adaptive noise cancellation	2018	10.1109/ jbhi.2016.2632201	IEEE	duplicate	no
real-time stress evaluation using wireless body sensor networks	2018	10.1109/ wd.2018.8361691	IEEE	off topic	no
real-time video-based heart and respiration rate monitoring	2021	10.1109/ naecon49338.2021. 9696378	IEEE	duplicate	no
recent innovations and improvements in remote heart rate and heart disease measuring methods using rgb camera	2023	10.1109/ iccpct58313.2023.10 245666	IEEE	duplicate	no
reconstruction of corrupted photoplethysmography signals to facilitate continuous monitoring	2023	10.22489/ cinc.2023.121	IEEE	duplicate	no
reconstruction of corrupted photoplethysmography signals using recursive generative adversarial networks	2024	10.1109/ tim.2023.3335524	IEEE	duplicate	no
reflectance-based monolithic organic pulsemeter device for measuring photoplethysmogram signal	2018	10.1109/ i2mtc.2018.8409873	IEEE	duplicate	no

region of interest analysis using delaunay triangulation for facial video-based heart rate estimation	2024	10.1109/ tim.2024.3363786	IEEE	off topic	no
relaxation assessment based on heart rate variability and heart rate using photoplethsmograms	2022	10.1109/ispce-asia57917.2022.9970859	IEEE	duplicate	no
reliability estimation of heart rate measurement using wrist-worn devices	2021	10.23919/ icmu50196.2021.9638918	IEEE	off topic	no
remote health monitoring system for the estimation of blood pressure, heart rate, and blood oxygen saturation level	2023	10.1109/ jsen.2023.3235977	IEEE	duplicate	no
remote heart rate measurement from rgb-nir video based on spatial and spectral face patch selection	2018	10.1109/ embc.2018.8513464	IEEE	off topic	no
remote heart rate monitoring in smart environments from videos with self-supervised pretraining	2024	10.1109/ jiot.2023.3327623	IEEE	duplicate	no
remote photoplethysmography and heart rate estimation by dynamic region of interest tracking	2022	10.1109/ embc48229.2022.9871722	IEEE	duplicate	no
remote photoplethysmography measurement using constrained ica	2017	10.1109/ ehb.2017.7995453	IEEE	off topic	no
remote photoplethysmography signals enhancement based on generative adversarial networks	2023	10.1109/ iciba56860.2023.10165239	IEEE	off topic	no
remote photoplethysmography using nonlinear mode decomposition	2018	10.1109/ icassp.2018.8462538	IEEE	duplicate	no
remote photoplethysmography: rarely considered factors	2020	10.1109/ cvprw50498.2020.00156	IEEE	off topic	no
respiratory rate estimation from face videos	2019	10.1109/ bhi.2019.8834499	IEEE	off topic	no
respiratory rate estimations using three respiratory-induced variations on photoplethysmogram	2021	10.1109/ iceei52609.2021.9611130	IEEE	duplicate	no
rgb and near-infrared light reflectance/transmittance photoplethysmography for measuring heart rate during motion	2020	10.1109/ access.2020.2990438	IEEE	duplicate	no

robot assisted instantaneous heart rate estimator using camera based remote photoplethysmograpy via plane-orthogonal-to-skin and finite state machine	2020	10.1109/ embc44109.2020.9176648	IEEE	off topic	no
robust and computationally efficient approach for heart rate monitoring using photoplethysmographic signals during intensive physical excercise	2017	10.1109/ mercon.2017.7980497	IEEE	duplicate	no
robust de-noising technique for accurate heart rate estimation using wrist-type ppg signals	2020	10.1109/ jsen.2020.2982540	IEEE	duplicate	no
robust estimation of pulse rate from a wrist-type ppg during intensive exercise	2018	10.1109/ embc.2018.8513584	IEEE	inaccessible dataset	no
robust heart rate detection via multi-site photoplethysmography	2024	10.1109/ embc53108.2024.10782155	IEEE	multichannel	no
robust heart rate estimation during physical exercise using photoplethysmographic signals	2018	10.1109/ embc.2018.8512405	IEEE	duplicate	no
robust heart rate estimation from ppg signals with intense motion artifacts using cascade of adaptive filter and recurrent neural network	2019	10.1109/ tencon.2019.8929692	IEEE	duplicate	no
robust heart rate estimation with spatial–temporal attention network from facial videos	2022	10.1109/ tcds.2021.3062370	IEEE	off topic	no
robust heart rate monitoring by a single wrist-worn accelerometer based on signal decomposition	2021	10.1109/ jsen.2021.3075109	IEEE	duplicate	no
robust heart rate monitoring for quasi-periodic motions by wrist-type ppg signals	2020	10.1109/ jbhi.2019.2912708	IEEE	duplicate	no
robust motion artefact resistant circuit for calculation of mean arterial pressure from pulse transit time	2017	10.1109/ embc.2017.8037624	IEEE	duplicate	no
robust ppg-based ambulatory heart rate tracking algorithm	2020	10.1109/ embc44109.2020.9175346	IEEE	duplicate	no
rppg-based heart rate estimation using spatial-temporal attention network	2022	10.1109/ tcds.2021.3131197	IEEE	off topic	no
rtrppg: an ultra light 3dcnn for real-time remote photoplethysmography	2022	10.1109/ cvprw56347.2022.00233	IEEE	off topic	no
self-rppg: learning the optical & physiological mechanics of remote photoplethysmography with self-supervision	2022	10.1145/3551455.3559609	IEEE	duplicate	no

self-supervised rgb-nir fusion video vision transformer framework for rppg estimation	2022	10.1109/ tim.2022.3217867	IEEE	off topic	no
serial fusion of eulerian and lagrangian approaches for accurate heart-rate estimation using face videos	2017	10.1109/ embc.2017.8037447	IEEE	off topic	no
signal quality assessment for wearable multichannel photoplethysmography signals	2023	10.1109/ apsipaasc58517.2023.10317377	IEEE	duplicate	no
single-channel impedance plethysmography neck patch device for unobtrusive wearable cardiovascular monitoring	2020	10.1109/ access.2020.3029604	IEEE	hardware	no
sleep monitoring with intraorally measured photoplethysmography (ppg) signals	2022	10.1109/ sensors52175.2022.9967075	IEEE	duplicate	no
sleep/wake classification via remote ppg signals	2019	10.1109/ embc.2019.8857097	IEEE	duplicate	no
smart mandibular advancement device for intraoral monitoring of cardiorespiratory parameters and sleeping postures	2021	10.1109/ tbcas.2021.3065824	IEEE	duplicate	no
smartwatch ppg peak detection method for sinus rhythm and cardiac arrhythmia	2019	10.1109/ embc.2019.8857325	IEEE	duplicate	no
snr-based signal selection framework for heart rate estimation using rppg signals extracted from facial videos	2024	10.1109/ icbme64381.2024.10895960	IEEE	off topic	no
spatial-spectral-temporal fusion for remote heart rate estimation	2020	10.1109/ jsen.2020.2997785	IEEE	off topic	no
spectral reflectance based heart rate measurement from facial video	2019	10.1109/ icip.2019.8803508	IEEE	off topic	no
srppg: semi-supervised adversarial learning for remote photoplethysmography with noisy data	2023	10.1109/ smartcomp58114.2023.00021	IEEE	duplicate	no
stochastic modeling based nonlinear bayesian filtering for photoplethysmography denoising in wearable devices	2020	10.1109/ tii.2020.2988097	IEEE	duplicate	no
stress detection using cnn on the wesad dataset	2024	10.1109/ esic60604.2024.10481604	IEEE	duplicate	no

stretchable optical sensing patch system integrated heart rate, pulse oxygen saturation, and sweat ph detection	2019	10.1109/ tbme.2018.2866151	IEEE	hardware	no
strong robustness heart rate estimation using discrete fourier transform and personality heart rate characteristic	2017	10.23919/ chicc.2017.8028794	IEEE	duplicate	no
stsr: spectro-temporal super-resolution analysis of a reference signal less photoplethysmogram for heart rate estimation during physical activity	2022	10.1109/ tim.2022.3192831	IEEE	duplicate	no
style transfer with bio-realistic appearance manipulation for skin-tone inclusive rppg	2022	10.1109/ iccp54855.2022.9887649	IEEE	off topic	no
summit vitals: multi-camera and multi-signal biosensing at high altitudes	2024	10.1109/ swc62898.2024.00072	IEEE	off topic	no
sustained attention detection system in learning environments	2020	10.1109/ epe50722.2020.9305027	IEEE	duplicate	no
synthesis of photoplethysmograph signals from noises with desired heart and respiration rates	2024	10.1109/ ciec59440.2024.10468279	IEEE	duplicate	no
taylor-fourier analysis of photoplethysmography signals for heart rate measurement	2024	10.1109/ i2mtc60896.2024.10560902	IEEE	duplicate	no
the use of wrist emg increases the ppg heart rate accuracy in smartwatches	2022	10.1109/ jsen.2022.3219297	IEEE	duplicate	no
time–frequency learning framework for rppg signal estimation using scalogram-based feature map of facial video data	2023	10.1109/ tim.2023.3287243	IEEE	off topic	no
toward a universal bcg validation using a mechanical emulator	2024	10.1109/ i2mtc60896.2024.10560923	IEEE	duplicate	no
toward nonintrusive camera-based heart rate variability estimation in the car under naturalistic condition	2022	10.1109/ jiot.2021.3131742	IEEE	duplicate	no
towards low-power heart rate estimation based on user’s demographics and activity level for wearables	2023	10.1109/ icassp49357.2023.10095339	IEEE	duplicate	no

towards quantifying stress in patients with a history of myocardial infarction: validating ecg-derived patch features	2023	10.1109/ embc40787.2023.10 340614	IEEE	duplicate	no
towards wearable acute stress detection and mitigation via real-time photoplethysmogram feature detection	2024	10.1109/ sensors60989.2024. 10784720	IEEE	off topic	no
under display ambient light sensor for non-invasive vital signs monitoring	2024	10.1109/ sensors60989.2024. 10784918	IEEE	off topic	no
unsupervised enhancement of classical remote ppg algorithms using 1d-cnn and contrastive loss for accurate heart rate estimation	2024	10.1109/ memea60663.2024. 10596846	IEEE	off topic	no
using consumer-graded wearable devices for sleep apnea pre-diagnosis: a survey and recommendations	2023	10.1109/ jcsse58229.2023.10 202122	IEEE	off topic	no
using imaging photoplethysmography (ippg) signal for blood pressure estimation	2020	10.1109/ mvip49855.2020.911 6902	IEEE	duplicate	no
using rear smartphone cameras as sensors for measuring heart rate variability	2021	10.1109/ access.2021.305406 5	IEEE	duplicate	no
validating a ppg device for estimating heart rate in various activity conditions	2024	10.1109/ esgco63003.2024.10 766973	IEEE	off topic	no
validation of heart rate estimation from photoplethysmograph and accelerometer recordings during post-exercise relaxation	2024	10.1109/ icetran62308.2024.1 0645151	IEEE	off topic	no
validation of heart rate monitoring using ppg sensor at dorsalis pedis artery	2024	10.1109/ rem63063.2024.107 35507	IEEE	off topic	no
var-hr: noncontact heart rate measurement using an rgb camera based on adaptive region selection with singular value decomposition	2024	10.1109/ lsens.2024.3375892	IEEE	duplicate	no
variational mode decomposition-based heart rate estimation using wrist-type photoplethysmography during physical exercise	2018	10.1109/ icpr.2018.8545685	IEEE	duplicate	no
variations of heart rate, pulse arrival time and blood pressure in a versatile laboratory protocol	2018	10.1109/ embc.2018.8512876	IEEE	duplicate	no

video based heart rate extraction using skin roi segmentation and attention cnn	2021	10.1109/ biocas49922.2021.9 645004	IEEE	off topic	no
video segment time-channel frequency attention network for remote photoplethysmography	2024	10.1109/ cvidl62147.2024.106 03505	IEEE	off topic	no
video-based heart rate measurement against uneven illuminations using multivariate singular spectrum analysis	2022	10.1109/ lsp.2022.3215112	IEEE	off topic	no
video-based heartbeat rate measuring method using ballistocardiography	2017	10.1109/ jsen.2017.2708133	IEEE	off topic	no
video-based measurement of physiological parameters using peak-to-valley method for minimization of initial dead zone	2018	10.1109/ icmlde.2018.00018	IEEE	off topic	no
video-based physiological measurement using 3d central difference convolution attention network	2021	10.1109/ ijcb52358.2021.9484 405	IEEE	duplicate	no
visual heart rate estimation from facial video based on cnn	2020	10.1109/ iciea48937.2020.924 8356	IEEE	off topic	no
wearable ear blood oxygen saturation and pulse measurement system based on ppg	2018	10.1109/ smartworld.2018.000 54	IEEE	duplicate	no
wearable multichannel photoplethysmography framework for heart rate monitoring during intensive exercise	2018	10.1109/ jsen.2018.2801385	IEEE	duplicate	no
wearable ppg sensor with bluetooth data transmission for continual measurement in low magnetic field environment	2021	10.23919/ ae51540.2021.9542 901	IEEE	duplicate	no
wearable visual-mimo for healthcare applications	2019	10.1109/ icasert.2019.893460 0	IEEE	off topic	no
where can exercisers sense heart rates accurately and comfortably on their bodies?	2019	10.1109/ ismict.2019.8743675	IEEE	duplicate	no
wi-fi based integrated system for the monitoring of heart rate and peripheral capillary oxygen saturation	2020	10.1109/ meco49872.2020.91 34347	IEEE	duplicate	no

wristband for bio-signal monitoring	2022	10.1109/ estc55720.2022.9939541	IEEE	duplicate	no
"but for the blind spot": accuracy and diagnostic performance of smart watch cardiac features in pediatric patients	2024	10.1016/ j.hrthm.2024.01.021	PUBMED	duplicate	no
2021 ishne/hrs/ehra/aphrs collaborative statement on mhealth in arrhythmia management: digital medical tools for heart rhythm professionals: from the international society for holter and noninvasive electrocardiology/heart rhythm society/european heart rhythm association/asia pacific heart rhythm society	2021	10.1016/ j.cvdhj.2020.11.004	PUBMED	off topic	no
a 172 μw compressively sampled photoplethysmographic (ppg) readout asic with heart rate estimation directly from compressively sampled data	2017	10.1109/ tbcas.2017.2661701	PUBMED	duplicate	no
a 2.3-5.7 μw tri-modal self-adaptive photoplethysmography sensor interface ic for heart rate, spo(2), and pulse transit time co-monitoring	2024	10.1109/ tbcas.2024.3360140	PUBMED	duplicate	no
a comparative study of accuracy in major adaptive filters for motion artifact removal in sleep apnea tests	2024	10.1007/s11517-023-02979-9	PUBMED	duplicate	no
a comparison of reflective photoplethysmography for detection of heart rate, blood oxygen saturation, and respiration rate at various anatomical locations	2019	10.3390/s19081874	PUBMED	duplicate	no
a comparison of wearable tonometry, photoplethysmography, and electrocardiography for cuffless measurement of blood pressure in an ambulatory setting	2022	10.1109/ jbhi.2022.3153259	PUBMED	duplicate	no
a comprehensive accuracy assessment of samsung smartwatch heart rate and heart rate variability	2022	10.1371/ journal.pone.0268361	PUBMED	duplicate	no
a correlational study on cardiopulmonary endurance in male patients with new-onset type 2 diabetes	2022	10.2147/ dmso.s352798	PUBMED	duplicate	no
a dual-channel ppg readout system with motion-tolerant adaptability for oled-opd sensors	2022	10.1109/ tbcas.2021.3138996	PUBMED	duplicate	no
a fast fpga hardware accelerator for remote heart rate detection based on rgb vision	2024	10.1109/ tbcas.2024.3354505	PUBMED	duplicate	no

a flexible and miniaturized chest patch for real-time ppg/ecg/bio-z monitoring	2022	10.1109/embc48229.2022.9872005	PUBMED	duplicate	no
a low-power photoplethysmogram-based heart rate sensor using heartbeat locked loop	2018	10.1109/tbcas.2018.2876671	PUBMED	duplicate	no
a motion-tolerant approach for monitoring spo(2) and heart rate using photoplethysmography signal with dual frame length processing and multi-classifier fusion	2017	10.1016/j.compbiomed.2017.10.017	PUBMED	duplicate	no
a multi-wavelength opto-electronic patch sensor to effectively detect physiological changes against human skin types	2017	10.3390/bios7020022	PUBMED	duplicate	no
a new approach to hr monitoring using photoplethysmographic signals during intensive physical exercise	2021	10.1007/s13246-021-01003-4	PUBMED	duplicate	no
a new principle of pulse detection based on terahertz wave plethysmography	2022	10.1038/s41598-022-09801-w	PUBMED	duplicate	no
a new wearable device for blood pressure estimation using photoplethysmogram	2019	10.3390/s19112557	PUBMED	duplicate	no
a novel and low-complexity peak detection algorithm for heart rate estimation from low-amplitude photoplethysmographic (ppg) signals	2018	10.1080/03091902.2019.1572237	PUBMED	duplicate	no
a novel chest-based ppg measurement system	2024	10.1109/jtehm.2024.3471468	PUBMED	duplicate	no
a novel clustering-based algorithm for continuous and noninvasive cuff-less blood pressure estimation	2022	10.1155/2022/3549238	PUBMED	duplicate	no
a novel diversity method for smartphone camera-based heart rhythm signals in the presence of motion and noise artifacts	2019	10.1371/journal.pone.0218248	PUBMED	duplicate	no
a novel signal restoration method of noisy photoplethysmograms for uninterrupted health monitoring	2023	10.3390/s24010141	PUBMED	duplicate	no
a phase ii, randomized, double-blind, double-dummy, active-controlled clinical trial to investigate the efficacy and safety of nw low-glu® in patients newly diagnosed with type 2 diabetes mellitus	2022	10.1155/2022/9176026	PUBMED	duplicate	no
a robust dynamic heart-rate detection algorithm framework during intense physical activities using photoplethysmographic signals	2017	10.3390/s17112450	PUBMED	duplicate	no

a robust motion artifact detection algorithm for accurate detection of heart rates from photoplethysmographic signals using time-frequency spectral features	2017	10.1109/jbhi.2016.2612059	PUBMED	duplicate	no
a robust random forest-based approach for heart rate monitoring using photoplethysmography signal contaminated by intense motion artifacts	2017	10.3390/s17020385	PUBMED	duplicate	no
a sliding scale signal quality metric of photoplethysmography applicable to measuring heart rate across clinical contexts with chest mounting as a case study	2023	10.3390/s23073429	PUBMED	duplicate	no
a smart mandibular advancement device for intraoral cardiorespiratory monitoring	2020	10.1109/embc44109.2020.9176520	PUBMED	duplicate	no
a solution for co-frequency and low snr problems in heart rate estimation based on photoplethysmography signals	2022	10.1007/s11517-022-02678-x	PUBMED	duplicate	no
a sparse sampling sensor front-end ic for low power continuous spo (2) & hr monitoring	2022	10.1109/tbcas.2022.3223971	PUBMED	duplicate	no
a study on the effect of contact pressure during physical activity on photoplethysmographic heart rate measurements	2020	10.3390/s20185052	PUBMED	duplicate	no
a teenager physical fitness evaluation model based on 1d-cnn with lstm and wearable running ppg recordings	2022	10.3390/bios12040202	PUBMED	duplicate	no
a wireless wearable sensor patch for the real-time estimation of continuous beat-to-beat blood pressure	2019	10.1109/embc.2019.8857446	PUBMED	duplicate	no
accuracy of a wrist-worn heart rate sensing device during elective pediatric surgical procedures	2018	10.3390/children5030038	PUBMED	duplicate	no
accuracy of heart rate measurement by the fitbit charge 2 during wheelchair activities in people with spinal cord injury: instrument validation study	2022	10.2196/27637	PUBMED	duplicate	no
accuracy of heart-rate-recovery parameters assessed from a wrist-worn photoplethysmography monitor (polar unite)	2023	10.1123/ijspp.2023-0120	PUBMED	duplicate	no
accuracy of optical heart rate sensing technology in wearable fitness trackers for young and older adults: validation and comparison study	2020	10.2196/14707	PUBMED	duplicate	no

accuracy of the wearable activity tracker garmin forerunner 235 for the assessment of heart rate during rest and activity	2019	10.1080/02640414.2018.1535563	PUBMED	duplicate	no
accuracy of wearable devices for measuring heart rate during conventional and nordic walking	2021	10.1002/pmrj.12424	PUBMED	duplicate	no
accurate heart rate monitoring during physical exercises using ppg	2017	10.1109/tbme.2017.2676243	PUBMED	duplicate	no
acute effects of breakfast fruits meal sequence and postprandial exercise on the blood glucose level and dpp4 activity among type 2 diabetes mellitus patients: a pilot study	2022	10.1155/2022/4875993	PUBMED	off topic	no
adapting smartphone-based photoplethysmograpy to suboptimal scenarios	2017	10.1088/1361-6579/aa51db	PUBMED	duplicate	no
adaptive scheduling of acceleration and gyroscope for motion artifact cancelation in photoplethysmography	2022	10.1016/j.cmpb.2022.107126	PUBMED	duplicate	no
affective video recommender systems: a survey	2022	10.3389/fnins.2022.984404	PUBMED	duplicate	no
an adaptive filter based motion artifact cancellation technique using multi-wavelength ppg for accurate hr estimation	2023	10.1109/tbcas.2023.3315297	PUBMED	duplicate	no
an effective photoplethysmography heart rate estimation framework integrating two-level denoising method and heart rate tracking algorithm guided by finite state machine	2022	10.1109/jbhi.2022.3165071	PUBMED	duplicate	no
an ml-based approach to reconstruct heart rate from ppg in presence of motion artifacts	2023	10.3390/bios13070718	PUBMED	duplicate	no
analysis of photoplethysmogram signal to estimate heart rate during physical activity using fractional fourier transform - a sampling frequency independent and reference signal-less method	2023	10.1016/j.cmpb.2022.107294	PUBMED	duplicate	no
and-rppg: a novel denoising-rppg network for improving remote heart rate estimation	2022	10.1016/j.compbiomed.2021.105146	PUBMED	duplicate	no
association between coronary artery atherosclerosis and plasma glucose levels assessed by dual-source computed tomography	2018	10.21037/jtd.2018.10.62	PUBMED	duplicate	no

association of baseline optical coherence tomography angiography with the development of glaucomatous visual field defects in preperimetric glaucoma eyes	2023	10.1136/bjo-2021-321025	PUBMED	duplicate	no
automated heart rate detection in seismocardiograms using electrocardiogram-based algorithms-a feasibility study	2024	10.3390/bioengineering11060596	PUBMED	duplicate	no
automatic heart rate detection during sleep using tracheal audio recordings from wireless acoustic sensor	2023	10.3390/diagnostics13182914	PUBMED	duplicate	no
binary cornet: accelerator for hr estimation from wrist-ppg	2020	10.1109/tbcas.2020.3001675	PUBMED	duplicate	no
blending human and machine: feasibility of measuring fatigue through the aviation headset	2020	10.1177/0018720819849783	PUBMED	duplicate	no
boosting algorithms based cuff-less blood pressure estimation from clinically relevant ecg and ppg morphological features	2023	10.1109/embc40787.2023.10340405	PUBMED	duplicate	no
bovine seminal plasma osteopontin: structural modelling, recombinant expression and its relationship with semen quality	2021	10.1111/and.13905	PUBMED	off topic	no
brazilian açaí berry seeds: an abundant waste applied in the synthesis of carbon-based acid catalysts for transesterification of low free fatty acid waste cooking oil	2021	10.1007/s11356-020-12054-7	PUBMED	off topic	no
brno university of technology smartphone ppg database (but ppg): annotated dataset for ppg quality assessment and heart rate estimation	2021	10.1155/2021/3453007	PUBMED	duplicate	no
camera-based cardiovascular screening based on heart rate and its variability in pre- and post-exercise conditions	2023	10.1109/embc40787.2023.10340871	PUBMED	duplicate	no
can smartwatch prevent sudden cardiac deaths? a case of smartwatch failure in arrhythmogenic right ventricular dysplasia	2021	10.7759/cureus.15904	PUBMED	off topic	no
changes in maternal heart rate variability and photoplethysmography morphology after corticosteroid administration: a prospective, observational study	2024	10.3390/jcm13082442	PUBMED	duplicate	no
characterization of quadratic nonlinearity between motion artifact and acceleration data and its application to heartbeat rate estimation	2017	10.3390/s17081872	PUBMED	duplicate	no

characterization study of neck photoplethysmography	2018	10.1109/ embc.2018.8513247	PUBMED	duplicate	no
chest-based real-time pulse and respiration monitoring based on bio-impedance	2020	10.1109/ embc44109.2020.91 76348	PUBMED	duplicate	no
classifying nocturnal blood pressure patterns using photoplethysmogram features	2022	10.1109/ embc48229.2022.98 71099	PUBMED	duplicate	no
clinical applications of contactless photoplethysmography for vital signs monitoring in pediatrics: a systematic review and meta-analysis	2023	10.1017/ cts.2023.557	PUBMED	duplicate	no
clinical validation of heart rate apps: mixed-methods evaluation study	2017	10.2196/ mhealth.7254	PUBMED	duplicate	no
consumer-led screening for atrial fibrillation: a report from the mafa-ii trial long-term extension cohort	2022	10.1016/ j.jacasi.2022.07.006	PUBMED	off topic	no
continuous heart rhythm monitoring using mobile photoplethysmography in ambulatory patients	2020	10.1016/ j.jelectrocard.2020.0 4.017	PUBMED	duplicate	no
continuous maternal hemodynamics monitoring at delivery using a novel, noninvasive, wireless,ppg-based sensor	2020	10.3390/ jcm10010008	PUBMED	duplicate	no
cornet: deep learning framework for ppg-based heart rate estimation and biometric identification in ambulant environment	2019	10.1109/ tbcas.2019.2892297	PUBMED	duplicate	no
d-dimer predicts long-term cause-specific mortality, cardiovascular events, and cancer in patients with stable coronary heart disease: lipid study	2018	10.1161/ circulationaha.117.0 29901	PUBMED	off topic	no
deeppulse: an uncertainty-aware deep neural network for heart rate estimations from wrist-worn photoplethysmography	2022	10.1109/ embc48229.2022.98 71813	PUBMED	duplicate	no
design and development of a wristband for continuous vital signs monitoring of covid-19 patients	2021	10.1109/ embc46164.2021.96 30299	PUBMED	duplicate	no
detection of cardiovascular disease based on ppg signals using machine learning with cloud computing	2022	10.1155/2022/16726 77	PUBMED	duplicate	no
detection of heart rate using smartphone gyroscope data: a scoping review	2023	10.3389/ fcvm.2023.1329290	PUBMED	duplicate	no

determination of saturation, heart rate, and respiratory rate at forearm using a nellcor™ forehead spo(2)-saturation sensor	2017	10.1007/s10877-016-9940-7	PUBMED	duplicate	no
development and clinical application of a novel autonomic transient response-based screening system for major depressive disorder using a fingertip photoplethysmographic sensor	2018	10.3389/fbioe.2018.00064	PUBMED	duplicate	no
disease pharmacokinetic-pharmacodynamic modelling in acute intermittent porphyria to support the development of mrna-based therapies	2020	10.1111/bph.15040	PUBMED	off topic	no
do photopletysmographic parameters of arterial stiffness differ depending on the presence of arterial hypertension and/or atherosclerosis?	2024	10.3390/s24144572	PUBMED	off topic	no
dual wavelength photoplethysmography framework for heart rate calculation	2022	10.3390/s22249955	PUBMED	duplicate	no
early physiologic numerical and waveform characteristics of simulated hemorrhagic events with healthy volunteers donating blood	2024	10.1097/cce.0000000000001073	PUBMED	duplicate	no
edge-enabled heart rate estimation from multisensor ppg signals	2023	10.1155/2023/4682760	PUBMED	duplicate	no
effect of ambient lighting and skin tone on estimation of heart rate and pulse transit time from video plethysmography	2020	10.1109/embc44109.2020.9176731	PUBMED	duplicate	no
effect of increasing heart rate on finger photoplethysmography fitness index (ppgf) in subjects with implanted cardiac pacemakers	2018	10.1371/journal.pone.0207301	PUBMED	duplicate	no
effect of low-dose mulberry fruit extract on postprandial glucose and insulin responses: a randomized pilot trial in individuals with type 2 diabetes	2024	10.3390/nu16142177	PUBMED	off topic	no
effect of position and fastening belt pressure on the accuracy of ppg-based heart rate sensor	2018	10.1109/embc.2018.8513333	PUBMED	duplicate	no
effect of urban environment on cardiovascular health: a feasibility pilot study using machine learning to predict heart rate variability in patients with heart failure	2024	10.1093/ehjdh/ztae050	PUBMED	off topic	no
effects of lifestyle intervention on plasma trimethylamine n-oxide in obese adults	2019	10.3390/nu11010179	PUBMED	duplicate	no
effects of transcutaneous trigeminal electrical stimulation and sound therapy in patients with tinnitus	2023	10.3349/ymj.2022.0611	PUBMED	off topic	no

efficacy and safety of a fixed-dose combination of vildagliptin and pioglitazone in indian patients with type 2 diabetes mellitus: a randomized, open-label, comparative, phase iii study	2023	10.7759/ cureus.44548	PUBMED	off topic	no
efficacy and safety profile of sitagliptin, vildagliptin, and metformin in newly diagnosed type 2 diabetic subjects	2021	10.1111/1440-1681.13561	PUBMED	duplicate	no
empowering people with a user-friendly wearable platform for unobtrusive monitoring of vital physiological parameters	2022	10.3390/s22145226	PUBMED	duplicate	no
enhance heart rate measurement from remote ppg with head motion awareness from image	2024	10.1109/ embc53108.2024.10782369	PUBMED	duplicate	no
enhanced contactless heart rate monitoring using camera with motion artifact removal during physical activities	2023	10.1109/ embc40787.2023.10340279	PUBMED	duplicate	no
ensemble empirical mode decomposition with principal component analysis: a novel approach for extracting respiratory rate and heart rate from photoplethysmographic signal	2018	10.1109/ jbhi.2017.2679108	PUBMED	duplicate	no
estimating heart rate and respiratory rate from a single lead electrocardiogram using ensemble empirical mode decomposition and spectral data fusion	2021	10.3390/s21041184	PUBMED	duplicate	no
estimation and validation of arterial blood pressure using photoplethysmogram morphology features in conjunction with pulse arrival time in large open databases	2021	10.1109/ jbhi.2020.3009658	PUBMED	duplicate	no
estimation of heart rate variability parameters by machine learning approaches applied to facial infrared thermal imaging	2022	10.3389/ fcvm.2022.893374	PUBMED	duplicate	no
evaluation of autonomic nervous system, saliva cortisol levels, and cognitive function in major depressive disorder patients	2018	10.1155/2018/7343592	PUBMED	duplicate	no
event-related driver stress detection with smartphones among young novice drivers	2022	10.1080/00140139.2021.2020342	PUBMED	duplicate	no
exploring the technological dimension of autonomous sensory meridian response-induced physiological responses	2024	10.7717/peerj.17754	PUBMED	duplicate	no

external temperature sensor assisted a new low power photoplethysmography readout system for accurate measurement of the bio-signs	2021	10.1007/s00542-020-05106-y	PUBMED	duplicate	no
facial remote photoplethysmography for continuous heart rate monitoring during prolonged cold liquid bolus administration	2024	10.1109/embc53108.2024.10781709	PUBMED	duplicate	no
feasibility of a wrist-worn wearable device for estimating mental health status in patients with mental illness	2023	10.3389/fpsyt.2023.1189765	PUBMED	duplicate	no
feasibility of electrodermal activity and photoplethysmography data acquisition at the foot using a sock form factor	2023	10.3390/s23020620	PUBMED	duplicate	no
feasibility study of deep neural network for heart rate estimation from wearable photoplethysmography and acceleration signals	2019	10.1109/embc.2019.8857618	PUBMED	duplicate	no
feasible assessment of recovery and cardiovascular health: accuracy of nocturnal hr and hrv assessed via ring ppg in comparison to medical grade ecg	2020	10.1088/1361-6579/ab840a	PUBMED	duplicate	no
finite state machine framework for instantaneous heart rate validation using wearable photoplethysmography during intensive exercise	2019	10.1109/jbhi.2018.2871177	PUBMED	duplicate	no
frequency domain analysis of photoplethysmographic and arterial pressure waveforms for assessing hemodynamics in children with congenital heart surgery	2024	10.4097/kja.23433	PUBMED	off topic	no
from lab to life: evaluating the reliability and validity of psychophysiological data from wearable devices in laboratory and ambulatory settings	2024	10.3758/s13428-024-02387-3	PUBMED	duplicate	no
fusion method to estimate heart rate from facial videos based on rppg and rbcg	2021	10.3390/s21206764	PUBMED	duplicate	no
head hemodynamics and systemic responses during auditory stimulation	2022	10.14814/phy2.15372	PUBMED	duplicate	no
heart rate detection using microsoft kinect: validation and comparison to wearable devices	2017	10.3390/s17081776	PUBMED	duplicate	no
heart rate estimation from ballistocardiogram signals processing via low-cost telemedicine architectures: a comparative performance evaluation	2023	10.3389/fdgth.2023.1222898	PUBMED	duplicate	no

heart rate estimation from neck photoplethysmography using fft-based scoring and a shallow neural network	2024	10.1109/embc53108.2024.10781693	PUBMED	duplicate	no
heart rate estimation using on-nail wearable photoplethysmography	2024	10.1109/embc53108.2024.10782437	PUBMED	duplicate	no
heart rate extraction from novel neck photoplethysmography signals	2019	10.1109/embc.2019.8857415	PUBMED	duplicate	no
heart rate imputation using accelerometers for wearable devices	2024	10.1109/embc53108.2024.10781795	PUBMED	duplicate	no
heart rate monitoring using sparse spectral curve tracing	2020	10.1109/embc44109.2020.9175349	PUBMED	duplicate	no
heart rate variability and heart rate monitoring of nurses using ppg and ecg signals during working condition: a pilot study	2022	10.1002/hsr2.477	PUBMED	duplicate	no
hepatic venous pressure gradient and rebleeding risk of patients with nonalcoholic steatohepatitis cirrhosis after variceal bleeding	2023	10.3389/fmed.2023.1224506	PUBMED	duplicate	no
highly wearable cuff-less blood pressure and heart rate monitoring with single-arm electrocardiogram and photoplethysmogram signals	2017	10.1186/s12938-017-0317-z	PUBMED	duplicate	no
identification of ictal tachycardia in focal motor- and non-motor seizures by means of a wearable ppg sensor	2021	10.3390/s21186017	PUBMED	duplicate	no
improved heart rate tracking using multiple wrist-type photoplethysmography during physical activities	2018	10.1109/embc.2018.8512736	PUBMED	duplicate	no
improvement of germinated brown rice quality with autoclaving treatment	2020	10.1002/fsn3.1459	PUBMED	duplicate	no
in-ear pulse rate measurement: a valid alternative to heart rate derived from electrocardiography?	2019	10.3390/s19173641	PUBMED	duplicate	no
incidence of multiple metachronous gastric cancers after pyloric-preserving gastrectomy	2020	10.1007/s00268-020-05492-4	PUBMED	duplicate	no
information retrieval from photoplethysmographic sensors: a comprehensive comparison of practical interpolation and breath-extraction techniques at different sampling rates	2022	10.3390/s22041428	PUBMED	duplicate	no

information-based similarity of ordinal pattern sequences as a novel descriptor in obstructive sleep apnea screening based on wearable photoplethysmography bracelets	2022	10.3390/bios12121089	PUBMED	duplicate	no
insights into vascular physiology from sleep photoplethysmography	2023	10.1093/sleep/zsad172	PUBMED	duplicate	no
integrated coronary disease burden and patterns to discriminate vessels benefiting from percutaneous coronary intervention	2022	10.1002/ccd.29983	PUBMED	duplicate	no
interface sensors with skin piezo-thermic transduction enable motion artifact removal for wearable physiological monitoring	2021	10.1016/j.bios.2021.113325	PUBMED	duplicate	no
investigation on pulse wave forward peak detection and its applications in cardiovascular health	2022	10.1109/tbme.2021.3103552	PUBMED	duplicate	no
is continuous heart rate monitoring of livestock a dream or is it realistic? a review	2020	10.3390/s20082291	PUBMED	duplicate	no
is heart rate a confounding factor for photoplethysmography markers? a systematic review	2020	10.3390/ijerph17072591	PUBMED	duplicate	no
kick ring ll: a multi-sensor ring capturing respiration, electrocardiogram, oxygen saturation, and skin temperature(1)	2020	10.1109/embc44109.2020.9176654	PUBMED	duplicate	no
learning based quality indicator aiding heart rate estimation in wrist-worn ppg	2021	10.1109/embc46164.2021.9630910	PUBMED	duplicate	no
long covid burden and risk factors in 10 uk longitudinal studies and electronic health records	2022	10.1038/s41467-022-30836-0	PUBMED	off topic	no
long-term effects of lowering postprandial glucose level on cardiovascular outcomes in early-stage diabetic patients with coronary artery disease: 10-year post-trial follow-up analysis of the diana study	2023	10.1016/j.jdiacomp.2023.108469	PUBMED	duplicate	no
low-frequency variability in photoplethysmographic waveform and heart rate during on-pump cardiac surgery with or without cardioplegia	2020	10.1038/s41598-020-58196-z	PUBMED	duplicate	no
lstm-based real-time signal quality assessment for blood volume pulse analysis	2023	10.1364/boe.477143	PUBMED	duplicate	no
lstm-only model for low-complexity hr estimation from wrist ppg	2021	10.1109/embc46164.2021.9630942	PUBMED	duplicate	no

measure by measure: resting heart rate across the 24-hour cycle	2023	10.1371/ journal.pdig.0000236	PUBMED	off topic	no
measurement and identification of mental workload during simulated computer tasks with multimodal methods and machine learning	2020	10.1080/00140139.2 020.1759699	PUBMED	duplicate	no
measurement of heart rate using the withings scanwatch device during free-living activities: validation study	2022	10.2196/34280	PUBMED	duplicate	no
measuring heart rate variability using commercially available devices in healthy children: a validity and reliability study	2020	10.3390/ ejihpe10010029	PUBMED	duplicate	no
methods for analyzing infant heart rate variability: a preliminary study	2023	10.1002/bdr2.2177	PUBMED	duplicate	no
mismatch repair deficiency, chemotherapy and survival for resectable gastric cancer: an observational study from the german star cohort and a meta-analysis	2023	10.1007/s00432-022-03953-y	PUBMED	off topic	no
modulation model of the photoplethysmography signal for vital sign extraction	2021	10.1109/ jbhi.2020.3013811	PUBMED	duplicate	no
mombat: heart rate monitoring from face video using pulse modeling and bayesian tracking	2020	10.1016/ j.compbiomed.2020. 103813	PUBMED	duplicate	no
monitoring of heart rate and inter-beat intervals with wrist plethysmography in patients with atrial fibrillation	2018	10.1088/1361-6579/aac9a9	PUBMED	duplicate	no
mortality after paclitaxel coated balloon angioplasty and stenting of superficial femoral and popliteal artery in the vascular quality initiative	2020	10.1161/ circinterventions.119 .008528	PUBMED	off topic	no
motion artifact canceling ppg heart rate sensor based on an adaptive filter algorithm with variable tap length	2020	10.1109/ embc44109.2020.91 76715	PUBMED	duplicate	no
motion artifact reduction for wrist-worn photoplethysmograph sensors based on different wavelengths	2019	10.3390/s19030673	PUBMED	duplicate	no
motion artifact reduction in wearable photoplethysmography based on multi-channel sensors with multiple wavelengths	2020	10.3390/s20051493	PUBMED	duplicate	no

motion artifact removal for ppg signals based on accurate fundamental frequency estimation and notch filtering	2018	10.1109/embc.2018.8512878	PUBMED	duplicate	no
multi-mode particle filtering methods for heart rate estimation from wearable photoplethysmography	2019	10.1109/tbme.2019.2895685	PUBMED	duplicate	no
multi-sensor wearable health device framework for real-time monitoring of elderly patients using a mobile application and high-resolution parameter estimation	2022	10.3389/fnhum.2021.750591	PUBMED	duplicate	no
nocturnal pulse wave amplitude attenuations are associated with long-term cardiovascular events	2023	10.1016/j.ijcard.2023.05.047	PUBMED	duplicate	no
noise-robust heart rate estimation algorithm from photoplethysmography signal with low computational complexity	2019	10.1155/2019/6283279	PUBMED	duplicate	no
non-contact heart rate and blood pressure estimations from video analysis and machine learning modelling applied to food sensory responses: a case study for chocolate	2018	10.3390/s18061802	PUBMED	duplicate	no
non-contact heart rate monitoring in neonatal intensive care unit using rgb camera	2020	10.1109/embc44109.2020.9175651	PUBMED	duplicate	no
non-invasive methods for heart rate measurement in fish based on photoplethysmography	2024	10.1242/jeb.246464	PUBMED	animal	no
non-invasive pulse arrival time is associated with cardiac index in pediatric heart transplant patients with normal ejection fraction	2024	10.1088/1361-6579/ad61b9	PUBMED	duplicate	no
noninvasive blood oxygen, heartbeat rate, and blood pressure parameter monitoring by photoplethysmography signals	2022	10.1016/j.heliyon.2022.e11698	PUBMED	duplicate	no
novel tailoring algorithm for abrupt motion artifact removal in photoplethysmogram signals	2017	10.1007/s13534-017-0037-0	PUBMED	duplicate	no
optimization of bioactive phenolics extraction and cosmeceutical activity of eco-friendly polypropylene-glycol-lactic-acid-based extracts of olive leaf	2022	10.3390/molecules27020529	PUBMED	off topic	no
parhelia: particle filter-based heart rate estimation from photoplethysmographic signals during physical exercise	2018	10.1109/tbme.2017.2697911	PUBMED	duplicate	no

pathological and oncological outcomes of pylorus-preserving versus conventional distal gastrectomy in early gastric cancer: a systematic review and meta-analysis	2022	10.1186/s12957-022-02766-0	PUBMED	duplicate	no
pbs qd-coated si micro-hole array/graphene vdw schottky near-infrared photodiode for ppg heart rate measurement	2023	10.3390/s23167214	PUBMED	duplicate	no
personalized stress monitoring using wearable sensors in everyday settings	2021	10.1109/embc46164.2021.9630224	PUBMED	duplicate	no
photobiomodulation improves cell survival and death parameters in cardiomyocytes exposed to hypoxia/reoxygenation	2024	10.1016/j.jphotobiol.2024.112991	PUBMED	off topic	no
photoplethysmographic waveform analysis for autonomic reactivity assessment in depression	2021	10.1109/tbme.2020.3025908	PUBMED	duplicate	no
photoplethysmography wave morphology in patients with atrial fibrillation	2023	10.1088/1361-6579/acc725	PUBMED	duplicate	no
pilot study assessing the influence of skin type on the heart rate measurements obtained by photoplethysmography with the apple watch	2019	10.1007/s10916-019-1325-2	PUBMED	duplicate	no
postprandial plasma glucose measured from blood taken between 4 and 7.9 h is positively associated with mortality from hypertension and cardiovascular disease	2024	10.3390/jcdd11020053	PUBMED	off topic	no
power spectral densities of nocturnal pulse oximetry signals differ in osa patients with and without daytime sleepiness	2020	10.1016/j.sleep.2020.07.015	PUBMED	duplicate	no
ppg neurons in the nucleus of the solitary tract modulate heart rate but do not mediate glp-1 receptor agonist-induced tachycardia in mice	2020	10.1016/j.molmet.2020.101024	PUBMED	duplicate	no
ppgnet: deep network for device independent heart rate estimation from photoplethysmogram	2019	10.1109/embc.2019.8856989	PUBMED	duplicate	no
precision heart rate estimation using a ppg sensor patch equipped with new algorithms of pre-quality checking and hankel decomposition	2023	10.3390/s23136180	PUBMED	duplicate	no
process design for optimized respiration identification based on heart rate variability for efficient respiratory sinus arrhythmia biofeedback	2022	10.3390/ijerph19042087	PUBMED	duplicate	no

profiling the propagation of error from ppg to hrv features in a wearable physiological-monitoring device	2018	10.1049/ htl.2017.0039	PUBMED	duplicate	no
prognostic implications of prestent pullback pressure gradient and poststent quantitative flow ratio in patients undergoing percutaneous coronary intervention	2022	10.1161/ jaha.121.024903	PUBMED	duplicate	no
proinflammatory cytokines predict the incidence of diabetic peripheral neuropathy over 5 years in chinese type 2 diabetes patients: a prospective cohort study	2020	10.1016/ j.eclinm.2020.10064 9	PUBMED	off topic	no
propensity-score matched analysis of three years survival of trans carotid artery revascularization versus carotid endarterectomy in the vascular quality initiative medicare-linked database	2023	10.1097/ sla.00000000000060 09	PUBMED	off topic	no
q-ppg: energy-efficient ppg-based heart rate monitoring on wearable devices	2021	10.1109/ tbcas.2021.3122017	PUBMED	duplicate	no
quantifying the accuracy of inter-beat intervals acquired from consumer-grade photoplethysmography wristbands using an electrocardiogram-aided information-based similarity approach	2024	10.1088/1361- 6579/ad2c14	PUBMED	duplicate	no
quantitative flow ratio as a continuous predictor of myocardial infarction	2023	10.4244/eij-d-23- 00026	PUBMED	off topic	no
randomized noninferiority trial of radiation exposure during coronary angiography: the transradial and transfemoral approach by experienced operators in daily routine (expert) trial	2023	10.14503/thij-22- 7930	PUBMED	off topic	no
rapid vital sign extraction for real-time opto-physiological monitoring at varying physical activity intensity levels	2023	10.1109/ jbhi.2023.3268240	PUBMED	duplicate	no
real-time quality index to control data loss in real-life cardiac monitoring applications	2021	10.3390/s21165357	PUBMED	duplicate	no
real-time robust heart rate estimation from wrist-type ppg signals using multiple reference adaptive noise cancellation	2018	10.1109/ jbhi.2016.2632201	PUBMED	duplicate	no
real-world heart rate norms in the health eheart study	2019	10.1038/s41746- 019-0134-9	PUBMED	duplicate	no
recommendations for determining the validity of consumer wearable heart rate devices: expert statement and checklist of the interlive network	2021	10.1136/ bjsports-2020- 103148	PUBMED	duplicate	no

reference signal less fourier analysis based motion artifact removal algorithm for wearable photoplethysmography devices to estimate heart rate during physical exercises	2022	10.1016/j.compbiomed.2021.105081	PUBMED	duplicate	no
remote monitoring of atrial fibrillation recurrence using mhealth technology (remote-af)	2024	10.1093/ehjdh/ztae011	PUBMED	duplicate	no
remote photoplethysmography and heart rate estimation by dynamic region of interest tracking	2022	10.1109/embc48229.2022.9871722	PUBMED	duplicate	no
removal of motion artifacts in photoplethysmograph sensors during intensive exercise for accurate heart rate calculation based on frequency estimation and notch filtering	2019	10.3390/s19153312	PUBMED	duplicate	no
resting and postexercise heart rate detection from fingertip and facial photoplethysmography using a smartphone camera: a validation study	2017	10.2196/mhealth.7275	PUBMED	duplicate	no
risk factors of poor prognosis in patients with pyrrolidine alkaloid-induced hepatic sinusoidal obstruction syndrome after transjugular intrahepatic portosystemic shunt	2021	10.1007/s12072-020-10126-x	PUBMED	duplicate	no
robust heart rate detection via multi-site photoplethysmography	2024	10.1109/embc53108.2024.10782155	PUBMED	duplicate	no
robust heart rate estimation during physical exercise using photoplethysmographic signals	2018	10.1109/embc.2018.8512405	PUBMED	duplicate	no
robust heart rate estimation using wrist-type photoplethysmographic signals during physical exercise: an approach based on adaptive filtering	2017	10.1088/1361-6579/aa506e	PUBMED	duplicate	no
robust heart rate monitoring for quasi-periodic motions by wrist-type ppg signals	2020	10.1109/jbhi.2019.2912708	PUBMED	duplicate	no
robust motion artefact resistant circuit for calculation of mean arterial pressure from pulse transit time	2017	10.1109/embc.2017.8037624	PUBMED	duplicate	no
robust ppg-based ambulatory heart rate tracking algorithm	2020	10.1109/embc44109.2020.9175346	PUBMED	duplicate	no
signal quality index based on template cross-correlation in multimodal biosignal chair for smart healthcare	2021	10.3390/s21227564	PUBMED	duplicate	no

sleep apnea screening based on photoplethysmography data from wearable bracelets using an information-based similarity approach	2021	10.1016/ j.cmpb.2021.106442	PUBMED	duplicate	no
sleep/wake classification via remote ppg signals	2019	10.1109/ embc.2019.8857097	PUBMED	duplicate	no
smart garment fabrics to enable non-contact opto-physiological monitoring	2018	10.3390/ bios8020033	PUBMED	duplicate	no
smart mandibular advancement device for intraoral monitoring of cardiorespiratory parameters and sleeping postures	2021	10.1109/ tbcas.2021.3065824	PUBMED	duplicate	no
smartphone-based photoplethysmographic imaging for heart rate monitoring	2017	10.1080/03091902.2 017.1299233	PUBMED	duplicate	no
smartwatch ppg peak detection method for sinus rhythm and cardiac arrhythmia	2019	10.1109/ embc.2019.8857325	PUBMED	duplicate	no
sources of inaccuracy in photoplethysmography for continuous cardiovascular monitoring	2021	10.3390/ bios11040126	PUBMED	duplicate	no
spatiotemporal distribution of individuals as an indicator for the social system of lepilemur sahamalaza	2019	10.1002/ajp.22984	PUBMED	off topic	no
spot measurement of heart rate based on morphology of photoplethysmographic (ppg) signals	2017	10.1080/03091902.2 016.1223198	PUBMED	duplicate	no
state-dependent gaussian kernel-based power spectrum modification for accurate instantaneous heart rate estimation	2019	10.1371/ journal.pone.021501 4	PUBMED	duplicate	no
targeted decrease of portal hepatic pressure gradient improves ascites control after tips	2023	10.1002/hep.32676	PUBMED	duplicate	no
task-evoked pulse wave amplitude tracks cognitive load	2023	10.1038/s41598-023-48917-5	PUBMED	duplicate	no
the accuracy of heartbeat detection using photoplethysmography technology in cardiac patients	2021	10.1016/ j.jelectrocard.2021.0 6.009	PUBMED	duplicate	no
the accuracy of wrist-worn photoplethysmogram-measured heart and respiratory rates in abdominal surgery patients: observational prospective clinical validation study	2023	10.2196/40474	PUBMED	duplicate	no
the dutch car-t tumorboard experience: population-based real-world data on patients with relapsed or refractory large b-cell lymphoma referred for cd19-directed car t-cell therapy in the netherlands	2023	10.3390/ cancers15174334	PUBMED	off topic	no

the essential oil of hyptis mutabilis in ichthyophthirius multifiliis infection and its effect on hematological, biochemical, and immunological parameters in silver catfish, rhamdia quelen	2017	10.1645/16-174	PUBMED	off topic	no
the impact of healthy pregnancy on features of heart rate variability and pulse wave morphology derived from wrist-worn photoplethysmography	2023	10.1038/s41598-023-47980-2	PUBMED	duplicate	no
time-varying assessment of heart rate variability parameters using respiratory information	2017	10.1016/j.compbiomed.2017.07.022	PUBMED	duplicate	no
towards a machine learning-based digital twin for non-invasive human bio-signal fusion	2022	10.3390/s22249747	PUBMED	duplicate	no
towards quantifying stress in patients with a history of myocardial infarction: validating ecg-derived patch features	2023	10.1109/embc40787.2023.10340614	PUBMED	duplicate	no
transjugular intrahepatic portosystemic shunt placement in patients with schistosomiasis-induced liver fibrosis	2019	10.1007/s00270-019-02295-6	PUBMED	duplicate	no
trends in heart rate and heart rate variability during pregnancy and the 3-month postpartum period: continuous monitoring in a free-living context	2022	10.2196/33458	PUBMED	duplicate	no
unobtrusive photoplethysmographic monitoring under the foot sole while in a standing posture	2018	10.3390/s18103239	PUBMED	duplicate	no
use of a smart watch for early detection of paroxysmal atrial fibrillation: validation study	2020	10.2196/14857	PUBMED	duplicate	no
use of heart rate variability and photoplethysmograph-derived parameters as assessment signals of radiofrequency therapy efficacy for chronic pain	2017	10.1111/papr.12536	PUBMED	duplicate	no
utility of photoplethysmography for heart rate estimation among inpatients	2018	10.1111/imj.13777	PUBMED	duplicate	no
validation of heart rate extracted from wrist-based photoplethysmography in the perioperative setting: prospective observational study	2021	10.2196/27765	PUBMED	duplicate	no
validity of resting heart rate derived from contact-based smartphone photoplethysmography compared with electrocardiography: a scoping review and checklist for optimal acquisition and reporting	2024	10.3389/fdgth.2024.1326511	PUBMED	duplicate	no

validity of the polar vantage m watch when measuring heart rate at different exercise intensities	2021	10.7717/peerj.10893	PUBMED	duplicate	no
validity of the wrist-worn polar vantage v2 to measure heart rate and heart rate variability at rest	2021	10.3390/s22010137	PUBMED	duplicate	no
variations of heart rate, pulse arrival time and blood pressure in a versatile laboratory protocol	2018	10.1109/embc.2018.8512876	PUBMED	duplicate	no
video-based heart rate monitoring across a range of skin pigmentations during an acute hypoxic challenge	2018	10.1007/s10877-017-0076-1	PUBMED	off topic	no
video-based pulse rate variability measurement using periodic variance maximization and adaptive two-window peak detection	2020	10.3390/s20102752	PUBMED	duplicate	no
wearable multisensor ring-shaped probe for assessing stress and blood oxygenation: design and preliminary measurements	2023	10.3390/bios13040460	PUBMED	duplicate	no
wearable ring-shaped biomedical device for physiological monitoring through finger-based acquisition of electrocardiographic, photoplethysmographic, and galvanic skin response signals: design and preliminary measurements	2024	10.3390/bios14040205	PUBMED	duplicate	no
wearable vital signs monitoring for patients with asthma: a review	2020	10.1109/jsen.2022.3224411	PUBMED	off topic	no
wireless, non-invasive, wearable device for continuous remote monitoring of hemodynamic parameters in a swine model of controlled hemorrhagic shock	2020	10.1038/s41598-020-74686-6	PUBMED	duplicate	no
wrist ballistocardiography and invasively recorded blood pressure in healthy volunteers during reclining bike exercise	2023	10.3389/fphys.2023.1189732	PUBMED	duplicate	no
wrist-based photoplethysmography assessment of heart rate and heart rate variability: validation of whoop	2021	10.3390/s21103571	PUBMED	duplicate	no
wrist-worn optical and chest strap heart rate comparison in a heterogeneous sample of healthy individuals and in coronary artery disease patients	2018	10.1186/s13102-018-0098-0	PUBMED	duplicate	no
your blush gives you away: detecting hidden mental states with remote photoplethysmography and thermal imaging	2024	10.7717/peerj-cs.1912	PUBMED	duplicate	no
“but for the blind spot”: accuracy and diagnostic performance of smart watch cardiac features in pediatric patients	2024	10.1016/j.hrthm.2024.01.021	SCOPUS	off topic	no

1-dimensional convolutional neural network based blood pressure estimation with photo plethysmography signals and semi-classical signal analysis	2022	10.37391/ ijeer.100228	SCOPUS	multichannel	no
17.8 a 2.6µw monolithic cmos photoplethysmographic sensor operating with 2µw led power	2019	10.1109/ isscc.2019.8662404	SCOPUS	hardware	no
a 0.5v ppg-based heart rate and variability detection system	2018	10.1109/ biocas.2018.858475 6	SCOPUS	hardware	no
a 1.8v 16µa 136.5db dr ppg/nirs recording ic using noise shaping triple slope light to digital converter	2023	10.1109/ cicc57935.2023.101 21288	SCOPUS	hardware	no
a 171µw ppg-based vitals monitoring soc for asthmatic patients	2022	10.1109/ biocas54905.2022.9 948619	SCOPUS	hardware	no
a 172 µw compressively sampled photoplethysmographic (ppg) readout asic with heart rate estimation directly from compressively sampled data	2017	10.1109/ tbcas.2017.2661701	SCOPUS	hardware	no
a 1mw vitals monitoring system for asthmatic patients based on photoplethysmography	2019	10.1109/ biocas.2019.891872 4	SCOPUS	hardware	no
a 2.3-5.7 µw tri-modal self-adaptive photoplethysmography sensor interface ic for heart rate, spo2, and pulse transit time co-monitoring	2024	10.1109/ tbcas.2024.3360140	SCOPUS	hardware	no
A 206 µW Vital Signs Monitoring System on Chip for Measuring Five Vitals	2024	10.1109/ tvlsi.2024.3415469	SCOPUS	hardware	no
a 28µw 134db dr 2nd-order noise-shaping slope light-to-digital converter for chest ppg monitoring	2021	10.1109/ isscc42613.2021.93 65757	SCOPUS	hardware	no
a 385µw photoplethysmography-based vitals monitoring soc with 110db current-to-digital converter	2022	10.1109/ iscas48785.2022.99 37795	SCOPUS	hardware	no
a 400-to-1000nm 24µ w monolithic ppg sensor with 0.3a/w spectral responsivity for miniature wearables	2021	10.1109/ isscc42613.2021.93 66035	SCOPUS	hardware	no
a 43.4µw photoplethysmogram-based heart-rate sensor using heart-beat-locked loop	2018	10.1109/ isscc.2018.8310390	SCOPUS	hardware	no

a camera-based pulse transit time estimation approach towards non-intrusive blood pressure monitoring	2019	10.1109/ ichi.2019.8904498	SCOPUS	off topic	no
a comparative evaluation of a wearable mems tactile sensors array and a photoplethysmography sensor for atrial fibrillation detection under sitting condition	2021	10.1109/ transducers50396.2 021.9495407	SCOPUS	off topic	no
a comparative study of accuracy in major adaptive filters for motion artifact removal in sleep apnea tests	2024	10.1007/s11517- 023-02979-9	SCOPUS	off topic	no
a comparison of reflective photoplethysmography for detection of heart rate, blood oxygen saturation, and respiration rate at various anatomical locations	2019	10.3390/s19081874	SCOPUS	off topic	no
a comparison of wearable tonometry, photoplethysmography, and electrocardiography for cuffless measurement of blood pressure in an ambulatory setting	2022	10.1109/ jbhi.2022.3153259	SCOPUS	off topic	no
a comprehensive accuracy assessment of samsung smartwatch heart rate and heart rate variability	2022	10.1371/ journal.pone.026836 1	SCOPUS	off topic	no
a correlational study on cardiopulmonary endurance in male patients with new-onset type 2 diabetes	2022	10.2147/ dmso.s352798	SCOPUS	off topic	no
a deep learning approach to estimate spo2 from ppg signals	2022	10.1145/3569192.35 69215	SCOPUS	off topic	no
a deep learning approach to estimate the respiratory rate from photoplethysmogram; [un enfoque de aprendizaje profundo para estimar la frecuencia respiratoria del fotopletismograma]	2022	10.17163/ ings.n27.2022.09	SCOPUS	off topic	no
a deep learning-based continuous blood pressure measurement by dual photoplethysmography signals	2022	10.32604/ cmc.2022.020493	SCOPUS	off topic	no
a deep learning-based ppg quality assessment approach for heart rate and heart rate variability	2023	10.1145/3616019	SCOPUS	inaccessible dataset	yes
a dual-channel ppg readout system with motion-tolerant adaptability for oled-opd sensors	2022	10.1109/ tbcas.2021.3138996	SCOPUS	multichannel	no
a dynamic reconfigurable wearable device to acquire high quality ppg signal and robust heart rate estimate based on deep learning algorithm for smart healthcare system	2022	10.1016/ j.biosx.2022.100223	SCOPUS	hardware	no
a fast fpga hardware accelerator for remote heart rate detection based on rgb vision	2024	10.1109/ tbcas.2024.3354505	SCOPUS	hardware	no

a flexible and miniaturized chest patch for real-time ppg/ecg/bio-z monitoring	2022	10.1109/embc48229.2022.9872005	SCOPUS	hardware	no
a frame work to estimate heart rate and arterial oxygen saturation (spo2)	2017	10.1109/iccsp.2017.8286669	SCOPUS	off topic	no
a headphone-based heart rate and heart rate variability monitoring unit	2023	10.1007/978-3-031-28725-1_13	SCOPUS	off topic	no
a high accuracy & ultra-low power ppg-derived hr estimation ai processor for wearable devices	2023	10.1109/icet58434.2023.10212060	SCOPUS	hardware	no
a high-efficiency and real-time method for quality evaluation of ppg signals	2020	10.1088/1757-899x/711/1/012100	SCOPUS	off topic	no
a low-complexity model-free approach for real-time cardiac anomaly detection based on singular spectrum analysis and nonparametric control charts	2018	10.3390/technologies6010026	SCOPUS	off topic	no
a low-cost methodology for developing personal health monitoring devices to examine psychological states and the impact of exercises	2023	10.1007/s41870-023-01473-7	SCOPUS	off topic	no
a low-power compressive sampling (cs) photoplethysmogram (ppg) readout with embedded feature extraction	2019	10.1007/978-3-030-05870-8_5	SCOPUS	off topic	no
a low-power heart rate sensor with adaptive heartbeat locked loop	2021	10.1109/iscas51556.2021.9401726	SCOPUS	inaccessible dataset	no
a low-power photoplethysmogram-based heart rate sensor using heartbeat locked loop	2018	10.1109/tbcas.2018.2876671	SCOPUS	hardware	no
a low-power ppg processor for real-time biometric identification and heart rate estimation	2023	10.1109/tcsii.2023.3291891	SCOPUS	hardware	no
a low-power predictive sampling ppg sensor	2024	10.1109/iscas58744.2024.10557982	SCOPUS	hardware	no
a lstm-based realtime signal quality assessment for photoplethysmogram and remote photoplethysmogram	2021	10.1109/cvprw53098.2021.00424	SCOPUS	off topic	no
a machine learning approach for heart rate estimation from ppg signal using random forest regression algorithm	2019	10.1109/ecace.2019.8679356	SCOPUS	off topic	no

a machine learning method to improve non-contact heart rate monitoring using an rgb camera	2018	10.1109/ access.2018.287275 6	SCOPUS	off topic	no
a machine learning-empowered system for long-term motion-tolerant wearable monitoring of blood pressure and heart rate with ear-ecg/ppg	2017	10.1109/ access.2017.270747 2	SCOPUS	multichannel	no
a motion heart-rate monitor watch with improved grey differential equation model based on reflective photoplethysmography	2017	10.1007/s40846-017-0241-5	SCOPUS	off topic	no
a motion robust remote-ppg approach to driver’s health state monitoring	2017	10.1007/978-3-319-54407-6_31	SCOPUS	off topic	no
a motion-tolerant approach for monitoring spo2 and heart rate using photoplethysmography signal with dual frame length processing and multi-classifier fusion	2017	10.1016/ j.compbiomed.2017. 10.017	SCOPUS	multichannel	no
a multi-wavelength opto-electronic patch sensor to effectively detect physiological changes against human skin types	2017	10.3390/ bios7020022	SCOPUS	off topic	no
a multiplexed electronic architecture for opto-electronic patch sensor to effectively monitor heart rate and oxygen saturation	2018	10.1117/12.2287833	SCOPUS	off topic	no
a new approach to hr monitoring using photoplethysmographic signals during intensive physical exercise	2021	10.1007/s13246-021-01003-4	SCOPUS	inaccessible dataset	no
a new intelligent approach for automatic stress level assessment based on multiple physiological parameters monitoring	2024	10.1109/ tim.2023.3342218	SCOPUS	off topic	no
a new low power photoplethysmography signal acquisition system for mental stress estimation	2021	10.1115/isps2021-65097	SCOPUS	off topic	no
a new principle of pulse detection based on terahertz wave plethysmography	2022	10.1038/s41598-022-09801-w	SCOPUS	off topic	no
a newwearable device for blood pressure estimation using photoplethysmogram	2019	10.3390/s19112557	SCOPUS	off topic	no
a non-contact heart rate estimation framework based on photoplethysmography amplitude variation elimination and data fusion	2021	10.1109/ icbme54433.2021.97 50345	SCOPUS	off topic	no
a non-invasive heart rate and blood pressure monitoring system using piezoelectric and photoplethysmographic sensors	2022	10.1016/ j.measurement.2022 .111211	SCOPUS	multichannel	no

a novel adaptive spectrum noise cancellation approach for enhancing heartbeat rate monitoring in a wearable device	2018	10.1109/access.2018.2805223	SCOPUS	denoising	no
a novel and low-complexity peak detection algorithm for heart rate estimation from low-amplitude photoplethysmographic (ppg) signals	2018	10.1080/03091902.2019.1572237	SCOPUS	not accessible	no
a novel approach framework based on statistics for reconstruction and heartrate estimation from ppg with heavy motion artifacts	2018	10.1007/s11432-017-9168-2	SCOPUS	INCLUDED	yes
a novel clustering-based algorithm for continuous and noninvasive cuff-less blood pressure estimation	2022	10.1155/2022/3549238	SCOPUS	off topic	no
a novel convolutional neural network deep learning implementation for cuffless heart rate and blood pressure estimation	2023	10.3390/app132212403	SCOPUS	off topic	no
a novel diversity method for smartphone camera-based heart rhythm signals in the presence of motion and noise artifacts	2019	10.1371/journal.pone.0218248	SCOPUS	off topic	no
a novel motion artifact removal method via joint basis pursuit linear program to accurately monitor heart rate	2019	10.1109/jsen.2019.2927994	SCOPUS	denoising	no
a novel signal restoration method of noisy photoplethysmograms for uninterrupted health monitoring	2024	10.3390/s24010141	SCOPUS	off topic	no
a novel strategy for signal reconstruction of noisy segments in photoplethysmographic recordings	2022	10.1109/ehb55594.2022.9991390	SCOPUS	denoising	no
a novel technique to compress photoplethysmogram signal: improvised with particle swarm optimization and rivest-shamir-adleman algorithm	2022	10.1109/calcon56258.2022.10060588	SCOPUS	off topic	no
a perturbation-injection-locked sensor with self-oscillating active csrr for vital-sign detection from fingertip	2019	10.1109/mwsym.2019.8700980	SCOPUS	off topic	no
a phase ii, randomized, double-blind, double-dummy, active-controlled clinical trial to investigate the efficacy and safety of nw low-glu® in patients newly diagnosed with type 2 diabetes mellitus	2022	10.1155/2022/9176026	SCOPUS	off topic	no
a practical approach to health status monitoring based on heart rate and respiratory rate assessment	2022	10.1109/memea54994.2022.9856576	SCOPUS	hardware	no

a preliminary investigation of the effect of contact pressure on the accuracy of heart rate monitoring by wearable ppg wrist band	2019	10.1109/metroi4.2019.8792834	SCOPUS	off topic	no
a random tree based algorithm for blood pressure estimation	2020	10.1109/imbioc47321.2020.9385038	SCOPUS	off topic	no
a real-time algorithm for ppg signal processing during intense physical activity	2020	10.1007/978-3-030-42029-1_2	SCOPUS	not accessible	no
a real-time software to the acquisition of heart rate and photoplethysmography signal using two region of interest simultaneously via webcam	2017	10.1109/urtc.2017.8284207	SCOPUS	off topic	no
a review of ppg/nirs acquisition asic and system	2021	10.1109/asicon52560.2021.9620430	SCOPUS	review	no
a review on video-based heart rate, respiratory rate and blood pressure estimation	2023	10.1007/978-3-031-29313-9_12	SCOPUS	off topic	no
a robust dynamic heart-rate detection algorithm framework during intense physical activities using photoplethysmographic signals	2017	10.3390/s17112450	SCOPUS	inaccessible dataset	no
a robust fusion method for motion artifacts reduction in photoplethysmography signal	2020	10.1109/tim.2020.3006636	SCOPUS	denoising	no
a robust motion artifact detection algorithm for accurate detection of heart rates from photoplethysmographic signals using time-frequency spectral features	2017	10.1109/jbhi.2016.2612059	SCOPUS	off topic	no
a robust random forest-based approach for heart rate monitoring using photoplethysmography signal contaminated by intense motion artifacts	2017	10.3390/s17020385	SCOPUS	inaccessible dataset	no
a sliding scale signal quality metric of photoplethysmography applicable to measuring heart rate across clinical contexts with chest mounting as a case study	2023	10.3390/s23073429	SCOPUS	off topic	no
a smart mandibular advancement device for intraoral cardiorespiratory monitoring	2020	10.1109/embc44109.2020.9176520	SCOPUS	off topic	no
a sock-embedded wireless sensing system employing paper-substrate-based mems tactile sensors array and low-power asic for accurate and comfortable afib monitoring	2023	10.1109/jsen.2023.3329100	SCOPUS	off topic	no

a solution for co-frequency and low snr problems in heart rate estimation based on photoplethysmography signals	2022	10.1007/s11517-022-02678-x	SCOPUS	multichannel	yes
a sparse sampling sensor front-end ic for low power continuous spo2& hr monitoring	2022	10.1109/tbcas.2022.3223971	SCOPUS	off topic	no
a study on the effect of contact pressure during physical activity on photoplethysmographic heart rate measurements	2020	10.3390/s20185052	SCOPUS	off topic	no
a subcutaneously injectable implant for multimodal physiological monitoring in animals	2024	10.1109/jsen.2024.3366195	SCOPUS	off topic	no
a survey on fatigue and drowsiness detection techniques in driving	2021	10.1109/icccis51004.2021.9397224	SCOPUS	off topic	no
a teenager physical fitness evaluation model based on 1d-cnn with lstm and wearable running ppg recordings	2022	10.3390/bios12040202	SCOPUS	off topic	no
a trellis-based peak frequency tracking technique for photoplethysmographic heart rate sensing during exercise	2020	10.1109/ismict48699.2020.9152718	SCOPUS	off topic	no
a wearable photoplethysmography sensor for non-invasive equine heart rate monitoring	2023	10.1016/j.atech.2023.100264	SCOPUS	animal	no
a wearable wireless medical sensor network system towards internet-of-patients	2018	10.1109/icsens.2018.8589642	SCOPUS	animal	no
a weiner filter based robust algorithm for estimation of heart rate from wrist based photoplethysmogram	2019	10.1145/3341162.3344837	SCOPUS	inaccessible dataset	no
a wireless low-power single-unit wearable system for continuous early warning score calculation	2023	10.1109/jsen.2023.3267146	SCOPUS	off topic	no
a wireless wearable sensor patch for the real-time estimation of continuous beat-to-beat blood pressure	2019	10.1109/embc.2019.8857446	SCOPUS	off topic	no
accuracy of a wrist-worn heart rate sensing device during elective pediatric surgical procedures	2018	10.3390/children5030038	SCOPUS	inaccessible dataset	no
accuracy of heart rate measured by military-grade wearable ecg monitor compared with reference and commercial monitors	2023	10.1136/military-2023-002541	SCOPUS	off topic	no
accuracy of heart rate measurement by the fitbit charge 2 during wheelchair activities in people with spinal cord injury: instrument validation study	2022	10.2196/27637	SCOPUS	off topic	no

accuracy of heart-rate-recovery parameters assessed from a wrist-worn photoplethysmography monitor (polar unite)	2024	10.1123/ijspp.2023-0120	SCOPUS	not accessible	no
accuracy of optical heart rate sensing technology in wearable fitness trackers for young and older adults: validation and comparison study	2020	10.2196/14707	SCOPUS	off topic	no
accuracy of the wearable activity tracker garmin forerunner 235 for the assessment of heart rate during rest and activity	2019	10.1080/02640414.2018.1535563	SCOPUS	off topic	no
accuracy of wearable devices for measuring heart rate during conventional and nordic walking	2021	10.1002/pmrj.12424	SCOPUS	off topic	no
accurate detection of heart rate and blood oxygen saturation in reflective photoplethysmography	2020	10.1109/isspit51521.2020.9408845	SCOPUS	inaccessible dataset	no
accurate heart rate monitoring during physical exercises using ppg	2017	10.1109/tbme.2017.2676243	SCOPUS	inaccessible dataset	no
accurate heart rate monitoring method during physical exercise from photoplethysmography signal	2019	10.1109/jsen.2018.2886001	SCOPUS	inaccessible dataset	no
accurate heart-rate estimation from face videos using quality-based fusion	2017	10.1109/icip.2017.8297060	SCOPUS	off topic	no
acquiring photoplethysmography (ppg) signal without led	2023	10.1109/i2mtc53148.2023.10175960	SCOPUS	off topic	no
adapting smartphone-based photoplethysmograpy to suboptimal scenarios	2017	10.1088/1361-6579/aa51db	SCOPUS	not accessible	no
adaptive algorithm for motion artifacts removal in wearable biomedical sensors during physical exercise	2023	10.1109/jsen.2023.3256959	SCOPUS	denoising	no
adaptive behavioral model of the electricity object management operator for intelligent current personnel condition monitoring systems	2020	10.1007/978-3-030-33491-8_38	SCOPUS	off topic	no
adaptive notch-filtration to effectively recover photoplethysmographic signals during physical activity	2022	10.1016/j.bspc.2021.103303	SCOPUS	denoising	no
adaptive scheduling of acceleration and gyroscope for motion artifact cancelation in photoplethysmography	2022	10.1016/j.cmpb.2022.107126	SCOPUS	denoising	no
advance continuous monitoring of blood pressure and respiration rate using denoising auto encoder and lstm	2022	10.1007/s00542-022-05249-0	SCOPUS	off topic	no
advance monitoring of blood pressure and respiratory rate using de-noising auto encoder	2021	10.1115/isps2021-65921	SCOPUS	off topic	no

affective video recommender systems: a survey	2022	10.3389/ fnins.2022.984404	SCOPUS	off topic	no
afodss: heart rate estimation method from photoplethysmographic signals with motion artifacts using fourier-sparse dual optimization	2019	10.1109/ jsen.2019.2928643	SCOPUS	inaccessible dataset	no
ambient light-driven wireless wearable finger patch for monitoring vital signs from ppg signal	2024	10.1109/ jsen.2023.3335309	SCOPUS	off topic	no
an adaptive filter based motion artifact cancellation technique using multi-wavelength ppg for accurate hr estimation	2023	10.1109/ tbcas.2023.3315297	SCOPUS	denoising	no
an applicable approach for extracting human heart rate and oxygen saturation during physical movements using a multi-wavelength illumination optoelectronic sensor system	2018	10.1117/12.2287854	SCOPUS	off topic	no
an automated device for recording peripheral arterial waveform	2019	10.23919/ cinc49843.2019.900 5815	SCOPUS	off topic	no
an edge-based scheme to support heart rate estimation for different physical exercises	2021	10.1002/itl2.192	SCOPUS	inaccessible dataset	no
an effective photoplethysmography heart rate estimation framework integrating two-level denoising method and heart rate tracking algorithm guided by finite state machine	2022	10.1109/ jbhi.2022.3165071	SCOPUS	inaccessible dataset	no
an extended system for external sensors data acquisition and validation during conducting polysomnography	2023	10.1109/ ssp53291.2023.1020 8050	SCOPUS	off topic	no
an improved algorithm for heart rate tracking during physical exercise using simultaneous wrist-type photoplethysmographic (ppg) and acceleration signals	2017	10.1109/ icbme.2016.7890946	SCOPUS	inaccessible dataset	no
an ml-based approach to reconstruct heart rate from ppg in presence of motion artifacts	2023	10.3390/ bios13070718	SCOPUS	inaccessible dataset	no
an overview of wearable photoplethysmographic sensors and various algorithms for tracking of heart rates †	2022	10.3390/ engproc2021010077	SCOPUS	review	no
an ultralow-power ppg sensor with adaptive predictive sampling	2024	10.1109/ jsen.2024.3378115	SCOPUS	hardware	no

an xgboost-based physical fitness evaluation model using advanced feature selection and bayesian hyper-parameter optimization for wearable running monitoring	2019	10.1016/ j.comnet.2019.01.026	SCOPUS	off topic	no
analysis of heart rate variability from multichannel photoplethysmography	2017	10.3233/978-1-61499-800-6-217	SCOPUS	multichannel	no
analysis of heart rate variability using wearable device	2021	10.1007/978-981-33-4069-5_37	SCOPUS	inaccessible dataset	no
analysis of minimum face video duration and the effect of video compression to image-based non-contact heart rate monitoring system	2020	10.11591/ eei.v9i1.1855	SCOPUS	off topic	no
analysis of non-invasive video based heart rate monitoring system obtained from various distances and different facial spot	2018	10.1088/1742-6596/1049/1/012003	SCOPUS	off topic	no
analysis of photoplethysmogram signal to estimate heart rate during physical activity using fractional fourier transform – a sampling frequency independent and reference signal-less method	2023	10.1016/ j.cmpb.2022.107294	SCOPUS	inaccessible dataset	no
analysis of vital signs using remote photoplethysmography (rppg)	2023	10.1007/s12652-023-04683-w	SCOPUS	off topic	no
and-rppg: a novel denoising-rppg network for improving remote heart rate estimation	2022	10.1016/ j.compbiomed.2021.105146	SCOPUS	off topic	no
application of neural networks for heart rate monitoring	2020	10.1016/ j.ifacol.2020.12.605	SCOPUS	inaccessible dataset	no
applying a deep learning network in continuous physiological parameter estimation based on photoplethysmography sensor signals	2022	10.1109/ jsen.2021.3126744	SCOPUS	off topic	no
artifact detection of wrist photoplethysmograph signals	2018	10.5220/0006594301820189	SCOPUS	denoising	no
artifact removal from data generated by nonlinear systems: heart rate estimation from blood volume pulse signal	2020	10.1021/ acs.iecr.9b04824	SCOPUS	inaccessible dataset	no
assessment of samsung galaxy watch4 ppg-based heart rate during light-to-vigorous physical activities	2024	10.1109/ lsens.2024.3408089	SCOPUS	off topic	no
association between coronary artery atherosclerosis and plasma glucose levels assessed by dual-source computed tomography	2018	10.21037/ jtd.2018.10.62	SCOPUS	off topic	no

association of baseline optical coherence tomography angiography with the development of glaucomatous visual field defects in preperimetric glaucoma eyes	2023	10.1136/bjo-2021-321025	SCOPUS	off topic	no
automated heart rate detection in seismocardiograms using electrocardiogram-based algorithms—a feasibility study	2024	10.3390/bioengineering11060596	SCOPUS	off topic	no
automatic heart rate detection during sleep using tracheal audio recordings from wireless acoustic sensor	2023	10.3390/diagnostics13182914	SCOPUS	off topic	no
autonomous heart rate tracking methodology using kalman filter and the em algorithm	2019	10.23919/fusion43075.2019.9011407	SCOPUS	inaccessible dataset	no
binary cornet: accelerator for hr estimation from wrist-ppg	2020	10.1109/tbcas.2020.3001675	SCOPUS	inaccessible dataset	no
blending human and machine: feasibility of measuring fatigue through the aviation headset	2020	10.1177/0018720819849783	SCOPUS	off topic	no
blood pressure and heart rate measurements using photoplethysmography with modified lrcn	2022	10.32604/cmc.2022.022679	SCOPUS	INCLUDED	yes
blood pressure estimation using photoplethysmogram signal and its morphological features	2020	10.1109/jsen.2019.2961411	SCOPUS	off topic	no
blood volume pulse extraction for non-contact heart rate measurement by digital camera using singular value decomposition and burg algorithm	2018	10.3390/en11051076	SCOPUS	off topic	no
boosting algorithms based cuff-less blood pressure estimation from clinically relevant ecg and ppg morphological features	2023	10.1109/embc40787.2023.10340405	SCOPUS	off topic	no
bp signal analysis using emerging techniques and its validation using ecg signal	2021	10.1007/s11220-021-00349-z	SCOPUS	off topic	no
brief research report: evaluation of photoplethysmographic heart rate monitoring for sheep under heat-stressed conditions	2022	10.3389/fanim.2022.1046557	SCOPUS	animal	no
brno university of technology smartphone ppg database (but ppg): annotated dataset for ppg quality assessment and heart rate estimation	2021	10.1155/2021/3453007	SCOPUS	off topic	no
camera-based cardiovascular screening based on heart rate and its variability in pre-and post-exercise conditions	2023	10.1109/embc40787.2023.10340871	SCOPUS	off topic	no

cardiac pulse monitoring through multi-scale spectrum	2024	10.1109/amathe61652.2024.10582227	SCOPUS	off topic	no
casinor: combination of adaptive filters using single noise reference signal for heart rate estimation from ppg signals	2020	10.1007/s11760-020-01692-6	SCOPUS	inaccessible dataset	no
changes in maternal heart rate variability and photoplethysmography morphology after corticosteroid administration: a prospective, observational study	2024	10.3390/jcm13082442	SCOPUS	off topic	no
characterization of a ppg wearable sensor to be embedded into an innovative ring-shaped device for healthcare monitoring	2021	10.1007/978-3-030-63107-9_5	SCOPUS	off topic	no
characterization of quadratic nonlinearity between motion artifact and acceleration data and its application to heartbeat rate estimation	2017	10.3390/s17081872	SCOPUS	denoising	no
characterization study of neck photoplethysmography	2018	10.1109/embc.2018.8513247	SCOPUS	off topic	no
chest wearable apparatus for cuffless continuous blood pressure measurements based on ppg and pcg signals	2020	10.1109/access.2020.2981300	SCOPUS	off topic	no
chest-based real-time pulse and respiration monitoring based on bio-impedance	2020	10.1109/embc44109.2020.9176348	SCOPUS	off topic	no
classifying engagement in e-learning through gru-tcn model using photoplethysmography signals	2024	10.1016/j.bspc.2023.105903	SCOPUS	off topic	no
classifying nocturnal blood pressure patterns using photoplethysmogram features	2022	10.1109/embc48229.2022.9871099	SCOPUS	off topic	no
clinical application research on wireless and remote continuous monitoring technology for maternal hemodynamics	2024	10.7517/issn.1674-0475.230716	SCOPUS	off topic	no
clinical applications of contactless photoplethysmography for vital signs monitoring in pediatrics: a systematic review and meta-analysis	2023	10.1017/cts.2023.557	SCOPUS	review	no
clinical validation of heart rate apps: mixed-methods evaluation study	2017	10.2196/mhealth.7254	SCOPUS	off topic	no

closed loop nitrified foam mpd delivers unprecedented drilling performance in a mature sour gas field in pakistan	2021	10.2523/iptc-21266-ms	SCOPUS	off topic	no
comparative assessment of smartwatch photoplethysmography accuracy	2024	10.1109/lsens.2023.3342292	SCOPUS	off topic	no
comparing remote heart rate accuracy using different wavelengths	2021	10.1109/scored53546.2021.9652720	SCOPUS	off topic	no
comparison between heart rate variability and pulse rate variability for bradycardia and tachycardia subjects	2018	10.1109/iccais.2018.8570697	SCOPUS	off topic	no
comparison of region of interest segmentation methods for video-based heart rate measurements	2018	10.1109/bibe.2018.00034	SCOPUS	off topic	no
comparison of seven shallow and deep regressors in continuous blood pressure and heart rate estimation using single-channel photoplethysmograms under three evaluation cases	2023	10.1016/j.bspc.2023.105029	SCOPUS	does not propose a new technique	no
comparison of wristband type devices to measure heart rate variability for mental stress assessment	2021	10.1109/ictc52510.2021.9620772	SCOPUS	off topic	no
comparisons in heart rate readings between the bioconnected wireless exercise earpiece and a polar t31-coded chest strap during a gxt	2018	10.14198/jhse.2018.133.05	SCOPUS	off topic	no
complementary photoplethysmogram synthesis from electrocardiogram using generative adversarial network	2021	10.1109/access.2021.3078534	SCOPUS	off topic	no
complete cardiorespiratory monitoring via wearable ultra low power ultrasound	2023	10.1109/ius51837.2023.10307398	SCOPUS	off topic	no
comprate: power efficient heart rate and heart rate variability monitoring on smartwearables	2019	10.1145/3359996.3364239	SCOPUS	off topic	no
compressed domain feature extraction	2019	10.1007/978-3-030-05870-8_4	SCOPUS	off topic	no
compressed estimation of heart and respiratory rates from a photoplethysmogram	2017	10.1109/biocas.2017.8325158	SCOPUS	off topic	no
contact and non-contact heart beat rate measurement techniques: challenges and issues	2021	10.47836/pjst.29.3.03	SCOPUS	off topic	no

contact-free monitoring of physiological parameters in people with profound intellectual and multiple disabilities	2019	10.1109/ iccvw.2019.00206	SCOPUS	off topic	no
contactless vital sign monitoring system for in-vehicle driver monitoring using a near-infrared time-of-flight camera	2022	10.3390/ app12094416	SCOPUS	off topic	no
continuous blood pressure monitoring from an autonomic nervous system perspective	2023	10.1117/12.2647821	SCOPUS	off topic	no
continuous heart rhythm monitoring using mobile photoplethysmography in ambulatory patients	2020	10.1016/ j.jelectrocard.2020.04.017	SCOPUS	inaccessible dataset	no
continuous maternal hemodynamics monitoring at delivery using a novel, noninvasive, wireless,ppg-based sensor	2021	10.3390/ jcm10010008	SCOPUS	off topic	no
continuous measurement of wrist artery pulse vibration signals using structured-light projection method	2020	10.1117/12.2555341	SCOPUS	off topic	no
cornet: deep learning framework for ppg-based heart rate estimation and biometric identification in ambulant environment	2019	10.1109/ tbcas.2019.2892297	SCOPUS	off topic	no
correction: resting and postexercise heart rate detection from fingertip and facial photoplethysmography using a smartphone camera: a validation study (jmir mhealth uhealth, (2017) 5, 3, 10.2196/mhealth.7275)	2019	10.2196/11616	SCOPUS	off topic	no
correlation analysis of heart rate variability between ppg and ecg for wearable devices in different postures	2017	10.1109/ smc.2017.8123077	SCOPUS	off topic	no
correlation between heart rate variability, blood pressure and heart function in patients with essential hypertension depending on the type of therapy	2021	10.18705/1607-419x-2020-26-5-581-589	SCOPUS	off topic	no
coverage of ppg-based wearable devices in office tasks	2023	10.22489/ cinc.2023.206	SCOPUS	off topic	no
criterion validation of an open-source wearable physiological sensors device	2021		SCOPUS	hardware	no
data-augmentation for deep learning based remote photoplethysmography methods	2021	10.1109/ ehb52898.2021.9657650	SCOPUS	off topic	no

deep learning based pulse rate prediction in noisy ppg signals with practical application to wearables; [derin öğrenme ile gürültülü ppg işaretlerde nabiz kestirimi ve giyilebilir teknolojilere uygulanmasi]	2022	10.1109/siu55565.2022.9864877	SCOPUS	language no supported	no
deep learning for heart rate estimation from reflectance photoplethysmography with acceleration power spectrum and acceleration intensity	2020	10.1109/access.2020.2981956	SCOPUS	not accessible	no
deep learning fused wearable pressure and ppg data for accurate heart rate monitoring	2021	10.1109/jsen.2021.3123243	SCOPUS	multichannel	no
deep learning-based image enhancement for robust remote photoplethysmography in various illumination scenarios	2023	10.1109/cvprw59228.2023.00647	SCOPUS	off topic	no
deepheart: a deep learning approach for accurate heart rate estimation from ppg signals	2021	10.1145/3441626	SCOPUS	inaccessible dataset	no
deepheart: accurate heart rate estimation from ppg signals based on deep learning	2019	10.1109/mass.2019.00051	SCOPUS	inaccessible dataset	no
deeppulse: an uncertainty-aware deep neural network for heart rate estimations from wrist-worn photoplethysmography	2022	10.1109/embc48229.2022.9871813	SCOPUS	multichannel	no
delnet based systolic peak delineation in remote ppg signal from facial video frames	2023	10.1109/icccnt56998.2023.10306618	SCOPUS	off topic	no
demo: a robust and customizable tracking algorithm for accurate heart rate estimation	2019	10.1145/3307334.3328573	SCOPUS	missing technical details	no
deriving heart rate and respiratory rate from pulse oximetry using neural networks	2021	10.1109/hnicem54116.2021.9731861	SCOPUS	off topic	no
design and development of a new wearable optic patch towards increased functionality and reduced motion artifacts	2024	10.1117/12.3002506	SCOPUS	off topic	no
design and development of a wristband for continuous vital signs monitoring of covid-19 patients	2021	10.1109/embc46164.2021.9630299	SCOPUS	off topic	no
design and implementation of 1024 point pipelined radix 4 fft processor on fpga for biomedical signal processing applications	2020	10.1109/ises50453.2020.00012	SCOPUS	hardware	no
design and implementation of a wearable wrist-type band for heart rate monitoring in motion status	2017		SCOPUS	language no supported	no

design and implementation of an spo2 based sensor for heart monitoring using an android application	2020	10.1088/1742-6596/1447/1/012004	SCOPUS	off topic	no
design and implementation of auscultation blood pressure measurement using vascular transit time and physiological parameters	2017	10.1109/smc.2017.8123084	SCOPUS	off topic	no
design of a low-cost wearable heart and respiratory rate measurement device using an arduino and bluetooth module	2021	10.1109/icccnt51525.2021.9579536	SCOPUS	hardware	no
design of a new long-time continuous photoplethysmography signal acquisition system to obtain accurate measurement of heart rate	2020	10.1115/isps2020-1916	SCOPUS	language no supported	no
design of a physiological parameter monitoring system, implementing internet of things communication protocols by using embedded systems	2022	10.1109/ropec55836.2022.10018715	SCOPUS	off topic	no
detection and removal of motion artifacts in ppg signals	2022	10.1007/s11036-019-01323-6	SCOPUS	denoising	no
detection of beat-to-beat intervals from wrist photoplethysmography in patients with sinus rhythm and atrial fibrillation after surgery	2018	10.1109/bhi.2018.8333387	SCOPUS	inaccessible dataset	no
detection of cardiovascular disease based on ppg signals using machine learning with cloud computing	2022	10.1155/2022/1672677	SCOPUS	off topic	no
detection of heart rate using smartphone gyroscope data: a scoping review	2023	10.3389/fcvm.2023.1329290	SCOPUS	review	no
determination of absolute heart beat from photoplethysmographic signals in the presence of motion artifacts	2018	10.1109/icaecc.2018.8479479	SCOPUS	inaccessible dataset	no
determination of saturation, heart rate, and respiratory rate at forearm using a nellcor™ forehead spo2-saturation sensor	2017	10.1007/s10877-016-9940-7	SCOPUS	off topic	no
development and clinical application of a novel autonomic transient response-based screening system for major depressive disorder using a fingertip photoplethysmographic sensor	2018	10.3389/fbioe.2018.00064	SCOPUS	off topic	no
development and validation of heart rate - incorporated finger photoplethysmography fitness index algorithm for cardiovascular disease risk assessment	2019	10.1109/iecbes.2018.08626725	SCOPUS	off topic	no

development of a combined time-frequency technique for accurate extraction of pnn50 metric from noisy heart rate measurements	2018	10.1007/s41315-018-0052-z	SCOPUS	off topic	no
development of a continuous blood pressure measurement and cardiovascular multi-indicator platform for asian populations by using a back propagation neural network and dual photoplethysmography sensor signal acquisition technology	2021	10.1155/2021/6613817	SCOPUS	off topic	no
development of a continuous blood pressure monitoring system based on pulse transit time and hemodynamic covariates	2020		SCOPUS	off topic	no
development of a custom wrist wearable for use in nursing homes	2023	10.1145/3580252.3589434	SCOPUS	off topic	no
development of a low cost heart rate monitoring and transmission system using ppg signal processing for wearable devices	2019	10.35940/ ijitee.j9829.0881019	SCOPUS	inaccessible dataset	no
development of a wearable in-ear ppg system for continuous monitoring	2020	10.1109/ jsen.2020.3008479	SCOPUS	hardware	no
doppler ultrasound based non-invasive heart rate telemonitoring system for wellbeing assessment	2018	10.5815/ ijisa.2018.12.07	SCOPUS	off topic	no
dual wavelength photoplethysmography framework for heart rate calculation	2022	10.3390/s22249955	SCOPUS	multichannel	yes
early physiologic numerical and waveform characteristics of simulated hemorrhagic events with healthy volunteers donating blood	2024	10.1097/ cce.0000000000001073	SCOPUS	off topic	no
ecg augmented pulse oximetry in atlantic salmon (salmo salar)—a pilot study	2023	10.1016/ j.compag.2023.108081	SCOPUS	off topic	no
edge-based computation of super-resolution superlet spectrograms for real-time estimation of heart rate using an iomt-based reference-signal-less ppg sensor	2024	10.1109/ jiot.2023.3322947	SCOPUS	off topic	no
edge-enabled heart rate estimation from multisensor ppg signals	2023	10.1155/2023/4682760	SCOPUS	multichannel	no
effect of ambient lighting and skin tone on estimation of heart rate and pulse transit time from video plethysmography	2020	10.1109/ embc44109.2020.9176731	SCOPUS	off topic	no

effect of increasing heart rate on finger photoplethysmography fitness index (ppgf) in subjects with implanted cardiac pacemakers	2018	10.1371/journal.pone.0207301	SCOPUS	off topic	no
effect of position and fastening belt pressure on the accuracy of ppg-based heart rate sensor	2018	10.1109/embc.2018.8513333	SCOPUS	off topic	no
effects of lifestyle intervention on plasma trimethylamine n-oxide in obese adults	2019	10.3390/nu11010179	SCOPUS	off topic	no
effects of pulse transit time and physiological differences on wearable device based blood pressure estimation	2017	10.1109/cacs.2016.7973917	SCOPUS	off topic	no
efficacy and safety profile of sitagliptin, vildagliptin, and metformin in newly diagnosed type 2 diabetic subjects	2021	10.1111/1440-1681.13561	SCOPUS	off topic	no
elicitation of anxiety without time pressure and its detection using physiological signals and artificial intelligence: a proof of concept	2024	10.1109/access.2024.3362668	SCOPUS	off topic	no
embedded low power heart rate estimation processor for flexible applications	2022	10.1109/ifetc53656.2022.9948467	SCOPUS	off topic	no
embedding temporal convolutional networks for energy-efficient ppg-based heart rate monitoring	2022	10.1145/3487910	SCOPUS	multichannel	yes
emotion based media playback system using ppg signal	2021	10.1109/wispnet51692.2021.9419475	SCOPUS	off topic	no
empowering people with a user-friendly wearable platform for unobtrusive monitoring of vital physiological parameters	2022	10.3390/s22145226	SCOPUS	off topic	no
encoding physical conditioning from inertial sensors for multi-step heart rate estimation	2021	10.1007/978-3-030-91699-2_5	SCOPUS	off topic	no
end-to-end ppg processing pipeline for wearables: from quality assessment and motion artifacts removal to hr/hrv feature extraction	2023	10.1109/bibm58861.2023.10385998	SCOPUS	inaccessible dataset	no
energy-efficient photoplethysmogram compression to estimate heart and respiratory rates simultaneously	2019	10.1109/access.2019.2919745	SCOPUS	off topic	yes
energy-efficient wearable-to-mobile offload of ml inference for ppg-based heart-rate estimation	2023	10.23919/date56975.2023.10137129	SCOPUS	hardware	no

enhanced contactless heart rate monitoring using camera with motion artifact removal during physical activities	2023	10.1109/ embc40787.2023.10 340279	SCOPUS	off topic	no
enhancement of remote ppg and heart rate estimation with optimal signal quality index	2022	10.1109/ bsn56160.2022.992 8503	SCOPUS	off topic	no
ensemble empirical mode decomposition with principal component analysis: a novel approach for extracting respiratory rate and heart rate from photoplethysmographic signal	2018	10.1109/ jbhi.2017.2679108	SCOPUS	INCLUDED	yes
essential feature extraction of photoplethysmography signal of men and women in their 20s	2017	10.4186/ ej.2017.21.4.259	SCOPUS	off topic	no
estimating heart rate and respiratory rate from a single lead electrocardiogram using ensemble empirical mode decomposition and spectral data fusion	2021	10.3390/s21041184	SCOPUS	off topic	no
estimating heart rate during steady-state activities and transitions based on signal of ppg and acc	2020	10.1109/ icma49215.2020.923 3764	SCOPUS	inaccessible dataset	no
estimation and validation of arterial blood pressure using photoplethysmogram morphology features in conjunction with pulse arrival time in large open databases	2021	10.1109/ jbhi.2020.3009658	SCOPUS	off topic	no
estimation of beat-by-beat blood pressure and heart rate from ecg and ppg using a fine-tuned deep cnn model	2022	10.1109/ access.2022.319585 7	SCOPUS	multichannel	yes
estimation of beat-to-beat interval from wearable photoplethysmography sensor on different measurement sites during daily activities	2018	10.1109/ icsens.2018.858961 1	SCOPUS	inaccessible dataset	no
estimation of breathing rate and heart rate from photoplethysmogram	2017	10.1109/ iceei.2017.8312414	SCOPUS	inaccessible dataset	no
estimation of heart rate variability parameters by machine learning approaches applied to facial infrared thermal imaging	2022	10.3389/ fcvm.2022.893374	SCOPUS	off topic	no
estimation of hrv and spo2 from wrist-worn commercial sensors for clinical settings	2018	10.1109/ bsn.2018.8329679	SCOPUS	off topic	no
evaluating reliability in wearable devices for sleep staging	2024	10.1038/s41746-024-01016-9	SCOPUS	off topic	no

evaluating the accuracy of heart rate sensors based on photoplethysmography for in-the-wild analysis	2019	10.1145/3329189.3329215	SCOPUS	inaccessible dataset	no
evaluation of autonomic nervous system, saliva cortisol levels, and cognitive function in major depressive disorder patients	2018	10.1155/2018/7343592	SCOPUS	off topic	no
evaluation of blood pressure estimation models based on pulse arrival time	2020	10.1016/j.compeleceng.2020.106616	SCOPUS	off topic	no
evaluation of the accuracy and reliability for photoplethysmography based heart rate and beat-to-beat detection during daily activities	2017	10.1007/978-981-10-5122-7_37	SCOPUS	inaccessible dataset	no
event-related driver stress detection with smartphones among young novice drivers	2022	10.1080/00140139.2021.2020342	SCOPUS	off topic	no
experimental investigation of fluidic drag on pvc pipes	2017		SCOPUS	off topic	no
experimental low cost reflective type oximeter for wearable health systems	2017	10.1016/j.bspc.2016.09.013	SCOPUS	off topic	no
exploiting system configurability towards dynamic accuracy-power trade-offs in sensor front-ends	2017	10.1109/acssc.2016.7869524	SCOPUS	off topic	no
exploring the technological dimension of autonomous sensory meridian response-induced physiological responses	2024	10.7717/peerj.17754	SCOPUS	off topic	no
external temperature sensor assisted a new low power photoplethysmography readout system for accurate measurement of the bio-signs	2021	10.1007/s00542-020-05106-y	SCOPUS	off topic	no
extraction of heart rate from ppg signal: a machine learning approach using decision tree regression algorithm	2019	10.1109/eict48899.2019.9068845	SCOPUS	inaccessible dataset	no
face video based touchless blood pressure and heart rate estimation	2017	10.1109/mmsp.2016.7813389	SCOPUS	off topic	no
facial video-based remote photoplethysmography signal estimation with vision transformer	2023	10.1109/icce-asia59966.2023.10326412	SCOPUS	off topic	no
facial-video-based physiological signal measurement: recent advances and affective applications	2021	10.1109/msp.2021.3106285	SCOPUS	off topic	no
feasibility of a wireless vital signal monitoring system in the nicu	2023	10.1109/bsn58485.2023.10331194	SCOPUS	off topic	no

feasibility of a wrist-worn wearable device for estimating mental health status in patients with mental illness	2023	10.3389/ fpsyt.2023.1189765	SCOPUS	off topic	no
feasibility of electrodermal activity and photoplethysmography data acquisition at the foot using a sock form factor	2023	10.3390/s23020620	SCOPUS	off topic	no
feasibility study of deep neural network for heart rate estimation from wearable photoplethysmography and acceleration signals	2019	10.1109/ embc.2019.8857618	SCOPUS	multichannel	no
feasible assessment of recovery and cardiovascular health: accuracy of nocturnal hr and hrv assessed via ring ppg in comparison to medical grade ecg	2020	10.1088/1361-6579/ab840a	SCOPUS	off topic	no
feature-level cross-attentional ppg and motion signal fusion for heart rate estimation	2023	10.1109/ compsac57700.2023.00267	SCOPUS	INCLUDED	yes
features extraction from cardiac-related signals: comparison among different measurement methods	2024	10.1088/1742-6596/2698/1/012026	SCOPUS	off topic	no
finding a way into an interpreter’s heart: methodological considerations on heart-rate variability building on an exploratory study	2022	10.13137/2421-714x/34392	SCOPUS	off topic	no
fingertip plethysmography using a sensitive wafer-level-packaged capacitive mems strain sensor	2024	10.1109/ mems58180.2024.10439370	SCOPUS	off topic	no
finite state machine framework for instantaneous heart rate validation using wearable photoplethysmography during intensive exercise	2019	10.1109/ jbhi.2018.2871177	SCOPUS	inaccessible dataset	no
first evaluation of the ptn-104 plethysmographic sensor for heart rate measurement	2021	10.2478/msr-2021-0017	SCOPUS	off topic	no
freq2time: weakly supervised learning of camera-based rppg from heart rate	2024	10.1109/ icassp48485.2024.10446054	SCOPUS	off topic	no
from lab to life: evaluating the reliability and validity of psychophysiological data from wearable devices in laboratory and ambulatory settings	2024	10.3758/s13428-024-02387-3	SCOPUS	off topic	no
fusion method to estimate heart rate from facial videos based on rppg and rbcg	2021	10.3390/s21206764	SCOPUS	off topic	no
harmonic sum-based method for heart rate estimation using ppg signals affected with motion artifacts	2018	10.1007/s12652-016-0422-z	SCOPUS	inaccessible dataset	no

harnessing magnetite poly(ethylene glycol)-block-poly(propylene glycol)-block-poly(ethylene glycol) (peg-ppg-peg) composites for efficient arsenic removal in water treatment	2023	10.1007/s41101-023-00205-z	SCOPUS	off topic	no
head hemodynamics and systemic responses during auditory stimulation	2022	10.14814/phy2.15372	SCOPUS	off topic	no
hearables: in-ear multimodal data fusion for robust heart rate estimation	2024	10.3390/biomedinformatics4020051	SCOPUS	off topic	no
heart action monitoring from pulse signals using a growing hybrid polynomial network	2023	10.1016/j.engappai.2022.105584	SCOPUS	off topic	no
heart pulse demodulation from emfit mattress sensor using spectral and source separation techniques	2022	10.22489/cinc.2022.304	SCOPUS	off topic	no
heart rate analysis through smartphone camera	2021	10.1109/metroind4.0iot51437.2021.9488509	SCOPUS	off topic	no
heart rate and breathing rate calculated from cheeks and lips using green and derived colors from video	2020	10.1109/ises50453.2020.00016	SCOPUS	off topic	no
heart rate and heart rate variability as classification features for mental fatigue using short-term ppg signals via smartphones instead of ecg recordings	2021	10.1109/iccsn52437.2021.9463614	SCOPUS	off topic	no
heart rate assessment by means of a novel approach applied to signals of different nature	2017	10.1088/1742-6596/778/1/012001	SCOPUS	multichannel	no
heart rate detection using microsoft kinect: validation and comparison to wearable devices	2017	10.3390/s17081776	SCOPUS	hardware	no
heart rate estimation algorithm from wrist-based photoplethysmogram using subspace learning method	2019	10.1109/percomw.2019.8730797	SCOPUS	inaccessible dataset	no
heart rate estimation during exercise from photoplethysmographic signals using convolutional neural network	2019	10.1109/biocas.2019.8919185	SCOPUS	not accessible	no
heart rate estimation from ballistocardiogram signals processing via low-cost telemedicine architectures: a comparative performance evaluation	2023	10.3389/fdgth.2023.1222898	SCOPUS	off topic	no

heart rate estimation from facial video sequences using fast independent component analysis	2022	10.1109/ ncc55593.2022.9806 810	SCOPUS	off topic	no
heart rate estimation from photoplethysmography signal for wearable health monitoring devices	2019	10.1016/ j.bspc.2019.01.021	SCOPUS	inaccessible dataset	no
heart rate estimation from remote photoplethysmography based on light-weight u-net and attention modules	2023	10.1109/ access.2023.328189 8	SCOPUS	off topic	no
heart rate estimation from wrist ppg signal during intense physical exercise	2023	10.1007/s42979-023-02173-6	SCOPUS	multichannel	no
heart rate estimation from wrist-type photoplethysmographic signals corrupted by intense motion artifacts using nlms adaptive filter and spectral peak tracking	2019	10.1109/ icaiit.2019.8834647	SCOPUS	inaccessible dataset	no
heart rate estimation from wrist-type photoplethysmography signals during physical exercise	2020	10.1016/ j.bspc.2019.101790	SCOPUS	inaccessible dataset	no
heart rate estimation from wrist-worn photoplethysmography: a review	2019	10.1109/ jsen.2019.2914166	SCOPUS	review	no
heart rate estimation of ppg signals with simultaneous accelerometry using adaptive neural network filtering	2020	10.1109/ tce.2019.2961263	SCOPUS	inaccessible dataset	no
heart rate extraction from novel neck photoplethysmography signals	2019	10.1109/ embc.2019.8857415	SCOPUS	inaccessible dataset	no
heart rate extraction from photoplethysmography signal: a multi model machine learning approach	2020	10.1109/ iicaiet49801.2020.92 57869	SCOPUS	inaccessible dataset	no
heart rate extraction from ppg signals using variational mode decomposition	2019	10.1016/ j.bbe.2018.11.001	SCOPUS	INCLUDED	yes
heart rate measurement on pc and phone using facial videos	2023	10.1109/ kst57286.2023.1008 6729	SCOPUS	off topic	no
heart rate monitoring during physical exercise from photoplethysmography using neural network	2019	10.1109/ lsens.2018.2878207	SCOPUS	inaccessible dataset	no
heart rate monitoring of the endurance runner during high intensity interval training: influence of device used on training functions	2023	10.1177/175433712 11037035	SCOPUS	off topic	no
heart rate monitoring using external camera	2023	10.1007/978-981-99-4932-8_36	SCOPUS	off topic	no

heart rate monitoring using ppg with smartphone camera	2021	10.1109/ bibm52615.2021.9669735	SCOPUS	off topic	no
heart rate monitoring using sparse spectral curve tracing	2020	10.1109/ embc44109.2020.9175349	SCOPUS	INCLUDED	yes
heart rate variability analysis from electrocardiogram (ecg) and photoplethysmogram (ppg) signals by using soft computing technique	2023	10.1109/ ihcsp56702.2023.10127200	SCOPUS	multichannel	no
heart rate variability and heart rate monitoring of nurses using ppg and ecg signals during working condition: a pilot study	2022	10.1002/hsr2.477	SCOPUS	off topic	no
heart rate variability extraction from videos signals: ica vs. evm comparison	2017	10.1109/ access.2017.2678521	SCOPUS	off topic	no
heart: motion-resilient heart rate monitoring with in-ear microphones	2023	10.1109/ percom56429.2023.10099317	SCOPUS	off topic	no
heartbeat and respiration rate prediction using combined photoplethysmography and ballisto cardiography	2023	10.32604/ iasc.2023.032155	SCOPUS	multichannel	no
heartbeat rate estimation using convolutional neural network	2024	10.1109/ icict60155.2024.10544653	SCOPUS	off topic	no
hepatic venous pressure gradient and rebleeding risk of patients with nonalcoholic steatohepatitis cirrhosis after variceal bleeding	2023	10.3389/ fmed.2023.1224506	SCOPUS	off topic	no
high-barrier-height ti3c2tx/si microstructure schottky junction-based self-powered photodetectors for photoplethysmographic monitoring	2022	10.1002/ admt.202200555	SCOPUS	off topic	no
high-responsivity van der waals schottky photodiodes based on maskless etched wafer-scale silicon nanoholes	2024	10.1109/ led.2024.3376001	SCOPUS	hardware	no
highly wearable cuff-less blood pressure and heart rate monitoring with single-arm electrocardiogram and photoplethysmogram signals	2017	10.1186/s12938-017-0317-z	SCOPUS	multichannel	no
hreadai: heart rate estimation from face mask videos by consolidating eulerian and lagrangian approaches	2024	10.1109/ tim.2023.3334359	SCOPUS	off topic	no

hrsense: heart rate remote sensing through bluetooth channel using a photoplethysmograph[formula presented]	2023	10.1016/ j.simpa.2023.100579	SCOPUS	off topic	no
human activity recognition using accelerometer and photoplethysmographic signals	2018	10.1007/978-3-319-59424-8_6	SCOPUS	off topic	no
human health monitoring technology	2017	10.1117/12.2262399	SCOPUS	off topic	no
identification of ictal tachycardia in focal motor- and non-motor seizures by means of a wearable ppg sensor	2021	10.3390/s21186017	SCOPUS	off topic	no
illumination variation interference suppression in remote ppg using pls and memd	2017	10.1049/ el.2016.3611	SCOPUS	off topic	no
implementation of adaptive threshold for peak detection of photoplethysmography applied on microcontroller	2019	10.1109/ iceeie47180.2019.8981423	SCOPUS	hardware	no
improved heart rate estimation from photoplethysmography during physical exercise using combination of nlms and rls adaptive filters	2018	10.1109/ tencon.2018.8650495	SCOPUS	inaccessible dataset	no
improved heart rate tracking using multiple wrist-type photoplethysmography during physical activities	2018	10.1109/ embc.2018.8512736	SCOPUS	inaccessible dataset	no
improved method for motion artifact reduction from finger photoplethysmogram signal	2023		SCOPUS	denoising	no
improvement of germinated brown rice quality with autoclaving treatment	2020	10.1002/fsn3.1459	SCOPUS	off topic	no
improving cuff-less continuous blood pressure estimation with linear regression analysis	2022	10.3390/ electronics11091442	SCOPUS	off topic	no
improving heart rate and heart rate variability estimation from video through a hr-rr-tuned filter	2023	10.1109/ icassp49357.2023.10096576	SCOPUS	off topic	no
improving heart rate estimation on consumer grade wrist-worn device using post-calibration approach	2020	10.1109/ jsen.2020.2979191	SCOPUS	inaccessible dataset	no
improving ppg-based heart-rate monitoring with synthetically generated data	2022	10.1109/ biocas54905.2022.9948584	SCOPUS	multichannel	no
in your ear: a multimodal hearables device for the assessment of the state of body and mind	2023	10.1109/ mpuls.2024.3357008	SCOPUS	off topic	no

in-ear ppg for vital signs	2022	10.1109/ mprv.2021.3121171	SCOPUS	off topic	no
in-ear pulse rate measurement: a valid alternative to heart rate derived from electrocardiography?	2019	10.3390/s19173641	SCOPUS	off topic	no
incidence of multiple metachronous gastric cancers after pyloric-preserving gastrectomy	2020	10.1007/s00268-020-05492-4	SCOPUS	off topic	no
increase of oxygenation ability in a population of professional flautists with the new and innovative tool of diaphragmatic respiratory re-education in orthostasis	2020	10.19193/0393-6384_2020_4_369	SCOPUS	off topic	no
information retrieval from photoplethysmographic sensors: a comprehensive comparison of practical interpolation and breath-extraction techniques at different sampling rates	2022	10.3390/s22041428	SCOPUS	off topic	no
information-based similarity of ordinal pattern sequences as a novel descriptor in obstructive sleep apnea screening based on wearable photoplethysmography bracelets	2022	10.3390/ bios12121089	SCOPUS	off topic	no
insights into vascular physiology from sleep photoplethysmography	2023	10.1093/sleep/ zsad172	SCOPUS	off topic	no
integrated coronary disease burden and patterns to discriminate vessels benefiting from percutaneous coronary intervention	2022	10.1002/ccd.29983	SCOPUS	off topic	no
integrated smart patch for heart rate and respiratory rate monitoring	2023	10.23919/ measurement59122. 2023.10164383	SCOPUS	off topic	no
integrated vision and sensor based analysis for sleep apnea using featfacenet deep learning	2024	10.1007/s42835-023-01549-1	SCOPUS	off topic	no
interface sensors with skin piezo-thermic transduction enable motion artifact removal for wearable physiological monitoring	2021	10.1016/ j.bios.2021.113325	SCOPUS	off topic	no
investigation on pulse wave forward peak detection and its applications in cardiovascular health	2022	10.1109/ tbme.2021.3103552	SCOPUS	off topic	no
is continuous heart rate monitoring of livestock a dream or is it realistic? a review	2020	10.3390/s20082291	SCOPUS	review	no
is heart rate a confounding factor for photoplethysmography markers? a systematic review	2020	10.3390/ ijerph17072591	SCOPUS	review	no

kick ring ll: a multi-sensor ring capturing respiration, electrocardiogram, oxygen saturation, and skin temperature1	2020	10.1109/embc44109.2020.9176654	SCOPUS	off topic	no
learning based quality indicator aiding heart rate estimation in wrist-worn ppg	2021	10.1109/embc46164.2021.9630910	SCOPUS	inaccessible dataset	no
lgi-rppg-net: a shallow encoder-decoder model for rppg signal estimation from facial video streams	2024	10.1016/j.bspc.2023.105687	SCOPUS	off topic	no
linear and nonlinear analyses of heart rate variability signals under mental load	2022	10.1016/j.bspc.2022.103758	SCOPUS	off topic	no
live demonstration: a ring-type blood pressure monitoring system based on photoplesthygraphy	2017	10.1109/biocas.2017.8325091	SCOPUS	off topic	no
live demonstration: a smart ring for continuous health data monitoring based on photoplethysmography	2023	10.1109/aicas57966.2023.10168644	SCOPUS	off topic	no
live demonstration: a wearable wireless medical sensor network system towards internet-of-patients	2018	10.1109/icsens.2018.8589897	SCOPUS	off topic	no
long-term effects of lowering postprandial glucose level on cardiovascular outcomes in early-stage diabetic patients with coronary artery disease: 10-year post-trial follow-up analysis of the diana study	2023	10.1016/j.jdiacomp.2023.108469	SCOPUS	off topic	no
low complexity heart rate measurement from wearable wrist-type photoplethysmographic sensors robust to motion artifacts	2018	10.1109/icassp.2018.8461520	SCOPUS	multichannel	no
low intricate digital twin method to predict cardiac arrhythmia	2023	10.1109/iceccme57830.2023.10252270	SCOPUS	off topic	no
low-cost microcontrolled based wireless heart rate and oxygen saturation monitor	2018	10.1109/conielecomp.2018.8327195	SCOPUS	hardware	no
low-frequency variability in photoplethysmographic waveform and heart rate during on-pump cardiac surgery with or without cardioplegia	2020	10.1038/s41598-020-58196-z	SCOPUS	off topic	no
low-power continuous heart and respiration rates monitoring on wearable devices	2019	10.1109/icassp.2019.8683825	SCOPUS	multichannel	no

low-power high-sensitivity photoplethysmography sensor for wearable health monitoring system	2021	10.1109/ jsen.2021.3062189	SCOPUS	hardware	no
lstm-based real-time signal quality assessment for blood volume pulse analysis	2023	10.1364/boe.477143	SCOPUS	off topic	no
lstm-only model for low-complexity hr estimation from wrist ppg	2021	10.1109/ embc46164.2021.9630942	SCOPUS	inaccessible dataset	no
machine learning framework for inter-beat interval estimation using wearable photoplethysmography sensors	2024	10.1016/ j.bspc.2023.105689	SCOPUS	inaccessible dataset	no
managing shallow water flow during tophole drilling operations in the southern north sea - a case study	2019	10.2118/194156-ms	SCOPUS	off topic	no
matlab gui design for heart rate monitoring using smart phone camera; [akilli telefon kamerasi kullanarak kalp hizi tahmini için matlab gui tasarimi]	2017	10.1109/ tiptekno.2017.8238030	SCOPUS	off topic	no
measurement and identification of mental workload during simulated computer tasks with multimodal methods and machine learning	2020	10.1080/00140139.2020.1759699	SCOPUS	off topic	no
measurement of heart rate using the withings scanwatch device during free-living activities: validation study	2022	10.2196/34280	SCOPUS	off topic	no
measurement of heartbeats for well-being assessment using photoplethysmographic signals	2020	10.1007/978-981-15-7031-5_46	SCOPUS	hardware	no
measuring heart rate and heart rate variability with smartphone camera	2021	10.1109/ mdm52706.2021.00049	SCOPUS	off topic	no
measuring heart rate during physical exercise by subspace decomposition and kalman smoothing	2018	10.1109/ tim.2017.2770818	SCOPUS	inaccessible dataset	no
measuring heart rate variability using commercially available devices in healthy children: a validity and reliability study	2020	10.3390/ ejihpe10010029	SCOPUS	off topic	no
mechanical pain assessment through parameters derived from photoplethysmographic (ppg) signals: a pilot study	2020	10.1007/978-3-030-42517-3_13	SCOPUS	off topic	no
methods for analyzing infant heart rate variability: a preliminary study	2023	10.1002/bdr2.2177	SCOPUS	off topic	no

modified squirrel algorithm for fault tolerance using photoplethysmography in iot applications	2021	10.1109/iceca52323.2021.9676083	SCOPUS	off topic	no
modtrap: improved heart rate tracking and preprocessing of motion-corrupted photoplethysmographic data for personalized healthcare	2020	10.1016/j.bspc.2019.101676	SCOPUS	inaccessible dataset	no
modulation model of the photoplethysmography signal for vital sign extraction	2021	10.1109/jbhi.2020.3013811	SCOPUS	off topic	no
mombat: heart rate monitoring from face video using pulse modeling and bayesian tracking	2020	10.1016/j.compbiomed.2020.103813	SCOPUS	off topic	no
monitoring of heart rate and inter-beat intervals with wrist plethysmography in patients with atrial fibrillation	2018	10.1088/1361-6579/aac9a9	SCOPUS	off topic	no
monitoring of heart rate, blood oxygen saturation, and blood pressure using a smartphone	2020	10.1016/j.bspc.2020.101928	SCOPUS	off topic	no
motion artifact canceling ppg heart rate sensor based on an adaptive filter algorithm with variable tap length	2020	10.1109/embc44109.2020.9176715	SCOPUS	denoising	no
motion artifact cancellation in wearable photoplethysmography using gyroscope	2019	10.1109/jsen.2018.2879970	SCOPUS	denoising	no
motion artifact mitigation for wearable pulse oximetry	2018	10.1109/bsn.2018.8329662	SCOPUS	denoising	no
motion artifact reduction for wrist-worn photoplethysmograph sensors based on different wavelengths	2019	10.3390/s19030673	SCOPUS	denoising	no
motion artifact reduction from ppg signals during intense exercise using filtered x-lms	2017	10.1109/iscas.2017.8050418	SCOPUS	denoising	no
motion artifact reduction in wearable photoplethysmography based on multi-channel sensors with multiple wavelengths	2020	10.3390/s20051493	SCOPUS	denoising	no
motion artifact removal and feature extraction from ppg signals using efficient signal processing algorithms	2018	10.1109/icacci.2018.8554599	SCOPUS	denoising	no
motion artifact removal for ppg signals based on accurate fundamental frequency estimation and notch filtering	2018	10.1109/embc.2018.8512878	SCOPUS	denoising	no
motion artifacts detection algorithm for non contact photoplethysmography using five tap filter	2019		SCOPUS	off topic	no

motion-robust multimodal heart rate estimation using bcg fused remote-ppg with deep facial roi tracker and pose constrained kalman filter	2021	10.1109/tim.2021.3060572	SCOPUS	off topic	no
movement, sweating, and contact pressure as sources of heart rate inaccuracy in wearable devices	2022	10.22489/cinc.2022.232	SCOPUS	off topic	no
movement, sweating, and contact pressure as sources of heart rate inaccuracy in wearable devices	2022	10.22489/cinc.2022.237	SCOPUS	off topic	no
multi-modal physiological sensing on the upper arm	2022	10.1117/12.2606468	SCOPUS	multichannel	no
multi-mode particle filtering methods for heart rate estimation from wearable photoplethysmography	2019	10.1109/tbme.2019.2895685	SCOPUS	denoising	no
multi-physiological parameters integrated medical system for home healthcare application	2021	10.1109/asicon52560.2021.9620524	SCOPUS	off topic	no
multi-sensor wearable health device framework for real-time monitoring of elderly patients using a mobile application and high-resolution parameter estimation	2022	10.3389/fnhum.2021.750591	SCOPUS	off topic	no
multibiometric system based on face video and heart rate estimation	2021	10.1109/iwbf50991.2021.9465081	SCOPUS	off topic	no
mutual dynamics of synchronization of low-frequency oscillations in circulation vegetative regulation and indicators of variability of the heart rhythm in patients after operations with artificial circulation in the early postoperative period	2018	10.18484/2305-0047.2018.1.24	SCOPUS	off topic	no
mvpd: a multimodal video physiology database for rppg	2023	10.1109/seai59139.2023.10217565	SCOPUS	off topic	no
nas-ppg: ppg-based heart rate estimation using neural architecture search	2021	10.1109/jsen.2021.3073047	SCOPUS	multichannel	no
new ways to estimate blood pressure, heartrate variability and spo2via smartphone camera - proof of concept	2020	10.1145/3448891.3448951	SCOPUS	off topic	no
nocturnal pulse wave amplitude attenuations are associated with long-term cardiovascular events	2023	10.1016/j.ijcard.2023.05.047	SCOPUS	off topic	no
noise-robust heart rate estimation algorithm from photoplethysmography signal with low computational complexity	2019	10.1155/2019/6283279	SCOPUS	inaccessible dataset	no

non-contact fingertip microwave plethysmography based on near-field sensing with super-regenerative oscillator	2021	10.1109/ims19712.2021.9574888	SCOPUS	off topic	no
non-contact heart rate and blood pressure estimations from video analysis and machine learning modelling applied to food sensory responses: a case study for chocolate	2018	10.3390/s18061802	SCOPUS	off topic	no
non-contact heart rate measurement from face video sequences using sift and fastica	2024	10.1504/ijbet.2024.138617	SCOPUS	off topic	no
non-contact heart rate measurement from facial video data using a 2d-vmd scheme	2022	10.1109/jsen.2022.3169963	SCOPUS	off topic	no
non-contact heart rate monitoring analysis from various distances with different face regions	2017	10.11591/ijece.v7i6.pp3030-3036	SCOPUS	off topic	no
non-contact heart rate monitoring in neonatal intensive care unit using rgb camera	2020	10.1109/embc44109.2020.9175651	SCOPUS	off topic	no
non-contact ppg signal and heart rate estimation with multi-hierarchical convolutional network	2023	10.1016/j.patcog.2023.109421	SCOPUS	off topic	no
non-invasive blood pressure and heart rate sensing using photoplethysmogram sensor	2021	10.1109/cspa52141.2021.9377302	SCOPUS	hardware	no
non-invasive blood pressure monitoring integrated in a smart watch form factor	2018		SCOPUS	off topic	no
non-invasive cuff free blood pressure and heart rate measurement from photoplethysmography (ppg) signal using machine learning	2024	10.1007/s11277-024-11070-x	SCOPUS	hardware	no
non-invasive pulse arrival time is associated with cardiac index in pediatric heart transplant patients with normal ejection fraction	2024	10.1088/1361-6579/ad61b9	SCOPUS	off topic	no
noninvasive blood oxygen, heartbeat rate, and blood pressure parameter monitoring by photoplethysmography signals	2022	10.1016/j.heliyon.2022.e11698	SCOPUS	hardware	no
noninvasive heart rate and blood glucose level estimation using photoplethysmography	2021	10.1109/icict4sd50815.2021.9396849	SCOPUS	off topic	no

novel tailoring algorithm for abrupt motion artifact removal in photoplethysmogram signals	2017	10.1007/s13534-017-0037-0	SCOPUS	denoising	no
oil recovery from fractured reservoirs using in situ and preformed particle gels in micromodel structures	2019	10.1007/s13202-019-0627-8	SCOPUS	off topic	no
on the use of wavelet transform based adaptive filtering for de-noising of pulse oximeter signals	2021	10.1109/i2mtc50364.2021.9459833	SCOPUS	denoising	no
on-device signal quality guided and embedded physiologic information for high fidelity continuous ppg compression	2024	10.1109/tim.2024.3351256	SCOPUS	off topic	no
optical measurement of tissue perfusion changes as an alternative to electrocardiography for heart rate monitoring in atlantic salmon (salmo salar)	2021	10.1186/s40317-021-00264-w	SCOPUS	off topic	no
optimizing performance of reflectance-based organic photoplethysmogram (ppg) sensor	2018	10.1117/12.2321060	SCOPUS	off topic	no
opto-physiological monitoring senor to enable accurate physiological monitoring in real time and at any time	2024	10.1117/12.3001452	SCOPUS	off topic	no
p2e-lgan: ppg to ecg reconstruction methodology using lstm based generative adversarial network	2024	10.1109/iscas58744.2024.10558493	SCOPUS	off topic	no
p2e-wgan: ecg waveform synthesis from ppg with conditional wasserstein generative adversarial networks	2021	10.1145/3412841.3441979	SCOPUS	off topic	no
parameter optimization of motion artifact canceling ppg-based heart rate sensor by means of cross validation	2017	10.1109/ismict.2017.7891771	SCOPUS	denoising	no
parhelia: particle filter-based heart rate estimation from photoplethysmographic signals during physical exercise	2018	10.1109/tbme.2017.2697911	SCOPUS	multichannel	no
pathological and oncological outcomes of pylorus-preserving versus conventional distal gastrectomy in early gastric cancer: a systematic review and meta-analysis	2022	10.1186/s12957-022-02766-0	SCOPUS	off topic	no
pbs qd-coated si micro-hole array/graphene vdw schottky near-infrared photodiode for ppg heart rate measurement	2023	10.3390/s23167214	SCOPUS	hardware	no

pbvi for optimal photoplethysmography noise filter selection using human activity recognition observations for improved heart rate estimation on multi-sensor systems	2024	10.1115/1.4065219	SCOPUS	denoising	no
peak frequency tracking versus outlier rejection for photoplethysmographic heart rate sensing during exercise	2020	10.1109/ismict48699.2020.9152726	SCOPUS	inaccessible dataset	no
performance analysis of adaptive filter and machine learning algorithms for heart rate estimation using ppg signal	2021	10.1109/ict4da53266.2021.9672242	SCOPUS	inaccessible dataset	no
personalized stress monitoring using wearable sensors in everyday settings	2021	10.1109/embc46164.2021.9630224	SCOPUS	off topic	no
photoplethysmograhic sensors, potential and limitations: is it time for regulation? a comprehensive review	2023	10.1016/j.measurement.2023.113150	SCOPUS	review	no
photoplethysmographic waveform analysis for autonomic reactivity assessment in depression	2021	10.1109/tbme.2020.3025908	SCOPUS	off topic	no
photoplethysmography and inertial sensors in wearable devices for healthcare: multimodal signal processing for increasing accuracy	2024	10.1201/9781003346678-5	SCOPUS	multichannel	no
photoplethysmography heart rate monitoring: state-of-the-art design	2021	10.4018/ijehmc.20210501.oa2	SCOPUS	review	no
photoplethysmography wave morphology in patients with atrial fibrillation	2023	10.1088/1361-6579/acc725	SCOPUS	off topic	no
photoplethysmography-based heart action monitoring using a growing multilayer network	2023	10.1109/jsen.2022.3228517	SCOPUS	off topic	no
physiological measures in vr experiments: some aspects of plethysmogram and heart rate	2023	10.1007/978-3-031-35132-7_32	SCOPUS	off topic	no
physiological sensing on the upper arm with a wireless multi-modal wearable	2024	10.1117/12.3003163	SCOPUS	off topic	no
physiopatch: a multimodal and adaptable wearable patch for cardiovascular and cardiopulmonary assessment	2024	10.1109/jsen.2024.3403846	SCOPUS	off topic	no
pilot study assessing the influence of skin type on the heart rate measurements obtained by photoplethysmography with the apple watch	2019	10.1007/s10916-019-1325-2	SCOPUS	hardware	no

portable and real-time iot-based healthcare monitoring system for daily medical applications	2023	10.1109/ tcss.2022.3207562	SCOPUS	off topic	no
power spectral densities of nocturnal pulse oximetry signals differ in osa patients with and without daytime sleepiness	2020	10.1016/ j.sleep.2020.07.015	SCOPUS	off topic	no
power-aware heart rate monitoring using particle filters	2021	10.1109/ islped52811.2021.95 02471	SCOPUS	missing technical details	no
pp-net: a deep learning framework for ppg-based blood pressure and heart rate estimation	2020	10.1109/ jsen.2020.2990864	SCOPUS	multichannel	no
ppg derived heart rate estimation during intensive physical exercise	2019	10.1109/ access.2019.291314 8	SCOPUS	inaccessible dataset	no
ppg neurons in the nucleus of the solitary tract modulate heart rate but do not mediate glp-1 receptor agonist-induced tachycardia in mice	2020	10.1016/ j.molmet.2020.10102 4	SCOPUS	animal	no
ppg-based heart rate estimation using wiener filter, phase vocoder and viterbi decoding	2017	10.1109/ icassp.2017.795230 9	SCOPUS	inaccessible dataset	no
ppg-based heart rate estimation with efficient sensor sampling and learning models	2022	10.1109/hpcc-dss-smartcity-dependsys57074.20 22.00294	SCOPUS	inaccessible dataset	no
ppg-based non-invasive methodologies for pervasive monitoring of vitals: bp and hr	2021	10.1007/978-981-16-5324-7_3	SCOPUS	off topic	no
ppg-gan: an adversarial network to de-noise ppg signals during physical activity	2022	10.1109/ healthcom54947.202 2.9982757	SCOPUS	denoising	no
ppgnet: deep network for device independent heart rate estimation from photoplethysmogram	2019	10.1109/ embc.2019.8856989	SCOPUS	inaccessible dataset	no
precision heart rate estimation using a ppg sensor patch equipped with new algorithms of pre-quality checking and hankel decomposition	2023	10.3390/s23136180	SCOPUS	inaccessible dataset	no
prediction of noise-type and its elimination from photoplethysmograph signal	2024	10.1109/ ciec59440.2024.104 68270	SCOPUS	denoising	no

preheat: precision heart rate monitoring from intense motion artifact corrupted ppg signals using constrained rls and wavelets	2017	10.1016/ j.bspc.2017.05.010	SCOPUS	inaccessible dataset	no
preliminary assessment of the samsung galaxy watch 5 accuracy for the monitoring of heart rate and heart rate variability parameters	2024	10.1007/978-3-031-49062-0_3	SCOPUS	off topic	no
pressurized mud cap drilling used to drill and complete carbonate gas reservoir with poor injectivity and severe dynamic losses offshore malaysia	2022	10.2118/210550-ms	SCOPUS	off topic	no
principles of construction of hybrid microsystems for biomedical applications; [принципи побудови гібридних мікросистем для біомедичних застосувань]	2022	10.15330/ pcss.23.4.776-784	SCOPUS	hardware	no
privacy protected contactless cardio-respiratory monitoring using defocused cameras during sleep	2023	10.1109/ healthcom56612.2023.10472346	SCOPUS	off topic	no
process design for optimized respiration identification based on heart rate variability for efficient respiratory sinus arrhythmia biofeedback	2022	10.3390/ ijerph19042087	SCOPUS	off topic	no
profiling the propagation of error from ppg to hrv features in a wearable physiological-monitoring device	2018	10.1049/ htl.2017.0039	SCOPUS	inaccessible dataset	no
prognostic implications of prestent pullback pressure gradient and poststent quantitative flow ratio in patients undergoing percutaneous coronary intervention	2022	10.1161/ jaha.121.024903	SCOPUS	off topic	no
q-ppg: energy-efficient ppg-based heart rate monitoring on wearable devices	2021	10.1109/ tbcas.2021.3122017	SCOPUS	multichannel	yes
quantifying the accuracy of inter-beat intervals acquired from consumer-grade photoplethysmography wristbands using an electrocardiogram-aided information-based similarity approach	2024	10.1088/1361-6579/ad2c14	SCOPUS	not accessible	no
rapid vital sign extraction for real-time opto-physiological monitoring at varying physical activity intensity levels	2023	10.1109/ jbhi.2023.3268240	SCOPUS	off topic	no
real time and anytime opto-physiological monitoring	2023	10.1117/12.3006570	SCOPUS	not accessible	no
real-time automatic peaks and onsets detection of photoplethysmographic signals	2018	10.1007/978-981-10-7251-2_9	SCOPUS	not accessible	no
real-time dynamic data analysis model based on wearable smartband	2018	10.1007/978-3-319-69096-4_61	SCOPUS	off topic	no

real-time heartbeat sensing with face video using a webcam and opencv	2023	10.1109/cises58720.2023.10183446	SCOPUS	off topic	no
real-time hr estimation from wrist ppg using binary lstms	2019	10.1109/biocas.2019.8918726	SCOPUS	inaccessible dataset	no
real-time photoplethysmographic heart rate measurement using deep neural network filters	2021	10.4218/etrij.2020-0394	SCOPUS	off topic	no
real-time pulse oximetry extraction using a lightweight algorithm and a task pipeline scheme	2021	10.1109/mocast52088.2021.9493400	SCOPUS	hardware	no
real-time quality index to control data loss in real-life cardiac monitoring applications	2021	10.3390/s21165357	SCOPUS	off topic	no
real-time robust heart rate estimation from wrist-type ppg signals using multiple reference adaptive noise cancellation	2018	10.1109/jbhi.2016.2632201	SCOPUS	inaccessible dataset	no
real-time video-based heart and respiration rate monitoring	2021	10.1109/naecon49338.2021.9696378	SCOPUS	off topic	no
real-world heart rate norms in the health eheart study	2019	10.1038/s41746-019-0134-9	SCOPUS	off topic	no
recent innovations and improvements in remote heart rate and heart disease measuring methods using rgb camera	2023	10.1109/iccpct58313.2023.10245666	SCOPUS	off topic	no
recommendations for determining the validity of consumer wearable heart rate devices: expert statement and checklist of the interlive network	2021	10.1136/bjsports-2020-103148	SCOPUS	off topic	no
reconstruction of corrupted photoplethysmography signals to facilitate continuous monitoring	2023	10.22489/cinc.2023.121	SCOPUS	denoising	no
reconstruction of corrupted photoplethysmography signals using recursive generative adversarial networks	2024	10.1109/tim.2023.3335524	SCOPUS	denoising	no
reduced complexity algorithm for heart rate monitoring from ppg signals using automatic activity intensity classifier	2019	10.1016/j.bspc.2019.04.026	SCOPUS	inaccessible dataset	no
reduced well construction time with accurate geostopping in harsh drilling conditions: case study from kuwait	2021	10.2118/202077-ms	SCOPUS	off topic	no

reference signal less fourier analysis based motion artifact removal algorithm for wearable photoplethysmography devices to estimate heart rate during physical exercises	2022	10.1016/ j.compbiomed.2021. 105081	SCOPUS	INCLUDED	yes
reflectance-based monolithic organic pulsemeter device for measuring photoplethysmogram signal	2018	10.1109/ i2mtc.2018.8409873	SCOPUS	off topic	no
relaxation assessment based on heart rate variability and heart rate using photoplethsmograms	2022	10.1109/ispce-asia57917.2022.997 0859	SCOPUS	hardware	no
reliability assessment of telemedicine data by analyzing photoplethysmography with deep neural network technology	2021	10.3795/ksme-b.2021.45.5.261	SCOPUS	off topic	no
reliability estimation and filtering of heart rate measurement using inertial sensor during exercise	2022	10.18494/sam3969	SCOPUS	inaccessible dataset	no
remote health monitoring system for the estimation of blood pressure, heart rate, and blood oxygen saturation level	2023	10.1109/ jsen.2023.3235977	SCOPUS	hardware	no
remote heart rate measurement using plethysmographic wave analysis	2023	10.1007/978-3-031-29313-9_23	SCOPUS	off topic	no
remote heart rate monitoring in smart environments from videos with self-supervised pretraining	2024	10.1109/ jiot.2023.3327623	SCOPUS	off topic	no
remote monitoring of atrial fibrillation recurrence using mhealth technology (remote-af)	2024	10.1093/ehjdh/ ztae011	SCOPUS	off topic	no
remote photoplethysmography and heart rate estimation by dynamic region of interest tracking	2022	10.1109/ embc48229.2022.98 71722	SCOPUS	off topic	no
remote photoplethysmography using nonlinear mode decomposition	2018	10.1109/ icassp.2018.846253 8	SCOPUS	off topic	no
remote real-time heart rate monitoring with recursive motion artifact removal using ppg signals from a smartphone camera	2023	10.1007/s11042-023-14399-w	SCOPUS	off topic	no
removal of motion artifacts in photoplethysmograph sensors during intensive exercise for accurate heart rate calculation based on frequency estimation and notch filtering	2019	10.3390/s19153312	SCOPUS	denoising	no

respiratory rate estimations using three respiratory-induced variations on photoplethysmogram	2021	10.1109/ iceei52609.2021.961 1130	SCOPUS	off topic	no
resting and postexercise heart rate detection from fingertip and facial photoplethysmography using a smartphone camera: a validation study	2017	10.2196/ mhealth.7275	SCOPUS	off topic	no
resumption and progression of meiosis and circulating levels of steroids and prostaglandin f2α of piaractus mesopotamicus induced by hypophysation with prostaglandin f2α	2021	10.1111/are.14957	SCOPUS	off topic	no
rgb and near-infrared light reflectance/transmittance photoplethysmography for measuring heart rate during motion	2020	10.1109/ access.2020.299043 8	SCOPUS	inaccessible dataset	no
risk factors of poor prognosis in patients with pyrrolidine alkaloid-induced hepatic sinusoidal obstruction syndrome after transjugular intrahepatic portosystemic shunt	2021	10.1007/s12072-020-10126-x	SCOPUS	off topic	no
robust and computationally efficient approach for heart rate monitoring using photoplethysmographic signals during intensive physical excercise	2017	10.1109/ mercon.2017.79804 97	SCOPUS	inaccessible dataset	no
robust de-noising technique for accurate heart rate estimation using wrist-type ppg signals	2020	10.1109/ jsen.2020.2982540	SCOPUS	denoising	no
robust heart rate estimation during physical exercise using photoplethysmographic signals	2018	10.1109/ embc.2018.8512405	SCOPUS	inaccessible dataset	no
robust heart rate estimation from ppg signals with intense motion artifacts using cascade of adaptive filter and recurrent neural network	2019	10.1109/ tencon.2019.892969 2	SCOPUS	inaccessible dataset	no
robust heart rate estimation using wrist-type photoplethysmographic signals during physical exercise: an approach based on adaptive filtering	2017	10.1088/1361-6579/aa506e	SCOPUS	not accessible	no
robust heart rate monitoring by a single wrist-worn accelerometer based on signal decomposition	2021	10.1109/ jsen.2021.3075109	SCOPUS	denoising	no
robust heart rate monitoring for quasi-periodic motions by wrist-type ppg signals	2020	10.1109/ jbhi.2019.2912708	SCOPUS	inaccessible dataset	no
robust motion artefact resistant circuit for calculation of mean arterial pressure from pulse transit time	2017	10.1109/ embc.2017.8037624	SCOPUS	denoising	no

robust ppg-based ambulatory heart rate tracking algorithm	2020	10.1109/ embc44109.2020.91 75346	SCOPUS	INCLUDED	yes
security framework for physiological signals using auto encoder	2020	10.5373/jardcs/ v12sp1/20201107	SCOPUS	off topic	no
self-rppg: learning the optical & physiological mechanics of remote photoplethysmography with self-supervision	2022	10.1145/3551455.35 59609	SCOPUS	off topic	no
sfst: a robust framework for heart rate monitoring from photoplethysmography signals during physical activities	2017	10.1016/ j.bspc.2016.12.005	SCOPUS	inaccessible dataset	no
signal quality assessment for wearable multichannel photoplethysmography signals	2023	10.1109/ apsipaasc58517.202 3.10317377	SCOPUS	multichannel	no
signal quality index based on template cross-correlation in multimodal biosignal chair for smart healthcare	2021	10.3390/s21227564	SCOPUS	off topic	no
simultaneous measurement and correlation of ppg signals taken from two different body parts for enhanced biometric security via two-level authentication	2017	10.1145/3137003.31 37004	SCOPUS	off topic	no
sirs prediction method based on ppg signal	2019	10.1117/12.2559762	SCOPUS	off topic	no
sleep apnea screening based on photoplethysmography data from wearable bracelets using an information-based similarity approach	2021	10.1016/ j.cmpb.2021.106442	SCOPUS	off topic	no
sleep monitoring with intraorally measured photoplethysmography (ppg) signals	2022	10.1109/ sensors52175.2022. 9967075	SCOPUS	off topic	no
sleep/wake classification via remote ppg signals	2019	10.1109/ embc.2019.8857097	SCOPUS	off topic	no
smart garment fabrics to enable non-contact opto-physiological monitoring	2018	10.3390/ bios8020033	SCOPUS	off topic	no
smart mandibular advancement device for intraoral monitoring of cardiorespiratory parameters and sleeping postures	2021	10.1109/ tbcas.2021.3065824	SCOPUS	off topic	no
smartphone-based photoplethysmographic imaging for heart rate monitoring	2017	10.1080/03091902.2 017.1299233	SCOPUS	off topic	no

smartwatch ppg peak detection method for sinus rhythm and cardiac arrhythmia	2019	10.1109/ embc.2019.8857325	SCOPUS	inaccessible dataset	no
sources of inaccuracy in photoplethysmography for continuous cardiovascular monitoring	2021	10.3390/ bios11040126	SCOPUS	off topic	no
spot measurement of heart rate based on morphology of photoplethysmographic (ppg) signals	2017	10.1080/03091902.2 016.1223198	SCOPUS	not accessible	no
srppg: semi-supervised adversarial learning for remote photoplethysmography with noisy data	2023	10.1109/ smartcomp58114.20 23.00021	SCOPUS	off topic	no
state-dependent gaussian kernel-based power spectrum modification for accurate instantaneous heart rate estimation	2019	10.1371/ journal.pone.021501 4	SCOPUS	multichannel	no
stochastic modeling based nonlinear bayesian filtering for photoplethysmography denoising in wearable devices	2020	10.1109/ tii.2020.2988097	SCOPUS	denoising	no
stress detection using cnn on the wesad dataset	2024	10.1109/ esic60604.2024.104 81604	SCOPUS	off topic	no
strong robustness heart rate estimation using discrete fourier transform and personality heart rate characteristic	2017	10.23919/ chicc.2017.8028794	SCOPUS	inaccessible dataset	no
stsr: spectro-temporal super-resolution analysis of a reference signal less photoplethysmogram for heart rate estimation during physical activity	2022	10.1109/ tim.2022.3192831	SCOPUS	inaccessible dataset	no
supervised heart rate tracking using wrist-type photoplethysmographic (ppg) signals during physical exercise without simultaneous acceleration signals	2017	10.1109/ globalsip.2016.7906 025	SCOPUS	inaccessible dataset	no
sustained attention detection system in learning environments	2020	10.1109/ epe50722.2020.930 5027	SCOPUS	off topic	no
synthesis of photoplethysmograph signals from noises with desired heart and respiration rates	2024	10.1109/ ciec59440.2024.104 68279	SCOPUS	denoising	no
targeted decrease of portal hepatic pressure gradient improves ascites control after tips	2023	10.1002/hep.32676	SCOPUS	off topic	no
task-evoked pulse wave amplitude tracks cognitive load	2023	10.1038/s41598-023-48917-5	SCOPUS	off topic	no

taylor-fourier analysis of photoplethysmography signals for heart rate measurement	2024	10.1109/i2mtc60896.2024.10560902	SCOPUS	inaccessible dataset	no
the accuracy of heartbeat detection using photoplethysmography technology in cardiac patients	2021	10.1016/j.jelectrocard.2021.06.009	SCOPUS	inaccessible dataset	no
the accuracy of wrist-worn photoplethysmogram–measured heart and respiratory rates in abdominal surgery patients: observational prospective clinical validation study	2023	10.2196/40474	SCOPUS	inaccessible dataset	no
the biophysical parameter measurements from ppg signal	2017	10.1142/s021951941740005x	SCOPUS	off topic	no
the impact of healthy pregnancy on features of heart rate variability and pulse wave morphology derived from wrist-worn photoplethysmography	2023	10.1038/s41598-023-47980-2	SCOPUS	off topic	no
the multi wavelength arrayed flexible ppg sensing patch for to estimate heart rate and blood oxygen	2020	10.1115/isps2020-1923	SCOPUS	not accessible	no
the prediction of atherosclerosis index based on photoplethysmograph	2021	10.1155/2021/2234514	SCOPUS	off topic	no
the real-time image sequences-based stress assessment vision system for mental health	2024	10.3390/electronics13112180	SCOPUS	off topic	no
the relationship between stress levels measured by a questionnaire and the data obtained by smart glasses and finger pulse oximeters among polish dental students	2021	10.3390/app11188648	SCOPUS	off topic	no
the study on workload analysis of construction workers operating at elevated jobsite using wearable physiological monitor	2024	10.1080/02533839.2024.2334208	SCOPUS	off topic	no
the use of wrist emg increases the ppg heart rate accuracy in smartwatches	2022	10.1109/jsen.2022.3219297	SCOPUS	inaccessible dataset	no
time-varying assessment of heart rate variability parameters using respiratory information	2017	10.1016/j.compbiomed.2017.07.022	SCOPUS	off topic	no
toward a universal bcg validation using a mechanical emulator	2024	10.1109/i2mtc60896.2024.10560923	SCOPUS	off topic	no

toward nonintrusive camera-based heart rate variability estimation in the car under naturalistic condition	2022	10.1109/ jiot.2021.3131742	SCOPUS	off topic	no
towards a machine learning-based digital twin for non-invasive human bio-signal fusion	2022	10.3390/s22249747	SCOPUS	off topic	no
towards low-power heart rate estimation based on user's demographics and activity level for wearables	2023	10.1109/ icassp49357.2023.10095339	SCOPUS	inaccessible dataset	no
towards quantifying stress in patients with a history of myocardial infarction: validating ecg-derived patch features	2023	10.1109/ embc40787.2023.10340614	SCOPUS	off topic	no
transjugular intrahepatic portosystemic shunt placement in patients with schistosomiasis-induced liver fibrosis	2019	10.1007/s00270-019-02295-6	SCOPUS	off topic	no
trends in heart rate and heart rate variability during pregnancy and the 3-month postpartum period: continuous monitoring in a free-living context	2022	10.2196/33458	SCOPUS	off topic	no
ultra-low-invasion non-damaging spacer system and nano-silicate based high-crush resistant cement cures lost circulation and established well integrity: a case study	2022	10.2118/211531-ms	SCOPUS	off topic	no
unobtrusive photoplethysmographic monitoring under the foot sole while in a standing posture	2018	10.3390/s18103239	SCOPUS	off topic	no
use of a smart watch for early detection of paroxysmal atrial fibrillation: validation study	2020	10.2196/14857	SCOPUS	off topic	no
use of heart rate variability and photoplethysmograph-derived parameters as assessment signals of radiofrequency therapy efficacy for chronic pain	2017	10.1111/papr.12536	SCOPUS	off topic	no
using imaging photoplethysmography (ippg) signal for blood pressure estimation	2020	10.1109/ mvip49855.2020.9116902	SCOPUS	off topic	no
using rear smartphone cameras as sensors for measuring heart rate variability	2021	10.1109/ access.2021.3054065	SCOPUS	off topic	no
utility of photoplethysmography for heart rate estimation among inpatients	2018	10.1111/imj.13777	SCOPUS	off topic	no
validation of heart rate extracted from wrist-based photoplethysmography in the perioperative setting: prospective observational study	2021	10.2196/27765	SCOPUS	off topic	no

validity of resting heart rate derived from contact-based smartphone photoplethysmography compared with electrocardiography: a scoping review and checklist for optimal acquisition and reporting	2024	10.3389/fdgth.2024.1326511	SCOPUS	off topic	no
validity of the polar vantage m watch when measuring heart rate at different exercise intensities	2021	10.7717/peerj.10893	SCOPUS	off topic	no
validity of the wrist-worn polar vantage v2 to measure heart rate and heart rate variability at rest	2022	10.3390/s22010137	SCOPUS	off topic	no
var-hr: noncontact heart rate measurement using an rgb camera based on adaptive region selection with singular value decomposition	2024	10.1109/lsens.2024.3375892	SCOPUS	off topic	no
variational mode decomposition-based heart rate estimation using wrist-type photoplethysmography during physical exercise	2018	10.1109/icpr.2018.8545685	SCOPUS	inaccessible dataset	no
variations of heart rate, pulse arrival time and blood pressure in a versatile laboratory protocol	2018	10.1109/embc.2018.8512876	SCOPUS	off topic	no
video-based hr measurement using adaptive facial regions with multiple color spaces	2024	10.1016/j.bbe.2023.12.001	SCOPUS	off topic	no
video-based human heart rate measurement using joint blind source separation	2017	10.1016/j.bspc.2016.08.020	SCOPUS	off topic	no
video-based physiological measurement using 3d central difference convolution attention network	2021	10.1109/ijcb52358.2021.9484405	SCOPUS	off topic	no
video-based pulse rate variability measurement using periodic variance maximization and adaptive two-window peak detection	2020	10.3390/s20102752	SCOPUS	off topic	no
wavelet-based embedded algorithm for respiratory rate estimation from ppg signal	2017	10.1016/j.bspc.2017.03.009	SCOPUS	off topic	no
wearable devices made of a wireless vertical-type light-emitting diode package on a flexible polyimide substrate with a conductive layer	2021	10.1021/acsaelm.0c01072	SCOPUS	off topic	no
wearable ear blood oxygen saturation and pulse measurement system based on ppg	2018	10.1109/smartworld.2018.00054	SCOPUS	off topic	no
wearable heart rate monitor technology accuracy in research: a comparative study between ppg and ecg technology	2017	10.1177/1541931213601804	SCOPUS	off topic	no

wearable multichannel photoplethysmography framework for heart rate monitoring during intensive exercise	2018	10.1109/ jsen.2018.2801385	SCOPUS	inaccessible dataset	no
wearable multisensor ring-shaped probe for assessing stress and blood oxygenation: design and preliminary measurements	2023	10.3390/ bios13040460	SCOPUS	off topic	no
wearable ppg sensor with bluetooth data transmission for continual measurement in low magnetic field environment	2021	10.23919/ ae51540.2021.9542 901	SCOPUS	inaccessible dataset	no
wearable ring-shaped biomedical device for physiological monitoring through finger-based acquisition of electrocardiographic, photoplethysmographic, and galvanic skin response signals: design and preliminary measurements	2024	10.3390/ bios14040205	SCOPUS	off topic	no
where can exercisers sense heart rates accurately and comfortably on their bodies?	2019	10.1109/ ismict.2019.8743675	SCOPUS	off topic	no
wi-fi based integrated system for the monitoring of heart rate and peripheral capillary oxygen saturation	2020	10.1109/ meco49872.2020.91 34347	SCOPUS	off topic	no
wireless heart rate and oxygen saturation monitor	2019	10.1063/1.5095913	SCOPUS	inaccessible dataset	no
wireless, non-invasive, wearable device for continuous remote monitoring of hemodynamic parameters in a swine model of controlled hemorrhagic shock	2020	10.1038/s41598-020-74686-6	SCOPUS	off topic	no
wrist ballistocardiography and invasively recorded blood pressure in healthy volunteers during reclining bike exercise	2023	10.3389/ fphys.2023.1189732	SCOPUS	off topic	no
wrist-based photoplethysmography assessment of heart rate and heart rate variability: validation of whoop	2021	10.3390/s21103571	SCOPUS	inaccessible dataset	no
wrist-worn optical and chest strap heart rate comparison in a heterogeneous sample of healthy individuals and in coronary artery disease patients	2018	10.1186/s13102-018-0098-0	SCOPUS	off topic	no
wristband for bio-signal monitoring	2022	10.1109/ estc55720.2022.993 9541	SCOPUS	off topic	no
your blush gives you away: detecting hidden mental states with remote photoplethysmography and thermal imaging	2024	10.7717/peerj-cs.1912	SCOPUS	off topic	no

94
